# Seven new species of *Spathidexia* Townsend (Diptera: Tachinidae) reared from caterpillars in Area de Conservación Guanacaste, Costa Rica

**DOI:** 10.3897/BDJ.3.e4597

**Published:** 2015-03-25

**Authors:** AJ Fleming, D. Monty Wood, Daniel Janzen, Winnie Hallwachs, M. Alex Smith

**Affiliations:** ‡ Agriculture Agri-Food Canada, Ottawa, Canada; § University of Pennsylvania, Philadelphia, United States of America; | Department of Integrative Biology, Guelph, Canada

**Keywords:** *
Spathidexia
*, Diptera, Tachinidae, tropical rain forest, tropical dry forest, parasitoid fly, host-specificity, caterpillars

## Abstract

We describe seven new species of *Spathidexia* (Diptera: Tachinidae) reared from Area de Conservación Guanacaste (ACG), northwestern Costa Rica. All were reared from ­various species of ACG caterpillars during an ongoing inventory of caterpillars, their food plants and their parasitoids. By coupling morphology, photographic documentation, life history and molecular data, we provide a clear and concise description of each species. All are known to be previously undescribed as a result of a comprehensive study of the genus by DMW. *Spathidexia atripalpus* **sp. n.**, *Spathidexia juanvialesi* **sp. n.**, *Spathidexia marioburgosi* **sp. n.**, *Spathidexia luisrobertogallegosi* **sp. n.**, *Spathidexia luteola* **sp. n.**, *Spathidexia hernanrodriguezi* **sp. n.** and *Spathidexia aurantiaca* **sp. n.** are all authored and described by Fleming and Wood. *Minthodexiopsis* Townsend is proposed by Wood as a new synonym of *Spathidexia*. A new combination proposed by Wood as a result of the new synonymy is *S. flavicornis* (Brauer & Bergenstamm) **comb. n.**

## Introduction

The tachinid genus *Spathidexia* Townsend 1912 is a small New World genus in the tribe Thelairini of the Dexiinae (Diptera, Tachinidae) (O’Hara and Wood 2004). Thelairini is classified as a small cosmopolitan tribe characterized by: an abdominal mid-dorsal depression that does not reach the hind margin of Tergite1+2, bare eyes, plumose antennae, and bearing 3 pairs of marginal scutellars of which the apicals are often crossed (Crosskey 1984, Mesnil 1939). The tribe Thelairini is known to occur from Hesperiidae, and Arctiidae (Arnaud 1978). The name Spathidexia is derived from the Latin term "spatha", in reference to the females' blade-like ovipositor. Males and females within this genus are sometimes strongly dimorphic with only the females possessing proclinate orbital bristles, or distinctive abdominal coloration and pattern. A complete description of the generic characters was given by Arnaud (1960) (we provide a diagnosis and description of the genus based on this work). The genus *Spathidexia* was erected for a female specimen caught at Melrose Highlands, Mass., in 1908, collected by Mr. D.H. Clemons. The type species *Spathidexia clemonsi* Townsend was named after the original collector of the specimen. The genus remained monotypic until Reinhard 1934 added *Spathidexia cerussata* Reinhard and *Spathidexia rasilis* Reinhard. Arnaud (1960) built on this and raised the number of species to nine, in addition to providing a hint on its biology and extended the generic distribution well into the Neotropics. Today we know that *Spathidexia* contains 17 nominal species, ranging throughout much of the New World, although specimens are still somewhat rare in collections. Wood and Zumbado (2010) attributed seven species to *Spathidexia* living north of Mexico and 12 represented in the Neotropics, with only minimal known overlap between their distributions.

This work aims to build on the existing knowledge and describes seven new species of *Spathidexia*, all reared from caterpillars collected in ACG. In fact, all species of *Spathidexia* reared in ACG have been found to be undescribed species. These species are recognized based on differences in external morphology, and COI (coxI or cytochrome oxidase I) gene sequences (a.k.a. “DNA barcodes”). By coupling COI data with morphological descriptions we are able to show that abdominal markings are not only different between males and females but are also consistent within a species. As such, they are ideal tools for visual species differentiation.

The treatments reported here is focused on placing names on the species reared from ACG, thereby preparing them for later detailed ecological and behavioral accounts and studies that will normally extend across ACG ecological groups, whole ecosystems, and taxonomic assemblages much larger than a genus. However, all ACG *Spathidexia* reared to date in ACG are parasitoids of monocot-eating caterpillars in the Nymphalidae (Satyrinae) or Hesperiidae (Hesperiinae).

## Materials and methods

### Acronyms for depositories

BMNH The Natural History Museum, London, United Kingdom

CNC Canadian National Collection of Insects, Arachnids and Nematodes, Ottawa, Canada

USNM National Museum of Natural History, Washington, D.C., USA

INBio Instituto Nacional de Biodiversidad, Santo Domingo de Heredia, Costa Rica

NHMW Naturhistorisches Museum, Wien, Austria

MZUT Musei di Zoologia, Università di Torino, Torino, Italia

CAS California Academy of Sciences, San Francisco, CA, USA

### Geographic area of the study and rearing intensity

All flies and rearing information described here were found by the 35+ year-old ongoing inventory of the caterpillars, their food plants and their parasitoids of the dry forest, rain forest, cloud forest, and intergrades, in the 125,000+ ha terrestrial portion of Area de Conservación Guanacaste (ACG) in northwestern Costa Rica (Fleming et al. 2014a, Fleming et al. 2014b​,Fernandez-Triana et al. 2014, Janzen and Hallwachs 2011, Janzen et al. 2009, Janzen et al. 2011, Rodriguez et al. 2012, Smith et al. 2012, Smith et al. 2006, Smith et al. 2007, Smith et al. 2008). The tachinid rearing methods are described at http://janzen.bio.upenn.edu/caterpillars/methodology/how/parasitoid_husbandry.htm. The data collected through this initiative contains copious information on parasitoid biology and associated hosts. Among the fly parasitoids encountered, the most species-rich, represented by hundreds of species is the family Tachinidae.

In brief, caterpillars at all instars (and sometimes pupae) are found in the wild by a wide variety of search methods, and reared in captivity on the food plant species on which they were found, until they produce an adult, a parasitoid, or die of other causes. Each caterpillar is documented as an individual, as are the adult parasitic flies.

This inventory has reared about 600,000 wild-caught caterpillars since 1978. All frequencies of parasitism reported here need to be considered against this background inventory. Equally, it is patently obvious that the inventory searches some kinds of vegetation and height off the ground much more thoroughly than others, and it also searches throughout the year. Comparison of reared species of parasitoids with those collected by net or Malaise traps demonstrates that, to date, the caterpillar inventory has encountered well less than half the species of caterpillar parasitoids present in ACG. The largest unsampled void is the upper foliage of the canopy above about 3–4 m above the ground.

### Imaging and dissections

Our descriptions of new species are deliberately brief and only include some differentiating descriptions of body parts and colors that are commonly used in tachinid identification. These brief descriptions are complemented with an extensive series of color photos of every species to illustrate the readily-observed differences among these species.

Habitus photographs were taken using a Canon T3i digital SLR, using a 65mm Macro Photo Lens 1:2.8 (MP–E 65mm), mounted on a microscope track stand (AmScope, Model: TS200) modified to accept a Manfrotto QR 200PL–14 quick release plate. Images were shot in aperture priority, allowing the camera to control shutter speed at f/4.5. Stacks of 40 images were taken at equal distance increments. Illumination was provided with a homemade reflective dome (instruction for dome creation can be found at: http://www.cdfa.ca.gov/plant/ppd/entomology/Dome/kd–200.html) placed over a 144 LED ringlight (AmScope, Model: LED–144–YK).

The photographic series were processed using Photoshop CS6, and digitally stacked with Zerene Stacker Software v1.04. This produced a final composite image maximizing image quality and depth of field.

The terminology used for components of the terminalia (which refers here only to the sclerotized parts of the genitalia, and not to the soft internal structures) and other body parts, follows Cumming and Wood (2009).

All specimens listed as examined are considered paratypes, except for the holotype which is noted separately.

Wherever a specimen label has been cited, the information is presented using the following symbols: /, indicates the end of a line; //, indicates the end of a label. Labels are presented from top (closest to the specimen) to bottom, with any comments about the label being given in square brackets.

### Voucher specimen management

All caterpillars reared from the ACG efforts receive a unique voucher code in the format of yy–SRNP–xxxxx. Any parasitoid emerging from this caterpillar receives the same voucher code, and then if/when later the parasitoid is dealt with individually, it receives a second voucher code unique to it, in the format of DHJPARxxxxxxx. The voucher codes and collateral data assigned to both host and emergent parasitoids are available at http://janzen.bio.upenn.edu/caterpillars/database.lasso. To date, all DHJPARxxxxxxx coded tachinids have had one leg removed for attempted DNA barcoding at the Biodiversity Institute of Ontario (BIO) at the University of Guelph, with all collateral data and all successful barcodes permanently and publically deposited in the Barcode of Life Data System (BOLD, www.boldsystems.org) (Ratnasingham and Hebert 2007), and later migrated to GenBank as well. A neighbor–joining (NJ) tree (Saitou and Nei 1987) for all *Spathidexia* reared and DNA barcoded by this inventory through 2013 is included as Supplemental Appendix 1. The inventory grows continually and new specimens can be found by searching the genus *Spathidexia* in BOLD. Each barcoded specimen also has an accession code, all information for the sequences associated with each individual specimen (including GenBank and BOLD accession) can be retrieved from the Barcode of Life Data System (BOLD) (Ratnasingham and Hebert 2007) via the publicly available dataset: dx.doi.org/10.5883/DS-ASSPATHI.

Inventoried Tachinidae were collected under Costa Rican government research permits issued to DHJ since 1978, and likewise exported under permit by DHJ from Costa Rica to Philadelphia, and then to the final depository in the Canadian National Insect collection in Ottawa, Canada. Tachinid identifications for the inventory were done by DHJ in coordination with a) visual inspection by AJF and DMW, b) DNA barcoding by BIO, MAS, and BOLD, and c) correlation with host caterpillar identifications by DHJ and WH through the inventory itself. Dates of capture of each reared fly in the inventory are the dates of eclosion of the fly, and not the date of capture of the caterpillar. This is because the fly eclosion date is much more representative of the time when that fly species is on the wing than is the time of capture of the caterpillar or (rarely) finding a parasitized pupa. However, the collector listed is the parataxonomist who found the caterpillar, rather than the person who retrieved the newly eclosed fly from its rearing bag or bottle, and processed it by freezing, pinning, labeling and oven–drying. Fly biology and degrees of parasitization by these flies will be the detailed subject of later papers.

### DNA barcoding

DNA barcodes (standardised 5’ region of the mitochondrial cytochrome *c* oxidase I (COI) gene) for all ACG inventory specimens were obtained using DNA extracts prepared from single legs using a glass fibre protocol (Ivanova et al. 2006). Total genomic DNA was re-suspended in 30 μl of dH_2_O, and a 658-bp region near the 5’ terminus of the COI gene was amplified using standard primers (LepF1–LepR1 (Hebert et al. 2004)) following established protocols (Smith et al. 2006, Smith et al. 2007, Smith et al. 2008).

### Generic synonyms of *Spathidexia*

*Spathidexia* Townsend 1912: 110. Type species: *Spathidexia clemonsi* Townsend 1912, by original designation.

*Gymnopalpus* Townsend 1919b: 172. Type species: *Gymnopalpus setipennis* Townsend 1919b, by original designation. Synonymy by Wood 1987: 1265 & O’Hara and Wood 1998: 755.

*Minthodexiopsis* Townsend 1927: 221. Type species: *Minthodexia flavicornis* Brauer and Bergenstamm 1891, by original designation. **Syn. nov.**

*Minthohoughia* Townsend 1919a: 581. Type species: *Minthohoughia cylindrica* Townsend 1919a, by original designation. Synonymy by Guimarães 1971: 95.

*Stenaulacodoria* Townsend 1928: 161. Type species: *Stenaulacodoria spatulata* Townsend 1928, by original designation. Synonymy by Thompson 1963: 494.

### Previously described species included in *Spathidexia*

*antillensis* Arnaud 1960: 6 (*Spathidexia*). Holotype male (USNM) [examined by DMW]. Type locality: Puerto Rico, Mayaguez.

*brasiliensis* Arnaud 1960: 10 (*Spathidexia*). Holotype male (CAS) [examined by DMW]. Type locality: Brazil, Santa Catarina, Nova Teutonia.

*cerussata* Reinhard 1934: 152 (*Spathidexia*). Holotype male (CNC) [examined by DMW]. Type locality: USA, Ohio, Amherst.

*cinereicollis* van der Wulp 1891: 255 (*Thelairodes*). Syntypes, 1 headless male, and 1 female (BMNH) [examined by DMW]. Type localities: Female: Mexico, Guerrero, Amula 6000 feet; Male: Sierra de las Aguas Escondidas 9500 feet. Combination established by Townsend 1915: 366, also mentioned as new combination in Guimarães 1971: 95.

*clemonsi* Townsend 1912: 110 (*Spathidexia*). Holotype female (USNM) [examined by DMW]. Type locality: USA, Massachusetts, Melrose Highlands.

*creolensis* Reinhard 1955: 131 (*Spathidexia*). Holotype female (USNM) [examined by DMW]. Type locality: USA, Florida, Miami.

*cylindrica* Townsend 1919a: 581 (*Minthohoughia*). Holotype female (USNM) [examined by DMW]. Type locality: Peru, Lima. Combination established by Guimarães 1971: 95.

*dicta* Giglio-Tos 1893: 5 (*Plagia*). Holotype male, published as female (MZUT) [examined by DMW]. Type locality: Mexico.

*dunningii* Coquillett 1895: 54 (*Thryptocera*). Lectotype female by fixation of Coquillett 1897: 60 (statement “From the type specimen” is regarded as a lectotype fixation); also by designation of Arnaud 1960: 22. (USNM) [examined by DMW]. Type locality: USA, Illinois, Algonquin. Combination established by Townsend 1915: 366.

*rasilis* Reinhard 1934: 153 (*Spathidexia*). Holotype female (CNC) [examined by DMW]. Type locality: USA, Wisconsin, Madison. Synonymy with *dunningii* established by Arnaud 1960: 5, 18.

*elegans* Reinhard 1964: 37 (*Minthodexiopsis*). Holotype male (CNC) [examined by DMW]. Type locality: USA, Texas, Cameron Co.

*flavicornis* Brauer and Bergenstamm 1891: 376 (*Minthodexia*). Holotype female (NHMW) [not examined]. Type locality: Venezuela. **N. comb.** *

*nexa* Reinhard 1953: 94 (*Spathidexia*). Holotype female (CNC) [examined by DMW]. Type locality: Mexico, Distrito Federal, Ciudad de Mexico.

*niveomarginata* van der Wulp 1890: 200 (*Anisia*). Holotype female (BMNH) [examined by DMW]. Type locality: Mexico, Guerrero, Amula, 6000ft. Combination established by Guimarães 1971: 95.

*pallida* van der Wulp 1891: 255 (*Thelairodes*). Holotype female (BMNH) [examined by DMW]. Type locality: Mexico, Guerrero, Venta de Zopilote, 2800ft.

*reinhardi* Arnaud 1960: 26 (*Spathidexia*). Holotype male (USNM) [examined by DMW]. Type locality: USA, Texas, College Station.

*setipennis* Townsend 1919b: 172 (*Gymnopalpus*). Holotype female (USNM) [examined by DMW]. Type locality: Guatemala, Los Amates. Combination established by Wood 1987: 1265 & O’Hara and Wood 1998: 755.

*spatulata* Townsend 1928: 162 (*Stenaulacodoria*). Holotype female (USNM) [examined by DMW]. Type locality: Peru, Cañete. Combination by Thompson 1963: 494.

*The new combination is proposed by DMW and result from new generic synonymy and the examination of the type material of other nominal species. Type label information is included for those species.

### Diagnosis of the genus *Spathidexia*

Arnaud 1960 provided a detailed description of the genus *Spathidexia*. The genus is characterized as follows: small to medium sized, with narrow parafacial, and bare to sparsely haired eyes, lower margin almost at level of vibrissa. Frons moderately wide in both sexes, with a pair of proclinate orbital bristles in both sexes in many species, some males with reclinate orbital bristles in line with frontal bristles. Frontal bristles reaching to middle of second antennal segment. The antennae descend to near the lower facial margin, with the third segment three times longer than the pedicel. First flagellomere in both sexes parallel sided. Parafacial bare or lightly haired along upper portion. Facial ridge strongly divergent, often posessing supravibrissal bristles along facial ridge. Usually with apical and subapical scutellars crossed at their tips, if subapicals not crossed then these are at least strongly convergent. Abdomen lacking discal bristles; female with flattened rectractile ovipositor which is either long and sharply pointed or short and blunt ended. Wing short and broad without unusual modification in tachinid veination. Vein R_1_ may be bare or haired with vein R_4+5_ haired up to at least crossvein r-m. It is different from its sister taxon *Thelairodes* by the presence of 3–4 meral bristles, with *Thelairodes* possessing 5 or more.

## Taxon treatments

### Spathidexia atripalpus

Fleming & Wood 2015 sp. n.

urn:lsid:zoobank.org:act:0D7D1AFF-015A-48E5-809E-EEA094A3319F

#### Materials

- **Type status:** Holotype. **Occurrence:** occurrenceDetails: http://janzen.sas.upenn.edu; catalogNumber: DHJPAR0048611; recordedBy: D.H. Janzen & W. Hallwachs; individualID: DHJPAR0048611; individualCount: 1; sex: M; lifeStage: adult; preparations: pinned; otherCatalogNumbers: 12-SRNP-30227; **Taxon:** scientificName: Spathidexia atripalpus; phylum: Arthropoda; class: Insecta; order: Diptera; family: Tachinidae; genus: Spathidexia; specificEpithet: atripalpus; scientificNameAuthorship: Fleming & Wood, 2015; **Location:** continent: Central America; country: Costa Rica; countryCode: CR; stateProvince: Guanacaste; county: Area de Conservacion Guanacaste; locality: Sector Pitilla; verbatimLocality: Ingas; verbatimElevation: 580; verbatimLatitude: 11.00311; verbatimLongitude: -85.288; verbatimCoordinateSystem: Decimal; decimalLatitude: 11.00311; decimalLongitude: -85.288; **Identification:** identifiedBy: AJ Fleming; dateIdentified: 2014; **Event:** samplingProtocol: reared from caterpillar of *Magneuptychia libye* (Nymphalidae); verbatimEventDate: 19/Mar/12; **Record Level:** language: en; institutionCode: CNC; collectionCode: Insects; basisOfRecord: Pinned Specimen
- **Type status:** Paratype. **Occurrence:** occurrenceDetails: http://janzen.sas.upenn.edu; catalogNumber: DHJPAR0018619; recordedBy: D.H. Janzen & W. Hallwachs; individualID: DHJPAR0018619; individualCount: 1; lifeStage: adult; preparations: pinned; otherCatalogNumbers: 99-SRNP-2997; **Taxon:** scientificName: Spathidexia atripalpus; phylum: Arthropoda; class: Insecta; order: Diptera; family: Tachinidae; genus: Spathidexia; specificEpithet: atripalpus; scientificNameAuthorship: Fleming & Wood, 2015; **Location:** continent: Central America; country: Costa Rica; countryCode: CR; stateProvince: Guanacaste; county: Area de Conservacion Guanacaste; locality: Sector El Hacha; verbatimLocality: Estacion Los Almendros; verbatimElevation: 290; verbatimLatitude: 11.03226; verbatimLongitude: -85.52776; verbatimCoordinateSystem: Decimal; decimalLatitude: 11.03226; decimalLongitude: -85.52776; **Identification:** identifiedBy: AJ Fleming; dateIdentified: 2014; **Event:** samplingProtocol: reared from caterpillar of *Pierella pallida* (Nymphalidae); verbatimEventDate: 24/Jul/99; **Record Level:** language: en; institutionCode: CNC; collectionCode: Insects; basisOfRecord: Pinned Specimen
- **Type status:** Paratype. **Occurrence:** occurrenceDetails: http://janzen.sas.upenn.edu; catalogNumber: DHJPAR0048578; recordedBy: D.H. Janzen & W. Hallwachs; individualID: DHJPAR0048578; individualCount: 1; lifeStage: adult; preparations: pinned; otherCatalogNumbers: 12-SRNP-33263; **Taxon:** scientificName: Spathidexia atripalpus; phylum: Arthropoda; class: Insecta; order: Diptera; family: Tachinidae; genus: Spathidexia; specificEpithet: atripalpus; scientificNameAuthorship: Fleming & Wood, 2015; **Location:** continent: Central America; country: Costa Rica; countryCode: CR; stateProvince: Guanacaste; county: Area de Conservacion Guanacaste; locality: Sector Pitilla; verbatimLocality: Ingas; verbatimElevation: 580; verbatimLatitude: 11.00311; verbatimLongitude: -85.42041; verbatimCoordinateSystem: Decimal; decimalLatitude: 11.00311; decimalLongitude: -85.42041; **Identification:** identifiedBy: AJ Fleming; dateIdentified: 2014; **Event:** samplingProtocol: reared from caterpillar of *Pareuptychia ocirrhoe* (Nymphalidae); verbatimEventDate: 26/Jan/12; **Record Level:** language: en; institutionCode: CNC; collectionCode: Insects; basisOfRecord: Pinned Specimen

#### Description

**Male** (Fig. 1a, b, c), **head**: Eye bare; frontal vitta dark black, narrowed apically to equal width of the ocellar triangle, face 3 times as wide as frontal vitta; frontal bristles arise no lower than level of pedicel; small black hairs lining the margin of the frontal vitta; antenna black; arista black and plumose, trichia at least 4 times as long as width of base of arista, tapering at apex of arista; proclinate orbital bristles absent; parafrontal entirely silver; parafacial silver; palpi black grey; short row of black supravibrissal hairs along facial margin. **Thorax**: greyish-gold when viewed dorsally with four longitudinal black vittae, these becoming fused post-suturally, appearing as two indistinct blotches covering more than 2/3rds of thorax; three post sutural dorsocentral bristles; scutellum bearing white or yellowish pruinosity over its entirety (occupying 1/2 or more of total area) darkened along its anterior edge; legs black with silvery sheen on anterior tibia. **Wings**: pale smoky greyish in color, vein R_4+5_ setose from node up to r-m, R_1_ bare. **Abdomen**: abdominal tergites dark shiny black, with bright gold bands covering 1/3^rd^ or more of the anterior margins of abdominal tergites T3 and T4, these bands wrapping around to the underside of the abdomen. When viewed laterally bright yellow blotches appear along tergites T1+2, T3 and the anterior margin of T4; T3, T4 and T5 possessing medial marginal bristles. Lateral marginal bristles on T1+2. Small black hairs visible over entire body, in particular visible extending from underside of T1+2.

**Female** (Fig. 1d, e, f), **head**: Frontal vitta dark black, parallel sided apically equal to twice the width of the ocellar triangle, face 3 times as wide as frontal vitta; frontal bristles arise n**​** o lower than level of pedicel; black hairs lining the margin of the frontal vitta; proclinate orbital bristles present; antenna black; arista black and plumose, trichia at least 4 times as long as width of base of arista, tapering at apex of arista; parafrontal entirely silver; parafacial silvery; palpi black, slightly haired along lower surface; short row of black supravibrissal hairs along facial margin. **Thorax**: greyish when viewed dorsally with four longitudinal black vittae, these becoming fused post-suturally, appearing as two indistinct blotches covering just more than 2/3 thorax postsuturally; three post sutural dorsocentral bristles; scutellum bearing white or yellowish pruinosity over its ½ of its entirety; legs black with silvery sheen on anterior femur. **Wings**: pale smoky greyish in color, vein R_4+5 _setose from node up to r-m, R_1_ bare. **Abdomen**: abdominal tergites dark velvety black, bright grayish bands covering at up to 1/2 of tergal surface along anterior margin of T3 and T4; yellow abdominal band wrapping around the abdomen on the posterior margin of T1+2 visible only when viewed laterally; T3, T4 and T5 possessing medial marginal bristles. Lateral marginal bristles on T1+2. Small black hairs visible over entire body, not as hirsute as males but still present, in particular visible extending from anepimeron, and from underside of T1+2.

#### Diagnosis

The presence of black palpi is its strongest diagnostic character, differentiating it from almost all other species. This species differs from *S. atypica*, which also has black(ish) palpi, in having 3 postsutural dorsocentral bristles, bare eyes, and the presence of small black hairs covering entire body especially prominent extending from anepimeron and underside of T_1+2_. Differs, from *S. antillensis*, which also bears two bold thoracic vittae but does not bear black palpi.

#### Etymology

From the Latin “*ater*”, color black, and the noun “*palpus*”, literally the palm of the hand, in reference to the black palpi present in both sexes of this species.

#### Distribution

Costa Rica, ACG, Prov. Guanacaste, rain forest and rain forest/dry forest ecotone, 290-580m elevation.

#### Ecology

Reared from four species of grass-eating rain forest satyrine Nymphalidae (4 records). One larva per host.

### Spathidexia marioburgosi

Fleming & Wood 2015 sp. n.

urn:lsid:zoobank.org:act:387FAB10-FC62-4C30-824F-D9C872A9EB6A

#### Materials

- **Type status:** Holotype. **Occurrence:** occurrenceDetails: http://janzen.sas.upenn.edu; catalogNumber: DHJPAR0029985; recordedBy: D.H. Janzen & W. Hallwachs; individualID: DHJPAR0029985; individualCount: 1; sex: M; lifeStage: adult; preparations: pinned; otherCatalogNumbers: 08-SRNP-22719; **Taxon:** scientificName: Spathidexia marioburgosi; phylum: Arthropoda; class: Insecta; order: Diptera; family: Tachinidae; genus: Spathidexia; specificEpithet: marioburgosi; scientificNameAuthorship: Fleming & Wood, 2015; **Location:** continent: Central America; country: Costa Rica; countryCode: CR; stateProvince: Guanacaste; county: Area de Conservacion Guanacaste; locality: Sector Del Oro; verbatimLocality: Bosque Aguirre; verbatimElevation: 620; verbatimLatitude: 11.001; verbatimLongitude: -85.438; verbatimCoordinateSystem: Decimal; decimalLatitude: 11.001; decimalLongitude: -85.438; **Identification:** identifiedBy: AJ Fleming; dateIdentified: 2014; **Event:** samplingProtocol: reared from caterpillar of *Talides sinois* (Hesperiidae); verbatimEventDate: 14-Sep-2008; **Record Level:** language: en; institutionCode: CNC; collectionCode: Insects; basisOfRecord: Pinned Specimen
- **Type status:** Paratype. **Occurrence:** occurrenceDetails: http://janzen.sas.upenn.edu; catalogNumber: DHJPAR0005445; recordedBy: D.H. Janzen & W. Hallwachs; individualID: DHJPAR0005445; individualCount: 1; lifeStage: adult; preparations: pinned; otherCatalogNumbers: 05-SRNP-6295; **Taxon:** scientificName: Spathidexia marioburgosi; phylum: Arthropoda; class: Insecta; order: Diptera; family: Tachinidae; genus: Spathidexia; specificEpithet: marioburgosi; scientificNameAuthorship: Fleming & Wood, 2015; **Location:** continent: Central America; country: Costa Rica; countryCode: CR; stateProvince: Alajuela; county: Area de Conservacion Guanacaste; locality: Sector San Cristobal; verbatimLocality: Rio Areno; verbatimElevation: 460; verbatimLatitude: 10.914; verbatimLongitude: -85.382; verbatimCoordinateSystem: Decimal; decimalLatitude: 10.914; decimalLongitude: -85.382; **Identification:** identifiedBy: AJ Fleming; dateIdentified: 2014; **Event:** samplingProtocol: reared from caterpillar of *Talides sergestus* (Hesperiidae); verbatimEventDate: 07-Nov-2005; **Record Level:** language: en; institutionCode: CNC; collectionCode: Insects; basisOfRecord: Pinned Specimen
- **Type status:** Paratype. **Occurrence:** occurrenceDetails: http://janzen.sas.upenn.edu; catalogNumber: DHJPAR0005453; recordedBy: D.H. Janzen & W. Hallwachs; individualID: DHJPAR0005453; individualCount: 1; lifeStage: adult; preparations: pinned; otherCatalogNumbers: 05-SRNP-6890; **Taxon:** scientificName: Spathidexia marioburgosi; phylum: Arthropoda; class: Insecta; order: Diptera; family: Tachinidae; genus: Spathidexia; specificEpithet: marioburgosi; scientificNameAuthorship: Fleming & Wood, 2015; **Location:** continent: Central America; country: Costa Rica; countryCode: CR; stateProvince: Alajuela; county: Area de Conservacion Guanacaste; locality: Sector San Cristobal; verbatimLocality: Sendero Huerta; verbatimElevation: 527; verbatimLatitude: 10.931; verbatimLongitude: -85.372; verbatimCoordinateSystem: Decimal; decimalLatitude: 10.931; decimalLongitude: -85.372; **Identification:** identifiedBy: AJ Fleming; dateIdentified: 2014; **Event:** samplingProtocol: reared from caterpillar of *Talides sinois* (Hesperiidae); verbatimEventDate: 01-Dec-2005; **Record Level:** language: en; institutionCode: CNC; collectionCode: Insects; basisOfRecord: Pinned Specimen
- **Type status:** Paratype. **Occurrence:** occurrenceDetails: http://janzen.sas.upenn.edu; catalogNumber: DHJPAR0005454; recordedBy: D.H. Janzen & W. Hallwachs; individualID: DHJPAR0005454; individualCount: 1; lifeStage: adult; preparations: pinned; otherCatalogNumbers: 05-SRNP-6517; **Taxon:** scientificName: Spathidexia marioburgosi; phylum: Arthropoda; class: Insecta; order: Diptera; family: Tachinidae; genus: Spathidexia; specificEpithet: marioburgosi; scientificNameAuthorship: Fleming & Wood, 2015; **Location:** continent: Central America; country: Costa Rica; countryCode: CR; stateProvince: Alajuela; county: Area de Conservacion Guanacaste; locality: Sector San Cristobal; verbatimLocality: Rio Blanco Abajo; verbatimElevation: 500; verbatimLatitude: 10.9; verbatimLongitude: -85.373; verbatimCoordinateSystem: Decimal; decimalLatitude: 10.9; decimalLongitude: -85.373; **Identification:** identifiedBy: AJ Fleming; dateIdentified: 2014; **Event:** samplingProtocol: reared from caterpillar of *Talides sergestus* (Hesperiidae); verbatimEventDate: 01-Dec-2005; **Record Level:** language: en; institutionCode: CNC; collectionCode: Insects; basisOfRecord: Pinned Specimen
- **Type status:** Paratype. **Occurrence:** occurrenceDetails: http://janzen.sas.upenn.edu; catalogNumber: DHJPAR0005458; recordedBy: D.H. Janzen & W. Hallwachs; individualID: DHJPAR0005458; individualCount: 1; lifeStage: adult; preparations: pinned; otherCatalogNumbers: 05-SRNP-7188; **Taxon:** scientificName: Spathidexia marioburgosi; phylum: Arthropoda; class: Insecta; order: Diptera; family: Tachinidae; genus: Spathidexia; specificEpithet: marioburgosi; scientificNameAuthorship: Fleming & Wood, 2015; **Location:** continent: Central America; country: Costa Rica; countryCode: CR; stateProvince: Alajuela; county: Area de Conservacion Guanacaste; locality: Sector San Cristobal; verbatimLocality: Sendero Huerta; verbatimElevation: 527; verbatimLatitude: 10.931; verbatimLongitude: -85.372; verbatimCoordinateSystem: Decimal; decimalLatitude: 10.931; decimalLongitude: -85.372; **Identification:** identifiedBy: AJ Fleming; dateIdentified: 2014; **Event:** samplingProtocol: reared from caterpillar of *Talides sergestus* (Hesperiidae); verbatimEventDate: 13-Dec-2005; **Record Level:** language: en; institutionCode: CNC; collectionCode: Insects; basisOfRecord: Pinned Specimen
- **Type status:** Paratype. **Occurrence:** occurrenceDetails: http://janzen.sas.upenn.edu; catalogNumber: DHJPAR0005459; recordedBy: D.H. Janzen & W. Hallwachs; individualID: DHJPAR0005459; individualCount: 1; lifeStage: adult; preparations: pinned; otherCatalogNumbers: 05-SRNP-7049; **Taxon:** scientificName: Spathidexia marioburgosi; phylum: Arthropoda; class: Insecta; order: Diptera; family: Tachinidae; genus: Spathidexia; specificEpithet: marioburgosi; scientificNameAuthorship: Fleming & Wood, 2015; **Location:** continent: Central America; country: Costa Rica; countryCode: CR; stateProvince: Alajuela; county: Area de Conservacion Guanacaste; locality: Sector San Cristobal; verbatimLocality: Vado Rio Cucaracho; verbatimElevation: 640; verbatimLatitude: 10.87; verbatimLongitude: -85.392; verbatimCoordinateSystem: Decimal; decimalLatitude: 10.87; decimalLongitude: -85.392; **Identification:** identifiedBy: AJ Fleming; dateIdentified: 2014; **Event:** samplingProtocol: reared from caterpillar of *Talides sergestus* (Hesperiidae); verbatimEventDate: 09-Dec-2005; **Record Level:** language: en; institutionCode: CNC; collectionCode: Insects; basisOfRecord: Pinned Specimen
- **Type status:** Paratype. **Occurrence:** occurrenceDetails: http://janzen.sas.upenn.edu; catalogNumber: DHJPAR0005463; recordedBy: D.H. Janzen & W. Hallwachs; individualID: DHJPAR0005463; individualCount: 1; lifeStage: adult; preparations: pinned; otherCatalogNumbers: 05-SRNP-42898; **Taxon:** scientificName: Spathidexia marioburgosi; phylum: Arthropoda; class: Insecta; order: Diptera; family: Tachinidae; genus: Spathidexia; specificEpithet: marioburgosi; scientificNameAuthorship: Fleming & Wood, 2015; **Location:** continent: Central America; country: Costa Rica; countryCode: CR; stateProvince: Alajuela; county: Area de Conservacion Guanacaste; locality: Sector Rincon Rain Forest; verbatimLocality: Montanya Figueres; verbatimElevation: 460; verbatimLatitude: 10.884; verbatimLongitude: -85.291; verbatimCoordinateSystem: Decimal; decimalLatitude: 10.884; decimalLongitude: -85.291; **Identification:** identifiedBy: AJ Fleming; dateIdentified: 2014; **Event:** samplingProtocol: reared from caterpillar of *Talides sinois* (Hesperiidae); verbatimEventDate: 09-Nov-2005; **Record Level:** language: en; institutionCode: CNC; collectionCode: Insects; basisOfRecord: Pinned Specimen
- **Type status:** Paratype. **Occurrence:** occurrenceDetails: http://janzen.sas.upenn.edu; catalogNumber: DHJPAR0005467; recordedBy: D.H. Janzen & W. Hallwachs; individualID: DHJPAR0005467; individualCount: 1; lifeStage: adult; preparations: pinned; otherCatalogNumbers: 05-SRNP-43349; **Taxon:** scientificName: Spathidexia marioburgosi; phylum: Arthropoda; class: Insecta; order: Diptera; family: Tachinidae; genus: Spathidexia; specificEpithet: marioburgosi; scientificNameAuthorship: Fleming & Wood, 2015; **Location:** continent: Central America; country: Costa Rica; countryCode: CR; stateProvince: Alajuela; county: Area de Conservacion Guanacaste; locality: Sector Rincon Rain Forest; verbatimLocality: Estacion Caribe; verbatimElevation: 415; verbatimLatitude: 10.902; verbatimLongitude: -85.275; verbatimCoordinateSystem: Decimal; decimalLatitude: 10.902; decimalLongitude: -85.275; **Identification:** identifiedBy: AJ Fleming; dateIdentified: 2014; **Event:** samplingProtocol: reared from caterpillar of *Talides sinois* (Hesperiidae); verbatimEventDate: 03-Dec-2005; **Record Level:** language: en; institutionCode: CNC; collectionCode: Insects; basisOfRecord: Pinned Specimen
- **Type status:** Paratype. **Occurrence:** occurrenceDetails: http://janzen.sas.upenn.edu; catalogNumber: DHJPAR0005470; recordedBy: D.H. Janzen & W. Hallwachs; individualID: DHJPAR0005470; individualCount: 1; lifeStage: adult; preparations: pinned; otherCatalogNumbers: 05-SRNP-43130; **Taxon:** scientificName: Spathidexia marioburgosi; phylum: Arthropoda; class: Insecta; order: Diptera; family: Tachinidae; genus: Spathidexia; specificEpithet: marioburgosi; scientificNameAuthorship: Fleming & Wood, 2015; **Location:** continent: Central America; country: Costa Rica; countryCode: CR; stateProvince: Alajuela; county: Area de Conservacion Guanacaste; locality: Sector Rincon Rain Forest; verbatimLocality: Sendero Juntas; verbatimElevation: 400; verbatimLatitude: 10.907; verbatimLongitude: -85.288; verbatimCoordinateSystem: Decimal; decimalLatitude: 10.907; decimalLongitude: -85.288; **Identification:** identifiedBy: AJ Fleming; dateIdentified: 2014; **Event:** samplingProtocol: reared from caterpillar of *Talides sinois* (Hesperiidae); verbatimEventDate: 20-Nov-2005; **Record Level:** language: en; institutionCode: CNC; collectionCode: Insects; basisOfRecord: Pinned Specimen
- **Type status:** Paratype. **Occurrence:** occurrenceDetails: http://janzen.sas.upenn.edu; catalogNumber: DHJPAR0005471; recordedBy: D.H. Janzen & W. Hallwachs; individualID: DHJPAR0005471; individualCount: 1; lifeStage: adult; preparations: pinned; otherCatalogNumbers: 05-SRNP-43431; **Taxon:** scientificName: Spathidexia marioburgosi; phylum: Arthropoda; class: Insecta; order: Diptera; family: Tachinidae; genus: Spathidexia; specificEpithet: marioburgosi; scientificNameAuthorship: Fleming & Wood, 2015; **Location:** continent: Central America; country: Costa Rica; countryCode: CR; stateProvince: Alajuela; county: Area de Conservacion Guanacaste; locality: Sector Rincon Rain Forest; verbatimLocality: Sendero Guaca; verbatimElevation: 400; verbatimLatitude: 10.906; verbatimLongitude: -85.283; verbatimCoordinateSystem: Decimal; decimalLatitude: 10.906; decimalLongitude: -85.283; **Identification:** identifiedBy: AJ Fleming; dateIdentified: 2014; **Event:** samplingProtocol: reared from caterpillar of *Talides sinois* (Hesperiidae); verbatimEventDate: 25-Dec-2005; **Record Level:** language: en; institutionCode: CNC; collectionCode: Insects; basisOfRecord: Pinned Specimen
- **Type status:** Paratype. **Occurrence:** occurrenceDetails: http://janzen.sas.upenn.edu; catalogNumber: DHJPAR0005473; recordedBy: D.H. Janzen & W. Hallwachs; individualID: DHJPAR0005473; individualCount: 1; lifeStage: adult; preparations: pinned; otherCatalogNumbers: 05-SRNP-25074; **Taxon:** scientificName: Spathidexia marioburgosi; phylum: Arthropoda; class: Insecta; order: Diptera; family: Tachinidae; genus: Spathidexia; specificEpithet: marioburgosi; scientificNameAuthorship: Fleming & Wood, 2015; **Location:** continent: Central America; country: Costa Rica; countryCode: CR; stateProvince: Guanacaste; county: Area de Conservacion Guanacaste; locality: Sector Del Oro; verbatimLocality: Sendero Puertas; verbatimElevation: 400; verbatimLatitude: 11.011; verbatimLongitude: -85.488; verbatimCoordinateSystem: Decimal; decimalLatitude: 11.011; decimalLongitude: -85.488; **Identification:** identifiedBy: AJ Fleming; dateIdentified: 2014; **Event:** samplingProtocol: reared from caterpillar of *Talides sinois* (Hesperiidae); verbatimEventDate: 08-Dec-2005; **Record Level:** language: en; institutionCode: CNC; collectionCode: Insects; basisOfRecord: Pinned Specimen
- **Type status:** Paratype. **Occurrence:** occurrenceDetails: http://janzen.sas.upenn.edu; catalogNumber: DHJPAR0011559; recordedBy: D.H. Janzen & W. Hallwachs; individualID: DHJPAR0011559; individualCount: 1; lifeStage: adult; preparations: pinned; otherCatalogNumbers: 05-SRNP-8130; **Taxon:** scientificName: Spathidexia marioburgosi; phylum: Arthropoda; class: Insecta; order: Diptera; family: Tachinidae; genus: Spathidexia; specificEpithet: marioburgosi; scientificNameAuthorship: Fleming & Wood, 2015; **Location:** continent: Central America; country: Costa Rica; countryCode: CR; stateProvince: Alajuela; county: Area de Conservacion Guanacaste; locality: Sector San Cristobal; verbatimLocality: Finca San Gabriel; verbatimElevation: 645; verbatimLatitude: 10.878; verbatimLongitude: -85.393; verbatimCoordinateSystem: Decimal; decimalLatitude: 10.878; decimalLongitude: -85.393; **Identification:** identifiedBy: AJ Fleming; dateIdentified: 2014; **Event:** samplingProtocol: reared from caterpillar of *Talides sinois* (Hesperiidae); verbatimEventDate: 06-Feb-2006; **Record Level:** language: en; institutionCode: CNC; collectionCode: Insects; basisOfRecord: Pinned Specimen
- **Type status:** Paratype. **Occurrence:** occurrenceDetails: http://janzen.sas.upenn.edu; catalogNumber: DHJPAR0011572; recordedBy: D.H. Janzen & W. Hallwachs; individualID: DHJPAR0011572; individualCount: 1; lifeStage: adult; preparations: pinned; otherCatalogNumbers: 05-SRNP-43349; **Taxon:** scientificName: Spathidexia marioburgosi; phylum: Arthropoda; class: Insecta; order: Diptera; family: Tachinidae; genus: Spathidexia; specificEpithet: marioburgosi; scientificNameAuthorship: Fleming & Wood, 2015; **Location:** continent: Central America; country: Costa Rica; countryCode: CR; stateProvince: Alajuela; county: Area de Conservacion Guanacaste; locality: Sector Rincon Rain Forest; verbatimLocality: Estacion Caribe; verbatimElevation: 415; verbatimLatitude: 10.902; verbatimLongitude: -85.275; verbatimCoordinateSystem: Decimal; decimalLatitude: 10.902; decimalLongitude: -85.275; **Identification:** identifiedBy: AJ Fleming; dateIdentified: 2014; **Event:** samplingProtocol: reared from caterpillar of *Talides sergestus* (Hesperiidae); verbatimEventDate: 03-Dec-2005; **Record Level:** language: en; institutionCode: CNC; collectionCode: Insects; basisOfRecord: Pinned Specimen
- **Type status:** Paratype. **Occurrence:** occurrenceDetails: http://janzen.sas.upenn.edu; catalogNumber: DHJPAR0015271; recordedBy: D.H. Janzen & W. Hallwachs; individualID: DHJPAR0015271; individualCount: 1; lifeStage: adult; preparations: pinned; otherCatalogNumbers: 03-SRNP-28308; **Taxon:** scientificName: Spathidexia marioburgosi; phylum: Arthropoda; class: Insecta; order: Diptera; family: Tachinidae; genus: Spathidexia; specificEpithet: marioburgosi; scientificNameAuthorship: Fleming & Wood, 2015; **Location:** continent: Central America; country: Costa Rica; countryCode: CR; stateProvince: Guanacaste; county: Area de Conservacion Guanacaste; locality: Sector Del Oro; verbatimLocality: Uncaria; verbatimElevation: 370; verbatimLatitude: 11.018; verbatimLongitude: -85.474; verbatimCoordinateSystem: Decimal; decimalLatitude: 11.018; decimalLongitude: -85.474; **Identification:** identifiedBy: AJ Fleming; dateIdentified: 2014; **Event:** samplingProtocol: reared from caterpillar of *Talides sergestus* (Hesperiidae); verbatimEventDate: 06-Oct-2003; **Record Level:** language: en; institutionCode: CNC; collectionCode: Insects; basisOfRecord: Pinned Specimen
- **Type status:** Paratype. **Occurrence:** occurrenceDetails: http://janzen.sas.upenn.edu; catalogNumber: DHJPAR0016073; recordedBy: D.H. Janzen & W. Hallwachs; individualID: DHJPAR0016073; individualCount: 1; lifeStage: adult; preparations: pinned; otherCatalogNumbers: 06-SRNP-4769; **Taxon:** scientificName: Spathidexia marioburgosi; phylum: Arthropoda; class: Insecta; order: Diptera; family: Tachinidae; genus: Spathidexia; specificEpithet: marioburgosi; scientificNameAuthorship: Fleming & Wood, 2015; **Location:** continent: Central America; country: Costa Rica; countryCode: CR; stateProvince: Alajuela; county: Area de Conservacion Guanacaste; locality: Sector San Cristobal; verbatimLocality: Rio Blanco Abajo; verbatimElevation: 500; verbatimLatitude: 10.9; verbatimLongitude: -85.373; verbatimCoordinateSystem: Decimal; decimalLatitude: 10.9; decimalLongitude: -85.373; **Identification:** identifiedBy: AJ Fleming; dateIdentified: 2014; **Event:** samplingProtocol: reared from caterpillar of *Talides sinois* (Hesperiidae); verbatimEventDate: 16-Jul-2006; **Record Level:** language: en; institutionCode: CNC; collectionCode: Insects; basisOfRecord: Pinned Specimen
- **Type status:** Paratype. **Occurrence:** occurrenceDetails: http://janzen.sas.upenn.edu; catalogNumber: DHJPAR0016107; recordedBy: D.H. Janzen & W. Hallwachs; individualID: DHJPAR0016107; individualCount: 1; lifeStage: adult; preparations: pinned; otherCatalogNumbers: 06-SRNP-58077; **Taxon:** scientificName: Spathidexia marioburgosi; phylum: Arthropoda; class: Insecta; order: Diptera; family: Tachinidae; genus: Spathidexia; specificEpithet: marioburgosi; scientificNameAuthorship: Fleming & Wood, 2015; **Location:** continent: Central America; country: Costa Rica; countryCode: CR; stateProvince: Guanacaste; county: Area de Conservacion Guanacaste; locality: Sector Mundo Nuevo; verbatimLocality: Quebrada Tibio Perla; verbatimElevation: 330; verbatimLatitude: 10.763; verbatimLongitude: -85.43; verbatimCoordinateSystem: Decimal; decimalLatitude: 10.763; decimalLongitude: -85.43; **Identification:** identifiedBy: AJ Fleming; dateIdentified: 2014; **Event:** samplingProtocol: reared from caterpillar of *Talides sinois* (Hesperiidae); verbatimEventDate: 21-Sep-2006; **Record Level:** language: en; institutionCode: CNC; collectionCode: Insects; basisOfRecord: Pinned Specimen
- **Type status:** Paratype. **Occurrence:** occurrenceDetails: http://janzen.sas.upenn.edu; catalogNumber: DHJPAR0016178; recordedBy: D.H. Janzen & W. Hallwachs; individualID: DHJPAR0016178; individualCount: 1; lifeStage: adult; preparations: pinned; otherCatalogNumbers: 06-SRNP-7631; **Taxon:** scientificName: Spathidexia marioburgosi; phylum: Arthropoda; class: Insecta; order: Diptera; family: Tachinidae; genus: Spathidexia; specificEpithet: marioburgosi; scientificNameAuthorship: Fleming & Wood, 2015; **Location:** continent: Central America; country: Costa Rica; countryCode: CR; stateProvince: Alajuela; county: Area de Conservacion Guanacaste; locality: Sector San Cristobal; verbatimLocality: Finca San Gabriel; verbatimElevation: 645; verbatimLatitude: 10.878; verbatimLongitude: -85.393; verbatimCoordinateSystem: Decimal; decimalLatitude: 10.878; decimalLongitude: -85.393; **Identification:** identifiedBy: AJ Fleming; dateIdentified: 2014; **Event:** samplingProtocol: reared from caterpillar of *Talides sinois* (Hesperiidae); verbatimEventDate: 12-Oct-2006; **Record Level:** language: en; institutionCode: CNC; collectionCode: Insects; basisOfRecord: Pinned Specimen
- **Type status:** Paratype. **Occurrence:** occurrenceDetails: http://janzen.sas.upenn.edu; catalogNumber: DHJPAR0016180; recordedBy: D.H. Janzen & W. Hallwachs; individualID: DHJPAR0016180; individualCount: 1; lifeStage: adult; preparations: pinned; otherCatalogNumbers: 06-SRNP-58374; **Taxon:** scientificName: Spathidexia marioburgosi; phylum: Arthropoda; class: Insecta; order: Diptera; family: Tachinidae; genus: Spathidexia; specificEpithet: marioburgosi; scientificNameAuthorship: Fleming & Wood, 2015; **Location:** continent: Central America; country: Costa Rica; countryCode: CR; stateProvince: Guanacaste; county: Area de Conservacion Guanacaste; locality: Sector Mundo Nuevo; verbatimLocality: Vado Huacas; verbatimElevation: 490; verbatimLatitude: 10.755; verbatimLongitude: -85.391; verbatimCoordinateSystem: Decimal; decimalLatitude: 10.755; decimalLongitude: -85.391; **Identification:** identifiedBy: AJ Fleming; dateIdentified: 2014; **Event:** samplingProtocol: reared from caterpillar of *Talides sergestus* (Hesperiidae); verbatimEventDate: 11-Oct-2006; **Record Level:** language: en; institutionCode: CNC; collectionCode: Insects; basisOfRecord: Pinned Specimen
- **Type status:** Paratype. **Occurrence:** occurrenceDetails: http://janzen.sas.upenn.edu; catalogNumber: DHJPAR0016236; recordedBy: D.H. Janzen & W. Hallwachs; individualID: DHJPAR0016236; individualCount: 1; lifeStage: adult; preparations: pinned; otherCatalogNumbers: 06-SRNP-58370; **Taxon:** scientificName: Spathidexia marioburgosi; phylum: Arthropoda; class: Insecta; order: Diptera; family: Tachinidae; genus: Spathidexia; specificEpithet: marioburgosi; scientificNameAuthorship: Fleming & Wood, 2015; **Location:** continent: Central America; country: Costa Rica; countryCode: CR; stateProvince: Guanacaste; county: Area de Conservacion Guanacaste; locality: Sector Mundo Nuevo; verbatimLocality: Vado Huacas; verbatimElevation: 490; verbatimLatitude: 10.755; verbatimLongitude: -85.391; verbatimCoordinateSystem: Decimal; decimalLatitude: 10.755; decimalLongitude: -85.391; **Identification:** identifiedBy: AJ Fleming; dateIdentified: 2014; **Event:** samplingProtocol: reared from caterpillar of *Talides sinois* (Hesperiidae); verbatimEventDate: 11-Oct-2006; **Record Level:** language: en; institutionCode: CNC; collectionCode: Insects; basisOfRecord: Pinned Specimen
- **Type status:** Paratype. **Occurrence:** occurrenceDetails: http://janzen.sas.upenn.edu; catalogNumber: DHJPAR0016242; recordedBy: D.H. Janzen & W. Hallwachs; individualID: DHJPAR0016242; individualCount: 1; lifeStage: adult; preparations: pinned; otherCatalogNumbers: 06-SRNP-6683; **Taxon:** scientificName: Spathidexia marioburgosi; phylum: Arthropoda; class: Insecta; order: Diptera; family: Tachinidae; genus: Spathidexia; specificEpithet: marioburgosi; scientificNameAuthorship: Fleming & Wood, 2015; **Location:** continent: Central America; country: Costa Rica; countryCode: CR; stateProvince: Alajuela; county: Area de Conservacion Guanacaste; locality: Sector San Cristobal; verbatimLocality: Finca San Gabriel; verbatimElevation: 645; verbatimLatitude: 10.878; verbatimLongitude: -85.393; verbatimCoordinateSystem: Decimal; decimalLatitude: 10.878; decimalLongitude: -85.393; **Identification:** identifiedBy: AJ Fleming; dateIdentified: 2014; **Event:** samplingProtocol: reared from caterpillar of *Talides sergestus* (Hesperiidae); verbatimEventDate: 01-Sep-2006; **Record Level:** language: en; institutionCode: CNC; collectionCode: Insects; basisOfRecord: Pinned Specimen
- **Type status:** Paratype. **Occurrence:** occurrenceDetails: http://janzen.sas.upenn.edu; catalogNumber: DHJPAR0016313; recordedBy: D.H. Janzen & W. Hallwachs; individualID: DHJPAR0016313; individualCount: 1; lifeStage: adult; preparations: pinned; otherCatalogNumbers: 06-SRNP-6796; **Taxon:** scientificName: Spathidexia marioburgosi; phylum: Arthropoda; class: Insecta; order: Diptera; family: Tachinidae; genus: Spathidexia; specificEpithet: marioburgosi; scientificNameAuthorship: Fleming & Wood, 2015; **Location:** continent: Central America; country: Costa Rica; countryCode: CR; stateProvince: Alajuela; county: Area de Conservacion Guanacaste; locality: Sector San Cristobal; verbatimLocality: Puente Palma; verbatimElevation: 460; verbatimLatitude: 10.916; verbatimLongitude: -85.379; verbatimCoordinateSystem: Decimal; decimalLatitude: 10.916; decimalLongitude: -85.379; **Identification:** identifiedBy: AJ Fleming; dateIdentified: 2014; **Event:** samplingProtocol: reared from caterpillar of *Talides sinois* (Hesperiidae); verbatimEventDate: 08-Sep-2006; **Record Level:** language: en; institutionCode: CNC; collectionCode: Insects; basisOfRecord: Pinned Specimen
- **Type status:** Paratype. **Occurrence:** occurrenceDetails: http://janzen.sas.upenn.edu; catalogNumber: DHJPAR0016330; recordedBy: D.H. Janzen & W. Hallwachs; individualID: DHJPAR0016330; individualCount: 1; lifeStage: adult; preparations: pinned; otherCatalogNumbers: 06-SRNP-46866; **Taxon:** scientificName: Spathidexia marioburgosi; phylum: Arthropoda; class: Insecta; order: Diptera; family: Tachinidae; genus: Spathidexia; specificEpithet: marioburgosi; scientificNameAuthorship: Fleming & Wood, 2015; **Location:** continent: Central America; country: Costa Rica; countryCode: CR; stateProvince: Guanacaste; county: Area de Conservacion Guanacaste; locality: Sector Cacao; verbatimLocality: Gongora Bananal; verbatimElevation: 600; verbatimLatitude: 10.889; verbatimLongitude: -85.476; verbatimCoordinateSystem: Decimal; decimalLatitude: 10.889; decimalLongitude: -85.476; **Identification:** identifiedBy: AJ Fleming; dateIdentified: 2014; **Event:** samplingProtocol: reared from caterpillar of *Talides sinois* (Hesperiidae); verbatimEventDate: 05-Sep-2006; **Record Level:** language: en; institutionCode: CNC; collectionCode: Insects; basisOfRecord: Pinned Specimen
- **Type status:** Paratype. **Occurrence:** occurrenceDetails: http://janzen.sas.upenn.edu; catalogNumber: DHJPAR0016488; recordedBy: D.H. Janzen & W. Hallwachs; individualID: DHJPAR0016488; individualCount: 1; lifeStage: adult; preparations: pinned; otherCatalogNumbers: 06-SRNP-47185; **Taxon:** scientificName: Spathidexia marioburgosi; phylum: Arthropoda; class: Insecta; order: Diptera; family: Tachinidae; genus: Spathidexia; specificEpithet: marioburgosi; scientificNameAuthorship: Fleming & Wood, 2015; **Location:** continent: Central America; country: Costa Rica; countryCode: CR; stateProvince: Guanacaste; county: Area de Conservacion Guanacaste; locality: Sector Cacao; verbatimLocality: Puente Gongora; verbatimElevation: 540; verbatimLatitude: 10.885; verbatimLongitude: -85.472; verbatimCoordinateSystem: Decimal; decimalLatitude: 10.885; decimalLongitude: -85.472; **Identification:** identifiedBy: AJ Fleming; dateIdentified: 2014; **Event:** samplingProtocol: reared from caterpillar of *Talides sinois* (Hesperiidae); verbatimEventDate: 24-Sep-2006; **Record Level:** language: en; institutionCode: CNC; collectionCode: Insects; basisOfRecord: Pinned Specimen
- **Type status:** Paratype. **Occurrence:** occurrenceDetails: http://janzen.sas.upenn.edu; catalogNumber: DHJPAR0016537; recordedBy: D.H. Janzen & W. Hallwachs; individualID: DHJPAR0016537; individualCount: 1; lifeStage: adult; preparations: pinned; otherCatalogNumbers: 06-SRNP-44678; **Taxon:** scientificName: Spathidexia marioburgosi; phylum: Arthropoda; class: Insecta; order: Diptera; family: Tachinidae; genus: Spathidexia; specificEpithet: marioburgosi; scientificNameAuthorship: Fleming & Wood, 2015; **Location:** continent: Central America; country: Costa Rica; countryCode: CR; stateProvince: Alajuela; county: Area de Conservacion Guanacaste; locality: Sector Rincon Rain Forest; verbatimLocality: Sendero Llano; verbatimElevation: 400; verbatimLatitude: 10.903; verbatimLongitude: -85.29; verbatimCoordinateSystem: Decimal; decimalLatitude: 10.903; decimalLongitude: -85.29; **Identification:** identifiedBy: AJ Fleming; dateIdentified: 2014; **Event:** samplingProtocol: reared from caterpillar of *Talides sergestus* (Hesperiidae); verbatimEventDate: 06-Jan-2007; **Record Level:** language: en; institutionCode: CNC; collectionCode: Insects; basisOfRecord: Pinned Specimen
- **Type status:** Paratype. **Occurrence:** occurrenceDetails: http://janzen.sas.upenn.edu; catalogNumber: DHJPAR0016600; recordedBy: D.H. Janzen & W. Hallwachs; individualID: DHJPAR0016600; individualCount: 1; lifeStage: adult; preparations: pinned; otherCatalogNumbers: 06-SRNP-48089; **Taxon:** scientificName: Spathidexia marioburgosi; phylum: Arthropoda; class: Insecta; order: Diptera; family: Tachinidae; genus: Spathidexia; specificEpithet: marioburgosi; scientificNameAuthorship: Fleming & Wood, 2015; **Location:** continent: Central America; country: Costa Rica; countryCode: CR; stateProvince: Guanacaste; county: Area de Conservacion Guanacaste; locality: Sector Cacao; verbatimLocality: Puente Gongora; verbatimElevation: 540; verbatimLatitude: 10.885; verbatimLongitude: -85.472; verbatimCoordinateSystem: Decimal; decimalLatitude: 10.885; decimalLongitude: -85.472; **Identification:** identifiedBy: AJ Fleming; dateIdentified: 2014; **Event:** samplingProtocol: reared from caterpillar of *Talides sinois* (Hesperiidae); verbatimEventDate: 12-Dec-2006; **Record Level:** language: en; institutionCode: CNC; collectionCode: Insects; basisOfRecord: Pinned Specimen
- **Type status:** Paratype. **Occurrence:** occurrenceDetails: http://janzen.sas.upenn.edu; catalogNumber: DHJPAR0019550; recordedBy: D.H. Janzen & W. Hallwachs; individualID: DHJPAR0019550; individualCount: 1; lifeStage: adult; preparations: pinned; otherCatalogNumbers: 06-SRNP-8343; **Taxon:** scientificName: Spathidexia marioburgosi; phylum: Arthropoda; class: Insecta; order: Diptera; family: Tachinidae; genus: Spathidexia; specificEpithet: marioburgosi; scientificNameAuthorship: Fleming & Wood, 2015; **Location:** continent: Central America; country: Costa Rica; countryCode: CR; stateProvince: Alajuela; county: Area de Conservacion Guanacaste; locality: Sector San Cristobal; verbatimLocality: Puente Palma; verbatimElevation: 460; verbatimLatitude: 10.916; verbatimLongitude: -85.379; verbatimCoordinateSystem: Decimal; decimalLatitude: 10.916; decimalLongitude: -85.379; **Identification:** identifiedBy: AJ Fleming; dateIdentified: 2014; **Event:** samplingProtocol: reared from caterpillar of *Talides sergestus* (Hesperiidae); verbatimEventDate: 31-Oct-2006; **Record Level:** language: en; institutionCode: CNC; collectionCode: Insects; basisOfRecord: Pinned Specimen
- **Type status:** Paratype. **Occurrence:** occurrenceDetails: http://janzen.sas.upenn.edu; catalogNumber: DHJPAR0019612; recordedBy: D.H. Janzen & W. Hallwachs; individualID: DHJPAR0019612; individualCount: 1; lifeStage: adult; preparations: pinned; otherCatalogNumbers: 07-SRNP-65140; **Taxon:** scientificName: Spathidexia marioburgosi; phylum: Arthropoda; class: Insecta; order: Diptera; family: Tachinidae; genus: Spathidexia; specificEpithet: marioburgosi; scientificNameAuthorship: Fleming & Wood, 2015; **Location:** continent: Central America; country: Costa Rica; countryCode: CR; stateProvince: Alajuela; county: Area de Conservacion Guanacaste; locality: El Ensayo; verbatimLocality: Metereologico; verbatimElevation: 330; verbatimLatitude: 10.76261; verbatimLongitude: -85.42979; verbatimCoordinateSystem: Decimal; decimalLatitude: 10.76261; decimalLongitude: -85.42979; **Identification:** identifiedBy: AJ Fleming; dateIdentified: 2014; **Event:** samplingProtocol: reared from caterpillar of *Talides sergestus* (Hesperiidae); verbatimEventDate: 18-May-2007; **Record Level:** language: en; institutionCode: CNC; collectionCode: Insects; basisOfRecord: Pinned Specimen
- **Type status:** Paratype. **Occurrence:** occurrenceDetails: http://janzen.sas.upenn.edu; catalogNumber: DHJPAR0019613; recordedBy: D.H. Janzen & W. Hallwachs; individualID: DHJPAR0019613; individualCount: 1; lifeStage: adult; preparations: pinned; otherCatalogNumbers: 07-SRNP-56562; **Taxon:** scientificName: Spathidexia marioburgosi; phylum: Arthropoda; class: Insecta; order: Diptera; family: Tachinidae; genus: Spathidexia; specificEpithet: marioburgosi; scientificNameAuthorship: Fleming & Wood, 2015; **Location:** continent: Central America; country: Costa Rica; countryCode: CR; stateProvince: Guanacaste; county: Area de Conservacion Guanacaste; locality: Sector Mundo Nuevo; verbatimLocality: Quebrada Tibio Perla; verbatimElevation: 330; verbatimLatitude: 10.763; verbatimLongitude: -85.43; verbatimCoordinateSystem: Decimal; decimalLatitude: 10.763; decimalLongitude: -85.43; **Identification:** identifiedBy: AJ Fleming; dateIdentified: 2014; **Event:** samplingProtocol: reared from caterpillar of *Talides sinois* (Hesperiidae); verbatimEventDate: 10-Jun-2007; **Record Level:** language: en; institutionCode: CNC; collectionCode: Insects; basisOfRecord: Pinned Specimen
- **Type status:** Paratype. **Occurrence:** occurrenceDetails: http://janzen.sas.upenn.edu; catalogNumber: DHJPAR0019709; recordedBy: D.H. Janzen & W. Hallwachs; individualID: DHJPAR0019709; individualCount: 1; lifeStage: adult; preparations: pinned; otherCatalogNumbers: 07-SRNP-56533; **Taxon:** scientificName: Spathidexia marioburgosi; phylum: Arthropoda; class: Insecta; order: Diptera; family: Tachinidae; genus: Spathidexia; specificEpithet: marioburgosi; scientificNameAuthorship: Fleming & Wood, 2015; **Location:** continent: Central America; country: Costa Rica; countryCode: CR; stateProvince: Guanacaste; county: Area de Conservacion Guanacaste; locality: Sector Mundo Nuevo; verbatimLocality: Quebrada Tibio Perla; verbatimElevation: 330; verbatimLatitude: 10.763; verbatimLongitude: -85.43; verbatimCoordinateSystem: Decimal; decimalLatitude: 10.763; decimalLongitude: -85.43; **Identification:** identifiedBy: AJ Fleming; dateIdentified: 2014; **Event:** samplingProtocol: reared from caterpillar of *Talides sinois* (Hesperiidae); verbatimEventDate: 10-Jun-2007; **Record Level:** language: en; institutionCode: CNC; collectionCode: Insects; basisOfRecord: Pinned Specimen
- **Type status:** Paratype. **Occurrence:** occurrenceDetails: http://janzen.sas.upenn.edu; catalogNumber: DHJPAR0019710; recordedBy: D.H. Janzen & W. Hallwachs; individualID: DHJPAR0019710; individualCount: 1; lifeStage: adult; preparations: pinned; otherCatalogNumbers: 07-SRNP-1851; **Taxon:** scientificName: Spathidexia marioburgosi; phylum: Arthropoda; class: Insecta; order: Diptera; family: Tachinidae; genus: Spathidexia; specificEpithet: marioburgosi; scientificNameAuthorship: Fleming & Wood, 2015; **Location:** continent: Central America; country: Costa Rica; countryCode: CR; stateProvince: Alajuela; county: Area de Conservacion Guanacaste; locality: Sector San Cristobal; verbatimLocality: Finca San Gabriel; verbatimElevation: 645; verbatimLatitude: 10.878; verbatimLongitude: -85.393; verbatimCoordinateSystem: Decimal; decimalLatitude: 10.878; decimalLongitude: -85.393; **Identification:** identifiedBy: AJ Fleming; dateIdentified: 2014; **Event:** samplingProtocol: reared from caterpillar of *Talides sinois* (Hesperiidae); verbatimEventDate: 17-May-2007; **Record Level:** language: en; institutionCode: CNC; collectionCode: Insects; basisOfRecord: Pinned Specimen
- **Type status:** Paratype. **Occurrence:** occurrenceDetails: http://janzen.sas.upenn.edu; catalogNumber: DHJPAR0019711; recordedBy: D.H. Janzen & W. Hallwachs; individualID: DHJPAR0019711; individualCount: 1; lifeStage: adult; preparations: pinned; otherCatalogNumbers: 07-SRNP-1852; **Taxon:** scientificName: Spathidexia marioburgosi; phylum: Arthropoda; class: Insecta; order: Diptera; family: Tachinidae; genus: Spathidexia; specificEpithet: marioburgosi; scientificNameAuthorship: Fleming & Wood, 2015; **Location:** continent: Central America; country: Costa Rica; countryCode: CR; stateProvince: Alajuela; county: Area de Conservacion Guanacaste; locality: Sector San Cristobal; verbatimLocality: Finca San Gabriel; verbatimElevation: 645; verbatimLatitude: 10.878; verbatimLongitude: -85.393; verbatimCoordinateSystem: Decimal; decimalLatitude: 10.878; decimalLongitude: -85.393; **Identification:** identifiedBy: AJ Fleming; dateIdentified: 2014; **Event:** samplingProtocol: reared from caterpillar of *Talides sinois* (Hesperiidae); verbatimEventDate: 18-May-2007; **Record Level:** language: en; institutionCode: CNC; collectionCode: Insects; basisOfRecord: Pinned Specimen
- **Type status:** Paratype. **Occurrence:** occurrenceDetails: http://janzen.sas.upenn.edu; catalogNumber: DHJPAR0019801; recordedBy: D.H. Janzen & W. Hallwachs; individualID: DHJPAR0019801; individualCount: 1; lifeStage: adult; preparations: pinned; otherCatalogNumbers: 07-SRNP-1852; **Taxon:** scientificName: Spathidexia marioburgosi; phylum: Arthropoda; class: Insecta; order: Diptera; family: Tachinidae; genus: Spathidexia; specificEpithet: marioburgosi; scientificNameAuthorship: Fleming & Wood, 2015; **Location:** continent: Central America; country: Costa Rica; countryCode: CR; stateProvince: Alajuela; county: Area de Conservacion Guanacaste; locality: Sector San Cristobal; verbatimLocality: Finca San Gabriel; verbatimElevation: 645; verbatimLatitude: 10.878; verbatimLongitude: -85.393; verbatimCoordinateSystem: Decimal; decimalLatitude: 10.878; decimalLongitude: -85.393; **Identification:** identifiedBy: AJ Fleming; dateIdentified: 2014; **Event:** samplingProtocol: reared from caterpillar of *Talides sinois* (Hesperiidae); verbatimEventDate: 18-May-2007; **Record Level:** language: en; institutionCode: CNC; collectionCode: Insects; basisOfRecord: Pinned Specimen
- **Type status:** Paratype. **Occurrence:** occurrenceDetails: http://janzen.sas.upenn.edu; catalogNumber: DHJPAR0019954; recordedBy: D.H. Janzen & W. Hallwachs; individualID: DHJPAR0019954; individualCount: 1; lifeStage: adult; preparations: pinned; otherCatalogNumbers: 07-SRNP-56555; **Taxon:** scientificName: Spathidexia marioburgosi; phylum: Arthropoda; class: Insecta; order: Diptera; family: Tachinidae; genus: Spathidexia; specificEpithet: marioburgosi; scientificNameAuthorship: Fleming & Wood, 2015; **Location:** continent: Central America; country: Costa Rica; countryCode: CR; stateProvince: Guanacaste; county: Area de Conservacion Guanacaste; locality: Sector Mundo Nuevo; verbatimLocality: Quebrada Tibio Perla; verbatimElevation: 330; verbatimLatitude: 10.763; verbatimLongitude: -85.43; verbatimCoordinateSystem: Decimal; decimalLatitude: 10.763; decimalLongitude: -85.43; **Identification:** identifiedBy: AJ Fleming; dateIdentified: 2014; **Event:** samplingProtocol: reared from caterpillar of *Talides sergestus* (Hesperiidae); verbatimEventDate: 05-Jun-2007; **Record Level:** language: en; institutionCode: CNC; collectionCode: Insects; basisOfRecord: Pinned Specimen
- **Type status:** Paratype. **Occurrence:** occurrenceDetails: http://janzen.sas.upenn.edu; catalogNumber: DHJPAR0020920; recordedBy: D.H. Janzen & W. Hallwachs; individualID: DHJPAR0020920; individualCount: 1; lifeStage: adult; preparations: pinned; otherCatalogNumbers: 07-SRNP-58809; **Taxon:** scientificName: Spathidexia marioburgosi; phylum: Arthropoda; class: Insecta; order: Diptera; family: Tachinidae; genus: Spathidexia; specificEpithet: marioburgosi; scientificNameAuthorship: Fleming & Wood, 2015; **Location:** continent: Central America; country: Costa Rica; countryCode: CR; stateProvince: Guanacaste; county: Area de Conservacion Guanacaste; locality: Sector Mundo Nuevo; verbatimLocality: Vado Miramonte; verbatimElevation: 305; verbatimLatitude: 10.772; verbatimLongitude: -85.434; verbatimCoordinateSystem: Decimal; decimalLatitude: 10.772; decimalLongitude: -85.434; **Identification:** identifiedBy: AJ Fleming; dateIdentified: 2014; **Event:** samplingProtocol: reared from caterpillar of *Talides sinois* (Hesperiidae); verbatimEventDate: 17-Aug-2007; **Record Level:** language: en; institutionCode: CNC; collectionCode: Insects; basisOfRecord: Pinned Specimen
- **Type status:** Paratype. **Occurrence:** occurrenceDetails: http://janzen.sas.upenn.edu; catalogNumber: DHJPAR0020936; recordedBy: D.H. Janzen & W. Hallwachs; individualID: DHJPAR0020936; individualCount: 1; lifeStage: adult; preparations: pinned; otherCatalogNumbers: 07-SRNP-58951; **Taxon:** scientificName: Spathidexia marioburgosi; phylum: Arthropoda; class: Insecta; order: Diptera; family: Tachinidae; genus: Spathidexia; specificEpithet: marioburgosi; scientificNameAuthorship: Fleming & Wood, 2015; **Location:** continent: Central America; country: Costa Rica; countryCode: CR; stateProvince: Guanacaste; county: Area de Conservacion Guanacaste; locality: Sector Mundo Nuevo; verbatimLocality: Quebrada Tibio Perla; verbatimElevation: 330; verbatimLatitude: 10.763; verbatimLongitude: -85.43; verbatimCoordinateSystem: Decimal; decimalLatitude: 10.763; decimalLongitude: -85.43; **Identification:** identifiedBy: AJ Fleming; dateIdentified: 2014; **Event:** samplingProtocol: reared from caterpillar of *Talides sinois* (Hesperiidae); verbatimEventDate: 31-Aug-2007; **Record Level:** language: en; institutionCode: CNC; collectionCode: Insects; basisOfRecord: Pinned Specimen
- **Type status:** Paratype. **Occurrence:** occurrenceDetails: http://janzen.sas.upenn.edu; catalogNumber: DHJPAR0020937; recordedBy: D.H. Janzen & W. Hallwachs; individualID: DHJPAR0020937; individualCount: 1; lifeStage: adult; preparations: pinned; otherCatalogNumbers: 07-SRNP-58016; **Taxon:** scientificName: Spathidexia marioburgosi; phylum: Arthropoda; class: Insecta; order: Diptera; family: Tachinidae; genus: Spathidexia; specificEpithet: marioburgosi; scientificNameAuthorship: Fleming & Wood, 2015; **Location:** continent: Central America; country: Costa Rica; countryCode: CR; stateProvince: Guanacaste; county: Area de Conservacion Guanacaste; locality: Sector Mundo Nuevo; verbatimLocality: Quebrada Tibio Perla; verbatimElevation: 330; verbatimLatitude: 10.763; verbatimLongitude: -85.43; verbatimCoordinateSystem: Decimal; decimalLatitude: 10.763; decimalLongitude: -85.43; **Identification:** identifiedBy: AJ Fleming; dateIdentified: 2014; **Event:** samplingProtocol: reared from caterpillar of *Talides sinois* (Hesperiidae); verbatimEventDate: 25-Jul-2007; **Record Level:** language: en; institutionCode: CNC; collectionCode: Insects; basisOfRecord: Pinned Specimen
- **Type status:** Paratype. **Occurrence:** occurrenceDetails: http://janzen.sas.upenn.edu; catalogNumber: DHJPAR0020938; recordedBy: D.H. Janzen & W. Hallwachs; individualID: DHJPAR0020938; individualCount: 1; lifeStage: adult; preparations: pinned; otherCatalogNumbers: 07-SRNP-58356; **Taxon:** scientificName: Spathidexia marioburgosi; phylum: Arthropoda; class: Insecta; order: Diptera; family: Tachinidae; genus: Spathidexia; specificEpithet: marioburgosi; scientificNameAuthorship: Fleming & Wood, 2015; **Location:** continent: Central America; country: Costa Rica; countryCode: CR; stateProvince: Guanacaste; county: Area de Conservacion Guanacaste; locality: Sector Mundo Nuevo; verbatimLocality: Vado Miramonte; verbatimElevation: 305; verbatimLatitude: 10.772; verbatimLongitude: -85.434; verbatimCoordinateSystem: Decimal; decimalLatitude: 10.772; decimalLongitude: -85.434; **Identification:** identifiedBy: AJ Fleming; dateIdentified: 2014; **Event:** samplingProtocol: reared from caterpillar of *Talides* Burns02 (Hesperiidae); verbatimEventDate: 09-Aug-2007; **Record Level:** language: en; institutionCode: CNC; collectionCode: Insects; basisOfRecord: Pinned Specimen
- **Type status:** Paratype. **Occurrence:** occurrenceDetails: http://janzen.sas.upenn.edu; catalogNumber: DHJPAR0021856; recordedBy: D.H. Janzen & W. Hallwachs; individualID: DHJPAR0021856; individualCount: 1; lifeStage: adult; preparations: pinned; otherCatalogNumbers: 07-SRNP-58377; **Taxon:** scientificName: Spathidexia marioburgosi; phylum: Arthropoda; class: Insecta; order: Diptera; family: Tachinidae; genus: Spathidexia; specificEpithet: marioburgosi; scientificNameAuthorship: Fleming & Wood, 2015; **Location:** continent: Central America; country: Costa Rica; countryCode: CR; stateProvince: Guanacaste; county: Area de Conservacion Guanacaste; locality: Sector Mundo Nuevo; verbatimLocality: Vado Miramonte; verbatimElevation: 305; verbatimLatitude: 10.772; verbatimLongitude: -85.434; verbatimCoordinateSystem: Decimal; decimalLatitude: 10.772; decimalLongitude: -85.434; **Identification:** identifiedBy: AJ Fleming; dateIdentified: 2014; **Event:** samplingProtocol: reared from caterpillar of *Talides sergestus* (Hesperiidae); verbatimEventDate: 07-Aug-2007; **Record Level:** language: en; institutionCode: CNC; collectionCode: Insects; basisOfRecord: Pinned Specimen
- **Type status:** Paratype. **Occurrence:** occurrenceDetails: http://janzen.sas.upenn.edu; catalogNumber: DHJPAR0022284; recordedBy: D.H. Janzen & W. Hallwachs; individualID: DHJPAR0022284; individualCount: 1; lifeStage: adult; preparations: pinned; otherCatalogNumbers: 07-SRNP-59237; **Taxon:** scientificName: Spathidexia marioburgosi; phylum: Arthropoda; class: Insecta; order: Diptera; family: Tachinidae; genus: Spathidexia; specificEpithet: marioburgosi; scientificNameAuthorship: Fleming & Wood, 2015; **Location:** continent: Central America; country: Costa Rica; countryCode: CR; stateProvince: Guanacaste; county: Area de Conservacion Guanacaste; locality: Sector Mundo Nuevo; verbatimLocality: Sendero Aguacate; verbatimElevation: 335; verbatimLatitude: 10.769; verbatimLongitude: -85.435; verbatimCoordinateSystem: Decimal; decimalLatitude: 10.769; decimalLongitude: -85.435; **Identification:** identifiedBy: AJ Fleming; dateIdentified: 2014; **Event:** samplingProtocol: reared from caterpillar of *Talides sergestus* (Hesperiidae); verbatimEventDate: 03-Sep-2007; **Record Level:** language: en; institutionCode: CNC; collectionCode: Insects; basisOfRecord: Pinned Specimen
- **Type status:** Paratype. **Occurrence:** occurrenceDetails: http://janzen.sas.upenn.edu; catalogNumber: DHJPAR0022859; recordedBy: D.H. Janzen & W. Hallwachs; individualID: DHJPAR0022859; individualCount: 1; lifeStage: adult; preparations: pinned; otherCatalogNumbers: 07-SRNP-58809; **Taxon:** scientificName: Spathidexia marioburgosi; phylum: Arthropoda; class: Insecta; order: Diptera; family: Tachinidae; genus: Spathidexia; specificEpithet: marioburgosi; scientificNameAuthorship: Fleming & Wood, 2015; **Location:** continent: Central America; country: Costa Rica; countryCode: CR; stateProvince: Guanacaste; county: Area de Conservacion Guanacaste; locality: Sector Mundo Nuevo; verbatimLocality: Vado Miramonte; verbatimElevation: 305; verbatimLatitude: 10.772; verbatimLongitude: -85.434; verbatimCoordinateSystem: Decimal; decimalLatitude: 10.772; decimalLongitude: -85.434; **Identification:** identifiedBy: AJ Fleming; dateIdentified: 2014; **Event:** samplingProtocol: reared from caterpillar of *Talides* Burns04 (Hesperiidae); verbatimEventDate: 17-Aug-2007; **Record Level:** language: en; institutionCode: CNC; collectionCode: Insects; basisOfRecord: Pinned Specimen
- **Type status:** Paratype. **Occurrence:** occurrenceDetails: http://janzen.sas.upenn.edu; catalogNumber: DHJPAR0022860; recordedBy: D.H. Janzen & W. Hallwachs; individualID: DHJPAR0022860; individualCount: 1; lifeStage: adult; preparations: pinned; otherCatalogNumbers: 00-SRNP-12307; **Taxon:** scientificName: Spathidexia marioburgosi; phylum: Arthropoda; class: Insecta; order: Diptera; family: Tachinidae; genus: Spathidexia; specificEpithet: marioburgosi; scientificNameAuthorship: Fleming & Wood, 2015; **Location:** continent: Central America; country: Costa Rica; countryCode: CR; stateProvince: Alajuela; county: Area de Conservacion Guanacaste; locality: Sector San Cristobal; verbatimLocality: Quebrada Cementerio; verbatimElevation: 700; verbatimLatitude: 10.871; verbatimLongitude: -85.387; verbatimCoordinateSystem: Decimal; decimalLatitude: 10.871; decimalLongitude: -85.387; **Identification:** identifiedBy: AJ Fleming; dateIdentified: 2014; **Event:** samplingProtocol: reared from caterpillar of *Talides* Burns04 (Hesperiidae); verbatimEventDate: 19-Sep-2000; **Record Level:** language: en; institutionCode: CNC; collectionCode: Insects; basisOfRecord: Pinned Specimen
- **Type status:** Paratype. **Occurrence:** occurrenceDetails: http://janzen.sas.upenn.edu; catalogNumber: DHJPAR0022861; recordedBy: D.H. Janzen & W. Hallwachs; individualID: DHJPAR0022861; individualCount: 1; lifeStage: adult; preparations: pinned; otherCatalogNumbers: 00-SRNP-12306; **Taxon:** scientificName: Spathidexia marioburgosi; phylum: Arthropoda; class: Insecta; order: Diptera; family: Tachinidae; genus: Spathidexia; specificEpithet: marioburgosi; scientificNameAuthorship: Fleming & Wood, 2015; **Location:** continent: Central America; country: Costa Rica; countryCode: CR; stateProvince: Alajuela; county: Area de Conservacion Guanacaste; locality: Sector San Cristobal; verbatimLocality: Quebrada Cementerio; verbatimElevation: 700; verbatimLatitude: 10.871; verbatimLongitude: -85.387; verbatimCoordinateSystem: Decimal; decimalLatitude: 10.871; decimalLongitude: -85.387; **Identification:** identifiedBy: AJ Fleming; dateIdentified: 2014; **Event:** samplingProtocol: reared from caterpillar of *Talides* Burns04 (Hesperiidae); verbatimEventDate: 15-Sep-2000; **Record Level:** language: en; institutionCode: CNC; collectionCode: Insects; basisOfRecord: Pinned Specimen
- **Type status:** Paratype. **Occurrence:** occurrenceDetails: http://janzen.sas.upenn.edu; catalogNumber: DHJPAR0022862; recordedBy: D.H. Janzen & W. Hallwachs; individualID: DHJPAR0022862; individualCount: 1; lifeStage: adult; preparations: pinned; otherCatalogNumbers: 00-SRNP-12303; **Taxon:** scientificName: Spathidexia marioburgosi; phylum: Arthropoda; class: Insecta; order: Diptera; family: Tachinidae; genus: Spathidexia; specificEpithet: marioburgosi; scientificNameAuthorship: Fleming & Wood, 2015; **Location:** continent: Central America; country: Costa Rica; countryCode: CR; stateProvince: Alajuela; county: Area de Conservacion Guanacaste; locality: Sector San Cristobal; verbatimLocality: Quebrada Cementerio; verbatimElevation: 700; verbatimLatitude: 10.871; verbatimLongitude: -85.387; verbatimCoordinateSystem: Decimal; decimalLatitude: 10.871; decimalLongitude: -85.387; **Identification:** identifiedBy: AJ Fleming; dateIdentified: 2014; **Event:** samplingProtocol: reared from caterpillar of *Talides sinois* (Hesperiidae); verbatimEventDate: 14-Sep-2000; **Record Level:** language: en; institutionCode: CNC; collectionCode: Insects; basisOfRecord: Pinned Specimen
- **Type status:** Paratype. **Occurrence:** occurrenceDetails: http://janzen.sas.upenn.edu; catalogNumber: DHJPAR0022863; recordedBy: D.H. Janzen & W. Hallwachs; individualID: DHJPAR0022863; individualCount: 1; lifeStage: adult; preparations: pinned; otherCatalogNumbers: 00-SRNP-20936; **Taxon:** scientificName: Spathidexia marioburgosi; phylum: Arthropoda; class: Insecta; order: Diptera; family: Tachinidae; genus: Spathidexia; specificEpithet: marioburgosi; scientificNameAuthorship: Fleming & Wood, 2015; **Location:** continent: Central America; country: Costa Rica; countryCode: CR; stateProvince: Alajuela; county: Area de Conservacion Guanacaste; locality: Sector Rincon Rain Forest; verbatimLocality: Camino Rio Francia; verbatimElevation: 410; verbatimLatitude: 10.904; verbatimLongitude: -85.287; verbatimCoordinateSystem: Decimal; decimalLatitude: 10.904; decimalLongitude: -85.287; **Identification:** identifiedBy: AJ Fleming; dateIdentified: 2014; **Event:** samplingProtocol: reared from caterpillar of *Talides sinois* (Hesperiidae); verbatimEventDate: 06-Dec-2000; **Record Level:** language: en; institutionCode: CNC; collectionCode: Insects; basisOfRecord: Pinned Specimen
- **Type status:** Paratype. **Occurrence:** occurrenceDetails: http://janzen.sas.upenn.edu; catalogNumber: DHJPAR0022864; recordedBy: D.H. Janzen & W. Hallwachs; individualID: DHJPAR0022864; individualCount: 1; lifeStage: adult; preparations: pinned; otherCatalogNumbers: 00-SRNP-20919; **Taxon:** scientificName: Spathidexia marioburgosi; phylum: Arthropoda; class: Insecta; order: Diptera; family: Tachinidae; genus: Spathidexia; specificEpithet: marioburgosi; scientificNameAuthorship: Fleming & Wood, 2015; **Location:** continent: Central America; country: Costa Rica; countryCode: CR; stateProvince: Alajuela; county: Area de Conservacion Guanacaste; locality: Sector Rincon Rain Forest; verbatimLocality: Camino Rio Francia; verbatimElevation: 410; verbatimLatitude: 10.904; verbatimLongitude: -85.287; verbatimCoordinateSystem: Decimal; decimalLatitude: 10.904; decimalLongitude: -85.287; **Identification:** identifiedBy: AJ Fleming; dateIdentified: 2014; **Event:** samplingProtocol: reared from caterpillar of *Talides sinois* (Hesperiidae); verbatimEventDate: 12-Dec-2000; **Record Level:** language: en; institutionCode: CNC; collectionCode: Insects; basisOfRecord: Pinned Specimen
- **Type status:** Paratype. **Occurrence:** occurrenceDetails: http://janzen.sas.upenn.edu; catalogNumber: DHJPAR0022865; recordedBy: D.H. Janzen & W. Hallwachs; individualID: DHJPAR0022865; individualCount: 1; lifeStage: adult; preparations: pinned; otherCatalogNumbers: 00-SRNP-10863; **Taxon:** scientificName: Spathidexia marioburgosi; phylum: Arthropoda; class: Insecta; order: Diptera; family: Tachinidae; genus: Spathidexia; specificEpithet: marioburgosi; scientificNameAuthorship: Fleming & Wood, 2015; **Location:** continent: Central America; country: Costa Rica; countryCode: CR; stateProvince: Guanacaste; county: Area de Conservacion Guanacaste; locality: Sector Cacao; verbatimLocality: Sendero Toma Agua; verbatimElevation: 1140; verbatimLatitude: 10.928; verbatimLongitude: -85.467; verbatimCoordinateSystem: Decimal; decimalLatitude: 10.928; decimalLongitude: -85.467; **Identification:** identifiedBy: AJ Fleming; dateIdentified: 2014; **Event:** samplingProtocol: reared from caterpillar of *Talides sinois* (Hesperiidae); verbatimEventDate: 23-Nov-2000; **Record Level:** language: en; institutionCode: CNC; collectionCode: Insects; basisOfRecord: Pinned Specimen
- **Type status:** Paratype. **Occurrence:** occurrenceDetails: http://janzen.sas.upenn.edu; catalogNumber: DHJPAR0022866; recordedBy: D.H. Janzen & W. Hallwachs; individualID: DHJPAR0022866; individualCount: 1; lifeStage: adult; preparations: pinned; otherCatalogNumbers: 00-SRNP-21776; **Taxon:** scientificName: Spathidexia marioburgosi; phylum: Arthropoda; class: Insecta; order: Diptera; family: Tachinidae; genus: Spathidexia; specificEpithet: marioburgosi; scientificNameAuthorship: Fleming & Wood, 2015; **Location:** continent: Central America; country: Costa Rica; countryCode: CR; stateProvince: Alajuela; county: Area de Conservacion Guanacaste; locality: Sector San Cristobal; verbatimLocality: Quebrada Cementerio; verbatimElevation: 700; verbatimLatitude: 10.871; verbatimLongitude: -85.387; verbatimCoordinateSystem: Decimal; decimalLatitude: 10.871; decimalLongitude: -85.387; **Identification:** identifiedBy: AJ Fleming; dateIdentified: 2014; **Event:** samplingProtocol: reared from caterpillar of *Talides* Burns04 (Hesperiidae); verbatimEventDate: 30-Nov-2000; **Record Level:** language: en; institutionCode: CNC; collectionCode: Insects; basisOfRecord: Pinned Specimen
- **Type status:** Paratype. **Occurrence:** occurrenceDetails: http://janzen.sas.upenn.edu; catalogNumber: DHJPAR0022867; recordedBy: D.H. Janzen & W. Hallwachs; individualID: DHJPAR0022867; individualCount: 1; lifeStage: adult; preparations: pinned; otherCatalogNumbers: 00-SRNP-10300; **Taxon:** scientificName: Spathidexia marioburgosi; phylum: Arthropoda; class: Insecta; order: Diptera; family: Tachinidae; genus: Spathidexia; specificEpithet: marioburgosi; scientificNameAuthorship: Fleming & Wood, 2015; **Location:** continent: Central America; country: Costa Rica; countryCode: CR; stateProvince: Guanacaste; county: Area de Conservacion Guanacaste; locality: Sector Cacao; verbatimLocality: Cuesta Caimito; verbatimElevation: 640; verbatimLatitude: 10.891; verbatimLongitude: -85.472; verbatimCoordinateSystem: Decimal; decimalLatitude: 10.891; decimalLongitude: -85.472; **Identification:** identifiedBy: AJ Fleming; dateIdentified: 2014; **Event:** samplingProtocol: reared from caterpillar of *Talides sergestus* (Hesperiidae); verbatimEventDate: 09-Sep-2000; **Record Level:** language: en; institutionCode: CNC; collectionCode: Insects; basisOfRecord: Pinned Specimen
- **Type status:** Paratype. **Occurrence:** occurrenceDetails: http://janzen.sas.upenn.edu; catalogNumber: DHJPAR0022868; recordedBy: D.H. Janzen & W. Hallwachs; individualID: DHJPAR0022868; individualCount: 1; lifeStage: adult; preparations: pinned; otherCatalogNumbers: 99-SRNP-15104; **Taxon:** scientificName: Spathidexia marioburgosi; phylum: Arthropoda; class: Insecta; order: Diptera; family: Tachinidae; genus: Spathidexia; specificEpithet: marioburgosi; scientificNameAuthorship: Fleming & Wood, 2015; **Location:** continent: Central America; country: Costa Rica; countryCode: CR; stateProvince: Guanacaste; county: Area de Conservacion Guanacaste; locality: Sector Del Oro; verbatimLocality: Quebrada Serrano; verbatimElevation: 585; verbatimLatitude: 11; verbatimLongitude: -85.456; verbatimCoordinateSystem: Decimal; decimalLatitude: 11; decimalLongitude: -85.456; **Identification:** identifiedBy: AJ Fleming; dateIdentified: 2014; **Event:** samplingProtocol: reared from caterpillar of *Talides sinois* (Hesperiidae); verbatimEventDate: 05-Nov-1999; **Record Level:** language: en; institutionCode: CNC; collectionCode: Insects; basisOfRecord: Pinned Specimen
- **Type status:** Paratype. **Occurrence:** occurrenceDetails: http://janzen.sas.upenn.edu; catalogNumber: DHJPAR0022869; recordedBy: D.H. Janzen & W. Hallwachs; individualID: DHJPAR0022869; individualCount: 1; lifeStage: adult; preparations: pinned; otherCatalogNumbers: 04-SRNP-48259; **Taxon:** scientificName: Spathidexia marioburgosi; phylum: Arthropoda; class: Insecta; order: Diptera; family: Tachinidae; genus: Spathidexia; specificEpithet: marioburgosi; scientificNameAuthorship: Fleming & Wood, 2015; **Location:** continent: Central America; country: Costa Rica; countryCode: CR; stateProvince: Guanacaste; county: Area de Conservacion Guanacaste; locality: Sector Cacao; verbatimLocality: Gongora Bananal; verbatimElevation: 600; verbatimLatitude: 10.889; verbatimLongitude: -85.476; verbatimCoordinateSystem: Decimal; decimalLatitude: 10.889; decimalLongitude: -85.476; **Identification:** identifiedBy: AJ Fleming; dateIdentified: 2014; **Event:** samplingProtocol: reared from caterpillar of *Talides sinois* (Hesperiidae); verbatimEventDate: 22-Sep-2004; **Record Level:** language: en; institutionCode: CNC; collectionCode: Insects; basisOfRecord: Pinned Specimen
- **Type status:** Paratype. **Occurrence:** occurrenceDetails: http://janzen.sas.upenn.edu; catalogNumber: DHJPAR0022870; recordedBy: D.H. Janzen & W. Hallwachs; individualID: DHJPAR0022870; individualCount: 1; lifeStage: adult; preparations: pinned; otherCatalogNumbers: 04-SRNP-48421; **Taxon:** scientificName: Spathidexia marioburgosi; phylum: Arthropoda; class: Insecta; order: Diptera; family: Tachinidae; genus: Spathidexia; specificEpithet: marioburgosi; scientificNameAuthorship: Fleming & Wood, 2015; **Location:** continent: Central America; country: Costa Rica; countryCode: CR; stateProvince: Guanacaste; county: Area de Conservacion Guanacaste; locality: Sector Cacao; verbatimLocality: Quebrada Heliconia; verbatimElevation: 390; verbatimLatitude: 10.886; verbatimLongitude: -85.492; verbatimCoordinateSystem: Decimal; decimalLatitude: 10.886; decimalLongitude: -85.492; **Identification:** identifiedBy: AJ Fleming; dateIdentified: 2014; **Event:** samplingProtocol: reared from caterpillar of *Talides sinois* (Hesperiidae); verbatimEventDate: 30-Sep-2004; **Record Level:** language: en; institutionCode: CNC; collectionCode: Insects; basisOfRecord: Pinned Specimen
- **Type status:** Paratype. **Occurrence:** occurrenceDetails: http://janzen.sas.upenn.edu; catalogNumber: DHJPAR0022871; recordedBy: D.H. Janzen & W. Hallwachs; individualID: DHJPAR0022871; individualCount: 1; lifeStage: adult; preparations: pinned; otherCatalogNumbers: 04-SRNP-48245; **Taxon:** scientificName: Spathidexia marioburgosi; phylum: Arthropoda; class: Insecta; order: Diptera; family: Tachinidae; genus: Spathidexia; specificEpithet: marioburgosi; scientificNameAuthorship: Fleming & Wood, 2015; **Location:** continent: Central America; country: Costa Rica; countryCode: CR; stateProvince: Guanacaste; county: Area de Conservacion Guanacaste; locality: Sector Cacao; verbatimLocality: Gongora Bananal; verbatimElevation: 600; verbatimLatitude: 10.889; verbatimLongitude: -85.476; verbatimCoordinateSystem: Decimal; decimalLatitude: 10.889; decimalLongitude: -85.476; **Identification:** identifiedBy: AJ Fleming; dateIdentified: 2014; **Event:** samplingProtocol: reared from caterpillar of *Talides sinois* (Hesperiidae); verbatimEventDate: 23-Sep-2004; **Record Level:** language: en; institutionCode: CNC; collectionCode: Insects; basisOfRecord: Pinned Specimen
- **Type status:** Paratype. **Occurrence:** occurrenceDetails: http://janzen.sas.upenn.edu; catalogNumber: DHJPAR0022872; recordedBy: D.H. Janzen & W. Hallwachs; individualID: DHJPAR0022872; individualCount: 1; lifeStage: adult; preparations: pinned; otherCatalogNumbers: 00-SRNP-11612; **Taxon:** scientificName: Spathidexia marioburgosi; phylum: Arthropoda; class: Insecta; order: Diptera; family: Tachinidae; genus: Spathidexia; specificEpithet: marioburgosi; scientificNameAuthorship: Fleming & Wood, 2015; **Location:** continent: Central America; country: Costa Rica; countryCode: CR; stateProvince: Alajuela; county: Area de Conservacion Guanacaste; locality: Sector San Cristobal; verbatimLocality: Rio Blanco Abajo; verbatimElevation: 500; verbatimLatitude: 10.9; verbatimLongitude: -85.373; verbatimCoordinateSystem: Decimal; decimalLatitude: 10.9; decimalLongitude: -85.373; **Identification:** identifiedBy: AJ Fleming; dateIdentified: 2014; **Event:** samplingProtocol: reared from caterpillar of *Talides sinois* (Hesperiidae); verbatimEventDate: 04-Aug-2000; **Record Level:** language: en; institutionCode: CNC; collectionCode: Insects; basisOfRecord: Pinned Specimen
- **Type status:** Paratype. **Occurrence:** occurrenceDetails: http://janzen.sas.upenn.edu; catalogNumber: DHJPAR0022873; recordedBy: D.H. Janzen & W. Hallwachs; individualID: DHJPAR0022873; individualCount: 1; lifeStage: adult; preparations: pinned; otherCatalogNumbers: 04-SRNP-47061; **Taxon:** scientificName: Spathidexia marioburgosi; phylum: Arthropoda; class: Insecta; order: Diptera; family: Tachinidae; genus: Spathidexia; specificEpithet: marioburgosi; scientificNameAuthorship: Fleming & Wood, 2015; **Location:** continent: Central America; country: Costa Rica; countryCode: CR; stateProvince: Guanacaste; county: Area de Conservacion Guanacaste; locality: Sector Cacao; verbatimLocality: Cuesta Caimito; verbatimElevation: 640; verbatimLatitude: 10.891; verbatimLongitude: -85.472; verbatimCoordinateSystem: Decimal; decimalLatitude: 10.891; decimalLongitude: -85.472; **Identification:** identifiedBy: AJ Fleming; dateIdentified: 2014; **Event:** samplingProtocol: reared from caterpillar of *Talides sinois* (Hesperiidae); verbatimEventDate: 10-Aug-2004; **Record Level:** language: en; institutionCode: CNC; collectionCode: Insects; basisOfRecord: Pinned Specimen
- **Type status:** Paratype. **Occurrence:** occurrenceDetails: http://janzen.sas.upenn.edu; catalogNumber: DHJPAR0022874; recordedBy: D.H. Janzen & W. Hallwachs; individualID: DHJPAR0022874; individualCount: 1; lifeStage: adult; preparations: pinned; otherCatalogNumbers: 04-SRNP-47065; **Taxon:** scientificName: Spathidexia marioburgosi; phylum: Arthropoda; class: Insecta; order: Diptera; family: Tachinidae; genus: Spathidexia; specificEpithet: marioburgosi; scientificNameAuthorship: Fleming & Wood, 2015; **Location:** continent: Central America; country: Costa Rica; countryCode: CR; stateProvince: Guanacaste; county: Area de Conservacion Guanacaste; locality: Sector Cacao; verbatimLocality: Cuesta Caimito; verbatimElevation: 640; verbatimLatitude: 10.891; verbatimLongitude: -85.472; verbatimCoordinateSystem: Decimal; decimalLatitude: 10.891; decimalLongitude: -85.472; **Identification:** identifiedBy: AJ Fleming; dateIdentified: 2014; **Event:** samplingProtocol: reared from caterpillar of *Talides sinois* (Hesperiidae); verbatimEventDate: 09-Aug-2004; **Record Level:** language: en; institutionCode: CNC; collectionCode: Insects; basisOfRecord: Pinned Specimen
- **Type status:** Paratype. **Occurrence:** occurrenceDetails: http://janzen.sas.upenn.edu; catalogNumber: DHJPAR0022875; recordedBy: D.H. Janzen & W. Hallwachs; individualID: DHJPAR0022875; individualCount: 1; lifeStage: adult; preparations: pinned; otherCatalogNumbers: 03-SRNP-18879; **Taxon:** scientificName: Spathidexia marioburgosi; phylum: Arthropoda; class: Insecta; order: Diptera; family: Tachinidae; genus: Spathidexia; specificEpithet: marioburgosi; scientificNameAuthorship: Fleming & Wood, 2015; **Location:** continent: Central America; country: Costa Rica; countryCode: CR; stateProvince: Guanacaste; county: Area de Conservacion Guanacaste; locality: Sector Del Oro; verbatimLocality: Camino Mangos; verbatimElevation: 480; verbatimLatitude: 11.008; verbatimLongitude: -85.479; verbatimCoordinateSystem: Decimal; decimalLatitude: 11.008; decimalLongitude: -85.479; **Identification:** identifiedBy: AJ Fleming; dateIdentified: 2014; **Event:** samplingProtocol: reared from caterpillar of *Talides sergestus* (Hesperiidae); verbatimEventDate: 05-Sep-2003; **Record Level:** language: en; institutionCode: CNC; collectionCode: Insects; basisOfRecord: Pinned Specimen
- **Type status:** Paratype. **Occurrence:** occurrenceDetails: http://janzen.sas.upenn.edu; catalogNumber: DHJPAR0022876; recordedBy: D.H. Janzen & W. Hallwachs; individualID: DHJPAR0022876; individualCount: 1; lifeStage: adult; preparations: pinned; otherCatalogNumbers: 03-SRNP-28308; **Taxon:** scientificName: Spathidexia marioburgosi; phylum: Arthropoda; class: Insecta; order: Diptera; family: Tachinidae; genus: Spathidexia; specificEpithet: marioburgosi; scientificNameAuthorship: Fleming & Wood, 2015; **Location:** continent: Central America; country: Costa Rica; countryCode: CR; stateProvince: Guanacaste; county: Area de Conservacion Guanacaste; locality: Sector Del Oro; verbatimLocality: Uncaria; verbatimElevation: 370; verbatimLatitude: 11.018; verbatimLongitude: -85.474; verbatimCoordinateSystem: Decimal; decimalLatitude: 11.018; decimalLongitude: -85.474; **Identification:** identifiedBy: AJ Fleming; dateIdentified: 2014; **Event:** samplingProtocol: reared from caterpillar of *Talides sinois* (Hesperiidae); verbatimEventDate: 06-Oct-2003; **Record Level:** language: en; institutionCode: CNC; collectionCode: Insects; basisOfRecord: Pinned Specimen
- **Type status:** Paratype. **Occurrence:** occurrenceDetails: http://janzen.sas.upenn.edu; catalogNumber: DHJPAR0022877; recordedBy: D.H. Janzen & W. Hallwachs; individualID: DHJPAR0022877; individualCount: 1; lifeStage: adult; preparations: pinned; otherCatalogNumbers: 03-SRNP-12458.1; **Taxon:** scientificName: Spathidexia marioburgosi; phylum: Arthropoda; class: Insecta; order: Diptera; family: Tachinidae; genus: Spathidexia; specificEpithet: marioburgosi; scientificNameAuthorship: Fleming & Wood, 2015; **Location:** continent: Central America; country: Costa Rica; countryCode: CR; stateProvince: Alajuela; county: Area de Conservacion Guanacaste; locality: Sector Rincon Rain Forest; verbatimLocality: Camino Porvenir; verbatimElevation: 383; verbatimLatitude: 10.904; verbatimLongitude: -85.26; verbatimCoordinateSystem: Decimal; decimalLatitude: 10.904; decimalLongitude: -85.26; **Identification:** identifiedBy: AJ Fleming; dateIdentified: 2014; **Event:** samplingProtocol: reared from caterpillar of *Talides* Burns04 (Hesperiidae); verbatimEventDate: 08-Oct-2003; **Record Level:** language: en; institutionCode: CNC; collectionCode: Insects; basisOfRecord: Pinned Specimen
- **Type status:** Paratype. **Occurrence:** occurrenceDetails: http://janzen.sas.upenn.edu; catalogNumber: DHJPAR0022878; recordedBy: D.H. Janzen & W. Hallwachs; individualID: DHJPAR0022878; individualCount: 1; lifeStage: adult; preparations: pinned; otherCatalogNumbers: 03-SRNP-19475; **Taxon:** scientificName: Spathidexia marioburgosi; phylum: Arthropoda; class: Insecta; order: Diptera; family: Tachinidae; genus: Spathidexia; specificEpithet: marioburgosi; scientificNameAuthorship: Fleming & Wood, 2015; **Location:** continent: Central America; country: Costa Rica; countryCode: CR; stateProvince: Guanacaste; county: Area de Conservacion Guanacaste; locality: Sector Del Oro; verbatimLocality: Quebrada Trigal; verbatimElevation: 290; verbatimLatitude: 11.027; verbatimLongitude: -85.495; verbatimCoordinateSystem: Decimal; decimalLatitude: 11.027; decimalLongitude: -85.495; **Identification:** identifiedBy: AJ Fleming; dateIdentified: 2014; **Event:** samplingProtocol: reared from caterpillar of *Talides* Burns04 (Hesperiidae); verbatimEventDate: 15-Sep-2003; **Record Level:** language: en; institutionCode: CNC; collectionCode: Insects; basisOfRecord: Pinned Specimen
- **Type status:** Paratype. **Occurrence:** occurrenceDetails: http://janzen.sas.upenn.edu; catalogNumber: DHJPAR0022879; recordedBy: D.H. Janzen & W. Hallwachs; individualID: DHJPAR0022879; individualCount: 1; lifeStage: adult; preparations: pinned; otherCatalogNumbers: 03-SRNP-19466; **Taxon:** scientificName: Spathidexia marioburgosi; phylum: Arthropoda; class: Insecta; order: Diptera; family: Tachinidae; genus: Spathidexia; specificEpithet: marioburgosi; scientificNameAuthorship: Fleming & Wood, 2015; **Location:** continent: Central America; country: Costa Rica; countryCode: CR; stateProvince: Guanacaste; county: Area de Conservacion Guanacaste; locality: Sector Del Oro; verbatimLocality: Quebrada Trigal; verbatimElevation: 290; verbatimLatitude: 11.027; verbatimLongitude: -85.495; verbatimCoordinateSystem: Decimal; decimalLatitude: 11.027; decimalLongitude: -85.495; **Identification:** identifiedBy: AJ Fleming; dateIdentified: 2014; **Event:** samplingProtocol: reared from caterpillar of *Talides sinois* (Hesperiidae); verbatimEventDate: 12-Sep-2003; **Record Level:** language: en; institutionCode: CNC; collectionCode: Insects; basisOfRecord: Pinned Specimen
- **Type status:** Paratype. **Occurrence:** occurrenceDetails: http://janzen.sas.upenn.edu; catalogNumber: DHJPAR0022880; recordedBy: D.H. Janzen & W. Hallwachs; individualID: DHJPAR0022880; individualCount: 1; lifeStage: adult; preparations: pinned; otherCatalogNumbers: 03-SRNP-7910; **Taxon:** scientificName: Spathidexia marioburgosi; phylum: Arthropoda; class: Insecta; order: Diptera; family: Tachinidae; genus: Spathidexia; specificEpithet: marioburgosi; scientificNameAuthorship: Fleming & Wood, 2015; **Location:** continent: Central America; country: Costa Rica; countryCode: CR; stateProvince: Alajuela; county: Area de Conservacion Guanacaste; locality: Sector San Cristobal; verbatimLocality: Rio Blanco Abajo; verbatimElevation: 500; verbatimLatitude: 10.9; verbatimLongitude: -85.373; verbatimCoordinateSystem: Decimal; decimalLatitude: 10.9; decimalLongitude: -85.373; **Identification:** identifiedBy: AJ Fleming; dateIdentified: 2014; **Event:** samplingProtocol: reared from caterpillar of *Talides* Burns04 (Hesperiidae); verbatimEventDate: 22-Sep-2003; **Record Level:** language: en; institutionCode: CNC; collectionCode: Insects; basisOfRecord: Pinned Specimen
- **Type status:** Paratype. **Occurrence:** occurrenceDetails: http://janzen.sas.upenn.edu; catalogNumber: DHJPAR0022881; recordedBy: D.H. Janzen & W. Hallwachs; individualID: DHJPAR0022881; individualCount: 1; lifeStage: adult; preparations: pinned; otherCatalogNumbers: 03-SRNP-19471; **Taxon:** scientificName: Spathidexia marioburgosi; phylum: Arthropoda; class: Insecta; order: Diptera; family: Tachinidae; genus: Spathidexia; specificEpithet: marioburgosi; scientificNameAuthorship: Fleming & Wood, 2015; **Location:** continent: Central America; country: Costa Rica; countryCode: CR; stateProvince: Guanacaste; county: Area de Conservacion Guanacaste; locality: Sector Del Oro; verbatimLocality: Quebrada Trigal; verbatimElevation: 290; verbatimLatitude: 11.027; verbatimLongitude: -85.495; verbatimCoordinateSystem: Decimal; decimalLatitude: 11.027; decimalLongitude: -85.495; **Identification:** identifiedBy: AJ Fleming; dateIdentified: 2014; **Event:** samplingProtocol: reared from caterpillar of *Talides sinois* (Hesperiidae); verbatimEventDate: 05-Sep-2003; **Record Level:** language: en; institutionCode: CNC; collectionCode: Insects; basisOfRecord: Pinned Specimen
- **Type status:** Paratype. **Occurrence:** occurrenceDetails: http://janzen.sas.upenn.edu; catalogNumber: DHJPAR0022882; recordedBy: D.H. Janzen & W. Hallwachs; individualID: DHJPAR0022882; individualCount: 1; lifeStage: adult; preparations: pinned; otherCatalogNumbers: 04-SRNP-47065; **Taxon:** scientificName: Spathidexia marioburgosi; phylum: Arthropoda; class: Insecta; order: Diptera; family: Tachinidae; genus: Spathidexia; specificEpithet: marioburgosi; scientificNameAuthorship: Fleming & Wood, 2015; **Location:** continent: Central America; country: Costa Rica; countryCode: CR; stateProvince: Guanacaste; county: Area de Conservacion Guanacaste; locality: Sector Cacao; verbatimLocality: Cuesta Caimito; verbatimElevation: 640; verbatimLatitude: 10.891; verbatimLongitude: -85.472; verbatimCoordinateSystem: Decimal; decimalLatitude: 10.891; decimalLongitude: -85.472; **Identification:** identifiedBy: AJ Fleming; dateIdentified: 2014; **Event:** samplingProtocol: reared from caterpillar of *Talides sinois* (Hesperiidae); verbatimEventDate: 09-Aug-2004; **Record Level:** language: en; institutionCode: CNC; collectionCode: Insects; basisOfRecord: Pinned Specimen
- **Type status:** Paratype. **Occurrence:** occurrenceDetails: http://janzen.sas.upenn.edu; catalogNumber: DHJPAR0022883; recordedBy: D.H. Janzen & W. Hallwachs; individualID: DHJPAR0022883; individualCount: 1; lifeStage: adult; preparations: pinned; otherCatalogNumbers: 04-SRNP-1867; **Taxon:** scientificName: Spathidexia marioburgosi; phylum: Arthropoda; class: Insecta; order: Diptera; family: Tachinidae; genus: Spathidexia; specificEpithet: marioburgosi; scientificNameAuthorship: Fleming & Wood, 2015; **Location:** continent: Central America; country: Costa Rica; countryCode: CR; stateProvince: Alajuela; county: Area de Conservacion Guanacaste; locality: Sector San Cristobal; verbatimLocality: Cementerio Viejo; verbatimElevation: 570; verbatimLatitude: 10.881; verbatimLongitude: -85.389; verbatimCoordinateSystem: Decimal; decimalLatitude: 10.881; decimalLongitude: -85.389; **Identification:** identifiedBy: AJ Fleming; dateIdentified: 2014; **Event:** samplingProtocol: reared from caterpillar of *Talides sergestus* (Hesperiidae); verbatimEventDate: 11-May-2004; **Record Level:** language: en; institutionCode: CNC; collectionCode: Insects; basisOfRecord: Pinned Specimen
- **Type status:** Paratype. **Occurrence:** occurrenceDetails: http://janzen.sas.upenn.edu; catalogNumber: DHJPAR0022884; recordedBy: D.H. Janzen & W. Hallwachs; individualID: DHJPAR0022884; individualCount: 1; lifeStage: adult; preparations: pinned; otherCatalogNumbers: 04-SRNP-22687; **Taxon:** scientificName: Spathidexia marioburgosi; phylum: Arthropoda; class: Insecta; order: Diptera; family: Tachinidae; genus: Spathidexia; specificEpithet: marioburgosi; scientificNameAuthorship: Fleming & Wood, 2015; **Location:** continent: Central America; country: Costa Rica; countryCode: CR; stateProvince: Guanacaste; county: Area de Conservacion Guanacaste; locality: Sector Del Oro; verbatimLocality: Quebrada Suamposa; verbatimElevation: 290; verbatimLatitude: 11.024; verbatimLongitude: -85.491; verbatimCoordinateSystem: Decimal; decimalLatitude: 11.024; decimalLongitude: -85.491; **Identification:** identifiedBy: AJ Fleming; dateIdentified: 2014; **Event:** samplingProtocol: reared from caterpillar of *Talides sinois* (Hesperiidae); verbatimEventDate: 04-Jul-2004; **Record Level:** language: en; institutionCode: CNC; collectionCode: Insects; basisOfRecord: Pinned Specimen
- **Type status:** Paratype. **Occurrence:** occurrenceDetails: http://janzen.sas.upenn.edu; catalogNumber: DHJPAR0022885; recordedBy: D.H. Janzen & W. Hallwachs; individualID: DHJPAR0022885; individualCount: 1; lifeStage: adult; preparations: pinned; otherCatalogNumbers: 03-SRNP-8968; **Taxon:** scientificName: Spathidexia marioburgosi; phylum: Arthropoda; class: Insecta; order: Diptera; family: Tachinidae; genus: Spathidexia; specificEpithet: marioburgosi; scientificNameAuthorship: Fleming & Wood, 2015; **Location:** continent: Central America; country: Costa Rica; countryCode: CR; stateProvince: Alajuela; county: Area de Conservacion Guanacaste; locality: Sector San Cristobal; verbatimLocality: Rio Blanco Abajo; verbatimElevation: 500; verbatimLatitude: 10.9; verbatimLongitude: -85.373; verbatimCoordinateSystem: Decimal; decimalLatitude: 10.9; decimalLongitude: -85.373; **Identification:** identifiedBy: AJ Fleming; dateIdentified: 2014; **Event:** samplingProtocol: reared from caterpillar of *Talides sinois* (Hesperiidae); verbatimEventDate: 04-Nov-2003; **Record Level:** language: en; institutionCode: CNC; collectionCode: Insects; basisOfRecord: Pinned Specimen
- **Type status:** Paratype. **Occurrence:** occurrenceDetails: http://janzen.sas.upenn.edu; catalogNumber: DHJPAR0022886; recordedBy: D.H. Janzen & W. Hallwachs; individualID: DHJPAR0022886; individualCount: 1; lifeStage: adult; preparations: pinned; otherCatalogNumbers: 04-SRNP-95; **Taxon:** scientificName: Spathidexia marioburgosi; phylum: Arthropoda; class: Insecta; order: Diptera; family: Tachinidae; genus: Spathidexia; specificEpithet: marioburgosi; scientificNameAuthorship: Fleming & Wood, 2015; **Location:** continent: Central America; country: Costa Rica; countryCode: CR; stateProvince: Alajuela; county: Area de Conservacion Guanacaste; locality: Sector San Cristobal; verbatimLocality: Finca San Gabriel; verbatimElevation: 645; verbatimLatitude: 10.878; verbatimLongitude: -85.393; verbatimCoordinateSystem: Decimal; decimalLatitude: 10.878; decimalLongitude: -85.393; **Identification:** identifiedBy: AJ Fleming; dateIdentified: 2014; **Event:** samplingProtocol: reared from caterpillar of *Talides sinois* (Hesperiidae); verbatimEventDate: 15-Feb-2003; **Record Level:** language: en; institutionCode: CNC; collectionCode: Insects; basisOfRecord: Pinned Specimen
- **Type status:** Paratype. **Occurrence:** occurrenceDetails: http://janzen.sas.upenn.edu; catalogNumber: DHJPAR0022887; recordedBy: D.H. Janzen & W. Hallwachs; individualID: DHJPAR0022887; individualCount: 1; lifeStage: adult; preparations: pinned; otherCatalogNumbers: 03-SRNP-29175; **Taxon:** scientificName: Spathidexia marioburgosi; phylum: Arthropoda; class: Insecta; order: Diptera; family: Tachinidae; genus: Spathidexia; specificEpithet: marioburgosi; scientificNameAuthorship: Fleming & Wood, 2015; **Location:** continent: Central America; country: Costa Rica; countryCode: CR; stateProvince: Guanacaste; county: Area de Conservacion Guanacaste; locality: Sector Del Oro; verbatimLocality: Quebrada Trigal; verbatimElevation: 290; verbatimLatitude: 11.027; verbatimLongitude: -85.495; verbatimCoordinateSystem: Decimal; decimalLatitude: 11.027; decimalLongitude: -85.495; **Identification:** identifiedBy: AJ Fleming; dateIdentified: 2014; **Event:** samplingProtocol: reared from caterpillar of *Talides sinois* (Hesperiidae); verbatimEventDate: 30-Oct-2003; **Record Level:** language: en; institutionCode: CNC; collectionCode: Insects; basisOfRecord: Pinned Specimen
- **Type status:** Paratype. **Occurrence:** occurrenceDetails: http://janzen.sas.upenn.edu; catalogNumber: DHJPAR0022888; recordedBy: D.H. Janzen & W. Hallwachs; individualID: DHJPAR0022888; individualCount: 1; lifeStage: adult; preparations: pinned; otherCatalogNumbers: 03-SRNP-29689; **Taxon:** scientificName: Spathidexia marioburgosi; phylum: Arthropoda; class: Insecta; order: Diptera; family: Tachinidae; genus: Spathidexia; specificEpithet: marioburgosi; scientificNameAuthorship: Fleming & Wood, 2015; **Location:** continent: Central America; country: Costa Rica; countryCode: CR; stateProvince: Guanacaste; county: Area de Conservacion Guanacaste; locality: Sector Del Oro; verbatimLocality: Margarita; verbatimElevation: 380; verbatimLatitude: 11.032; verbatimLongitude: -85.44; verbatimCoordinateSystem: Decimal; decimalLatitude: 11.032; decimalLongitude: -85.44; **Identification:** identifiedBy: AJ Fleming; dateIdentified: 2014; **Event:** samplingProtocol: reared from caterpillar of *Talides sinois* (Hesperiidae); verbatimEventDate: 07-Nov-2003; **Record Level:** language: en; institutionCode: CNC; collectionCode: Insects; basisOfRecord: Pinned Specimen
- **Type status:** Paratype. **Occurrence:** occurrenceDetails: http://janzen.sas.upenn.edu; catalogNumber: DHJPAR0022889; recordedBy: D.H. Janzen & W. Hallwachs; individualID: DHJPAR0022889; individualCount: 1; lifeStage: adult; preparations: pinned; otherCatalogNumbers: 03-SRNP-29255; **Taxon:** scientificName: Spathidexia marioburgosi; phylum: Arthropoda; class: Insecta; order: Diptera; family: Tachinidae; genus: Spathidexia; specificEpithet: marioburgosi; scientificNameAuthorship: Fleming & Wood, 2015; **Location:** continent: Central America; country: Costa Rica; countryCode: CR; stateProvince: Guanacaste; county: Area de Conservacion Guanacaste; locality: Sector Del Oro; verbatimLocality: Sendero Puertas; verbatimElevation: 400; verbatimLatitude: 11.011; verbatimLongitude: -85.488; verbatimCoordinateSystem: Decimal; decimalLatitude: 11.011; decimalLongitude: -85.488; **Identification:** identifiedBy: AJ Fleming; dateIdentified: 2014; **Event:** samplingProtocol: reared from caterpillar of *Talides* Burns02 (Hesperiidae); verbatimEventDate: 06-Nov-2003; **Record Level:** language: en; institutionCode: CNC; collectionCode: Insects; basisOfRecord: Pinned Specimen
- **Type status:** Paratype. **Occurrence:** occurrenceDetails: http://janzen.sas.upenn.edu; catalogNumber: DHJPAR0022890; recordedBy: D.H. Janzen & W. Hallwachs; individualID: DHJPAR0022890; individualCount: 1; lifeStage: adult; preparations: pinned; otherCatalogNumbers: 04-SRNP-30107; **Taxon:** scientificName: Spathidexia marioburgosi; phylum: Arthropoda; class: Insecta; order: Diptera; family: Tachinidae; genus: Spathidexia; specificEpithet: marioburgosi; scientificNameAuthorship: Fleming & Wood, 2015; **Location:** continent: Central America; country: Costa Rica; countryCode: CR; stateProvince: Guanacaste; county: Area de Conservacion Guanacaste; locality: Sector Pitilla; verbatimLocality: Pasmompa; verbatimElevation: 440; verbatimLatitude: 11.019; verbatimLongitude: -85.41; verbatimCoordinateSystem: Decimal; decimalLatitude: 11.019; decimalLongitude: -85.41; **Identification:** identifiedBy: AJ Fleming; dateIdentified: 2014; **Event:** samplingProtocol: reared from caterpillar of *Talides sinois* (Hesperiidae); verbatimEventDate: 05-Feb-2004; **Record Level:** language: en; institutionCode: CNC; collectionCode: Insects; basisOfRecord: Pinned Specimen
- **Type status:** Paratype. **Occurrence:** occurrenceDetails: http://janzen.sas.upenn.edu; catalogNumber: DHJPAR0022891; recordedBy: D.H. Janzen & W. Hallwachs; individualID: DHJPAR0022891; individualCount: 1; lifeStage: adult; preparations: pinned; otherCatalogNumbers: 04-SRNP-20014; **Taxon:** scientificName: Spathidexia marioburgosi; phylum: Arthropoda; class: Insecta; order: Diptera; family: Tachinidae; genus: Spathidexia; specificEpithet: marioburgosi; scientificNameAuthorship: Fleming & Wood, 2015; **Location:** continent: Central America; country: Costa Rica; countryCode: CR; stateProvince: Guanacaste; county: Area de Conservacion Guanacaste; locality: Sector Del Oro; verbatimLocality: Quebrada Lajosa; verbatimElevation: 400; verbatimLatitude: 11.033; verbatimLongitude: -85.429; verbatimCoordinateSystem: Decimal; decimalLatitude: 11.033; decimalLongitude: -85.429; **Identification:** identifiedBy: AJ Fleming; dateIdentified: 2014; **Event:** samplingProtocol: reared from caterpillar of *Talides sinois* (Hesperiidae); verbatimEventDate: 31-Jan-2004; **Record Level:** language: en; institutionCode: CNC; collectionCode: Insects; basisOfRecord: Pinned Specimen
- **Type status:** Paratype. **Occurrence:** occurrenceDetails: http://janzen.sas.upenn.edu; catalogNumber: DHJPAR0022892; recordedBy: D.H. Janzen & W. Hallwachs; individualID: DHJPAR0022892; individualCount: 1; lifeStage: adult; preparations: pinned; otherCatalogNumbers: 04-SRNP-1130; **Taxon:** scientificName: Spathidexia marioburgosi; phylum: Arthropoda; class: Insecta; order: Diptera; family: Tachinidae; genus: Spathidexia; specificEpithet: marioburgosi; scientificNameAuthorship: Fleming & Wood, 2015; **Location:** continent: Central America; country: Costa Rica; countryCode: CR; stateProvince: Alajuela; county: Area de Conservacion Guanacaste; locality: Sector San Cristobal; verbatimLocality: Puente Palma; verbatimElevation: 460; verbatimLatitude: 10.916; verbatimLongitude: -85.379; verbatimCoordinateSystem: Decimal; decimalLatitude: 10.916; decimalLongitude: -85.379; **Identification:** identifiedBy: AJ Fleming; dateIdentified: 2014; **Event:** samplingProtocol: reared from caterpillar of *Talides sinois* (Hesperiidae); verbatimEventDate: 31-Mar-2004; **Record Level:** language: en; institutionCode: CNC; collectionCode: Insects; basisOfRecord: Pinned Specimen
- **Type status:** Paratype. **Occurrence:** occurrenceDetails: http://janzen.sas.upenn.edu; catalogNumber: DHJPAR0022893; recordedBy: D.H. Janzen & W. Hallwachs; individualID: DHJPAR0022893; individualCount: 1; lifeStage: adult; preparations: pinned; otherCatalogNumbers: 03-SRNP-34139; **Taxon:** scientificName: Spathidexia marioburgosi; phylum: Arthropoda; class: Insecta; order: Diptera; family: Tachinidae; genus: Spathidexia; specificEpithet: marioburgosi; scientificNameAuthorship: Fleming & Wood, 2015; **Location:** continent: Central America; country: Costa Rica; countryCode: CR; stateProvince: Alajuela; county: Area de Conservacion Guanacaste; locality: Sector San Cristobal; verbatimLocality: Finca San Gabriel; verbatimElevation: 645; verbatimLatitude: 10.878; verbatimLongitude: -85.393; verbatimCoordinateSystem: Decimal; decimalLatitude: 10.878; decimalLongitude: -85.393; **Identification:** identifiedBy: AJ Fleming; dateIdentified: 2014; **Event:** samplingProtocol: reared from caterpillar of *Talides sinois* (Hesperiidae); verbatimEventDate: 12-Dec-2003; **Record Level:** language: en; institutionCode: CNC; collectionCode: Insects; basisOfRecord: Pinned Specimen
- **Type status:** Paratype. **Occurrence:** occurrenceDetails: http://janzen.sas.upenn.edu; catalogNumber: DHJPAR0022894; recordedBy: D.H. Janzen & W. Hallwachs; individualID: DHJPAR0022894; individualCount: 1; lifeStage: adult; preparations: pinned; otherCatalogNumbers: 04-SRNP-2436; **Taxon:** scientificName: Spathidexia marioburgosi; phylum: Arthropoda; class: Insecta; order: Diptera; family: Tachinidae; genus: Spathidexia; specificEpithet: marioburgosi; scientificNameAuthorship: Fleming & Wood, 2015; **Location:** continent: Central America; country: Costa Rica; countryCode: CR; stateProvince: Alajuela; county: Area de Conservacion Guanacaste; locality: Sector San Cristobal; verbatimLocality: Cementerio Viejo; verbatimElevation: 570; verbatimLatitude: 10.881; verbatimLongitude: -85.389; verbatimCoordinateSystem: Decimal; decimalLatitude: 10.881; decimalLongitude: -85.389; **Identification:** identifiedBy: AJ Fleming; dateIdentified: 2014; **Event:** samplingProtocol: reared from caterpillar of *Talides sergestus* (Hesperiidae); verbatimEventDate: 25-Jun-2004; **Record Level:** language: en; institutionCode: CNC; collectionCode: Insects; basisOfRecord: Pinned Specimen
- **Type status:** Paratype. **Occurrence:** occurrenceDetails: http://janzen.sas.upenn.edu; catalogNumber: DHJPAR0022895; recordedBy: D.H. Janzen & W. Hallwachs; individualID: DHJPAR0022895; individualCount: 1; lifeStage: adult; preparations: pinned; otherCatalogNumbers: 04-SRNP-22687; **Taxon:** scientificName: Spathidexia marioburgosi; phylum: Arthropoda; class: Insecta; order: Diptera; family: Tachinidae; genus: Spathidexia; specificEpithet: marioburgosi; scientificNameAuthorship: Fleming & Wood, 2015; **Location:** continent: Central America; country: Costa Rica; countryCode: CR; stateProvince: Guanacaste; county: Area de Conservacion Guanacaste; locality: Sector Del Oro; verbatimLocality: Quebrada Suamposa; verbatimElevation: 290; verbatimLatitude: 11.024; verbatimLongitude: -85.491; verbatimCoordinateSystem: Decimal; decimalLatitude: 11.024; decimalLongitude: -85.491; **Identification:** identifiedBy: AJ Fleming; dateIdentified: 2014; **Event:** samplingProtocol: reared from caterpillar of *Talides sinois* (Hesperiidae); verbatimEventDate: 04-Jul-2004; **Record Level:** language: en; institutionCode: CNC; collectionCode: Insects; basisOfRecord: Pinned Specimen
- **Type status:** Paratype. **Occurrence:** occurrenceDetails: http://janzen.sas.upenn.edu; catalogNumber: DHJPAR0022896; recordedBy: D.H. Janzen & W. Hallwachs; individualID: DHJPAR0022896; individualCount: 1; lifeStage: adult; preparations: pinned; otherCatalogNumbers: 04-SRNP-3396; **Taxon:** scientificName: Spathidexia marioburgosi; phylum: Arthropoda; class: Insecta; order: Diptera; family: Tachinidae; genus: Spathidexia; specificEpithet: marioburgosi; scientificNameAuthorship: Fleming & Wood, 2015; **Location:** continent: Central America; country: Costa Rica; countryCode: CR; stateProvince: Alajuela; county: Area de Conservacion Guanacaste; locality: Sector San Cristobal; verbatimLocality: Sendero Carmona; verbatimElevation: 670; verbatimLatitude: 10.876; verbatimLongitude: -85.386; verbatimCoordinateSystem: Decimal; decimalLatitude: 10.876; decimalLongitude: -85.386; **Identification:** identifiedBy: AJ Fleming; dateIdentified: 2014; **Event:** samplingProtocol: reared from caterpillar of *Talides sergestus* (Hesperiidae); verbatimEventDate: 02-Aug-2004; **Record Level:** language: en; institutionCode: CNC; collectionCode: Insects; basisOfRecord: Pinned Specimen
- **Type status:** Paratype. **Occurrence:** occurrenceDetails: http://janzen.sas.upenn.edu; catalogNumber: DHJPAR0022897; recordedBy: D.H. Janzen & W. Hallwachs; individualID: DHJPAR0022897; individualCount: 1; lifeStage: adult; preparations: pinned; otherCatalogNumbers: 04-SRNP-47058; **Taxon:** scientificName: Spathidexia marioburgosi; phylum: Arthropoda; class: Insecta; order: Diptera; family: Tachinidae; genus: Spathidexia; specificEpithet: marioburgosi; scientificNameAuthorship: Fleming & Wood, 2015; **Location:** continent: Central America; country: Costa Rica; countryCode: CR; stateProvince: Guanacaste; county: Area de Conservacion Guanacaste; locality: Sector Cacao; verbatimLocality: Cuesta Caimito; verbatimElevation: 640; verbatimLatitude: 10.891; verbatimLongitude: -85.472; verbatimCoordinateSystem: Decimal; decimalLatitude: 10.891; decimalLongitude: -85.472; **Identification:** identifiedBy: AJ Fleming; dateIdentified: 2014; **Event:** samplingProtocol: reared from caterpillar of *Talides sergestus* (Hesperiidae); verbatimEventDate: 30-Jul-2004; **Record Level:** language: en; institutionCode: CNC; collectionCode: Insects; basisOfRecord: Pinned Specimen
- **Type status:** Paratype. **Occurrence:** occurrenceDetails: http://janzen.sas.upenn.edu; catalogNumber: DHJPAR0022898; recordedBy: D.H. Janzen & W. Hallwachs; individualID: DHJPAR0022898; individualCount: 1; lifeStage: adult; preparations: pinned; otherCatalogNumbers: 02-SRNP-21538; **Taxon:** scientificName: Spathidexia marioburgosi; phylum: Arthropoda; class: Insecta; order: Diptera; family: Tachinidae; genus: Spathidexia; specificEpithet: marioburgosi; scientificNameAuthorship: Fleming & Wood, 2015; **Location:** continent: Central America; country: Costa Rica; countryCode: CR; stateProvince: Alajuela; county: Area de Conservacion Guanacaste; locality: Sector Rincon Rain Forest; verbatimLocality: Camino Rio Francia; verbatimElevation: 410; verbatimLatitude: 10.904; verbatimLongitude: -85.287; verbatimCoordinateSystem: Decimal; decimalLatitude: 10.904; decimalLongitude: -85.287; **Identification:** identifiedBy: AJ Fleming; dateIdentified: 2014; **Event:** samplingProtocol: reared from caterpillar of *Talides sinois* (Hesperiidae); verbatimEventDate: 25-Jan-2003; **Record Level:** language: en; institutionCode: CNC; collectionCode: Insects; basisOfRecord: Pinned Specimen
- **Type status:** Paratype. **Occurrence:** occurrenceDetails: http://janzen.sas.upenn.edu; catalogNumber: DHJPAR0022899; recordedBy: D.H. Janzen & W. Hallwachs; individualID: DHJPAR0022899; individualCount: 1; lifeStage: adult; preparations: pinned; otherCatalogNumbers: 02-SRNP-20361; **Taxon:** scientificName: Spathidexia marioburgosi; phylum: Arthropoda; class: Insecta; order: Diptera; family: Tachinidae; genus: Spathidexia; specificEpithet: marioburgosi; scientificNameAuthorship: Fleming & Wood, 2015; **Location:** continent: Central America; country: Costa Rica; countryCode: CR; stateProvince: Alajuela; county: Area de Conservacion Guanacaste; locality: Sector San Cristobal; verbatimLocality: Rio Blanco Abajo; verbatimElevation: 500; verbatimLatitude: 10.9; verbatimLongitude: -85.373; verbatimCoordinateSystem: Decimal; decimalLatitude: 10.9; decimalLongitude: -85.373; **Identification:** identifiedBy: AJ Fleming; dateIdentified: 2014; **Event:** samplingProtocol: reared from caterpillar of *Talides* Burns04 (Hesperiidae); verbatimEventDate: 12-Jan-2003; **Record Level:** language: en; institutionCode: CNC; collectionCode: Insects; basisOfRecord: Pinned Specimen
- **Type status:** Paratype. **Occurrence:** occurrenceDetails: http://janzen.sas.upenn.edu; catalogNumber: DHJPAR0022900; recordedBy: D.H. Janzen & W. Hallwachs; individualID: DHJPAR0022900; individualCount: 1; lifeStage: adult; preparations: pinned; otherCatalogNumbers: 02-SRNP-9934; **Taxon:** scientificName: Spathidexia marioburgosi; phylum: Arthropoda; class: Insecta; order: Diptera; family: Tachinidae; genus: Spathidexia; specificEpithet: marioburgosi; scientificNameAuthorship: Fleming & Wood, 2015; **Location:** continent: Central America; country: Costa Rica; countryCode: CR; stateProvince: Guanacaste; county: Area de Conservacion Guanacaste; locality: Sector Cacao; verbatimLocality: Sendero Toma Agua; verbatimElevation: 1140; verbatimLatitude: 10.928; verbatimLongitude: -85.467; verbatimCoordinateSystem: Decimal; decimalLatitude: 10.928; decimalLongitude: -85.467; **Identification:** identifiedBy: AJ Fleming; dateIdentified: 2014; **Event:** samplingProtocol: reared from caterpillar of *Talides sinois* (Hesperiidae); verbatimEventDate: 19-Aug-2002; **Record Level:** language: en; institutionCode: CNC; collectionCode: Insects; basisOfRecord: Pinned Specimen
- **Type status:** Paratype. **Occurrence:** occurrenceDetails: http://janzen.sas.upenn.edu; catalogNumber: DHJPAR0022901; recordedBy: D.H. Janzen & W. Hallwachs; individualID: DHJPAR0022901; individualCount: 1; lifeStage: adult; preparations: pinned; otherCatalogNumbers: 02-SRNP-28992; **Taxon:** scientificName: Spathidexia marioburgosi; phylum: Arthropoda; class: Insecta; order: Diptera; family: Tachinidae; genus: Spathidexia; specificEpithet: marioburgosi; scientificNameAuthorship: Fleming & Wood, 2015; **Location:** continent: Central America; country: Costa Rica; countryCode: CR; stateProvince: Guanacaste; county: Area de Conservacion Guanacaste; locality: Sector Del Oro; verbatimLocality: Guacimos; verbatimElevation: 380; verbatimLatitude: 11.015; verbatimLongitude: -85.475; verbatimCoordinateSystem: Decimal; decimalLatitude: 11.015; decimalLongitude: -85.475; **Identification:** identifiedBy: AJ Fleming; dateIdentified: 2014; **Event:** samplingProtocol: reared from caterpillar of *Talides sergestus* (Hesperiidae); verbatimEventDate: 16-Sep-2002; **Record Level:** language: en; institutionCode: CNC; collectionCode: Insects; basisOfRecord: Pinned Specimen
- **Type status:** Paratype. **Occurrence:** occurrenceDetails: http://janzen.sas.upenn.edu; catalogNumber: DHJPAR0022902; recordedBy: D.H. Janzen & W. Hallwachs; individualID: DHJPAR0022902; individualCount: 1; lifeStage: adult; preparations: pinned; otherCatalogNumbers: 02-SRNP-33351; **Taxon:** scientificName: Spathidexia marioburgosi; phylum: Arthropoda; class: Insecta; order: Diptera; family: Tachinidae; genus: Spathidexia; specificEpithet: marioburgosi; scientificNameAuthorship: Fleming & Wood, 2015; **Location:** continent: Central America; country: Costa Rica; countryCode: CR; stateProvince: Guanacaste; county: Area de Conservacion Guanacaste; locality: Sector El Hacha; verbatimLocality: Finca Araya; verbatimElevation: 295; verbatimLatitude: 11.015; verbatimLongitude: -85.511; verbatimCoordinateSystem: Decimal; decimalLatitude: 11.015; decimalLongitude: -85.511; **Identification:** identifiedBy: AJ Fleming; dateIdentified: 2014; **Event:** samplingProtocol: reared from caterpillar of *Talides sinois* (Hesperiidae); verbatimEventDate: 09-Dec-2002; **Record Level:** language: en; institutionCode: CNC; collectionCode: Insects; basisOfRecord: Pinned Specimen
- **Type status:** Paratype. **Occurrence:** occurrenceDetails: http://janzen.sas.upenn.edu; catalogNumber: DHJPAR0022903; recordedBy: D.H. Janzen & W. Hallwachs; individualID: DHJPAR0022903; individualCount: 1; lifeStage: adult; preparations: pinned; otherCatalogNumbers: 02-SRNP-28345; **Taxon:** scientificName: Spathidexia marioburgosi; phylum: Arthropoda; class: Insecta; order: Diptera; family: Tachinidae; genus: Spathidexia; specificEpithet: marioburgosi; scientificNameAuthorship: Fleming & Wood, 2015; **Location:** continent: Central America; country: Costa Rica; countryCode: CR; stateProvince: Guanacaste; county: Area de Conservacion Guanacaste; locality: Sector Del Oro; verbatimLocality: Camino Mangos; verbatimElevation: 480; verbatimLatitude: 11.008; verbatimLongitude: -85.479; verbatimCoordinateSystem: Decimal; decimalLatitude: 11.008; decimalLongitude: -85.479; **Identification:** identifiedBy: AJ Fleming; dateIdentified: 2014; **Event:** samplingProtocol: reared from caterpillar of *Talides* Burns04 (Hesperiidae); verbatimEventDate: 08-Sep-2002; **Record Level:** language: en; institutionCode: CNC; collectionCode: Insects; basisOfRecord: Pinned Specimen
- **Type status:** Paratype. **Occurrence:** occurrenceDetails: http://janzen.sas.upenn.edu; catalogNumber: DHJPAR0022904; recordedBy: D.H. Janzen & W. Hallwachs; individualID: DHJPAR0022904; individualCount: 1; lifeStage: adult; preparations: pinned; otherCatalogNumbers: 04-SRNP-47949; **Taxon:** scientificName: Spathidexia marioburgosi; phylum: Arthropoda; class: Insecta; order: Diptera; family: Tachinidae; genus: Spathidexia; specificEpithet: marioburgosi; scientificNameAuthorship: Fleming & Wood, 2015; **Location:** continent: Central America; country: Costa Rica; countryCode: CR; stateProvince: Guanacaste; county: Area de Conservacion Guanacaste; locality: Sector Cacao; verbatimLocality: Cuesta Caimito; verbatimElevation: 640; verbatimLatitude: 10.891; verbatimLongitude: -85.472; verbatimCoordinateSystem: Decimal; decimalLatitude: 10.891; decimalLongitude: -85.472; **Identification:** identifiedBy: AJ Fleming; dateIdentified: 2014; **Event:** samplingProtocol: reared from caterpillar of *Talides sinois* (Hesperiidae); verbatimEventDate: 13-Sep-2004; **Record Level:** language: en; institutionCode: CNC; collectionCode: Insects; basisOfRecord: Pinned Specimen
- **Type status:** Paratype. **Occurrence:** occurrenceDetails: http://janzen.sas.upenn.edu; catalogNumber: DHJPAR0022905; recordedBy: D.H. Janzen & W. Hallwachs; individualID: DHJPAR0022905; individualCount: 1; lifeStage: adult; preparations: pinned; otherCatalogNumbers: 02-SRNP-27830; **Taxon:** scientificName: Spathidexia marioburgosi; phylum: Arthropoda; class: Insecta; order: Diptera; family: Tachinidae; genus: Spathidexia; specificEpithet: marioburgosi; scientificNameAuthorship: Fleming & Wood, 2015; **Location:** continent: Central America; country: Costa Rica; countryCode: CR; stateProvince: Guanacaste; county: Area de Conservacion Guanacaste; locality: Sector Del Oro; verbatimLocality: Camino Mangos; verbatimElevation: 480; verbatimLatitude: 11.008; verbatimLongitude: -85.479; verbatimCoordinateSystem: Decimal; decimalLatitude: 11.008; decimalLongitude: -85.479; **Identification:** identifiedBy: AJ Fleming; dateIdentified: 2014; **Event:** samplingProtocol: reared from caterpillar of *Talides sergestus* (Hesperiidae); verbatimEventDate: 05-Sep-2002; **Record Level:** language: en; institutionCode: CNC; collectionCode: Insects; basisOfRecord: Pinned Specimen
- **Type status:** Paratype. **Occurrence:** occurrenceDetails: http://janzen.sas.upenn.edu; catalogNumber: DHJPAR0022906; recordedBy: D.H. Janzen & W. Hallwachs; individualID: DHJPAR0022906; individualCount: 1; lifeStage: adult; preparations: pinned; otherCatalogNumbers: 02-SRNP-27838; **Taxon:** scientificName: Spathidexia marioburgosi; phylum: Arthropoda; class: Insecta; order: Diptera; family: Tachinidae; genus: Spathidexia; specificEpithet: marioburgosi; scientificNameAuthorship: Fleming & Wood, 2015; **Location:** continent: Central America; country: Costa Rica; countryCode: CR; stateProvince: Guanacaste; county: Area de Conservacion Guanacaste; locality: Sector Del Oro; verbatimLocality: Camino Mangos; verbatimElevation: 480; verbatimLatitude: 11.008; verbatimLongitude: -85.479; verbatimCoordinateSystem: Decimal; decimalLatitude: 11.008; decimalLongitude: -85.479; **Identification:** identifiedBy: AJ Fleming; dateIdentified: 2014; **Event:** samplingProtocol: reared from caterpillar of *Talides sinois* (Hesperiidae); verbatimEventDate: 02-Sep-2002; **Record Level:** language: en; institutionCode: CNC; collectionCode: Insects; basisOfRecord: Pinned Specimen
- **Type status:** Paratype. **Occurrence:** occurrenceDetails: http://janzen.sas.upenn.edu; catalogNumber: DHJPAR0022907; recordedBy: D.H. Janzen & W. Hallwachs; individualID: DHJPAR0022907; individualCount: 1; lifeStage: adult; preparations: pinned; otherCatalogNumbers: 03-SRNP-34815; **Taxon:** scientificName: Spathidexia marioburgosi; phylum: Arthropoda; class: Insecta; order: Diptera; family: Tachinidae; genus: Spathidexia; specificEpithet: marioburgosi; scientificNameAuthorship: Fleming & Wood, 2015; **Location:** continent: Central America; country: Costa Rica; countryCode: CR; stateProvince: Alajuela; county: Area de Conservacion Guanacaste; locality: Sector San Cristobal; verbatimLocality: Sendero Corredor; verbatimElevation: 620; verbatimLatitude: 10.879; verbatimLongitude: -85.39; verbatimCoordinateSystem: Decimal; decimalLatitude: 10.879; decimalLongitude: -85.39; **Identification:** identifiedBy: AJ Fleming; dateIdentified: 2014; **Event:** samplingProtocol: reared from caterpillar of *Talides sinois* (Hesperiidae); verbatimEventDate: 21-Jan-2004; **Record Level:** language: en; institutionCode: CNC; collectionCode: Insects; basisOfRecord: Pinned Specimen
- **Type status:** Paratype. **Occurrence:** occurrenceDetails: http://janzen.sas.upenn.edu; catalogNumber: DHJPAR0022908; recordedBy: D.H. Janzen & W. Hallwachs; individualID: DHJPAR0022908; individualCount: 1; lifeStage: adult; preparations: pinned; otherCatalogNumbers: 03-SRNP-29255; **Taxon:** scientificName: Spathidexia marioburgosi; phylum: Arthropoda; class: Insecta; order: Diptera; family: Tachinidae; genus: Spathidexia; specificEpithet: marioburgosi; scientificNameAuthorship: Fleming & Wood, 2015; **Location:** continent: Central America; country: Costa Rica; countryCode: CR; stateProvince: Guanacaste; county: Area de Conservacion Guanacaste; locality: Sector Del Oro; verbatimLocality: Sendero Puertas; verbatimElevation: 400; verbatimLatitude: 11.011; verbatimLongitude: -85.488; verbatimCoordinateSystem: Decimal; decimalLatitude: 11.011; decimalLongitude: -85.488; **Identification:** identifiedBy: AJ Fleming; dateIdentified: 2014; **Event:** samplingProtocol: reared from caterpillar of *Talides sinois* (Hesperiidae); verbatimEventDate: 06-Nov-2003; **Record Level:** language: en; institutionCode: CNC; collectionCode: Insects; basisOfRecord: Pinned Specimen
- **Type status:** Paratype. **Occurrence:** occurrenceDetails: http://janzen.sas.upenn.edu; catalogNumber: DHJPAR0022909; recordedBy: D.H. Janzen & W. Hallwachs; individualID: DHJPAR0022909; individualCount: 1; lifeStage: adult; preparations: pinned; otherCatalogNumbers: 05-SRNP-47225; **Taxon:** scientificName: Spathidexia marioburgosi; phylum: Arthropoda; class: Insecta; order: Diptera; family: Tachinidae; genus: Spathidexia; specificEpithet: marioburgosi; scientificNameAuthorship: Fleming & Wood, 2015; **Location:** continent: Central America; country: Costa Rica; countryCode: CR; stateProvince: Guanacaste; county: Area de Conservacion Guanacaste; locality: Sector Cacao; verbatimLocality: Puente Gongora; verbatimElevation: 540; verbatimLatitude: 10.885; verbatimLongitude: -85.472; verbatimCoordinateSystem: Decimal; decimalLatitude: 10.885; decimalLongitude: -85.472; **Identification:** identifiedBy: AJ Fleming; dateIdentified: 2014; **Event:** samplingProtocol: reared from caterpillar of *Talides sinois* (Hesperiidae); verbatimEventDate: 11-Sep-2005; **Record Level:** language: en; institutionCode: CNC; collectionCode: Insects; basisOfRecord: Pinned Specimen
- **Type status:** Paratype. **Occurrence:** occurrenceDetails: http://janzen.sas.upenn.edu; catalogNumber: DHJPAR0022910; recordedBy: D.H. Janzen & W. Hallwachs; individualID: DHJPAR0022910; individualCount: 1; lifeStage: adult; preparations: pinned; otherCatalogNumbers: 05-SRNP-47226; **Taxon:** scientificName: Spathidexia marioburgosi; phylum: Arthropoda; class: Insecta; order: Diptera; family: Tachinidae; genus: Spathidexia; specificEpithet: marioburgosi; scientificNameAuthorship: Fleming & Wood, 2015; **Location:** continent: Central America; country: Costa Rica; countryCode: CR; stateProvince: Guanacaste; county: Area de Conservacion Guanacaste; locality: Sector Cacao; verbatimLocality: Puente Gongora; verbatimElevation: 540; verbatimLatitude: 10.885; verbatimLongitude: -85.472; verbatimCoordinateSystem: Decimal; decimalLatitude: 10.885; decimalLongitude: -85.472; **Identification:** identifiedBy: AJ Fleming; dateIdentified: 2014; **Event:** samplingProtocol: reared from caterpillar of *Talides sergestus* (Hesperiidae); verbatimEventDate: 18-Sep-2005; **Record Level:** language: en; institutionCode: CNC; collectionCode: Insects; basisOfRecord: Pinned Specimen
- **Type status:** Paratype. **Occurrence:** occurrenceDetails: http://janzen.sas.upenn.edu; catalogNumber: DHJPAR0022911; recordedBy: D.H. Janzen & W. Hallwachs; individualID: DHJPAR0022911; individualCount: 1; lifeStage: adult; preparations: pinned; otherCatalogNumbers: 05-SRNP-47373; **Taxon:** scientificName: Spathidexia marioburgosi; phylum: Arthropoda; class: Insecta; order: Diptera; family: Tachinidae; genus: Spathidexia; specificEpithet: marioburgosi; scientificNameAuthorship: Fleming & Wood, 2015; **Location:** continent: Central America; country: Costa Rica; countryCode: CR; stateProvince: Guanacaste; county: Area de Conservacion Guanacaste; locality: Sector Cacao; verbatimLocality: Puente Gongora; verbatimElevation: 540; verbatimLatitude: 10.885; verbatimLongitude: -85.472; verbatimCoordinateSystem: Decimal; decimalLatitude: 10.885; decimalLongitude: -85.472; **Identification:** identifiedBy: AJ Fleming; dateIdentified: 2014; **Event:** samplingProtocol: reared from caterpillar of *Talides sinois* (Hesperiidae); verbatimEventDate: 12-Sep-2005; **Record Level:** language: en; institutionCode: CNC; collectionCode: Insects; basisOfRecord: Pinned Specimen
- **Type status:** Paratype. **Occurrence:** occurrenceDetails: http://janzen.sas.upenn.edu; catalogNumber: DHJPAR0022912; recordedBy: D.H. Janzen & W. Hallwachs; individualID: DHJPAR0022912; individualCount: 1; lifeStage: adult; preparations: pinned; otherCatalogNumbers: 05-SRNP-4221; **Taxon:** scientificName: Spathidexia marioburgosi; phylum: Arthropoda; class: Insecta; order: Diptera; family: Tachinidae; genus: Spathidexia; specificEpithet: marioburgosi; scientificNameAuthorship: Fleming & Wood, 2015; **Location:** continent: Central America; country: Costa Rica; countryCode: CR; stateProvince: Alajuela; county: Area de Conservacion Guanacaste; locality: Sector San Cristobal; verbatimLocality: Quebrada Cementerio; verbatimElevation: 700; verbatimLatitude: 10.871; verbatimLongitude: -85.387; verbatimCoordinateSystem: Decimal; decimalLatitude: 10.871; decimalLongitude: -85.387; **Identification:** identifiedBy: AJ Fleming; dateIdentified: 2014; **Event:** samplingProtocol: reared from caterpillar of *Talides sinois* (Hesperiidae); verbatimEventDate: 01-Sep-2005; **Record Level:** language: en; institutionCode: CNC; collectionCode: Insects; basisOfRecord: Pinned Specimen
- **Type status:** Paratype. **Occurrence:** occurrenceDetails: http://janzen.sas.upenn.edu; catalogNumber: DHJPAR0022913; recordedBy: D.H. Janzen & W. Hallwachs; individualID: DHJPAR0022913; individualCount: 1; lifeStage: adult; preparations: pinned; otherCatalogNumbers: 05-SRNP-47041; **Taxon:** scientificName: Spathidexia marioburgosi; phylum: Arthropoda; class: Insecta; order: Diptera; family: Tachinidae; genus: Spathidexia; specificEpithet: marioburgosi; scientificNameAuthorship: Fleming & Wood, 2015; **Location:** continent: Central America; country: Costa Rica; countryCode: CR; stateProvince: Guanacaste; county: Area de Conservacion Guanacaste; locality: Sector Cacao; verbatimLocality: Sendero Palmas; verbatimElevation: 675; verbatimLatitude: 10.896; verbatimLongitude: -85.474; verbatimCoordinateSystem: Decimal; decimalLatitude: 10.896; decimalLongitude: -85.474; **Identification:** identifiedBy: AJ Fleming; dateIdentified: 2014; **Event:** samplingProtocol: reared from caterpillar of *Talides sergestus* (Hesperiidae); verbatimEventDate: 29-Aug-2005; **Record Level:** language: en; institutionCode: CNC; collectionCode: Insects; basisOfRecord: Pinned Specimen
- **Type status:** Paratype. **Occurrence:** occurrenceDetails: http://janzen.sas.upenn.edu; catalogNumber: DHJPAR0022914; recordedBy: D.H. Janzen & W. Hallwachs; individualID: DHJPAR0022914; individualCount: 1; lifeStage: adult; preparations: pinned; otherCatalogNumbers: 05-SRNP-47477; **Taxon:** scientificName: Spathidexia marioburgosi; phylum: Arthropoda; class: Insecta; order: Diptera; family: Tachinidae; genus: Spathidexia; specificEpithet: marioburgosi; scientificNameAuthorship: Fleming & Wood, 2015; **Location:** continent: Central America; country: Costa Rica; countryCode: CR; stateProvince: Guanacaste; county: Area de Conservacion Guanacaste; locality: Sector Cacao; verbatimLocality: Puente Gongora; verbatimElevation: 540; verbatimLatitude: 10.885; verbatimLongitude: -85.472; verbatimCoordinateSystem: Decimal; decimalLatitude: 10.885; decimalLongitude: -85.472; **Identification:** identifiedBy: AJ Fleming; dateIdentified: 2014; **Event:** samplingProtocol: reared from caterpillar of *Talides sinois* (Hesperiidae); verbatimEventDate: 21-Sep-2005; **Record Level:** language: en; institutionCode: CNC; collectionCode: Insects; basisOfRecord: Pinned Specimen
- **Type status:** Paratype. **Occurrence:** occurrenceDetails: http://janzen.sas.upenn.edu; catalogNumber: DHJPAR0022915; recordedBy: D.H. Janzen & W. Hallwachs; individualID: DHJPAR0022915; individualCount: 1; lifeStage: adult; preparations: pinned; otherCatalogNumbers: 05-SRNP-47479; **Taxon:** scientificName: Spathidexia marioburgosi; phylum: Arthropoda; class: Insecta; order: Diptera; family: Tachinidae; genus: Spathidexia; specificEpithet: marioburgosi; scientificNameAuthorship: Fleming & Wood, 2015; **Location:** continent: Central America; country: Costa Rica; countryCode: CR; stateProvince: Guanacaste; county: Area de Conservacion Guanacaste; locality: Sector Cacao; verbatimLocality: Puente Gongora; verbatimElevation: 540; verbatimLatitude: 10.885; verbatimLongitude: -85.472; verbatimCoordinateSystem: Decimal; decimalLatitude: 10.885; decimalLongitude: -85.472; **Identification:** identifiedBy: AJ Fleming; dateIdentified: 2014; **Event:** samplingProtocol: reared from caterpillar of *Talides sinois* (Hesperiidae); verbatimEventDate: 21-Sep-2005; **Record Level:** language: en; institutionCode: CNC; collectionCode: Insects; basisOfRecord: Pinned Specimen
- **Type status:** Paratype. **Occurrence:** occurrenceDetails: http://janzen.sas.upenn.edu; catalogNumber: DHJPAR0022916; recordedBy: D.H. Janzen & W. Hallwachs; individualID: DHJPAR0022916; individualCount: 1; lifeStage: adult; preparations: pinned; otherCatalogNumbers: 03-SRNP-11367; **Taxon:** scientificName: Spathidexia marioburgosi; phylum: Arthropoda; class: Insecta; order: Diptera; family: Tachinidae; genus: Spathidexia; specificEpithet: marioburgosi; scientificNameAuthorship: Fleming & Wood, 2015; **Location:** continent: Central America; country: Costa Rica; countryCode: CR; stateProvince: Alajuela; county: Area de Conservacion Guanacaste; locality: Sector Rincon Rain Forest; verbatimLocality: Sendero Platanal; verbatimElevation: 335; verbatimLatitude: 10.849; verbatimLongitude: -85.253; verbatimCoordinateSystem: Decimal; decimalLatitude: 10.849; decimalLongitude: -85.253; **Identification:** identifiedBy: AJ Fleming; dateIdentified: 2014; **Event:** samplingProtocol: reared from caterpillar of *Talides sinois* (Hesperiidae); verbatimEventDate: 16-Jul-2003; **Record Level:** language: en; institutionCode: CNC; collectionCode: Insects; basisOfRecord: Pinned Specimen
- **Type status:** Paratype. **Occurrence:** occurrenceDetails: http://janzen.sas.upenn.edu; catalogNumber: DHJPAR0022917; recordedBy: D.H. Janzen & W. Hallwachs; individualID: DHJPAR0022917; individualCount: 1; lifeStage: adult; preparations: pinned; otherCatalogNumbers: 02-SRNP-19519; **Taxon:** scientificName: Spathidexia marioburgosi; phylum: Arthropoda; class: Insecta; order: Diptera; family: Tachinidae; genus: Spathidexia; specificEpithet: marioburgosi; scientificNameAuthorship: Fleming & Wood, 2015; **Location:** continent: Central America; country: Costa Rica; countryCode: CR; stateProvince: Alajuela; county: Area de Conservacion Guanacaste; locality: Sector San Cristobal; verbatimLocality: Sendero Corredor; verbatimElevation: 620; verbatimLatitude: 10.879; verbatimLongitude: -85.39; verbatimCoordinateSystem: Decimal; decimalLatitude: 10.879; decimalLongitude: -85.39; **Identification:** identifiedBy: AJ Fleming; dateIdentified: 2014; **Event:** samplingProtocol: reared from caterpillar of *Talides sergestus* (Hesperiidae); verbatimEventDate: 30-Oct-2002; **Record Level:** language: en; institutionCode: CNC; collectionCode: Insects; basisOfRecord: Pinned Specimen
- **Type status:** Paratype. **Occurrence:** occurrenceDetails: http://janzen.sas.upenn.edu; catalogNumber: DHJPAR0022918; recordedBy: D.H. Janzen & W. Hallwachs; individualID: DHJPAR0022918; individualCount: 1; lifeStage: adult; preparations: pinned; otherCatalogNumbers: 02-SRNP-29143; **Taxon:** scientificName: Spathidexia marioburgosi; phylum: Arthropoda; class: Insecta; order: Diptera; family: Tachinidae; genus: Spathidexia; specificEpithet: marioburgosi; scientificNameAuthorship: Fleming & Wood, 2015; **Location:** continent: Central America; country: Costa Rica; countryCode: CR; stateProvince: Guanacaste; county: Area de Conservacion Guanacaste; locality: Sector Del Oro; verbatimLocality: Camino Mangos; verbatimElevation: 480; verbatimLatitude: 11.008; verbatimLongitude: -85.479; verbatimCoordinateSystem: Decimal; decimalLatitude: 11.008; decimalLongitude: -85.479; **Identification:** identifiedBy: AJ Fleming; dateIdentified: 2014; **Event:** samplingProtocol: reared from caterpillar of *Talides sinois* (Hesperiidae); verbatimEventDate: 16-Sep-2002; **Record Level:** language: en; institutionCode: CNC; collectionCode: Insects; basisOfRecord: Pinned Specimen
- **Type status:** Paratype. **Occurrence:** occurrenceDetails: http://janzen.sas.upenn.edu; catalogNumber: DHJPAR0022919; recordedBy: D.H. Janzen & W. Hallwachs; individualID: DHJPAR0022919; individualCount: 1; lifeStage: adult; preparations: pinned; otherCatalogNumbers: 02-SRNP-28992; **Taxon:** scientificName: Spathidexia marioburgosi; phylum: Arthropoda; class: Insecta; order: Diptera; family: Tachinidae; genus: Spathidexia; specificEpithet: marioburgosi; scientificNameAuthorship: Fleming & Wood, 2015; **Location:** continent: Central America; country: Costa Rica; countryCode: CR; stateProvince: Guanacaste; county: Area de Conservacion Guanacaste; locality: Sector Del Oro; verbatimLocality: Guacimos; verbatimElevation: 380; verbatimLatitude: 11.015; verbatimLongitude: -85.475; verbatimCoordinateSystem: Decimal; decimalLatitude: 11.015; decimalLongitude: -85.475; **Identification:** identifiedBy: AJ Fleming; dateIdentified: 2014; **Event:** samplingProtocol: reared from caterpillar of *Talides* Burns04 (Hesperiidae); verbatimEventDate: 16-Sep-2002; **Record Level:** language: en; institutionCode: CNC; collectionCode: Insects; basisOfRecord: Pinned Specimen
- **Type status:** Paratype. **Occurrence:** occurrenceDetails: http://janzen.sas.upenn.edu; catalogNumber: DHJPAR0022920; recordedBy: D.H. Janzen & W. Hallwachs; individualID: DHJPAR0022920; individualCount: 1; lifeStage: adult; preparations: pinned; otherCatalogNumbers: 02-SRNP-9935; **Taxon:** scientificName: Spathidexia marioburgosi; phylum: Arthropoda; class: Insecta; order: Diptera; family: Tachinidae; genus: Spathidexia; specificEpithet: marioburgosi; scientificNameAuthorship: Fleming & Wood, 2015; **Location:** continent: Central America; country: Costa Rica; countryCode: CR; stateProvince: Guanacaste; county: Area de Conservacion Guanacaste; locality: Sector Cacao; verbatimLocality: Sendero Toma Agua; verbatimElevation: 1140; verbatimLatitude: 10.928; verbatimLongitude: -85.467; verbatimCoordinateSystem: Decimal; decimalLatitude: 10.928; decimalLongitude: -85.467; **Identification:** identifiedBy: AJ Fleming; dateIdentified: 2014; **Event:** samplingProtocol: reared from caterpillar of *Talides* Burns04 (Hesperiidae); verbatimEventDate: 24-Aug-2002; **Record Level:** language: en; institutionCode: CNC; collectionCode: Insects; basisOfRecord: Pinned Specimen
- **Type status:** Paratype. **Occurrence:** occurrenceDetails: http://janzen.sas.upenn.edu; catalogNumber: DHJPAR0022921; recordedBy: D.H. Janzen & W. Hallwachs; individualID: DHJPAR0022921; individualCount: 1; lifeStage: adult; preparations: pinned; otherCatalogNumbers: 02-SRNP-9933; **Taxon:** scientificName: Spathidexia marioburgosi; phylum: Arthropoda; class: Insecta; order: Diptera; family: Tachinidae; genus: Spathidexia; specificEpithet: marioburgosi; scientificNameAuthorship: Fleming & Wood, 2015; **Location:** continent: Central America; country: Costa Rica; countryCode: CR; stateProvince: Guanacaste; county: Area de Conservacion Guanacaste; locality: Sector Cacao; verbatimLocality: Sendero Toma Agua; verbatimElevation: 1140; verbatimLatitude: 10.928; verbatimLongitude: -85.467; verbatimCoordinateSystem: Decimal; decimalLatitude: 10.928; decimalLongitude: -85.467; **Identification:** identifiedBy: AJ Fleming; dateIdentified: 2014; **Event:** samplingProtocol: reared from caterpillar of *Talides sinois* (Hesperiidae); verbatimEventDate: 17-Aug-2002; **Record Level:** language: en; institutionCode: CNC; collectionCode: Insects; basisOfRecord: Pinned Specimen
- **Type status:** Paratype. **Occurrence:** occurrenceDetails: http://janzen.sas.upenn.edu; catalogNumber: DHJPAR0022922; recordedBy: D.H. Janzen & W. Hallwachs; individualID: DHJPAR0022922; individualCount: 1; lifeStage: adult; preparations: pinned; otherCatalogNumbers: 03-SRNP-8968; **Taxon:** scientificName: Spathidexia marioburgosi; phylum: Arthropoda; class: Insecta; order: Diptera; family: Tachinidae; genus: Spathidexia; specificEpithet: marioburgosi; scientificNameAuthorship: Fleming & Wood, 2015; **Location:** continent: Central America; country: Costa Rica; countryCode: CR; stateProvince: Alajuela; county: Area de Conservacion Guanacaste; locality: Sector San Cristobal; verbatimLocality: Rio Blanco Abajo; verbatimElevation: 500; verbatimLatitude: 10.9; verbatimLongitude: -85.373; verbatimCoordinateSystem: Decimal; decimalLatitude: 10.9; decimalLongitude: -85.373; **Identification:** identifiedBy: AJ Fleming; dateIdentified: 2014; **Event:** samplingProtocol: reared from caterpillar of *Talides sergestus* (Hesperiidae); verbatimEventDate: 04-Nov-2003; **Record Level:** language: en; institutionCode: CNC; collectionCode: Insects; basisOfRecord: Pinned Specimen
- **Type status:** Paratype. **Occurrence:** occurrenceDetails: http://janzen.sas.upenn.edu; catalogNumber: DHJPAR0022923; recordedBy: D.H. Janzen & W. Hallwachs; individualID: DHJPAR0022923; individualCount: 1; lifeStage: adult; preparations: pinned; otherCatalogNumbers: 03-SRNP-23786; **Taxon:** scientificName: Spathidexia marioburgosi; phylum: Arthropoda; class: Insecta; order: Diptera; family: Tachinidae; genus: Spathidexia; specificEpithet: marioburgosi; scientificNameAuthorship: Fleming & Wood, 2015; **Location:** continent: Central America; country: Costa Rica; countryCode: CR; stateProvince: Guanacaste; county: Area de Conservacion Guanacaste; locality: Sector Cacao; verbatimLocality: Puente Gongora; verbatimElevation: 540; verbatimLatitude: 10.885; verbatimLongitude: -85.472; verbatimCoordinateSystem: Decimal; decimalLatitude: 10.885; decimalLongitude: -85.472; **Identification:** identifiedBy: AJ Fleming; dateIdentified: 2014; **Event:** samplingProtocol: reared from caterpillar of *Talides* Burns04 (Hesperiidae); verbatimEventDate: 30-Nov-2003; **Record Level:** language: en; institutionCode: CNC; collectionCode: Insects; basisOfRecord: Pinned Specimen
- **Type status:** Paratype. **Occurrence:** occurrenceDetails: http://janzen.sas.upenn.edu; catalogNumber: DHJPAR0022924; recordedBy: D.H. Janzen & W. Hallwachs; individualID: DHJPAR0022924; individualCount: 1; lifeStage: adult; preparations: pinned; otherCatalogNumbers: 04-SRNP-48016; **Taxon:** scientificName: Spathidexia marioburgosi; phylum: Arthropoda; class: Insecta; order: Diptera; family: Tachinidae; genus: Spathidexia; specificEpithet: marioburgosi; scientificNameAuthorship: Fleming & Wood, 2015; **Location:** continent: Central America; country: Costa Rica; countryCode: CR; stateProvince: Guanacaste; county: Area de Conservacion Guanacaste; locality: Sector Cacao; verbatimLocality: Quebrada Otilio; verbatimElevation: 550; verbatimLatitude: 10.89; verbatimLongitude: -85.48; verbatimCoordinateSystem: Decimal; decimalLatitude: 10.89; decimalLongitude: -85.48; **Identification:** identifiedBy: AJ Fleming; dateIdentified: 2014; **Event:** samplingProtocol: reared from caterpillar of *Talides sinois* (Hesperiidae); verbatimEventDate: 06-Sep-2004; **Record Level:** language: en; institutionCode: CNC; collectionCode: Insects; basisOfRecord: Pinned Specimen
- **Type status:** Paratype. **Occurrence:** occurrenceDetails: http://janzen.sas.upenn.edu; catalogNumber: DHJPAR0022925; recordedBy: D.H. Janzen & W. Hallwachs; individualID: DHJPAR0022925; individualCount: 1; lifeStage: adult; preparations: pinned; otherCatalogNumbers: 01-SRNP-22877; **Taxon:** scientificName: Spathidexia marioburgosi; phylum: Arthropoda; class: Insecta; order: Diptera; family: Tachinidae; genus: Spathidexia; specificEpithet: marioburgosi; scientificNameAuthorship: Fleming & Wood, 2015; **Location:** continent: Central America; country: Costa Rica; countryCode: CR; stateProvince: Alajuela; county: Area de Conservacion Guanacaste; locality: Sector San Cristobal; verbatimLocality: Sendero Corredor; verbatimElevation: 620; verbatimLatitude: 10.879; verbatimLongitude: -85.39; verbatimCoordinateSystem: Decimal; decimalLatitude: 10.879; decimalLongitude: -85.39; **Identification:** identifiedBy: AJ Fleming; dateIdentified: 2014; **Event:** samplingProtocol: reared from caterpillar of *Talides sinois* (Hesperiidae); verbatimEventDate: 31-Dec-2001; **Record Level:** language: en; institutionCode: CNC; collectionCode: Insects; basisOfRecord: Pinned Specimen
- **Type status:** Paratype. **Occurrence:** occurrenceDetails: http://janzen.sas.upenn.edu; catalogNumber: DHJPAR0022926; recordedBy: D.H. Janzen & W. Hallwachs; individualID: DHJPAR0022926; individualCount: 1; lifeStage: adult; preparations: pinned; otherCatalogNumbers: 02-SRNP-27700; **Taxon:** scientificName: *Spathidexia* marioburgosi; phylum: Arthropoda; class: Insecta; order: Diptera; family: Tachinidae; genus: Spathidexia; specificEpithet: marioburgosi; scientificNameAuthorship: Fleming & Wood, 2015; **Location:** continent: Central America; country: Costa Rica; countryCode: CR; stateProvince: Guanacaste; county: Area de Conservacion Guanacaste; locality: Sector Del Oro; verbatimLocality: Guacimos; verbatimElevation: 380; verbatimLatitude: 11.015; verbatimLongitude: -85.475; verbatimCoordinateSystem: Decimal; decimalLatitude: 11.015; decimalLongitude: -85.475; **Identification:** identifiedBy: AJ Fleming; dateIdentified: 2014; **Event:** samplingProtocol: reared from caterpillar of *Talides sinois* (Hesperiidae); verbatimEventDate: 04-Sep-2002; **Record Level:** language: en; institutionCode: CNC; collectionCode: Insects; basisOfRecord: Pinned Specimen
- **Type status:** Paratype. **Occurrence:** occurrenceDetails: http://janzen.sas.upenn.edu; catalogNumber: DHJPAR0022927; recordedBy: D.H. Janzen & W. Hallwachs; individualID: DHJPAR0022927; individualCount: 1; lifeStage: adult; preparations: pinned; otherCatalogNumbers: 02-SRNP-2319; **Taxon:** scientificName: Spathidexia marioburgosi; phylum: Arthropoda; class: Insecta; order: Diptera; family: Tachinidae; genus: Spathidexia; specificEpithet: marioburgosi; scientificNameAuthorship: Fleming & Wood, 2015; **Location:** continent: Central America; country: Costa Rica; countryCode: CR; stateProvince: Alajuela; county: Area de Conservacion Guanacaste; locality: Sector San Cristobal; verbatimLocality: Rio Blanco Abajo; verbatimElevation: 500; verbatimLatitude: 10.9; verbatimLongitude: -85.373; verbatimCoordinateSystem: Decimal; decimalLatitude: 10.9; decimalLongitude: -85.373; **Identification:** identifiedBy: AJ Fleming; dateIdentified: 2014; **Event:** samplingProtocol: reared from caterpillar of *Talides sinois* (Hesperiidae); verbatimEventDate: 24-Apr-2002; **Record Level:** language: en; institutionCode: CNC; collectionCode: Insects; basisOfRecord: Pinned Specimen
- **Type status:** Paratype. **Occurrence:** occurrenceDetails: http://janzen.sas.upenn.edu; catalogNumber: DHJPAR0022928; recordedBy: D.H. Janzen & W. Hallwachs; individualID: DHJPAR0022928; individualCount: 1; lifeStage: adult; preparations: pinned; otherCatalogNumbers: 03-SRNP-5624; **Taxon:** scientificName: Spathidexia marioburgosi; phylum: Arthropoda; class: Insecta; order: Diptera; family: Tachinidae; genus: Spathidexia; specificEpithet: marioburgosi; scientificNameAuthorship: Fleming & Wood, 2015; **Location:** continent: Central America; country: Costa Rica; countryCode: CR; stateProvince: Alajuela; county: Area de Conservacion Guanacaste; locality: Sector San Cristobal; verbatimLocality: Rio Blanco Abajo; verbatimElevation: 500; verbatimLatitude: 10.9; verbatimLongitude: -85.373; verbatimCoordinateSystem: Decimal; decimalLatitude: 10.9; decimalLongitude: -85.373; **Identification:** identifiedBy: AJ Fleming; dateIdentified: 2014; **Event:** samplingProtocol: reared from caterpillar of *Talides sergestus* (Hesperiidae); verbatimEventDate: 20-Mar-2003; **Record Level:** language: en; institutionCode: CNC; collectionCode: Insects; basisOfRecord: Pinned Specimen
- **Type status:** Paratype. **Occurrence:** occurrenceDetails: http://janzen.sas.upenn.edu; catalogNumber: DHJPAR0022929; recordedBy: D.H. Janzen & W. Hallwachs; individualID: DHJPAR0022929; individualCount: 1; lifeStage: adult; preparations: pinned; otherCatalogNumbers: 04-SRNP-42789; **Taxon:** scientificName: Spathidexia marioburgosi; phylum: Arthropoda; class: Insecta; order: Diptera; family: Tachinidae; genus: Spathidexia; specificEpithet: marioburgosi; scientificNameAuthorship: Fleming & Wood, 2015; **Location:** continent: Central America; country: Costa Rica; countryCode: CR; stateProvince: Alajuela; county: Area de Conservacion Guanacaste; locality: Sector Rincon Rain Forest; verbatimLocality: Camino Rio Francia; verbatimElevation: 410; verbatimLatitude: 10.904; verbatimLongitude: -85.287; verbatimCoordinateSystem: Decimal; decimalLatitude: 10.904; decimalLongitude: -85.287; **Identification:** identifiedBy: AJ Fleming; dateIdentified: 2014; **Event:** samplingProtocol: reared from caterpillar of *Talides sinois* (Hesperiidae); verbatimEventDate: 23-Dec-2004; **Record Level:** language: en; institutionCode: CNC; collectionCode: Insects; basisOfRecord: Pinned Specimen
- **Type status:** Paratype. **Occurrence:** occurrenceDetails: http://janzen.sas.upenn.edu; catalogNumber: DHJPAR0022930; recordedBy: D.H. Janzen & W. Hallwachs; individualID: DHJPAR0022930; individualCount: 1; lifeStage: adult; preparations: pinned; otherCatalogNumbers: 05-SRNP-20280; **Taxon:** scientificName: Spathidexia marioburgosi; phylum: Arthropoda; class: Insecta; order: Diptera; family: Tachinidae; genus: Spathidexia; specificEpithet: marioburgosi; scientificNameAuthorship: Fleming & Wood, 2015; **Location:** continent: Central America; country: Costa Rica; countryCode: CR; stateProvince: Guanacaste; county: Area de Conservacion Guanacaste; locality: Sector Del Oro; verbatimLocality: Quebrada Lajosa; verbatimElevation: 400; verbatimLatitude: 11.033; verbatimLongitude: -85.429; verbatimCoordinateSystem: Decimal; decimalLatitude: 11.033; decimalLongitude: -85.429; **Identification:** identifiedBy: AJ Fleming; dateIdentified: 2014; **Event:** samplingProtocol: reared from caterpillar of *Talides sinois* (Hesperiidae); verbatimEventDate: 22-Feb-2005; **Record Level:** language: en; institutionCode: CNC; collectionCode: Insects; basisOfRecord: Pinned Specimen
- **Type status:** Paratype. **Occurrence:** occurrenceDetails: http://janzen.sas.upenn.edu; catalogNumber: DHJPAR0022931; recordedBy: D.H. Janzen & W. Hallwachs; individualID: DHJPAR0022931; individualCount: 1; lifeStage: adult; preparations: pinned; otherCatalogNumbers: 05-SRNP-46753; **Taxon:** scientificName: Spathidexia marioburgosi; phylum: Arthropoda; class: Insecta; order: Diptera; family: Tachinidae; genus: Spathidexia; specificEpithet: marioburgosi; scientificNameAuthorship: Fleming & Wood, 2015; **Location:** continent: Central America; country: Costa Rica; countryCode: CR; stateProvince: Guanacaste; county: Area de Conservacion Guanacaste; locality: Sector Cacao; verbatimLocality: Cuesta Caimito; verbatimElevation: 640; verbatimLatitude: 10.891; verbatimLongitude: -85.472; verbatimCoordinateSystem: Decimal; decimalLatitude: 10.891; decimalLongitude: -85.472; **Identification:** identifiedBy: AJ Fleming; dateIdentified: 2014; **Event:** samplingProtocol: reared from caterpillar of *Talides sinois* (Hesperiidae); verbatimEventDate: 20-Aug-2005; **Record Level:** language: en; institutionCode: CNC; collectionCode: Insects; basisOfRecord: Pinned Specimen
- **Type status:** Paratype. **Occurrence:** occurrenceDetails: http://janzen.sas.upenn.edu; catalogNumber: DHJPAR0022932; recordedBy: D.H. Janzen & W. Hallwachs; individualID: DHJPAR0022932; individualCount: 1; lifeStage: adult; preparations: pinned; otherCatalogNumbers: 05-SRNP-46479; **Taxon:** scientificName: Spathidexia marioburgosi; phylum: Arthropoda; class: Insecta; order: Diptera; family: Tachinidae; genus: Spathidexia; specificEpithet: marioburgosi; scientificNameAuthorship: Fleming & Wood, 2015; **Location:** continent: Central America; country: Costa Rica; countryCode: CR; stateProvince: Guanacaste; county: Area de Conservacion Guanacaste; locality: Sector Cacao; verbatimLocality: Estacion Gongora; verbatimElevation: 570; verbatimLatitude: 10.887; verbatimLongitude: -85.474; verbatimCoordinateSystem: Decimal; decimalLatitude: 10.887; decimalLongitude: -85.474; **Identification:** identifiedBy: AJ Fleming; dateIdentified: 2014; **Event:** samplingProtocol: reared from caterpillar of *Talides sergestus* (Hesperiidae); verbatimEventDate: 01-Aug-2005; **Record Level:** language: en; institutionCode: CNC; collectionCode: Insects; basisOfRecord: Pinned Specimen
- **Type status:** Paratype. **Occurrence:** occurrenceDetails: http://janzen.sas.upenn.edu; catalogNumber: DHJPAR0022933; recordedBy: D.H. Janzen & W. Hallwachs; individualID: DHJPAR0022933; individualCount: 1; lifeStage: adult; preparations: pinned; otherCatalogNumbers: 05-SRNP-4521; **Taxon:** scientificName: Spathidexia marioburgosi; phylum: Arthropoda; class: Insecta; order: Diptera; family: Tachinidae; genus: Spathidexia; specificEpithet: marioburgosi; scientificNameAuthorship: Fleming & Wood, 2015; **Location:** continent: Central America; country: Costa Rica; countryCode: CR; stateProvince: Alajuela; county: Area de Conservacion Guanacaste; locality: Sector San Cristobal; verbatimLocality: Sendero Huerta; verbatimElevation: 527; verbatimLatitude: 10.931; verbatimLongitude: -85.372; verbatimCoordinateSystem: Decimal; decimalLatitude: 10.931; decimalLongitude: -85.372; **Identification:** identifiedBy: AJ Fleming; dateIdentified: 2014; **Event:** samplingProtocol: reared from caterpillar of *Talides sinois* (Hesperiidae); verbatimEventDate: 28-Aug-2005; **Record Level:** language: en; institutionCode: CNC; collectionCode: Insects; basisOfRecord: Pinned Specimen
- **Type status:** Paratype. **Occurrence:** occurrenceDetails: http://janzen.sas.upenn.edu; catalogNumber: DHJPAR0022934; recordedBy: D.H. Janzen & W. Hallwachs; individualID: DHJPAR0022934; individualCount: 1; lifeStage: adult; preparations: pinned; otherCatalogNumbers: 04-SRNP-48258; **Taxon:** scientificName: Spathidexia marioburgosi; phylum: Arthropoda; class: Insecta; order: Diptera; family: Tachinidae; genus: Spathidexia; specificEpithet: marioburgosi; scientificNameAuthorship: Fleming & Wood, 2015; **Location:** continent: Central America; country: Costa Rica; countryCode: CR; stateProvince: Guanacaste; county: Area de Conservacion Guanacaste; locality: Sector Cacao; verbatimLocality: Gongora Bananal; verbatimElevation: 600; verbatimLatitude: 10.889; verbatimLongitude: -85.476; verbatimCoordinateSystem: Decimal; decimalLatitude: 10.889; decimalLongitude: -85.476; **Identification:** identifiedBy: AJ Fleming; dateIdentified: 2014; **Event:** samplingProtocol: reared from caterpillar of *Talides sergestus* (Hesperiidae); verbatimEventDate: 27-Sep-2004; **Record Level:** language: en; institutionCode: CNC; collectionCode: Insects; basisOfRecord: Pinned Specimen
- **Type status:** Paratype. **Occurrence:** occurrenceDetails: http://janzen.sas.upenn.edu; catalogNumber: DHJPAR0022935; recordedBy: D.H. Janzen & W. Hallwachs; individualID: DHJPAR0022935; individualCount: 1; lifeStage: adult; preparations: pinned; otherCatalogNumbers: 04-SRNP-47696; **Taxon:** scientificName: Spathidexia marioburgosi; phylum: Arthropoda; class: Insecta; order: Diptera; family: Tachinidae; genus: Spathidexia; specificEpithet: marioburgosi; scientificNameAuthorship: Fleming & Wood, 2015; **Location:** continent: Central America; country: Costa Rica; countryCode: CR; stateProvince: Guanacaste; county: Area de Conservacion Guanacaste; locality: Sector Cacao; verbatimLocality: Quebrada Otilio; verbatimElevation: 550; verbatimLatitude: 10.89; verbatimLongitude: -85.48; verbatimCoordinateSystem: Decimal; decimalLatitude: 10.89; decimalLongitude: -85.48; **Identification:** identifiedBy: AJ Fleming; dateIdentified: 2014; **Event:** samplingProtocol: reared from caterpillar of *Talides sergestus* (Hesperiidae); verbatimEventDate: 31-Aug-2004; **Record Level:** language: en; institutionCode: CNC; collectionCode: Insects; basisOfRecord: Pinned Specimen
- **Type status:** Paratype. **Occurrence:** occurrenceDetails: http://janzen.sas.upenn.edu; catalogNumber: DHJPAR0022936; recordedBy: D.H. Janzen & W. Hallwachs; individualID: DHJPAR0022936; individualCount: 1; lifeStage: adult; preparations: pinned; otherCatalogNumbers: 04-SRNP-60869; **Taxon:** scientificName: Spathidexia marioburgosi; phylum: Arthropoda; class: Insecta; order: Diptera; family: Tachinidae; genus: Spathidexia; specificEpithet: marioburgosi; scientificNameAuthorship: Fleming & Wood, 2015; **Location:** continent: Central America; country: Costa Rica; countryCode: CR; stateProvince: Alajuela; county: Area de Conservacion Guanacaste; locality: Sector San Cristobal; verbatimLocality: Puente Palma; verbatimElevation: 460; verbatimLatitude: 10.916; verbatimLongitude: -85.379; verbatimCoordinateSystem: Decimal; decimalLatitude: 10.916; decimalLongitude: -85.379; **Identification:** identifiedBy: AJ Fleming; dateIdentified: 2014; **Event:** samplingProtocol: reared from caterpillar of *Talides* Burns02 (Hesperiidae); verbatimEventDate: 16-Dec-2004; **Record Level:** language: en; institutionCode: CNC; collectionCode: Insects; basisOfRecord: Pinned Specimen
- **Type status:** Paratype. **Occurrence:** occurrenceDetails: http://janzen.sas.upenn.edu; catalogNumber: DHJPAR0022937; recordedBy: D.H. Janzen & W. Hallwachs; individualID: DHJPAR0022937; individualCount: 1; lifeStage: adult; preparations: pinned; otherCatalogNumbers: 04-SRNP-56649; **Taxon:** scientificName: Spathidexia marioburgosi; phylum: Arthropoda; class: Insecta; order: Diptera; family: Tachinidae; genus: Spathidexia; specificEpithet: marioburgosi; scientificNameAuthorship: Fleming & Wood, 2015; **Location:** continent: Central America; country: Costa Rica; countryCode: CR; stateProvince: Guanacaste; county: Area de Conservacion Guanacaste; locality: Sector Pitilla; verbatimLocality: Casa Roberto; verbatimElevation: 520; verbatimLatitude: 11.011; verbatimLongitude: -85.421; verbatimCoordinateSystem: Decimal; decimalLatitude: 11.011; decimalLongitude: -85.421; **Identification:** identifiedBy: AJ Fleming; dateIdentified: 2014; **Event:** samplingProtocol: reared from caterpillar of *Talides sinois* (Hesperiidae); verbatimEventDate: 06-Feb-2005; **Record Level:** language: en; institutionCode: CNC; collectionCode: Insects; basisOfRecord: Pinned Specimen
- **Type status:** Paratype. **Occurrence:** occurrenceDetails: http://janzen.sas.upenn.edu; catalogNumber: DHJPAR0022938; recordedBy: D.H. Janzen & W. Hallwachs; individualID: DHJPAR0022938; individualCount: 1; lifeStage: adult; preparations: pinned; otherCatalogNumbers: 05-SRNP-2168; **Taxon:** scientificName: Spathidexia marioburgosi; phylum: Arthropoda; class: Insecta; order: Diptera; family: Tachinidae; genus: Spathidexia; specificEpithet: marioburgosi; scientificNameAuthorship: Fleming & Wood, 2015; **Location:** continent: Central America; country: Costa Rica; countryCode: CR; stateProvince: Alajuela; county: Area de Conservacion Guanacaste; locality: Sector San Cristobal; verbatimLocality: Sendero Pinyal; verbatimElevation: 630; verbatimLatitude: 10.872; verbatimLongitude: -85.393; verbatimCoordinateSystem: Decimal; decimalLatitude: 10.872; decimalLongitude: -85.393; **Identification:** identifiedBy: AJ Fleming; dateIdentified: 2014; **Event:** samplingProtocol: reared from caterpillar of *Talides sinois* (Hesperiidae); verbatimEventDate: 05-May-2005; **Record Level:** language: en; institutionCode: CNC; collectionCode: Insects; basisOfRecord: Pinned Specimen
- **Type status:** Paratype. **Occurrence:** occurrenceDetails: http://janzen.sas.upenn.edu; catalogNumber: DHJPAR0022939; recordedBy: D.H. Janzen & W. Hallwachs; individualID: DHJPAR0022939; individualCount: 1; lifeStage: adult; preparations: pinned; otherCatalogNumbers: 05-SRNP-21176; **Taxon:** scientificName: Spathidexia marioburgosi; phylum: Arthropoda; class: Insecta; order: Diptera; family: Tachinidae; genus: Spathidexia; specificEpithet: marioburgosi; scientificNameAuthorship: Fleming & Wood, 2015; **Location:** continent: Central America; country: Costa Rica; countryCode: CR; stateProvince: Guanacaste; county: Area de Conservacion Guanacaste; locality: Sector Del Oro; verbatimLocality: Tangelo; verbatimElevation: 410; verbatimLatitude: 11.018; verbatimLongitude: -85.45; verbatimCoordinateSystem: Decimal; decimalLatitude: 11.018; decimalLongitude: -85.45; **Identification:** identifiedBy: AJ Fleming; dateIdentified: 2014; **Event:** samplingProtocol: reared from caterpillar of *Talides sinois* (Hesperiidae); verbatimEventDate: 30-Mar-2005; **Record Level:** language: en; institutionCode: CNC; collectionCode: Insects; basisOfRecord: Pinned Specimen
- **Type status:** Paratype. **Occurrence:** occurrenceDetails: http://janzen.sas.upenn.edu; catalogNumber: DHJPAR0022940; recordedBy: D.H. Janzen & W. Hallwachs; individualID: DHJPAR0022940; individualCount: 1; lifeStage: adult; preparations: pinned; otherCatalogNumbers: 04-SRNP-48022; **Taxon:** scientificName: Spathidexia marioburgosi; phylum: Arthropoda; class: Insecta; order: Diptera; family: Tachinidae; genus: Spathidexia; specificEpithet: marioburgosi; scientificNameAuthorship: Fleming & Wood, 2015; **Location:** continent: Central America; country: Costa Rica; countryCode: CR; stateProvince: Guanacaste; county: Area de Conservacion Guanacaste; locality: Sector Cacao; verbatimLocality: Quebrada Otilio; verbatimElevation: 550; verbatimLatitude: 10.89; verbatimLongitude: -85.48; verbatimCoordinateSystem: Decimal; decimalLatitude: 10.89; decimalLongitude: -85.48; **Identification:** identifiedBy: AJ Fleming; dateIdentified: 2014; **Event:** samplingProtocol: reared from caterpillar of *Talides sergestus* (Hesperiidae); verbatimEventDate: 14-Sep-2004; **Record Level:** language: en; institutionCode: CNC; collectionCode: Insects; basisOfRecord: Pinned Specimen
- **Type status:** Paratype. **Occurrence:** occurrenceDetails: http://janzen.sas.upenn.edu; catalogNumber: DHJPAR0022941; recordedBy: D.H. Janzen & W. Hallwachs; individualID: DHJPAR0022941; individualCount: 1; lifeStage: adult; preparations: pinned; otherCatalogNumbers: 04-SRNP-48423; **Taxon:** scientificName: Spathidexia marioburgosi; phylum: Arthropoda; class: Insecta; order: Diptera; family: Tachinidae; genus: Spathidexia; specificEpithet: marioburgosi; scientificNameAuthorship: Fleming & Wood, 2015; **Location:** continent: Central America; country: Costa Rica; countryCode: CR; stateProvince: Guanacaste; county: Area de Conservacion Guanacaste; locality: Sector Cacao; verbatimLocality: Quebrada Heliconia; verbatimElevation: 390; verbatimLatitude: 10.886; verbatimLongitude: -85.492; verbatimCoordinateSystem: Decimal; decimalLatitude: 10.886; decimalLongitude: -85.492; **Identification:** identifiedBy: AJ Fleming; dateIdentified: 2014; **Event:** samplingProtocol: reared from caterpillar of *Talides sergestus* (Hesperiidae); verbatimEventDate: 05-Oct-2004; **Record Level:** language: en; institutionCode: CNC; collectionCode: Insects; basisOfRecord: Pinned Specimen
- **Type status:** Paratype. **Occurrence:** occurrenceDetails: http://janzen.sas.upenn.edu; catalogNumber: DHJPAR0022942; recordedBy: D.H. Janzen & W. Hallwachs; individualID: DHJPAR0022942; individualCount: 1; lifeStage: adult; preparations: pinned; otherCatalogNumbers: 04-SRNP-48023; **Taxon:** scientificName: Spathidexia marioburgosi; phylum: Arthropoda; class: Insecta; order: Diptera; family: Tachinidae; genus: Spathidexia; specificEpithet: marioburgosi; scientificNameAuthorship: Fleming & Wood, 2015; **Location:** continent: Central America; country: Costa Rica; countryCode: CR; stateProvince: Guanacaste; county: Area de Conservacion Guanacaste; locality: Sector Cacao; verbatimLocality: Quebrada Otilio; verbatimElevation: 550; verbatimLatitude: 10.89; verbatimLongitude: -85.48; verbatimCoordinateSystem: Decimal; decimalLatitude: 10.89; decimalLongitude: -85.48; **Identification:** identifiedBy: AJ Fleming; dateIdentified: 2014; **Event:** samplingProtocol: reared from caterpillar of *Talides* Burns04 (Hesperiidae); verbatimEventDate: 07-Sep-2004; **Record Level:** language: en; institutionCode: CNC; collectionCode: Insects; basisOfRecord: Pinned Specimen
- **Type status:** Paratype. **Occurrence:** occurrenceDetails: http://janzen.sas.upenn.edu; catalogNumber: DHJPAR0022943; recordedBy: D.H. Janzen & W. Hallwachs; individualID: DHJPAR0022943; individualCount: 1; lifeStage: adult; preparations: pinned; otherCatalogNumbers: 04-SRNP-48015; **Taxon:** scientificName: Spathidexia marioburgosi; phylum: Arthropoda; class: Insecta; order: Diptera; family: Tachinidae; genus: Spathidexia; specificEpithet: marioburgosi; scientificNameAuthorship: Fleming & Wood, 2015; **Location:** continent: Central America; country: Costa Rica; countryCode: CR; stateProvince: Guanacaste; county: Area de Conservacion Guanacaste; locality: Sector Cacao; verbatimLocality: Quebrada Otilio; verbatimElevation: 550; verbatimLatitude: 10.89; verbatimLongitude: -85.48; verbatimCoordinateSystem: Decimal; decimalLatitude: 10.89; decimalLongitude: -85.48; **Identification:** identifiedBy: AJ Fleming; dateIdentified: 2014; **Event:** samplingProtocol: reared from caterpillar of *Talides sinois* (Hesperiidae); verbatimEventDate: 06-Sep-2004; **Record Level:** language: en; institutionCode: CNC; collectionCode: Insects; basisOfRecord: Pinned Specimen
- **Type status:** Paratype. **Occurrence:** occurrenceDetails: http://janzen.sas.upenn.edu; catalogNumber: DHJPAR0022944; recordedBy: D.H. Janzen & W. Hallwachs; individualID: DHJPAR0022944; individualCount: 1; lifeStage: adult; preparations: pinned; otherCatalogNumbers: 04-SRNP-48263; **Taxon:** scientificName: Spathidexia marioburgosi; phylum: Arthropoda; class: Insecta; order: Diptera; family: Tachinidae; genus: Spathidexia; specificEpithet: marioburgosi; scientificNameAuthorship: Fleming & Wood, 2015; **Location:** continent: Central America; country: Costa Rica; countryCode: CR; stateProvince: Guanacaste; county: Area de Conservacion Guanacaste; locality: Sector Cacao; verbatimLocality: Gongora Bananal; verbatimElevation: 600; verbatimLatitude: 10.889; verbatimLongitude: -85.476; verbatimCoordinateSystem: Decimal; decimalLatitude: 10.889; decimalLongitude: -85.476; **Identification:** identifiedBy: AJ Fleming; dateIdentified: 2014; **Event:** samplingProtocol: reared from caterpillar of *Talides* Burns04 (Hesperiidae); verbatimEventDate: 26-Sep-2004; **Record Level:** language: en; institutionCode: CNC; collectionCode: Insects; basisOfRecord: Pinned Specimen
- **Type status:** Paratype. **Occurrence:** occurrenceDetails: http://janzen.sas.upenn.edu; catalogNumber: DHJPAR0022945; recordedBy: D.H. Janzen & W. Hallwachs; individualID: DHJPAR0022945; individualCount: 1; lifeStage: adult; preparations: pinned; otherCatalogNumbers: 04-SRNP-47944; **Taxon:** scientificName: Spathidexia marioburgosi; phylum: Arthropoda; class: Insecta; order: Diptera; family: Tachinidae; genus: Spathidexia; specificEpithet: marioburgosi; scientificNameAuthorship: Fleming & Wood, 2015; **Location:** continent: Central America; country: Costa Rica; countryCode: CR; stateProvince: Guanacaste; county: Area de Conservacion Guanacaste; locality: Sector Cacao; verbatimLocality: Cuesta Caimito; verbatimElevation: 640; verbatimLatitude: 10.891; verbatimLongitude: -85.472; verbatimCoordinateSystem: Decimal; decimalLatitude: 10.891; decimalLongitude: -85.472; **Identification:** identifiedBy: AJ Fleming; dateIdentified: 2014; **Event:** samplingProtocol: reared from caterpillar of *Talides* Burns02 (Hesperiidae); verbatimEventDate: 01-Sep-2004; **Record Level:** language: en; institutionCode: CNC; collectionCode: Insects; basisOfRecord: Pinned Specimen
- **Type status:** Paratype. **Occurrence:** occurrenceDetails: http://janzen.sas.upenn.edu; catalogNumber: DHJPAR0022946; recordedBy: D.H. Janzen & W. Hallwachs; individualID: DHJPAR0022946; individualCount: 1; lifeStage: adult; preparations: pinned; otherCatalogNumbers: 04-SRNP-34306; **Taxon:** scientificName: Spathidexia marioburgosi; phylum: Arthropoda; class: Insecta; order: Diptera; family: Tachinidae; genus: Spathidexia; specificEpithet: marioburgosi; scientificNameAuthorship: Fleming & Wood, 2015; **Location:** continent: Central America; country: Costa Rica; countryCode: CR; stateProvince: Guanacaste; county: Area de Conservacion Guanacaste; locality: Sector Pitilla; verbatimLocality: Estacion Pitilla; verbatimElevation: 675; verbatimLatitude: 10.989; verbatimLongitude: -85.426; verbatimCoordinateSystem: Decimal; decimalLatitude: 10.989; decimalLongitude: -85.426; **Identification:** identifiedBy: AJ Fleming; dateIdentified: 2014; **Event:** samplingProtocol: reared from caterpillar of *Talides sinois* (Hesperiidae); verbatimEventDate: 22-Sep-2004; **Record Level:** language: en; institutionCode: CNC; collectionCode: Insects; basisOfRecord: Pinned Specimen
- **Type status:** Paratype. **Occurrence:** occurrenceDetails: http://janzen.sas.upenn.edu; catalogNumber: DHJPAR0022947; recordedBy: D.H. Janzen & W. Hallwachs; individualID: DHJPAR0022947; individualCount: 1; lifeStage: adult; preparations: pinned; otherCatalogNumbers: 04-SRNP-47476; **Taxon:** scientificName: Spathidexia marioburgosi; phylum: Arthropoda; class: Insecta; order: Diptera; family: Tachinidae; genus: Spathidexia; specificEpithet: marioburgosi; scientificNameAuthorship: Fleming & Wood, 2015; **Location:** continent: Central America; country: Costa Rica; countryCode: CR; stateProvince: Guanacaste; county: Area de Conservacion Guanacaste; locality: Sector Cacao; verbatimLocality: Gongora Bananal; verbatimElevation: 600; verbatimLatitude: 10.889; verbatimLongitude: -85.476; verbatimCoordinateSystem: Decimal; decimalLatitude: 10.889; decimalLongitude: -85.476; **Identification:** identifiedBy: AJ Fleming; dateIdentified: 2014; **Event:** samplingProtocol: reared from caterpillar of *Talides sergestus* (Hesperiidae); verbatimEventDate: 17-Aug-2004; **Record Level:** language: en; institutionCode: CNC; collectionCode: Insects; basisOfRecord: Pinned Specimen
- **Type status:** Paratype. **Occurrence:** occurrenceDetails: http://janzen.sas.upenn.edu; catalogNumber: DHJPAR0022948; recordedBy: D.H. Janzen & W. Hallwachs; individualID: DHJPAR0022948; individualCount: 1; lifeStage: adult; preparations: pinned; otherCatalogNumbers: 04-SRNP-48018; **Taxon:** scientificName: Spathidexia marioburgosi; phylum: Arthropoda; class: Insecta; order: Diptera; family: Tachinidae; genus: Spathidexia; specificEpithet: marioburgosi; scientificNameAuthorship: Fleming & Wood, 2015; **Location:** continent: Central America; country: Costa Rica; countryCode: CR; stateProvince: Guanacaste; county: Area de Conservacion Guanacaste; locality: Sector Cacao; verbatimLocality: Quebrada Otilio; verbatimElevation: 550; verbatimLatitude: 10.89; verbatimLongitude: -85.48; verbatimCoordinateSystem: Decimal; decimalLatitude: 10.89; decimalLongitude: -85.48; **Identification:** identifiedBy: AJ Fleming; dateIdentified: 2014; **Event:** samplingProtocol: reared from caterpillar of *Talides sinois* (Hesperiidae); verbatimEventDate: 10-Sep-2004; **Record Level:** language: en; institutionCode: CNC; collectionCode: Insects; basisOfRecord: Pinned Specimen
- **Type status:** Paratype. **Occurrence:** occurrenceDetails: http://janzen.sas.upenn.edu; catalogNumber: DHJPAR0022949; recordedBy: D.H. Janzen & W. Hallwachs; individualID: DHJPAR0022949; individualCount: 1; lifeStage: adult; preparations: pinned; otherCatalogNumbers: 05-SRNP-47227; **Taxon:** scientificName: Spathidexia marioburgosi; phylum: Arthropoda; class: Insecta; order: Diptera; family: Tachinidae; genus: Spathidexia; specificEpithet: marioburgosi; scientificNameAuthorship: Fleming & Wood, 2015; **Location:** continent: Central America; country: Costa Rica; countryCode: CR; stateProvince: Guanacaste; county: Area de Conservacion Guanacaste; locality: Sector Cacao; verbatimLocality: Puente Gongora; verbatimElevation: 540; verbatimLatitude: 10.885; verbatimLongitude: -85.472; verbatimCoordinateSystem: Decimal; decimalLatitude: 10.885; decimalLongitude: -85.472; **Identification:** identifiedBy: AJ Fleming; dateIdentified: 2014; **Event:** samplingProtocol: reared from caterpillar of *Talides* Burns04 (Hesperiidae); verbatimEventDate: 15-Sep-2005; **Record Level:** language: en; institutionCode: CNC; collectionCode: Insects; basisOfRecord: Pinned Specimen
- **Type status:** Paratype. **Occurrence:** occurrenceDetails: http://janzen.sas.upenn.edu; catalogNumber: DHJPAR0022950; recordedBy: D.H. Janzen & W. Hallwachs; individualID: DHJPAR0022950; individualCount: 1; lifeStage: adult; preparations: pinned; otherCatalogNumbers: 04-SRNP-4450; **Taxon:** scientificName: Spathidexia marioburgosi; phylum: Arthropoda; class: Insecta; order: Diptera; family: Tachinidae; genus: Spathidexia; specificEpithet: marioburgosi; scientificNameAuthorship: Fleming & Wood, 2015; **Location:** continent: Central America; country: Costa Rica; countryCode: CR; stateProvince: Alajuela; county: Area de Conservacion Guanacaste; locality: Sector San Cristobal; verbatimLocality: Vado Rio Cucaracho; verbatimElevation: 640; verbatimLatitude: 10.87; verbatimLongitude: -85.392; verbatimCoordinateSystem: Decimal; decimalLatitude: 10.87; decimalLongitude: -85.392; **Identification:** identifiedBy: AJ Fleming; dateIdentified: 2014; **Event:** samplingProtocol: reared from caterpillar of *Talides* Burns04 (Hesperiidae); verbatimEventDate: 27-Sep-2004; **Record Level:** language: en; institutionCode: CNC; collectionCode: Insects; basisOfRecord: Pinned Specimen
- **Type status:** Paratype. **Occurrence:** occurrenceDetails: http://janzen.sas.upenn.edu; catalogNumber: DHJPAR0022951; recordedBy: D.H. Janzen & W. Hallwachs; individualID: DHJPAR0022951; individualCount: 1; lifeStage: adult; preparations: pinned; otherCatalogNumbers: 04-SRNP-48014; **Taxon:** scientificName: Spathidexia marioburgosi; phylum: Arthropoda; class: Insecta; order: Diptera; family: Tachinidae; genus: Spathidexia; specificEpithet: marioburgosi; scientificNameAuthorship: Fleming & Wood, 2015; **Location:** continent: Central America; country: Costa Rica; countryCode: CR; stateProvince: Guanacaste; county: Area de Conservacion Guanacaste; locality: Sector Cacao; verbatimLocality: Quebrada Otilio; verbatimElevation: 550; verbatimLatitude: 10.89; verbatimLongitude: -85.48; verbatimCoordinateSystem: Decimal; decimalLatitude: 10.89; decimalLongitude: -85.48; **Identification:** identifiedBy: AJ Fleming; dateIdentified: 2014; **Event:** samplingProtocol: reared from caterpillar of *Talides sinois* (Hesperiidae); verbatimEventDate: 13-Sep-2004; **Record Level:** language: en; institutionCode: CNC; collectionCode: Insects; basisOfRecord: Pinned Specimen
- **Type status:** Paratype. **Occurrence:** occurrenceDetails: http://janzen.sas.upenn.edu; catalogNumber: DHJPAR0022952; recordedBy: D.H. Janzen & W. Hallwachs; individualID: DHJPAR0022952; individualCount: 1; lifeStage: adult; preparations: pinned; otherCatalogNumbers: 04-SRNP-48856; **Taxon:** scientificName: Spathidexia marioburgosi; phylum: Arthropoda; class: Insecta; order: Diptera; family: Tachinidae; genus: Spathidexia; specificEpithet: marioburgosi; scientificNameAuthorship: Fleming & Wood, 2015; **Location:** continent: Central America; country: Costa Rica; countryCode: CR; stateProvince: Guanacaste; county: Area de Conservacion Guanacaste; locality: Sector Cacao; verbatimLocality: Gongora Bananal; verbatimElevation: 600; verbatimLatitude: 10.889; verbatimLongitude: -85.476; verbatimCoordinateSystem: Decimal; decimalLatitude: 10.889; decimalLongitude: -85.476; **Identification:** identifiedBy: AJ Fleming; dateIdentified: 2014; **Event:** samplingProtocol: reared from caterpillar of *Talides sergestus* (Hesperiidae); verbatimEventDate: 10-Oct-2004; **Record Level:** language: en; institutionCode: CNC; collectionCode: Insects; basisOfRecord: Pinned Specimen
- **Type status:** Paratype. **Occurrence:** occurrenceDetails: http://janzen.sas.upenn.edu; catalogNumber: DHJPAR0023084; recordedBy: D.H. Janzen & W. Hallwachs; individualID: DHJPAR0023084; individualCount: 1; lifeStage: adult; preparations: pinned; otherCatalogNumbers: 04-SRNP-47275; **Taxon:** scientificName: Spathidexia marioburgosi; phylum: Arthropoda; class: Insecta; order: Diptera; family: Tachinidae; genus: Spathidexia; specificEpithet: marioburgosi; scientificNameAuthorship: Fleming & Wood, 2015; **Location:** continent: Central America; country: Costa Rica; countryCode: CR; stateProvince: Guanacaste; county: Area de Conservacion Guanacaste; locality: Sector Cacao; verbatimLocality: Gongora Bananal; verbatimElevation: 600; verbatimLatitude: 10.889; verbatimLongitude: -85.476; verbatimCoordinateSystem: Decimal; decimalLatitude: 10.889; decimalLongitude: -85.476; **Identification:** identifiedBy: AJ Fleming; dateIdentified: 2014; **Event:** samplingProtocol: reared from caterpillar of *Talides sinois* (Hesperiidae); verbatimEventDate: 16-Aug-2004; **Record Level:** language: en; institutionCode: CNC; collectionCode: Insects; basisOfRecord: Pinned Specimen
- **Type status:** Paratype. **Occurrence:** occurrenceDetails: http://janzen.sas.upenn.edu; catalogNumber: DHJPAR0023086; recordedBy: D.H. Janzen & W. Hallwachs; individualID: DHJPAR0023086; individualCount: 1; lifeStage: adult; preparations: pinned; otherCatalogNumbers: 07-SRNP-24167; **Taxon:** scientificName: Spathidexia marioburgosi; phylum: Arthropoda; class: Insecta; order: Diptera; family: Tachinidae; genus: Spathidexia; specificEpithet: marioburgosi; scientificNameAuthorship: Fleming & Wood, 2015; **Location:** continent: Central America; country: Costa Rica; countryCode: CR; stateProvince: Guanacaste; county: Area de Conservacion Guanacaste; locality: Sector Del Oro; verbatimLocality: Bosque Aguirre; verbatimElevation: 620; verbatimLatitude: 11.001; verbatimLongitude: -85.438; verbatimCoordinateSystem: Decimal; decimalLatitude: 11.001; decimalLongitude: -85.438; **Identification:** identifiedBy: AJ Fleming; dateIdentified: 2014; **Event:** samplingProtocol: reared from caterpillar of *Talides* Burns04 (Hesperiidae); verbatimEventDate: 04-Dec-2007; **Record Level:** language: en; institutionCode: CNC; collectionCode: Insects; basisOfRecord: Pinned Specimen
- **Type status:** Paratype. **Occurrence:** occurrenceDetails: http://janzen.sas.upenn.edu; catalogNumber: DHJPAR0023665; recordedBy: D.H. Janzen & W. Hallwachs; individualID: DHJPAR0023665; individualCount: 1; lifeStage: adult; preparations: pinned; otherCatalogNumbers: 07-SRNP-65945; **Taxon:** scientificName: Spathidexia marioburgosi; phylum: Arthropoda; class: Insecta; order: Diptera; family: Tachinidae; genus: Spathidexia; specificEpithet: marioburgosi; scientificNameAuthorship: Fleming & Wood, 2015; **Location:** continent: Central America; country: Costa Rica; countryCode: CR; stateProvince: Alajuela; county: Area de Conservacion Guanacaste; locality: Brasilia; verbatimLocality: Piedrona; verbatimElevation: 340; verbatimLatitude: 11.016; verbatimLongitude: -85.359; verbatimCoordinateSystem: Decimal; decimalLatitude: 11.016; decimalLongitude: -85.359; **Identification:** identifiedBy: AJ Fleming; dateIdentified: 2014; **Event:** samplingProtocol: reared from caterpillar of *Talides sinois* (Hesperiidae); verbatimEventDate: 11-Dec-2007; **Record Level:** language: en; institutionCode: CNC; collectionCode: Insects; basisOfRecord: Pinned Specimen
- **Type status:** Paratype. **Occurrence:** occurrenceDetails: http://janzen.sas.upenn.edu; catalogNumber: DHJPAR0027837; recordedBy: D.H. Janzen & W. Hallwachs; individualID: DHJPAR0027837; individualCount: 1; lifeStage: adult; preparations: pinned; otherCatalogNumbers: 08-SRNP-40369; **Taxon:** scientificName: Spathidexia marioburgosi; phylum: Arthropoda; class: Insecta; order: Diptera; family: Tachinidae; genus: Spathidexia; specificEpithet: marioburgosi; scientificNameAuthorship: Fleming & Wood, 2015; **Location:** continent: Central America; country: Costa Rica; countryCode: CR; stateProvince: Alajuela; county: Area de Conservacion Guanacaste; locality: Sector Rincon Rain Forest; verbatimLocality: Sendero Juntas; verbatimElevation: 400; verbatimLatitude: 10.907; verbatimLongitude: -85.288; verbatimCoordinateSystem: Decimal; decimalLatitude: 10.907; decimalLongitude: -85.288; **Identification:** identifiedBy: AJ Fleming; dateIdentified: 2014; **Event:** samplingProtocol: reared from caterpillar of *Talides sinois* (Hesperiidae); verbatimEventDate: 03-Mar-2008; **Record Level:** language: en; institutionCode: CNC; collectionCode: Insects; basisOfRecord: Pinned Specimen
- **Type status:** Paratype. **Occurrence:** occurrenceDetails: http://janzen.sas.upenn.edu; catalogNumber: DHJPAR0027838; recordedBy: D.H. Janzen & W. Hallwachs; individualID: DHJPAR0027838; individualCount: 1; lifeStage: adult; preparations: pinned; otherCatalogNumbers: 08-SRNP-57067; **Taxon:** scientificName: Spathidexia marioburgosi; phylum: Arthropoda; class: Insecta; order: Diptera; family: Tachinidae; genus: Spathidexia; specificEpithet: marioburgosi; scientificNameAuthorship: Fleming & Wood, 2015; **Location:** continent: Central America; country: Costa Rica; countryCode: CR; stateProvince: Guanacaste; county: Area de Conservacion Guanacaste; locality: Sector Mundo Nuevo; verbatimLocality: Vado Huacas; verbatimElevation: 490; verbatimLatitude: 10.755; verbatimLongitude: -85.391; verbatimCoordinateSystem: Decimal; decimalLatitude: 10.755; decimalLongitude: -85.391; **Identification:** identifiedBy: AJ Fleming; dateIdentified: 2014; **Event:** samplingProtocol: reared from caterpillar of *Talides sinois* (Hesperiidae); verbatimEventDate: 26-Aug-2008; **Record Level:** language: en; institutionCode: CNC; collectionCode: Insects; basisOfRecord: Pinned Specimen
- **Type status:** Paratype. **Occurrence:** occurrenceDetails: http://janzen.sas.upenn.edu; catalogNumber: DHJPAR0029642; recordedBy: D.H. Janzen & W. Hallwachs; individualID: DHJPAR0029642; individualCount: 1; lifeStage: adult; preparations: pinned; otherCatalogNumbers: 08-SRNP-57068; **Taxon:** scientificName: Spathidexia marioburgosi; phylum: Arthropoda; class: Insecta; order: Diptera; family: Tachinidae; genus: Spathidexia; specificEpithet: marioburgosi; scientificNameAuthorship: Fleming & Wood, 2015; **Location:** continent: Central America; country: Costa Rica; countryCode: CR; stateProvince: Guanacaste; county: Area de Conservacion Guanacaste; locality: Sector Mundo Nuevo; verbatimLocality: Vado Huacas; verbatimElevation: 490; verbatimLatitude: 10.755; verbatimLongitude: -85.391; verbatimCoordinateSystem: Decimal; decimalLatitude: 10.755; decimalLongitude: -85.391; **Identification:** identifiedBy: AJ Fleming; dateIdentified: 2014; **Event:** samplingProtocol: reared from caterpillar of *Talides sinois* (Hesperiidae); verbatimEventDate: 27-Aug-2008; **Record Level:** language: en; institutionCode: CNC; collectionCode: Insects; basisOfRecord: Pinned Specimen
- **Type status:** Paratype. **Occurrence:** occurrenceDetails: http://janzen.sas.upenn.edu; catalogNumber: DHJPAR0029715; recordedBy: D.H. Janzen & W. Hallwachs; individualID: DHJPAR0029715; individualCount: 1; lifeStage: adult; preparations: pinned; otherCatalogNumbers: 08-SRNP-5240; **Taxon:** scientificName: Spathidexia marioburgosi; phylum: Arthropoda; class: Insecta; order: Diptera; family: Tachinidae; genus: Spathidexia; specificEpithet: marioburgosi; scientificNameAuthorship: Fleming & Wood, 2015; **Location:** continent: Central America; country: Costa Rica; countryCode: CR; stateProvince: Alajuela; county: Area de Conservacion Guanacaste; locality: Sector San Cristobal; verbatimLocality: Finca San Gabriel; verbatimElevation: 645; verbatimLatitude: 10.878; verbatimLongitude: -85.393; verbatimCoordinateSystem: Decimal; decimalLatitude: 10.878; decimalLongitude: -85.393; **Identification:** identifiedBy: AJ Fleming; dateIdentified: 2014; **Event:** samplingProtocol: reared from caterpillar of *Talides sinois* (Hesperiidae); verbatimEventDate: 21-Oct-2008; **Record Level:** language: en; institutionCode: CNC; collectionCode: Insects; basisOfRecord: Pinned Specimen
- **Type status:** Paratype. **Occurrence:** occurrenceDetails: http://janzen.sas.upenn.edu; catalogNumber: DHJPAR0029717; recordedBy: D.H. Janzen & W. Hallwachs; individualID: DHJPAR0029717; individualCount: 1; lifeStage: adult; preparations: pinned; otherCatalogNumbers: 08-SRNP-22738; **Taxon:** scientificName: Spathidexia marioburgosi; phylum: Arthropoda; class: Insecta; order: Diptera; family: Tachinidae; genus: Spathidexia; specificEpithet: marioburgosi; scientificNameAuthorship: Fleming & Wood, 2015; **Location:** continent: Central America; country: Costa Rica; countryCode: CR; stateProvince: Guanacaste; county: Area de Conservacion Guanacaste; locality: Sector Del Oro; verbatimLocality: Uncaria; verbatimElevation: 370; verbatimLatitude: 11.018; verbatimLongitude: -85.474; verbatimCoordinateSystem: Decimal; decimalLatitude: 11.018; decimalLongitude: -85.474; **Identification:** identifiedBy: AJ Fleming; dateIdentified: 2014; **Event:** samplingProtocol: reared from caterpillar of *Talides sergestus* (Hesperiidae); verbatimEventDate: 09-Sep-2008; **Record Level:** language: en; institutionCode: CNC; collectionCode: Insects; basisOfRecord: Pinned Specimen
- **Type status:** Paratype. **Occurrence:** occurrenceDetails: http://janzen.sas.upenn.edu; catalogNumber: DHJPAR0029719; recordedBy: D.H. Janzen & W. Hallwachs; individualID: DHJPAR0029719; individualCount: 1; lifeStage: adult; preparations: pinned; otherCatalogNumbers: 08-SRNP-22720; **Taxon:** scientificName: Spathidexia marioburgosi; phylum: Arthropoda; class: Insecta; order: Diptera; family: Tachinidae; genus: Spathidexia; specificEpithet: marioburgosi; scientificNameAuthorship: Fleming & Wood, 2015; **Location:** continent: Central America; country: Costa Rica; countryCode: CR; stateProvince: Guanacaste; county: Area de Conservacion Guanacaste; locality: Sector Del Oro; verbatimLocality: Bosque Aguirre; verbatimElevation: 620; verbatimLatitude: 11.001; verbatimLongitude: -85.438; verbatimCoordinateSystem: Decimal; decimalLatitude: 11.001; decimalLongitude: -85.438; **Identification:** identifiedBy: AJ Fleming; dateIdentified: 2014; **Event:** samplingProtocol: reared from caterpillar of *Talides sergestus* (Hesperiidae); verbatimEventDate: 11-Sep-2008; **Record Level:** language: en; institutionCode: CNC; collectionCode: Insects; basisOfRecord: Pinned Specimen
- **Type status:** Paratype. **Occurrence:** occurrenceDetails: http://janzen.sas.upenn.edu; catalogNumber: DHJPAR0029720; recordedBy: D.H. Janzen & W. Hallwachs; individualID: DHJPAR0029720; individualCount: 1; lifeStage: adult; preparations: pinned; otherCatalogNumbers: 08-SRNP-22723; **Taxon:** scientificName: Spathidexia marioburgosi; phylum: Arthropoda; class: Insecta; order: Diptera; family: Tachinidae; genus: Spathidexia; specificEpithet: marioburgosi; scientificNameAuthorship: Fleming & Wood, 2015; **Location:** continent: Central America; country: Costa Rica; countryCode: CR; stateProvince: Guanacaste; county: Area de Conservacion Guanacaste; locality: Sector Del Oro; verbatimLocality: Bosque Aguirre; verbatimElevation: 620; verbatimLatitude: 11.001; verbatimLongitude: -85.438; verbatimCoordinateSystem: Decimal; decimalLatitude: 11.001; decimalLongitude: -85.438; **Identification:** identifiedBy: AJ Fleming; dateIdentified: 2014; **Event:** samplingProtocol: reared from caterpillar of *Talides sergestus* (Hesperiidae); verbatimEventDate: 12-Sep-2008; **Record Level:** language: en; institutionCode: CNC; collectionCode: Insects; basisOfRecord: Pinned Specimen
- **Type status:** Paratype. **Occurrence:** occurrenceDetails: http://janzen.sas.upenn.edu; catalogNumber: DHJPAR0029721; recordedBy: D.H. Janzen & W. Hallwachs; individualID: DHJPAR0029721; individualCount: 1; lifeStage: adult; preparations: pinned; otherCatalogNumbers: 08-SRNP-22720; **Taxon:** scientificName: Spathidexia marioburgosi; phylum: Arthropoda; class: Insecta; order: Diptera; family: Tachinidae; genus: Spathidexia; specificEpithet: marioburgosi; scientificNameAuthorship: Fleming & Wood, 2015; **Location:** continent: Central America; country: Costa Rica; countryCode: CR; stateProvince: Guanacaste; county: Area de Conservacion Guanacaste; locality: Sector Del Oro; verbatimLocality: Bosque Aguirre; verbatimElevation: 620; verbatimLatitude: 11.001; verbatimLongitude: -85.438; verbatimCoordinateSystem: Decimal; decimalLatitude: 11.001; decimalLongitude: -85.438; **Identification:** identifiedBy: AJ Fleming; dateIdentified: 2014; **Event:** samplingProtocol: reared from caterpillar of *Talides sinois* (Hesperiidae); verbatimEventDate: 11-Sep-2008; **Record Level:** language: en; institutionCode: CNC; collectionCode: Insects; basisOfRecord: Pinned Specimen
- **Type status:** Paratype. **Occurrence:** occurrenceDetails: http://janzen.sas.upenn.edu; catalogNumber: DHJPAR0029985; recordedBy: D.H. Janzen & W. Hallwachs; individualID: DHJPAR0029985; individualCount: 1; lifeStage: adult; preparations: pinned; otherCatalogNumbers: 08-SRNP-5243; **Taxon:** scientificName: Spathidexia marioburgosi; phylum: Arthropoda; class: Insecta; order: Diptera; family: Tachinidae; genus: Spathidexia; specificEpithet: marioburgosi; scientificNameAuthorship: Fleming & Wood, 2015; **Location:** continent: Central America; country: Costa Rica; countryCode: CR; stateProvince: Alajuela; county: Area de Conservacion Guanacaste; locality: Sector San Cristobal; verbatimLocality: Finca San Gabriel; verbatimElevation: 645; verbatimLatitude: 10.878; verbatimLongitude: -85.393; verbatimCoordinateSystem: Decimal; decimalLatitude: 10.878; decimalLongitude: -85.393; **Identification:** identifiedBy: AJ Fleming; dateIdentified: 2014; **Event:** samplingProtocol: reared from caterpillar of *Talides sinois* (Hesperiidae); verbatimEventDate: 28-Oct-2008; **Record Level:** language: en; institutionCode: CNC; collectionCode: Insects; basisOfRecord: Pinned Specimen
- **Type status:** Paratype. **Occurrence:** occurrenceDetails: http://janzen.sas.upenn.edu; catalogNumber: DHJPAR0029988; recordedBy: D.H. Janzen & W. Hallwachs; individualID: DHJPAR0029988; individualCount: 1; lifeStage: adult; preparations: pinned; otherCatalogNumbers: 08-SRNP-5242; **Taxon:** scientificName: Spathidexia marioburgosi; phylum: Arthropoda; class: Insecta; order: Diptera; family: Tachinidae; genus: Spathidexia; specificEpithet: marioburgosi; scientificNameAuthorship: Fleming & Wood, 2015; **Location:** continent: Central America; country: Costa Rica; countryCode: CR; stateProvince: Alajuela; county: Area de Conservacion Guanacaste; locality: Sector San Cristobal; verbatimLocality: Finca San Gabriel; verbatimElevation: 645; verbatimLatitude: 10.878; verbatimLongitude: -85.393; verbatimCoordinateSystem: Decimal; decimalLatitude: 10.878; decimalLongitude: -85.393; **Identification:** identifiedBy: AJ Fleming; dateIdentified: 2014; **Event:** samplingProtocol: reared from caterpillar of *Talides sinois* (Hesperiidae); verbatimEventDate: 29-Oct-2008; **Record Level:** language: en; institutionCode: CNC; collectionCode: Insects; basisOfRecord: Pinned Specimen
- **Type status:** Paratype. **Occurrence:** occurrenceDetails: http://janzen.sas.upenn.edu; catalogNumber: DHJPAR0029994; recordedBy: D.H. Janzen & W. Hallwachs; individualID: DHJPAR0029994; individualCount: 1; lifeStage: adult; preparations: pinned; otherCatalogNumbers: 08-SRNP-5522; **Taxon:** scientificName: Spathidexia marioburgosi; phylum: Arthropoda; class: Insecta; order: Diptera; family: Tachinidae; genus: Spathidexia; specificEpithet: marioburgosi; scientificNameAuthorship: Fleming & Wood, 2015; **Location:** continent: Central America; country: Costa Rica; countryCode: CR; stateProvince: Alajuela; county: Area de Conservacion Guanacaste; locality: Sector San Cristobal; verbatimLocality: Sendero Huerta; verbatimElevation: 527; verbatimLatitude: 10.931; verbatimLongitude: -85.372; verbatimCoordinateSystem: Decimal; decimalLatitude: 10.931; decimalLongitude: -85.372; **Identification:** identifiedBy: AJ Fleming; dateIdentified: 2014; **Event:** samplingProtocol: reared from caterpillar of *Talides sinois* (Hesperiidae); verbatimEventDate: 08-Nov-2008; **Record Level:** language: en; institutionCode: CNC; collectionCode: Insects; basisOfRecord: Pinned Specimen
- **Type status:** Paratype. **Occurrence:** occurrenceDetails: http://janzen.sas.upenn.edu; catalogNumber: DHJPAR0030016; recordedBy: D.H. Janzen & W. Hallwachs; individualID: DHJPAR0030016; individualCount: 1; lifeStage: adult; preparations: pinned; otherCatalogNumbers: 08-SRNP-6840; **Taxon:** scientificName: Spathidexia marioburgosi; phylum: Arthropoda; class: Insecta; order: Diptera; family: Tachinidae; genus: Spathidexia; specificEpithet: marioburgosi; scientificNameAuthorship: Fleming & Wood, 2015; **Location:** continent: Central America; country: Costa Rica; countryCode: CR; stateProvince: Alajuela; county: Area de Conservacion Guanacaste; locality: Sector San Cristobal; verbatimLocality: Sendero Huerta; verbatimElevation: 527; verbatimLatitude: 10.931; verbatimLongitude: -85.372; verbatimCoordinateSystem: Decimal; decimalLatitude: 10.931; decimalLongitude: -85.372; **Identification:** identifiedBy: AJ Fleming; dateIdentified: 2014; **Event:** samplingProtocol: reared from caterpillar of *Talides sinois* (Hesperiidae); verbatimEventDate: 07-Jan-2009; **Record Level:** language: en; institutionCode: CNC; collectionCode: Insects; basisOfRecord: Pinned Specimen
- **Type status:** Paratype. **Occurrence:** occurrenceDetails: http://janzen.sas.upenn.edu; catalogNumber: DHJPAR0030034; recordedBy: D.H. Janzen & W. Hallwachs; individualID: DHJPAR0030034; individualCount: 1; lifeStage: adult; preparations: pinned; otherCatalogNumbers: 09-SRNP-40082; **Taxon:** scientificName: Spathidexia marioburgosi; phylum: Arthropoda; class: Insecta; order: Diptera; family: Tachinidae; genus: Spathidexia; specificEpithet: marioburgosi; scientificNameAuthorship: Fleming & Wood, 2015; **Location:** continent: Central America; country: Costa Rica; countryCode: CR; stateProvince: Alajuela; county: Area de Conservacion Guanacaste; locality: Sector Rincon Rain Forest; verbatimLocality: Rio Francia Arriba; verbatimElevation: 400; verbatimLatitude: 10.897; verbatimLongitude: -85.29; verbatimCoordinateSystem: Decimal; decimalLatitude: 10.897; decimalLongitude: -85.29; **Identification:** identifiedBy: AJ Fleming; dateIdentified: 2014; **Event:** samplingProtocol: reared from caterpillar of *Talides sergestus* (Hesperiidae); verbatimEventDate: 16-Feb-2009; **Record Level:** language: en; institutionCode: CNC; collectionCode: Insects; basisOfRecord: Pinned Specimen
- **Type status:** Paratype. **Occurrence:** occurrenceDetails: http://janzen.sas.upenn.edu; catalogNumber: DHJPAR0030044; recordedBy: D.H. Janzen & W. Hallwachs; individualID: DHJPAR0030044; individualCount: 1; lifeStage: adult; preparations: pinned; otherCatalogNumbers: 08-SRNP-66133; **Taxon:** scientificName: Spathidexia marioburgosi; phylum: Arthropoda; class: Insecta; order: Diptera; family: Tachinidae; genus: Spathidexia; specificEpithet: marioburgosi; scientificNameAuthorship: Fleming & Wood, 2015; **Location:** continent: Central America; country: Costa Rica; countryCode: CR; stateProvince: Alajuela; county: Area de Conservacion Guanacaste; locality: Brasilia; verbatimLocality: Gallinazo; verbatimElevation: 360; verbatimLatitude: 11.018; verbatimLongitude: -85.372; verbatimCoordinateSystem: Decimal; decimalLatitude: 11.018; decimalLongitude: -85.372; **Identification:** identifiedBy: AJ Fleming; dateIdentified: 2014; **Event:** samplingProtocol: reared from caterpillar of *Talides sinois* (Hesperiidae); verbatimEventDate: 15-Dec-2008; **Record Level:** language: en; institutionCode: CNC; collectionCode: Insects; basisOfRecord: Pinned Specimen
- **Type status:** Paratype. **Occurrence:** occurrenceDetails: http://janzen.sas.upenn.edu; catalogNumber: DHJPAR0030172; recordedBy: D.H. Janzen & W. Hallwachs; individualID: DHJPAR0030172; individualCount: 1; lifeStage: adult; preparations: pinned; otherCatalogNumbers: 08-SRNP-72761; **Taxon:** scientificName: Spathidexia marioburgosi; phylum: Arthropoda; class: Insecta; order: Diptera; family: Tachinidae; genus: Spathidexia; specificEpithet: marioburgosi; scientificNameAuthorship: Fleming & Wood, 2015; **Location:** continent: Central America; country: Costa Rica; countryCode: CR; stateProvince: Guanacaste; county: Area de Conservacion Guanacaste; locality: Sector Pitilla; verbatimLocality: Manguera; verbatimElevation: 470; verbatimLatitude: 10.996; verbatimLongitude: -85.398; verbatimCoordinateSystem: Decimal; decimalLatitude: 10.996; decimalLongitude: -85.398; **Identification:** identifiedBy: AJ Fleming; dateIdentified: 2014; **Event:** samplingProtocol: reared from caterpillar of *Talides sinois* (Hesperiidae); verbatimEventDate: 12-Nov-2008; **Record Level:** language: en; institutionCode: CNC; collectionCode: Insects; basisOfRecord: Pinned Specimen
- **Type status:** Paratype. **Occurrence:** occurrenceDetails: http://janzen.sas.upenn.edu; catalogNumber: DHJPAR0030192; recordedBy: D.H. Janzen & W. Hallwachs; individualID: DHJPAR0030192; individualCount: 1; lifeStage: adult; preparations: pinned; otherCatalogNumbers: 09-SRNP-20174; **Taxon:** scientificName: Spathidexia marioburgosi; phylum: Arthropoda; class: Insecta; order: Diptera; family: Tachinidae; genus: Spathidexia; specificEpithet: marioburgosi; scientificNameAuthorship: Fleming & Wood, 2015; **Location:** continent: Central America; country: Costa Rica; countryCode: CR; stateProvince: Guanacaste; county: Area de Conservacion Guanacaste; locality: Sector Del Oro; verbatimLocality: Bosque Aguirre; verbatimElevation: 620; verbatimLatitude: 11.001; verbatimLongitude: -85.438; verbatimCoordinateSystem: Decimal; decimalLatitude: 11.001; decimalLongitude: -85.438; **Identification:** identifiedBy: AJ Fleming; dateIdentified: 2014; **Event:** samplingProtocol: reared from caterpillar of *Talides sinois* (Hesperiidae); verbatimEventDate: 16-Feb-2009; **Record Level:** language: en; institutionCode: CNC; collectionCode: Insects; basisOfRecord: Pinned Specimen
- **Type status:** Paratype. **Occurrence:** occurrenceDetails: http://janzen.sas.upenn.edu; catalogNumber: DHJPAR0030207; recordedBy: D.H. Janzen & W. Hallwachs; individualID: DHJPAR0030207; individualCount: 1; lifeStage: adult; preparations: pinned; otherCatalogNumbers: 08-SRNP-24204; **Taxon:** scientificName: Spathidexia marioburgosi; phylum: Arthropoda; class: Insecta; order: Diptera; family: Tachinidae; genus: Spathidexia; specificEpithet: marioburgosi; scientificNameAuthorship: Fleming & Wood, 2015; **Location:** continent: Central America; country: Costa Rica; countryCode: CR; stateProvince: Guanacaste; county: Area de Conservacion Guanacaste; locality: Sector Del Oro; verbatimLocality: Puente Quebrada Trigal; verbatimElevation: 290; verbatimLatitude: 11.027; verbatimLongitude: -85.495; verbatimCoordinateSystem: Decimal; decimalLatitude: 11.027; decimalLongitude: -85.495; **Identification:** identifiedBy: AJ Fleming; dateIdentified: 2014; **Event:** samplingProtocol: reared from caterpillar of *Talides sergestus* (Hesperiidae); verbatimEventDate: 30-Nov-2008; **Record Level:** language: en; institutionCode: CNC; collectionCode: Insects; basisOfRecord: Pinned Specimen
- **Type status:** Paratype. **Occurrence:** occurrenceDetails: http://janzen.sas.upenn.edu; catalogNumber: DHJPAR0030233; recordedBy: D.H. Janzen & W. Hallwachs; individualID: DHJPAR0030233; individualCount: 1; lifeStage: adult; preparations: pinned; otherCatalogNumbers: 08-SRNP-5240; **Taxon:** scientificName: Spathidexia marioburgosi; phylum: Arthropoda; class: Insecta; order: Diptera; family: Tachinidae; genus: Spathidexia; specificEpithet: marioburgosi; scientificNameAuthorship: Fleming & Wood, 2015; **Location:** continent: Central America; country: Costa Rica; countryCode: CR; stateProvince: Alajuela; county: Area de Conservacion Guanacaste; locality: Sector San Cristobal; verbatimLocality: Finca San Gabriel; verbatimElevation: 645; verbatimLatitude: 10.878; verbatimLongitude: -85.393; verbatimCoordinateSystem: Decimal; decimalLatitude: 10.878; decimalLongitude: -85.393; **Identification:** identifiedBy: AJ Fleming; dateIdentified: 2014; **Event:** samplingProtocol: reared from caterpillar of *Talides sinois* (Hesperiidae); verbatimEventDate: 21-Oct-2008; **Record Level:** language: en; institutionCode: CNC; collectionCode: Insects; basisOfRecord: Pinned Specimen
- **Type status:** Paratype. **Occurrence:** occurrenceDetails: http://janzen.sas.upenn.edu; catalogNumber: DHJPAR0030427; recordedBy: D.H. Janzen & W. Hallwachs; individualID: DHJPAR0030427; individualCount: 1; lifeStage: adult; preparations: pinned; otherCatalogNumbers: 09-SRNP-593; **Taxon:** scientificName: Spathidexia marioburgosi; phylum: Arthropoda; class: Insecta; order: Diptera; family: Tachinidae; genus: Spathidexia; specificEpithet: marioburgosi; scientificNameAuthorship: Fleming & Wood, 2015; **Location:** continent: Central America; country: Costa Rica; countryCode: CR; stateProvince: Alajuela; county: Area de Conservacion Guanacaste; locality: Sector San Cristobal; verbatimLocality: Puente Palma; verbatimElevation: 460; verbatimLatitude: 10.916; verbatimLongitude: -85.379; verbatimCoordinateSystem: Decimal; decimalLatitude: 10.916; decimalLongitude: -85.379; **Identification:** identifiedBy: AJ Fleming; dateIdentified: 2014; **Event:** samplingProtocol: reared from caterpillar of *Talides sinois* (Hesperiidae); verbatimEventDate: 10-Mar-2009; **Record Level:** language: en; institutionCode: CNC; collectionCode: Insects; basisOfRecord: Pinned Specimen
- **Type status:** Paratype. **Occurrence:** occurrenceDetails: http://janzen.sas.upenn.edu; catalogNumber: DHJPAR0034326; recordedBy: D.H. Janzen & W. Hallwachs; individualID: DHJPAR0034326; individualCount: 1; lifeStage: adult; preparations: pinned; otherCatalogNumbers: 08-SRNP-5242; **Taxon:** scientificName: Spathidexia marioburgosi; phylum: Arthropoda; class: Insecta; order: Diptera; family: Tachinidae; genus: Spathidexia; specificEpithet: marioburgosi; scientificNameAuthorship: Fleming & Wood, 2015; **Location:** continent: Central America; country: Costa Rica; countryCode: CR; stateProvince: Alajuela; county: Area de Conservacion Guanacaste; locality: Sector San Cristobal; verbatimLocality: Finca San Gabriel; verbatimElevation: 645; verbatimLatitude: 10.878; verbatimLongitude: -85.393; verbatimCoordinateSystem: Decimal; decimalLatitude: 10.878; decimalLongitude: -85.393; **Identification:** identifiedBy: AJ Fleming; dateIdentified: 2014; **Event:** samplingProtocol: reared from caterpillar of *Talides sinois* (Hesperiidae); verbatimEventDate: 29-Oct-2008; **Record Level:** language: en; institutionCode: CNC; collectionCode: Insects; basisOfRecord: Pinned Specimen
- **Type status:** Paratype. **Occurrence:** occurrenceDetails: http://janzen.sas.upenn.edu; catalogNumber: DHJPAR0034332; recordedBy: D.H. Janzen & W. Hallwachs; individualID: DHJPAR0034332; individualCount: 1; lifeStage: adult; preparations: pinned; otherCatalogNumbers: 08-SRNP-5522; **Taxon:** scientificName: Spathidexia marioburgosi; phylum: Arthropoda; class: Insecta; order: Diptera; family: Tachinidae; genus: Spathidexia; specificEpithet: marioburgosi; scientificNameAuthorship: Fleming & Wood, 2015; **Location:** continent: Central America; country: Costa Rica; countryCode: CR; stateProvince: Alajuela; county: Area de Conservacion Guanacaste; locality: Sector San Cristobal; verbatimLocality: Sendero Huerta; verbatimElevation: 527; verbatimLatitude: 10.931; verbatimLongitude: -85.372; verbatimCoordinateSystem: Decimal; decimalLatitude: 10.931; decimalLongitude: -85.372; **Identification:** identifiedBy: AJ Fleming; dateIdentified: 2014; **Event:** samplingProtocol: reared from caterpillar of *Talides sinois* (Hesperiidae); verbatimEventDate: 08-Nov-2008; **Record Level:** language: en; institutionCode: CNC; collectionCode: Insects; basisOfRecord: Pinned Specimen
- **Type status:** Paratype. **Occurrence:** occurrenceDetails: http://janzen.sas.upenn.edu; catalogNumber: DHJPAR0034342; recordedBy: D.H. Janzen & W. Hallwachs; individualID: DHJPAR0034342; individualCount: 1; lifeStage: adult; preparations: pinned; otherCatalogNumbers: 08-SRNP-5243; **Taxon:** scientificName: Spathidexia marioburgosi; phylum: Arthropoda; class: Insecta; order: Diptera; family: Tachinidae; genus: Spathidexia; specificEpithet: marioburgosi; scientificNameAuthorship: Fleming & Wood, 2015; **Location:** continent: Central America; country: Costa Rica; countryCode: CR; stateProvince: Alajuela; county: Area de Conservacion Guanacaste; locality: Sector San Cristobal; verbatimLocality: Finca San Gabriel; verbatimElevation: 645; verbatimLatitude: 10.878; verbatimLongitude: -85.393; verbatimCoordinateSystem: Decimal; decimalLatitude: 10.878; decimalLongitude: -85.393; **Identification:** identifiedBy: AJ Fleming; dateIdentified: 2014; **Event:** samplingProtocol: reared from caterpillar of *Talides sinois* (Hesperiidae); verbatimEventDate: 28-Oct-2008; **Record Level:** language: en; institutionCode: CNC; collectionCode: Insects; basisOfRecord: Pinned Specimen
- **Type status:** Paratype. **Occurrence:** occurrenceDetails: http://janzen.sas.upenn.edu; catalogNumber: DHJPAR0034497; recordedBy: D.H. Janzen & W. Hallwachs; individualID: DHJPAR0034497; individualCount: 1; lifeStage: adult; preparations: pinned; otherCatalogNumbers: 09-SRNP-65324; **Taxon:** scientificName: Spathidexia marioburgosi; phylum: Arthropoda; class: Insecta; order: Diptera; family: Tachinidae; genus: Spathidexia; specificEpithet: marioburgosi; scientificNameAuthorship: Fleming & Wood, 2015; **Location:** continent: Central America; country: Costa Rica; countryCode: CR; stateProvince: Alajuela; county: Area de Conservacion Guanacaste; locality: Brasilia; verbatimLocality: Gallinazo; verbatimElevation: 360; verbatimLatitude: 11.018; verbatimLongitude: -85.372; verbatimCoordinateSystem: Decimal; decimalLatitude: 11.018; decimalLongitude: -85.372; **Identification:** identifiedBy: AJ Fleming; dateIdentified: 2014; **Event:** samplingProtocol: reared from caterpillar of *Talides* Burns (Hesperiidae); verbatimEventDate: 18-Mar-2009; **Record Level:** language: en; institutionCode: CNC; collectionCode: Insects; basisOfRecord: Pinned Specimen
- **Type status:** Paratype. **Occurrence:** occurrenceDetails: http://janzen.sas.upenn.edu; catalogNumber: DHJPAR0036458; recordedBy: D.H. Janzen & W. Hallwachs; individualID: DHJPAR0036458; individualCount: 1; lifeStage: adult; preparations: pinned; otherCatalogNumbers: 06-SRNP-4769; **Taxon:** scientificName: Spathidexia marioburgosi; phylum: Arthropoda; class: Insecta; order: Diptera; family: Tachinidae; genus: Spathidexia; specificEpithet: marioburgosi; scientificNameAuthorship: Fleming & Wood, 2015; **Location:** continent: Central America; country: Costa Rica; countryCode: CR; stateProvince: Alajuela; county: Area de Conservacion Guanacaste; locality: Sector San Cristobal; verbatimLocality: Rio Blanco Abajo; verbatimElevation: 500; verbatimLatitude: 10.9; verbatimLongitude: -85.373; verbatimCoordinateSystem: Decimal; decimalLatitude: 10.9; decimalLongitude: -85.373; **Identification:** identifiedBy: AJ Fleming; dateIdentified: 2014; **Event:** samplingProtocol: reared from caterpillar of *Talides sergestus* (Hesperiidae); verbatimEventDate: 16-Jul-2006; **Record Level:** language: en; institutionCode: CNC; collectionCode: Insects; basisOfRecord: Pinned Specimen
- **Type status:** Paratype. **Occurrence:** occurrenceDetails: http://janzen.sas.upenn.edu; catalogNumber: DHJPAR0038602; recordedBy: D.H. Janzen & W. Hallwachs; individualID: DHJPAR0038602; individualCount: 1; lifeStage: adult; preparations: pinned; otherCatalogNumbers: 05-SRNP-7124; **Taxon:** scientificName: Spathidexia marioburgosi; phylum: Arthropoda; class: Insecta; order: Diptera; family: Tachinidae; genus: Spathidexia; specificEpithet: marioburgosi; scientificNameAuthorship: Fleming & Wood, 2015; **Location:** continent: Central America; country: Costa Rica; countryCode: CR; stateProvince: Alajuela; county: Area de Conservacion Guanacaste; locality: Sector San Cristobal; verbatimLocality: Corrales Viejos; verbatimElevation: 495; verbatimLatitude: 10.9; verbatimLongitude: -85.381; verbatimCoordinateSystem: Decimal; decimalLatitude: 10.9; decimalLongitude: -85.381; **Identification:** identifiedBy: AJ Fleming; dateIdentified: 2014; **Event:** samplingProtocol: reared from caterpillar of *Talides sinois* (Hesperiidae); verbatimEventDate: 08-Dec-2005; **Record Level:** language: en; institutionCode: CNC; collectionCode: Insects; basisOfRecord: Pinned Specimen
- **Type status:** Paratype. **Occurrence:** occurrenceDetails: http://janzen.sas.upenn.edu; catalogNumber: DHJPAR0042322; recordedBy: D.H. Janzen & W. Hallwachs; individualID: DHJPAR0042322; individualCount: 1; lifeStage: adult; preparations: pinned; otherCatalogNumbers: 10-SRNP-22751; **Taxon:** scientificName: Spathidexia marioburgosi; phylum: Arthropoda; class: Insecta; order: Diptera; family: Tachinidae; genus: Spathidexia; specificEpithet: marioburgosi; scientificNameAuthorship: Fleming & Wood, 2015; **Location:** continent: Central America; country: Costa Rica; countryCode: CR; stateProvince: Guanacaste; county: Area de Conservacion Guanacaste; locality: Sector Del Oro; verbatimLocality: Bosque Aguirre; verbatimElevation: 620; verbatimLatitude: 11.0006; verbatimLongitude: -85.438; verbatimCoordinateSystem: Decimal; decimalLatitude: 11.0006; decimalLongitude: -85.438; **Identification:** identifiedBy: AJ Fleming; dateIdentified: 2014; **Event:** samplingProtocol: reared from caterpillar of *Thracides phidon* (Hesperiidae); verbatimEventDate: 23-Dec-2010; **Record Level:** language: en; institutionCode: CNC; collectionCode: Insects; basisOfRecord: Pinned Specimen
- **Type status:** Paratype. **Occurrence:** occurrenceDetails: http://janzen.sas.upenn.edu; catalogNumber: DHJPAR0042704; recordedBy: D.H. Janzen & W. Hallwachs; individualID: DHJPAR0042704; individualCount: 1; lifeStage: adult; preparations: pinned; otherCatalogNumbers: 11-SRNP-69824; **Taxon:** scientificName: Spathidexia marioburgosi; phylum: Arthropoda; class: Insecta; order: Diptera; family: Tachinidae; genus: Spathidexia; specificEpithet: marioburgosi; scientificNameAuthorship: Fleming & Wood, 2015; **Location:** continent: Central America; country: Costa Rica; countryCode: CR; stateProvince: Alajuela; county: Area de Conservacion Guanacaste; locality: Sector Rincon Rain Forest; verbatimLocality: Jacobo; verbatimElevation: 461; verbatimLatitude: 10.941; verbatimLongitude: -85.318; verbatimCoordinateSystem: Decimal; decimalLatitude: 10.941; decimalLongitude: -85.318; **Identification:** identifiedBy: AJ Fleming; dateIdentified: 2014; **Event:** samplingProtocol: reared from caterpillar of *Talides sergestus* (Hesperiidae); verbatimEventDate: 14-Apr-2011; **Record Level:** language: en; institutionCode: CNC; collectionCode: Insects; basisOfRecord: Pinned Specimen
- **Type status:** Paratype. **Occurrence:** occurrenceDetails: http://janzen.sas.upenn.edu; catalogNumber: DHJPAR0049601; recordedBy: D.H. Janzen & W. Hallwachs; individualID: DHJPAR0049601; individualCount: 1; lifeStage: adult; preparations: pinned; otherCatalogNumbers: 12-SRNP-2735; **Taxon:** scientificName: Spathidexia marioburgosi; phylum: Arthropoda; class: Insecta; order: Diptera; family: Tachinidae; genus: Spathidexia; specificEpithet: marioburgosi; scientificNameAuthorship: Fleming & Wood, 2015; **Location:** continent: Central America; country: Costa Rica; countryCode: CR; stateProvince: Guanacaste; county: Area de Conservacion Guanacaste; locality: Sector San Cristobal; verbatimLocality: Sendero Perdido; verbatimElevation: 620; verbatimLatitude: 10.8794; verbatimLongitude: -85.38607; verbatimCoordinateSystem: Decimal; decimalLatitude: 10.8794; decimalLongitude: -85.38607; **Identification:** identifiedBy: AJ Fleming; dateIdentified: 2014; **Event:** samplingProtocol: reared from caterpillar of *Talides sinois* (Hesperiidae); verbatimEventDate: 22-Jul-2012; **Record Level:** language: en; institutionCode: CNC; collectionCode: Insects; basisOfRecord: Pinned Specimen
- **Type status:** Paratype. **Occurrence:** occurrenceDetails: http://janzen.sas.upenn.edu; catalogNumber: DHJPAR0046411; recordedBy: D.H. Janzen & W. Hallwachs; individualID: DHJPAR0046411; individualCount: 1; lifeStage: adult; preparations: pinned; otherCatalogNumbers: 11-SRNP-4237; **Taxon:** scientificName: Spathidexia marioburgosi; phylum: Arthropoda; class: Insecta; order: Diptera; family: Tachinidae; genus: Spathidexia; specificEpithet: marioburgosi; scientificNameAuthorship: Fleming & Wood, 2015; **Location:** continent: Central America; country: Costa Rica; countryCode: CR; stateProvince: Alajuela; county: Area de Conservacion Guanacaste; locality: Sector San Cristobal; verbatimLocality: Sendero Huerta; verbatimElevation: 527; verbatimLatitude: 10.9305; verbatimLongitude: -85.37223; verbatimCoordinateSystem: Decimal; decimalLatitude: 10.9305; decimalLongitude: -85.37223; **Identification:** identifiedBy: AJ Fleming; dateIdentified: 2014; **Event:** samplingProtocol: reared from caterpillar of *Talides* Burns (Hesperiidae); verbatimEventDate: 31-Oct-2011; **Record Level:** language: en; institutionCode: CNC; collectionCode: Insects; basisOfRecord: Pinned Specimen
- **Type status:** Paratype. **Occurrence:** occurrenceDetails: http://janzen.sas.upenn.edu; catalogNumber: DHJPAR0046465; recordedBy: D.H. Janzen & W. Hallwachs; individualID: DHJPAR0046465; individualCount: 1; lifeStage: adult; preparations: pinned; otherCatalogNumbers: 11-SRNP-5052; **Taxon:** scientificName: Spathidexia marioburgosi; phylum: Arthropoda; class: Insecta; order: Diptera; family: Tachinidae; genus: Spathidexia; specificEpithet: marioburgosi; scientificNameAuthorship: Fleming & Wood, 2015; **Location:** continent: Central America; country: Costa Rica; countryCode: CR; stateProvince: Alajuela; county: Area de Conservacion Guanacaste; locality: Sector Rincon Rain Forest; verbatimLocality: Camino Albergue Oscar; verbatimElevation: 560; verbatimLatitude: 10.87741; verbatimLongitude: -85.32363; verbatimCoordinateSystem: Decimal; decimalLatitude: 10.87741; decimalLongitude: -85.32363; **Identification:** identifiedBy: AJ Fleming; dateIdentified: 2014; **Event:** samplingProtocol: reared from caterpillar of *Talides* Burns (Hesperiidae); verbatimEventDate: 17-Dec-2011; **Record Level:** language: en; institutionCode: CNC; collectionCode: Insects; basisOfRecord: Pinned Specimen
- **Type status:** Paratype. **Occurrence:** occurrenceDetails: http://janzen.sas.upenn.edu; catalogNumber: DHJPAR0046584; recordedBy: D.H. Janzen & W. Hallwachs; individualID: DHJPAR0046584; individualCount: 1; lifeStage: adult; preparations: pinned; otherCatalogNumbers: 11-SRNP-72686; **Taxon:** scientificName: Spathidexia marioburgosi; phylum: Arthropoda; class: Insecta; order: Diptera; family: Tachinidae; genus: Spathidexia; specificEpithet: marioburgosi; scientificNameAuthorship: Fleming & Wood, 2015; **Location:** continent: Central America; country: Costa Rica; countryCode: CR; stateProvince: Guanacaste; county: Area de Conservacion Guanacaste; locality: Sector Pitilla; verbatimLocality: Medrano; verbatimElevation: 380; verbatimLatitude: 11.01602; verbatimLongitude: -85.38053; verbatimCoordinateSystem: Decimal; decimalLatitude: 11.01602; decimalLongitude: -85.38053; **Identification:** identifiedBy: AJ Fleming; dateIdentified: 2014; **Event:** samplingProtocol: reared from caterpillar of *Talides* Burns02 (Hesperiidae); verbatimEventDate: 07-Dec-2011; **Record Level:** language: en; institutionCode: CNC; collectionCode: Insects; basisOfRecord: Pinned Specimen
- **Type status:** Paratype. **Occurrence:** occurrenceDetails: http://janzen.sas.upenn.edu; catalogNumber: DHJPAR0046683; recordedBy: D.H. Janzen & W. Hallwachs; individualID: DHJPAR0046683; individualCount: 1; lifeStage: adult; preparations: pinned; otherCatalogNumbers: 11-SRNP-67847; **Taxon:** scientificName: Spathidexia marioburgosi; phylum: Arthropoda; class: Insecta; order: Diptera; family: Tachinidae; genus: Spathidexia; specificEpithet: marioburgosi; scientificNameAuthorship: Fleming & Wood, 2015; **Location:** continent: Central America; country: Costa Rica; countryCode: CR; stateProvince: Alajuela; county: Area de Conservacion Guanacaste; locality: Sector Rincon Rain Forest; verbatimLocality: Palomo; verbatimElevation: 96; verbatimLatitude: 10.96187; verbatimLongitude: -85.28045; verbatimCoordinateSystem: Decimal; decimalLatitude: 10.96187; decimalLongitude: -85.28045; **Identification:** identifiedBy: AJ Fleming; dateIdentified: 2014; **Event:** samplingProtocol: reared from caterpillar of *Talides* Burns03 (Hesperiidae); verbatimEventDate: 22-Nov-2011; **Record Level:** language: en; institutionCode: CNC; collectionCode: Insects; basisOfRecord: Pinned Specimen
- **Type status:** Paratype. **Occurrence:** occurrenceDetails: http://janzen.sas.upenn.edu; catalogNumber: DHJPAR0048579; recordedBy: D.H. Janzen & W. Hallwachs; individualID: DHJPAR0048579; individualCount: 1; lifeStage: adult; preparations: pinned; otherCatalogNumbers: 12-SRNP-70340; **Taxon:** scientificName: Spathidexia marioburgosi; phylum: Arthropoda; class: Insecta; order: Diptera; family: Tachinidae; genus: Spathidexia; specificEpithet: marioburgosi; scientificNameAuthorship: Fleming & Wood, 2015; **Location:** continent: Central America; country: Costa Rica; countryCode: CR; stateProvince: Guanacaste; county: Area de Conservacion Guanacaste; locality: Sector Pitilla; verbatimLocality: Medrano; verbatimElevation: 380; verbatimLatitude: 11.01602; verbatimLongitude: -85.38053; verbatimCoordinateSystem: Decimal; decimalLatitude: 11.01602; decimalLongitude: -85.38053; **Identification:** identifiedBy: AJ Fleming; dateIdentified: 2014; **Event:** samplingProtocol: reared from caterpillar of *Talides sergestus* (Hesperiidae); verbatimEventDate: 12-Feb-2012; **Record Level:** language: en; institutionCode: CNC; collectionCode: Insects; basisOfRecord: Pinned Specimen
- **Type status:** Paratype. **Occurrence:** occurrenceDetails: http://janzen.sas.upenn.edu; catalogNumber: DHJPAR0050510; recordedBy: D.H. Janzen & W. Hallwachs; individualID: DHJPAR0050510; individualCount: 1; lifeStage: adult; preparations: pinned; otherCatalogNumbers: 12-SRNP-72629; **Taxon:** scientificName: Spathidexia marioburgosi; phylum: Arthropoda; class: Insecta; order: Diptera; family: Tachinidae; genus: Spathidexia; specificEpithet: marioburgosi; scientificNameAuthorship: Fleming & Wood, 2015; **Location:** continent: Central America; country: Costa Rica; countryCode: CR; stateProvince: Guanacaste; county: Area de Conservacion Guanacaste; locality: Sector Pitilla; verbatimLocality: Medrano; verbatimElevation: 380; verbatimLatitude: 11.01602; verbatimLongitude: -85.38053; verbatimCoordinateSystem: Decimal; decimalLatitude: 11.01602; decimalLongitude: -85.38053; **Identification:** identifiedBy: AJ Fleming; dateIdentified: 2014; **Event:** samplingProtocol: reared from caterpillar of *Talides* Burns03 (Hesperiidae); verbatimEventDate: 20-Dec-2012; **Record Level:** language: en; institutionCode: CNC; collectionCode: Insects; basisOfRecord: Pinned Specimen

#### Description

**Male** (Fig. 2a, b, c), **head**: Frontal vitta dark black, narrowed apically to less than the width of the ocellar triangle, face 6 times as wide as frontal vitta; frontal bristles arise no lower than level of pedicel; yellow hairs lining the margin of the frontal vitta; antenna orange; arista orange and plumose, trichia at least 4 times as long as width of base of arista, tapering at apex of arista; proclinate orbital bristles absent; parafrontal entirely silver with minute white hairs over its entire surface; parafacial silver; palpi orange; short row of yellow supravibrissal hairs along facial margin. **Thorax**: greyish-gold when viewed dorsally with four longitudinal grey vittae, these only slightly visible post-suturally, appearing broken at thoracic suture; three post sutural dorsocentral bristles; scutellum bearing white or yellowish pruinosity over its entirety; legs anterior femur bearing silvery pruinosity, tibiae yellow and hirsute with black tarsi. **Wings**: pale smoky yellowish in color, vein R_4+5_ setose from node up to r-m; R_1_ setose along its entire length. **Abdomen**: abdominal tergites dark shiny black medially, with bright grey band covering 1/3^rd^ or more of tergal surface arising at the margins of abdominal T4 and T5, these bands wrapping around to the underside of the abdomen. Bright yellow blotches appear along tergites T1+2, T3 and the anterior margin of T4, continuing ventrally to the tip of the abdomen; tergites T3, T4 and T5 possessing medial marginal bristles. Lateral marginal bristles on T1+2. Medium length yellow hairs visible over entire body, in particular visible extending from underside of T1+2.

**Female** (Fig. 2d, e, f), **head**: Frontal vitta dark black, narrowed apically to less than the width of the ocellar triangle, face 10 times as wide as frontal vitta; frontal bristles arise no lower than level of pedicel; yellow hairs lining the margin of the frontal vitta; antenna orange; arista orange and plumose, trichia at least 4 times as long as width of base of arista, tapering at apex of arista; proclinate orbital bristles present; parafrontal entirely silver with minute white hairs over its entire surface; parafacial silver; palpi orange; short row of yellow supravibrissal hairs along facial margin. **Thorax**: greyish-gold when viewed dorsally with four longitudinal grey vittae, these only slightly visible post-suturally, appearing broken at thoracic suture; three post sutural dorsocentral bristles; scutellum bearing white or yellowish pruinosity over its entirety; legs anterior femur bearing silvery pruinosity, tibiae yellow and hirsute with black tarsi. **Wings**: pale smoky greyish in color, vein R_4+5_ setose from node up to r-m; R_1_ setose along its entire length. **Abdomen**: abdominal tergites T_1+2_, and T_3_ dark shiny black medially, with bright grey band covering 1/3^rd^ or more of tergal surface arising at the anterior margins of abdominal tergites T4 and T5, these bands wrapping around to the underside. Bright yellow blotches appear along T1+2,T3 ending at the margin of T4, resuming along ventral surface of T5; T3, T4 and T5 possessing medial marginal bristles. Lateral marginal bristles on T1+2. Medium length yellow hairs visible over entire body, not as hirsute as males, in particular visible extending from underside of T1+2.

#### Diagnosis

Differs from *S. cerussata*, who also possesses fine white hairs on parafacialia, due to its broadly yellow side coloration, and yellow orange antenna.

#### Etymology

This species is named in honor of Sr. Mario Burgos Cespedes (RIP), whose large land holdings in Sector Pocosol became major parts of the newly forming Area de Conservacion Guanacaste in the late 1980's.

#### Distribution

Costa Rica, ACG, Prov. Alajuela and Guanacaste, rain forest and dry forest, 96–1140 m elevation.

#### Ecology

Reared 221 times from 5 species of *Talides* (Hesperiidae; Hesperiinae), all feeding on broad-leafed rain forest monocots (*Heliconia* spp., Heliconiaceae and *Musa* spp., introduced Musaceae). Usually 5–12 larvae per caterpillar. This frequently-reared species of *Spathidexia* displays a very shallow DNA barcode split into two groups (see Supplemental Appendix 1) that are identical in morphology, microgeographic location in ACG, and host records. We judge these specimens to be one species of *Spathidexia* but they may merit a subsequent deeper genetic probe.

### Spathidexia luisrobertogallegosi

Fleming & Wood 2015 sp. n.

urn:lsid:zoobank.org:act:2AC4E30F-69DD-4405-8FDA-218CBC86996D

#### Materials

- **Type status:** Holotype. **Occurrence:** occurrenceDetails: http://janzen.sas.upenn.edu; catalogNumber: DHJPAR0034394; recordedBy: D.H. Janzen & W. Hallwachs; individualID: DHJPAR0034394; individualCount: 1; sex: M; lifeStage: adult; preparations: pinned; otherCatalogNumbers: 09-SRNP-35179; **Taxon:** scientificName: Spathidexia luisrobertogallegosi; phylum: Arthropoda; class: Insecta; order: Diptera; family: Tachinidae; genus: Spathidexia; specificEpithet: luisrobertogallegosi; scientificNameAuthorship: Fleming & Wood, 2015; **Location:** continent: Central America; country: Costa Rica; countryCode: CR; stateProvince: Guanacaste; county: Area de Conservacion Guanacaste; locality: Sector Cacao; verbatimLocality: Sendero Nayo; verbatimElevation: 1090; verbatimLatitude: 10.924; verbatimLongitude: -85.47; verbatimCoordinateSystem: Decimal; decimalLatitude: 10.924; decimalLongitude: -85.47; **Identification:** identifiedBy: AJ Fleming; dateIdentified: 2014; **Event:** samplingProtocol: reared from caterpillar of *Conga chydea* (Hesperiidae); verbatimEventDate: 15-Apr-2009; **Record Level:** language: en; institutionCode: CNC; collectionCode: Insects; basisOfRecord: Pinned Specimen
- **Type status:** Paratype. **Occurrence:** occurrenceDetails: http://janzen.sas.upenn.edu; catalogNumber: DHJPAR0016675; recordedBy: D.H. Janzen & W. Hallwachs; individualID: DHJPAR0016675; individualCount: 1; lifeStage: adult; preparations: pinned; otherCatalogNumbers: 06-SRNP-47851; **Taxon:** scientificName: Spathidexia luisrobertogallegosi; phylum: Arthropoda; class: Insecta; order: Diptera; family: Tachinidae; genus: Spathidexia; specificEpithet: luisrobertogallegosi; scientificNameAuthorship: Fleming & Wood, 2015; **Location:** continent: Central America; country: Costa Rica; countryCode: CR; stateProvince: Guanacaste; county: Area de Conservacion Guanacaste; locality: Sector Cacao; verbatimLocality: Puente Gongora; verbatimElevation: 540; verbatimLatitude: 10.885; verbatimLongitude: -85.472; verbatimCoordinateSystem: Decimal; decimalLatitude: 10.885; decimalLongitude: -85.472; **Identification:** identifiedBy: AJ Fleming; dateIdentified: 2014; **Event:** samplingProtocol: reared from caterpillar of *Vehilius illudens* (Hesperiidae); verbatimEventDate: 22-Nov-2006; **Record Level:** language: en; institutionCode: CNC; collectionCode: Insects; basisOfRecord: Pinned Specimen
- **Type status:** Paratype. **Occurrence:** occurrenceDetails: http://janzen.sas.upenn.edu; catalogNumber: DHJPAR0017097; recordedBy: D.H. Janzen & W. Hallwachs; individualID: DHJPAR0017097; individualCount: 1; lifeStage: adult; preparations: pinned; otherCatalogNumbers: 07-SRNP-45135; **Taxon:** scientificName: Spathidexia luisrobertogallegosi; phylum: Arthropoda; class: Insecta; order: Diptera; family: Tachinidae; genus: Spathidexia; specificEpithet: luisrobertogallegosi; scientificNameAuthorship: Fleming & Wood, 2015; **Location:** continent: Central America; country: Costa Rica; countryCode: CR; stateProvince: Guanacaste; county: Area de Conservacion Guanacaste; locality: Sector Cacao; verbatimLocality: Puente Gongora; verbatimElevation: 540; verbatimLatitude: 10.885; verbatimLongitude: -85.472; verbatimCoordinateSystem: Decimal; decimalLatitude: 10.885; decimalLongitude: -85.472; **Identification:** identifiedBy: AJ Fleming; dateIdentified: 2014; **Event:** samplingProtocol: reared from caterpillar of *Conga chydea* (Hesperiidae); verbatimEventDate: 19-Feb-2007; **Record Level:** language: en; institutionCode: CNC; collectionCode: Insects; basisOfRecord: Pinned Specimen
- **Type status:** Paratype. **Occurrence:** occurrenceDetails: http://janzen.sas.upenn.edu; catalogNumber: DHJPAR0018737; recordedBy: D.H. Janzen & W. Hallwachs; individualID: DHJPAR0018737; individualCount: 1; lifeStage: adult; preparations: pinned; otherCatalogNumbers: 05-SRNP-20039; **Taxon:** scientificName: Spathidexia luisrobertogallegosi; phylum: Arthropoda; class: Insecta; order: Diptera; family: Tachinidae; genus: Spathidexia; specificEpithet: luisrobertogallegosi; scientificNameAuthorship: Fleming & Wood, 2015; **Location:** continent: Central America; country: Costa Rica; countryCode: CR; stateProvince: Guanacaste; county: Area de Conservacion Guanacaste; locality: Sector Del Oro; verbatimLocality: Quebrada Trigal; verbatimElevation: 290; verbatimLatitude: 11.027; verbatimLongitude: -85.495; verbatimCoordinateSystem: Decimal; decimalLatitude: 11.027; decimalLongitude: -85.495; **Identification:** identifiedBy: AJ Fleming; dateIdentified: 2014; **Event:** samplingProtocol: reared from caterpillar of *Nyctelius nyctelius* (Hesperiidae); verbatimEventDate: 07-Feb-2005; **Record Level:** language: en; institutionCode: CNC; collectionCode: Insects; basisOfRecord: Pinned Specimen
- **Type status:** Paratype. **Occurrence:** occurrenceDetails: http://janzen.sas.upenn.edu; catalogNumber: DHJPAR0018738; recordedBy: D.H. Janzen & W. Hallwachs; individualID: DHJPAR0018738; individualCount: 1; lifeStage: adult; preparations: pinned; otherCatalogNumbers: 04-SRNP-22017; **Taxon:** scientificName: Spathidexia luisrobertogallegosi; phylum: Arthropoda; class: Insecta; order: Diptera; family: Tachinidae; genus: Spathidexia; specificEpithet: luisrobertogallegosi; scientificNameAuthorship: Fleming & Wood, 2015; **Location:** continent: Central America; country: Costa Rica; countryCode: CR; stateProvince: Guanacaste; county: Area de Conservacion Guanacaste; locality: Sector Del Oro; verbatimLocality: Quebrada Raiz; verbatimElevation: 280; verbatimLatitude: 11.029; verbatimLongitude: -85.487; verbatimCoordinateSystem: Decimal; decimalLatitude: 11.029; decimalLongitude: -85.487; **Identification:** identifiedBy: AJ Fleming; dateIdentified: 2014; **Event:** samplingProtocol: reared from caterpillar of *Conga chydea* (Hesperiidae); verbatimEventDate: 07-Jun-2004; **Record Level:** language: en; institutionCode: CNC; collectionCode: Insects; basisOfRecord: Pinned Specimen
- **Type status:** Paratype. **Occurrence:** occurrenceDetails: http://janzen.sas.upenn.edu; catalogNumber: DHJPAR0018740; recordedBy: D.H. Janzen & W. Hallwachs; individualID: DHJPAR0018740; individualCount: 1; lifeStage: adult; preparations: pinned; otherCatalogNumbers: 02-SRNP-5601; **Taxon:** scientificName: Spathidexia luisrobertogallegosi; phylum: Arthropoda; class: Insecta; order: Diptera; family: Tachinidae; genus: Spathidexia; specificEpithet: luisrobertogallegosi; scientificNameAuthorship: Fleming & Wood, 2015; **Location:** continent: Central America; country: Costa Rica; countryCode: CR; stateProvince: Guanacaste; county: Area de Conservacion Guanacaste; locality: Sector Del Oro; verbatimLocality: Canyon Rio Mena; verbatimElevation: 560; verbatimLatitude: 10.996; verbatimLongitude: -85.456; verbatimCoordinateSystem: Decimal; decimalLatitude: 10.996; decimalLongitude: -85.456; **Identification:** identifiedBy: AJ Fleming; dateIdentified: 2014; **Event:** samplingProtocol: reared from caterpillar of *Conga chydea* (Hesperiidae); verbatimEventDate: 08-Mar-2002; **Record Level:** language: en; institutionCode: CNC; collectionCode: Insects; basisOfRecord: Pinned Specimen
- **Type status:** Paratype. **Occurrence:** occurrenceDetails: http://janzen.sas.upenn.edu; catalogNumber: DHJPAR0019615; recordedBy: D.H. Janzen & W. Hallwachs; individualID: DHJPAR0019615; individualCount: 1; lifeStage: adult; preparations: pinned; otherCatalogNumbers: 07-SRNP-35371; **Taxon:** scientificName: Spathidexia luisrobertogallegosi; phylum: Arthropoda; class: Insecta; order: Diptera; family: Tachinidae; genus: Spathidexia; specificEpithet: luisrobertogallegosi; scientificNameAuthorship: Fleming & Wood, 2015; **Location:** continent: Central America; country: Costa Rica; countryCode: CR; stateProvince: Guanacaste; county: Area de Conservacion Guanacaste; locality: Sector Cacao; verbatimLocality: Sendero Nayo; verbatimElevation: 1090; verbatimLatitude: 10.924; verbatimLongitude: -85.47; verbatimCoordinateSystem: Decimal; decimalLatitude: 10.924; decimalLongitude: -85.47; **Identification:** identifiedBy: AJ Fleming; dateIdentified: 2014; **Event:** samplingProtocol: reared from caterpillar of *Conga chydea* (Hesperiidae); verbatimEventDate: 20-Apr-2007; **Record Level:** language: en; institutionCode: CNC; collectionCode: Insects; basisOfRecord: Pinned Specimen
- **Type status:** Paratype. **Occurrence:** occurrenceDetails: http://janzen.sas.upenn.edu; catalogNumber: DHJPAR0019623; recordedBy: D.H. Janzen & W. Hallwachs; individualID: DHJPAR0019623; individualCount: 1; lifeStage: adult; preparations: pinned; otherCatalogNumbers: 07-SRNP-35277; **Taxon:** scientificName: Spathidexia luisrobertogallegosi; phylum: Arthropoda; class: Insecta; order: Diptera; family: Tachinidae; genus: Spathidexia; specificEpithet: luisrobertogallegosi; scientificNameAuthorship: Fleming & Wood, 2015; **Location:** continent: Central America; country: Costa Rica; countryCode: CR; stateProvince: Guanacaste; county: Area de Conservacion Guanacaste; locality: Sector Cacao; verbatimLocality: Sendero Arenales; verbatimElevation: 1080; verbatimLatitude: 10.925; verbatimLongitude: -85.467; verbatimCoordinateSystem: Decimal; decimalLatitude: 10.925; decimalLongitude: -85.467; **Identification:** identifiedBy: AJ Fleming; dateIdentified: 2014; **Event:** samplingProtocol: reared from caterpillar of *Conga chydea* (Hesperiidae); verbatimEventDate: 12-Apr-2007; **Record Level:** language: en; institutionCode: CNC; collectionCode: Insects; basisOfRecord: Pinned Specimen
- **Type status:** Paratype. **Occurrence:** occurrenceDetails: http://janzen.sas.upenn.edu; catalogNumber: DHJPAR0019633; recordedBy: D.H. Janzen & W. Hallwachs; individualID: DHJPAR0019633; individualCount: 1; lifeStage: adult; preparations: pinned; otherCatalogNumbers: 07-SRNP-35362; **Taxon:** scientificName: Spathidexia luisrobertogallegosi; phylum: Arthropoda; class: Insecta; order: Diptera; family: Tachinidae; genus: Spathidexia; specificEpithet: luisrobertogallegosi; scientificNameAuthorship: Fleming & Wood, 2015; **Location:** continent: Central America; country: Costa Rica; countryCode: CR; stateProvince: Guanacaste; county: Area de Conservacion Guanacaste; locality: Sector Cacao; verbatimLocality: Sendero Nayo; verbatimElevation: 1090; verbatimLatitude: 10.924; verbatimLongitude: -85.47; verbatimCoordinateSystem: Decimal; decimalLatitude: 10.924; decimalLongitude: -85.47; **Identification:** identifiedBy: AJ Fleming; dateIdentified: 2014; **Event:** samplingProtocol: reared from caterpillar of *Conga chydea* (Hesperiidae); verbatimEventDate: 12-Apr-2007; **Record Level:** language: en; institutionCode: CNC; collectionCode: Insects; basisOfRecord: Pinned Specimen
- **Type status:** Paratype. **Occurrence:** occurrenceDetails: http://janzen.sas.upenn.edu; catalogNumber: DHJPAR0019648; recordedBy: D.H. Janzen & W. Hallwachs; individualID: DHJPAR0019648; individualCount: 1; lifeStage: adult; preparations: pinned; otherCatalogNumbers: 07-SRNP-35646; **Taxon:** scientificName: Spathidexia luisrobertogallegosi; phylum: Arthropoda; class: Insecta; order: Diptera; family: Tachinidae; genus: Spathidexia; specificEpithet: luisrobertogallegosi; scientificNameAuthorship: Fleming & Wood, 2015; **Location:** continent: Central America; country: Costa Rica; countryCode: CR; stateProvince: Guanacaste; county: Area de Conservacion Guanacaste; locality: Sector Cacao; verbatimLocality: Estacion Cacao; verbatimElevation: 1150; verbatimLatitude: 10.927; verbatimLongitude: -85.468; verbatimCoordinateSystem: Decimal; decimalLatitude: 10.927; decimalLongitude: -85.468; **Identification:** identifiedBy: AJ Fleming; dateIdentified: 2014; **Event:** samplingProtocol: reared from caterpillar of *Conga chydea* (Hesperiidae); verbatimEventDate: 14-May-2007; **Record Level:** language: en; institutionCode: CNC; collectionCode: Insects; basisOfRecord: Pinned Specimen
- **Type status:** Paratype. **Occurrence:** occurrenceDetails: http://janzen.sas.upenn.edu; catalogNumber: DHJPAR0019649; recordedBy: D.H. Janzen & W. Hallwachs; individualID: DHJPAR0019649; individualCount: 1; lifeStage: adult; preparations: pinned; otherCatalogNumbers: 07-SRNP-35454; **Taxon:** scientificName: Spathidexia luisrobertogallegosi; phylum: Arthropoda; class: Insecta; order: Diptera; family: Tachinidae; genus: Spathidexia; specificEpithet: luisrobertogallegosi; scientificNameAuthorship: Fleming & Wood, 2015; **Location:** continent: Central America; country: Costa Rica; countryCode: CR; stateProvince: Guanacaste; county: Area de Conservacion Guanacaste; locality: Sector Cacao; verbatimLocality: Sendero Nayo; verbatimElevation: 1090; verbatimLatitude: 10.924; verbatimLongitude: -85.47; verbatimCoordinateSystem: Decimal; decimalLatitude: 10.924; decimalLongitude: -85.47; **Identification:** identifiedBy: AJ Fleming; dateIdentified: 2014; **Event:** samplingProtocol: reared from caterpillar of *Conga chydea* (Hesperiidae); verbatimEventDate: 05-May-2007; **Record Level:** language: en; institutionCode: CNC; collectionCode: Insects; basisOfRecord: Pinned Specimen
- **Type status:** Paratype. **Occurrence:** occurrenceDetails: http://janzen.sas.upenn.edu; catalogNumber: DHJPAR0019650; recordedBy: D.H. Janzen & W. Hallwachs; individualID: DHJPAR0019650; individualCount: 1; lifeStage: adult; preparations: pinned; otherCatalogNumbers: 07-SRNP-35357; **Taxon:** scientificName: Spathidexia luisrobertogallegosi; phylum: Arthropoda; class: Insecta; order: Diptera; family: Tachinidae; genus: Spathidexia; specificEpithet: luisrobertogallegosi; scientificNameAuthorship: Fleming & Wood, 2015; **Location:** continent: Central America; country: Costa Rica; countryCode: CR; stateProvince: Guanacaste; county: Area de Conservacion Guanacaste; locality: Sector Cacao; verbatimLocality: Sendero Nayo; verbatimElevation: 1090; verbatimLatitude: 10.924; verbatimLongitude: -85.47; verbatimCoordinateSystem: Decimal; decimalLatitude: 10.924; decimalLongitude: -85.47; **Identification:** identifiedBy: AJ Fleming; dateIdentified: 2014; **Event:** samplingProtocol: reared from caterpillar of *Conga chydea* (Hesperiidae); verbatimEventDate: 01-May-2007; **Record Level:** language: en; institutionCode: CNC; collectionCode: Insects; basisOfRecord: Pinned Specimen
- **Type status:** Paratype. **Occurrence:** occurrenceDetails: http://janzen.sas.upenn.edu; catalogNumber: DHJPAR0019651; recordedBy: D.H. Janzen & W. Hallwachs; individualID: DHJPAR0019651; individualCount: 1; lifeStage: adult; preparations: pinned; otherCatalogNumbers: 07-SRNP-35243; **Taxon:** scientificName: Spathidexia luisrobertogallegosi; phylum: Arthropoda; class: Insecta; order: Diptera; family: Tachinidae; genus: Spathidexia; specificEpithet: luisrobertogallegosi; scientificNameAuthorship: Fleming & Wood, 2015; **Location:** continent: Central America; country: Costa Rica; countryCode: CR; stateProvince: Guanacaste; county: Area de Conservacion Guanacaste; locality: Sector Cacao; verbatimLocality: Sendero Nayo; verbatimElevation: 1090; verbatimLatitude: 10.924; verbatimLongitude: -85.47; verbatimCoordinateSystem: Decimal; decimalLatitude: 10.924; decimalLongitude: -85.47; **Identification:** identifiedBy: AJ Fleming; dateIdentified: 2014; **Event:** samplingProtocol: reared from caterpillar of *Conga chydea* (Hesperiidae); verbatimEventDate: 03-Apr-2007; **Record Level:** language: en; institutionCode: CNC; collectionCode: Insects; basisOfRecord: Pinned Specimen
- **Type status:** Paratype. **Occurrence:** occurrenceDetails: http://janzen.sas.upenn.edu; catalogNumber: DHJPAR0019652; recordedBy: D.H. Janzen & W. Hallwachs; individualID: DHJPAR0019652; individualCount: 1; lifeStage: adult; preparations: pinned; otherCatalogNumbers: 07-SRNP-35297; **Taxon:** scientificName: Spathidexia luisrobertogallegosi; phylum: Arthropoda; class: Insecta; order: Diptera; family: Tachinidae; genus: Spathidexia; specificEpithet: luisrobertogallegosi; scientificNameAuthorship: Fleming & Wood, 2015; **Location:** continent: Central America; country: Costa Rica; countryCode: CR; stateProvince: Guanacaste; county: Area de Conservacion Guanacaste; locality: Sector Cacao; verbatimLocality: Cerro Pedregal; verbatimElevation: 1080; verbatimLatitude: 10.928; verbatimLongitude: -85.474; verbatimCoordinateSystem: Decimal; decimalLatitude: 10.928; decimalLongitude: -85.474; **Identification:** identifiedBy: AJ Fleming; dateIdentified: 2014; **Event:** samplingProtocol: reared from caterpillar of *Conga chydea* (Hesperiidae); verbatimEventDate: 14-Apr-2007; **Record Level:** language: en; institutionCode: CNC; collectionCode: Insects; basisOfRecord: Pinned Specimen
- **Type status:** Paratype. **Occurrence:** occurrenceDetails: http://janzen.sas.upenn.edu; catalogNumber: DHJPAR0019653; recordedBy: D.H. Janzen & W. Hallwachs; individualID: DHJPAR0019653; individualCount: 1; lifeStage: adult; preparations: pinned; otherCatalogNumbers: 07-SRNP-35367; **Taxon:** scientificName: Spathidexia luisrobertogallegosi; phylum: Arthropoda; class: Insecta; order: Diptera; family: Tachinidae; genus: Spathidexia; specificEpithet: luisrobertogallegosi; scientificNameAuthorship: Fleming & Wood, 2015; **Location:** continent: Central America; country: Costa Rica; countryCode: CR; stateProvince: Guanacaste; county: Area de Conservacion Guanacaste; locality: Sector Cacao; verbatimLocality: Sendero Nayo; verbatimElevation: 1090; verbatimLatitude: 10.924; verbatimLongitude: -85.47; verbatimCoordinateSystem: Decimal; decimalLatitude: 10.924; decimalLongitude: -85.47; **Identification:** identifiedBy: AJ Fleming; dateIdentified: 2014; **Event:** samplingProtocol: reared from caterpillar of *Conga chydea* (Hesperiidae); verbatimEventDate: 18-Apr-2007; **Record Level:** language: en; institutionCode: CNC; collectionCode: Insects; basisOfRecord: Pinned Specimen
- **Type status:** Paratype. **Occurrence:** occurrenceDetails: http://janzen.sas.upenn.edu; catalogNumber: DHJPAR0019654; recordedBy: D.H. Janzen & W. Hallwachs; individualID: DHJPAR0019654; individualCount: 1; lifeStage: adult; preparations: pinned; otherCatalogNumbers: 07-SRNP-35729; **Taxon:** scientificName: Spathidexia luisrobertogallegosi; phylum: Arthropoda; class: Insecta; order: Diptera; family: Tachinidae; genus: Spathidexia; specificEpithet: luisrobertogallegosi; scientificNameAuthorship: Fleming & Wood, 2015; **Location:** continent: Central America; country: Costa Rica; countryCode: CR; stateProvince: Guanacaste; county: Area de Conservacion Guanacaste; locality: Sector Cacao; verbatimLocality: Sendero Cima; verbatimElevation: 1460; verbatimLatitude: 10.933; verbatimLongitude: -85.457; verbatimCoordinateSystem: Decimal; decimalLatitude: 10.933; decimalLongitude: -85.457; **Identification:** identifiedBy: AJ Fleming; dateIdentified: 2014; **Event:** samplingProtocol: reared from caterpillar of hespJanzen01 Janzen38 (Hesperiidae); verbatimEventDate: 30-May-2007; **Record Level:** language: en; institutionCode: CNC; collectionCode: Insects; basisOfRecord: Pinned Specimen
- **Type status:** Paratype. **Occurrence:** occurrenceDetails: http://janzen.sas.upenn.edu; catalogNumber: DHJPAR0019655; recordedBy: D.H. Janzen & W. Hallwachs; individualID: DHJPAR0019655; individualCount: 1; lifeStage: adult; preparations: pinned; otherCatalogNumbers: 07-SRNP-35375; **Taxon:** scientificName: Spathidexia luisrobertogallegosi; phylum: Arthropoda; class: Insecta; order: Diptera; family: Tachinidae; genus: Spathidexia; specificEpithet: luisrobertogallegosi; scientificNameAuthorship: Fleming & Wood, 2015; **Location:** continent: Central America; country: Costa Rica; countryCode: CR; stateProvince: Guanacaste; county: Area de Conservacion Guanacaste; locality: Sector Cacao; verbatimLocality: Sendero Nayo; verbatimElevation: 1090; verbatimLatitude: 10.924; verbatimLongitude: -85.47; verbatimCoordinateSystem: Decimal; decimalLatitude: 10.924; decimalLongitude: -85.47; **Identification:** identifiedBy: AJ Fleming; dateIdentified: 2014; **Event:** samplingProtocol: reared from caterpillar of *Conga chydea* (Hesperiidae); verbatimEventDate: 09-Apr-2007; **Record Level:** language: en; institutionCode: CNC; collectionCode: Insects; basisOfRecord: Pinned Specimen
- **Type status:** Paratype. **Occurrence:** occurrenceDetails: http://janzen.sas.upenn.edu; catalogNumber: DHJPAR0019656; recordedBy: D.H. Janzen & W. Hallwachs; individualID: DHJPAR0019656; individualCount: 1; lifeStage: adult; preparations: pinned; otherCatalogNumbers: 07-SRNP-35335; **Taxon:** scientificName: Spathidexia luisrobertogallegosi; phylum: Arthropoda; class: Insecta; order: Diptera; family: Tachinidae; genus: Spathidexia; specificEpithet: luisrobertogallegosi; scientificNameAuthorship: Fleming & Wood, 2015; **Location:** continent: Central America; country: Costa Rica; countryCode: CR; stateProvince: Guanacaste; county: Area de Conservacion Guanacaste; locality: Sector Cacao; verbatimLocality: Sendero Arenales; verbatimElevation: 1080; verbatimLatitude: 10.925; verbatimLongitude: -85.467; verbatimCoordinateSystem: Decimal; decimalLatitude: 10.925; decimalLongitude: -85.467; **Identification:** identifiedBy: AJ Fleming; dateIdentified: 2014; **Event:** samplingProtocol: reared from caterpillar of *Conga chydea* (Hesperiidae); verbatimEventDate: 14-Apr-2007; **Record Level:** language: en; institutionCode: CNC; collectionCode: Insects; basisOfRecord: Pinned Specimen
- **Type status:** Paratype. **Occurrence:** occurrenceDetails: http://janzen.sas.upenn.edu; catalogNumber: DHJPAR0019657; recordedBy: D.H. Janzen & W. Hallwachs; individualID: DHJPAR0019657; individualCount: 1; lifeStage: adult; preparations: pinned; otherCatalogNumbers: 07-SRNP-35369; **Taxon:** scientificName: Spathidexia luisrobertogallegosi; phylum: Arthropoda; class: Insecta; order: Diptera; family: Tachinidae; genus: Spathidexia; specificEpithet: luisrobertogallegosi; scientificNameAuthorship: Fleming & Wood, 2015; **Location:** continent: Central America; country: Costa Rica; countryCode: CR; stateProvince: Guanacaste; county: Area de Conservacion Guanacaste; locality: Sector Cacao; verbatimLocality: Sendero Nayo; verbatimElevation: 1090; verbatimLatitude: 10.924; verbatimLongitude: -85.47; verbatimCoordinateSystem: Decimal; decimalLatitude: 10.924; decimalLongitude: -85.47; **Identification:** identifiedBy: AJ Fleming; dateIdentified: 2014; **Event:** samplingProtocol: reared from caterpillar of *Conga chydea* (Hesperiidae); verbatimEventDate: 20-Apr-2007; **Record Level:** language: en; institutionCode: CNC; collectionCode: Insects; basisOfRecord: Pinned Specimen
- **Type status:** Paratype. **Occurrence:** occurrenceDetails: http://janzen.sas.upenn.edu; catalogNumber: DHJPAR0019659; recordedBy: D.H. Janzen & W. Hallwachs; individualID: DHJPAR0019659; individualCount: 1; lifeStage: adult; preparations: pinned; otherCatalogNumbers: 07-SRNP-35298; **Taxon:** scientificName: Spathidexia luisrobertogallegosi; phylum: Arthropoda; class: Insecta; order: Diptera; family: Tachinidae; genus: Spathidexia; specificEpithet: luisrobertogallegosi; scientificNameAuthorship: Fleming & Wood, 2015; **Location:** continent: Central America; country: Costa Rica; countryCode: CR; stateProvince: Guanacaste; county: Area de Conservacion Guanacaste; locality: Sector Cacao; verbatimLocality: Cerro Pedregal; verbatimElevation: 1080; verbatimLatitude: 10.928; verbatimLongitude: -85.474; verbatimCoordinateSystem: Decimal; decimalLatitude: 10.928; decimalLongitude: -85.474; **Identification:** identifiedBy: AJ Fleming; dateIdentified: 2014; **Event:** samplingProtocol: reared from caterpillar of *Conga chydea* (Hesperiidae); verbatimEventDate: 18-Apr-2007; **Record Level:** language: en; institutionCode: CNC; collectionCode: Insects; basisOfRecord: Pinned Specimen
- **Type status:** Paratype. **Occurrence:** occurrenceDetails: http://janzen.sas.upenn.edu; catalogNumber: DHJPAR0019660; recordedBy: D.H. Janzen & W. Hallwachs; individualID: DHJPAR0019660; individualCount: 1; lifeStage: adult; preparations: pinned; otherCatalogNumbers: 07-SRNP-35372; **Taxon:** scientificName: Spathidexia luisrobertogallegosi; phylum: Arthropoda; class: Insecta; order: Diptera; family: Tachinidae; genus: Spathidexia; specificEpithet: luisrobertogallegosi; scientificNameAuthorship: Fleming & Wood, 2015; **Location:** continent: Central America; country: Costa Rica; countryCode: CR; stateProvince: Guanacaste; county: Area de Conservacion Guanacaste; locality: Sector Cacao; verbatimLocality: Sendero Nayo; verbatimElevation: 1090; verbatimLatitude: 10.924; verbatimLongitude: -85.47; verbatimCoordinateSystem: Decimal; decimalLatitude: 10.924; decimalLongitude: -85.47; **Identification:** identifiedBy: AJ Fleming; dateIdentified: 2014; **Event:** samplingProtocol: reared from caterpillar of *Conga chydea* (Hesperiidae); verbatimEventDate: 23-Apr-2007; **Record Level:** language: en; institutionCode: CNC; collectionCode: Insects; basisOfRecord: Pinned Specimen
- **Type status:** Paratype. **Occurrence:** occurrenceDetails: http://janzen.sas.upenn.edu; catalogNumber: DHJPAR0034378; recordedBy: D.H. Janzen & W. Hallwachs; individualID: DHJPAR0034378; individualCount: 1; lifeStage: adult; preparations: pinned; otherCatalogNumbers: 09-SRNP-35196; **Taxon:** scientificName: Spathidexia luisrobertogallegosi; phylum: Arthropoda; class: Insecta; order: Diptera; family: Tachinidae; genus: Spathidexia; specificEpithet: luisrobertogallegosi; scientificNameAuthorship: Fleming & Wood, 2015; **Location:** continent: Central America; country: Costa Rica; countryCode: CR; stateProvince: Guanacaste; county: Area de Conservacion Guanacaste; locality: Sector Cacao; verbatimLocality: Sendero Nayo; verbatimElevation: 1090; verbatimLatitude: 10.924; verbatimLongitude: -85.47; verbatimCoordinateSystem: Decimal; decimalLatitude: 10.924; decimalLongitude: -85.47; **Identification:** identifiedBy: AJ Fleming; dateIdentified: 2014; **Event:** samplingProtocol: reared from caterpillar of *Conga chydea* (Hesperiidae); verbatimEventDate: 05-May-2009; **Record Level:** language: en; institutionCode: CNC; collectionCode: Insects; basisOfRecord: Pinned Specimen
- **Type status:** Paratype. **Occurrence:** occurrenceDetails: http://janzen.sas.upenn.edu; catalogNumber: DHJPAR0034380; recordedBy: D.H. Janzen & W. Hallwachs; individualID: DHJPAR0034380; individualCount: 1; lifeStage: adult; preparations: pinned; otherCatalogNumbers: 09-SRNP-35197; **Taxon:** scientificName: Spathidexia luisrobertogallegosi; phylum: Arthropoda; class: Insecta; order: Diptera; family: Tachinidae; genus: Spathidexia; specificEpithet: luisrobertogallegosi; scientificNameAuthorship: Fleming & Wood, 2015; **Location:** continent: Central America; country: Costa Rica; countryCode: CR; stateProvince: Guanacaste; county: Area de Conservacion Guanacaste; locality: Sector Cacao; verbatimLocality: Sendero Nayo; verbatimElevation: 1090; verbatimLatitude: 10.924; verbatimLongitude: -85.47; verbatimCoordinateSystem: Decimal; decimalLatitude: 10.924; decimalLongitude: -85.47; **Identification:** identifiedBy: AJ Fleming; dateIdentified: 2014; **Event:** samplingProtocol: reared from caterpillar of *Conga chydea* (Hesperiidae); verbatimEventDate: 18-Apr-2009; **Record Level:** language: en; institutionCode: CNC; collectionCode: Insects; basisOfRecord: Pinned Specimen
- **Type status:** Paratype. **Occurrence:** occurrenceDetails: http://janzen.sas.upenn.edu; catalogNumber: DHJPAR0034381; recordedBy: D.H. Janzen & W. Hallwachs; individualID: DHJPAR0034381; individualCount: 1; lifeStage: adult; preparations: pinned; otherCatalogNumbers: 09-SRNP-35184; **Taxon:** scientificName: Spathidexia luisrobertogallegosi; phylum: Arthropoda; class: Insecta; order: Diptera; family: Tachinidae; genus: Spathidexia; specificEpithet: luisrobertogallegosi; scientificNameAuthorship: Fleming & Wood, 2015; **Location:** continent: Central America; country: Costa Rica; countryCode: CR; stateProvince: Guanacaste; county: Area de Conservacion Guanacaste; locality: Sector Cacao; verbatimLocality: Sendero Nayo; verbatimElevation: 1090; verbatimLatitude: 10.924; verbatimLongitude: -85.47; verbatimCoordinateSystem: Decimal; decimalLatitude: 10.924; decimalLongitude: -85.47; **Identification:** identifiedBy: AJ Fleming; dateIdentified: 2014; **Event:** samplingProtocol: reared from caterpillar of *Conga chydea* (Hesperiidae); verbatimEventDate: 20-Apr-2009; **Record Level:** language: en; institutionCode: CNC; collectionCode: Insects; basisOfRecord: Pinned Specimen
- **Type status:** Paratype. **Occurrence:** occurrenceDetails: http://janzen.sas.upenn.edu; catalogNumber: DHJPAR0034382; recordedBy: D.H. Janzen & W. Hallwachs; individualID: DHJPAR0034382; individualCount: 1; lifeStage: adult; preparations: pinned; otherCatalogNumbers: 09-SRNP-35251; **Taxon:** scientificName: Spathidexia luisrobertogallegosi; phylum: Arthropoda; class: Insecta; order: Diptera; family: Tachinidae; genus: Spathidexia; specificEpithet: luisrobertogallegosi; scientificNameAuthorship: Fleming & Wood, 2015; **Location:** continent: Central America; country: Costa Rica; countryCode: CR; stateProvince: Guanacaste; county: Area de Conservacion Guanacaste; locality: Sector Cacao; verbatimLocality: Sendero Arenales; verbatimElevation: 1080; verbatimLatitude: 10.925; verbatimLongitude: -85.467; verbatimCoordinateSystem: Decimal; decimalLatitude: 10.925; decimalLongitude: -85.467; **Identification:** identifiedBy: AJ Fleming; dateIdentified: 2014; **Event:** samplingProtocol: reared from caterpillar of *Conga chydea* (Hesperiidae); verbatimEventDate: 27-Sep-2009; **Record Level:** language: en; institutionCode: CNC; collectionCode: Insects; basisOfRecord: Pinned Specimen
- **Type status:** Paratype. **Occurrence:** occurrenceDetails: http://janzen.sas.upenn.edu; catalogNumber: DHJPAR0034383; recordedBy: D.H. Janzen & W. Hallwachs; individualID: DHJPAR0034383; individualCount: 1; lifeStage: adult; preparations: pinned; otherCatalogNumbers: 09-SRNP-35178; **Taxon:** scientificName: Spathidexia luisrobertogallegosi; phylum: Arthropoda; class: Insecta; order: Diptera; family: Tachinidae; genus: Spathidexia; specificEpithet: luisrobertogallegosi; scientificNameAuthorship: Fleming & Wood, 2015; **Location:** continent: Central America; country: Costa Rica; countryCode: CR; stateProvince: Guanacaste; county: Area de Conservacion Guanacaste; locality: Sector Cacao; verbatimLocality: Sendero Nayo; verbatimElevation: 1090; verbatimLatitude: 10.924; verbatimLongitude: -85.47; verbatimCoordinateSystem: Decimal; decimalLatitude: 10.924; decimalLongitude: -85.47; **Identification:** identifiedBy: AJ Fleming; dateIdentified: 2014; **Event:** samplingProtocol: reared from caterpillar of *Conga chydea* (Hesperiidae); verbatimEventDate: 15-Apr-2009; **Record Level:** language: en; institutionCode: CNC; collectionCode: Insects; basisOfRecord: Pinned Specimen
- **Type status:** Paratype. **Occurrence:** occurrenceDetails: http://janzen.sas.upenn.edu; catalogNumber: DHJPAR0034384; recordedBy: D.H. Janzen & W. Hallwachs; individualID: DHJPAR0034384; individualCount: 1; lifeStage: adult; preparations: pinned; otherCatalogNumbers: 09-SRNP-35182; **Taxon:** scientificName: Spathidexia luisrobertogallegosi; phylum: Arthropoda; class: Insecta; order: Diptera; family: Tachinidae; genus: Spathidexia; specificEpithet: luisrobertogallegosi; scientificNameAuthorship: Fleming & Wood, 2015; **Location:** continent: Central America; country: Costa Rica; countryCode: CR; stateProvince: Guanacaste; county: Area de Conservacion Guanacaste; locality: Sector Cacao; verbatimLocality: Sendero Nayo; verbatimElevation: 1090; verbatimLatitude: 10.924; verbatimLongitude: -85.47; verbatimCoordinateSystem: Decimal; decimalLatitude: 10.924; decimalLongitude: -85.47; **Identification:** identifiedBy: AJ Fleming; dateIdentified: 2014; **Event:** samplingProtocol: reared from caterpillar of *Conga chydea* (Hesperiidae); verbatimEventDate: 24-Apr-2009; **Record Level:** language: en; institutionCode: CNC; collectionCode: Insects; basisOfRecord: Pinned Specimen
- **Type status:** Paratype. **Occurrence:** occurrenceDetails: http://janzen.sas.upenn.edu; catalogNumber: DHJPAR0034385; recordedBy: D.H. Janzen & W. Hallwachs; individualID: DHJPAR0034385; individualCount: 1; lifeStage: adult; preparations: pinned; otherCatalogNumbers: 09-SRNP-35190; **Taxon:** scientificName: Spathidexia luisrobertogallegosi; phylum: Arthropoda; class: Insecta; order: Diptera; family: Tachinidae; genus: Spathidexia; specificEpithet: luisrobertogallegosi; scientificNameAuthorship: Fleming & Wood, 2015; **Location:** continent: Central America; country: Costa Rica; countryCode: CR; stateProvince: Guanacaste; county: Area de Conservacion Guanacaste; locality: Sector Cacao; verbatimLocality: Sendero Nayo; verbatimElevation: 1090; verbatimLatitude: 10.924; verbatimLongitude: -85.47; verbatimCoordinateSystem: Decimal; decimalLatitude: 10.924; decimalLongitude: -85.47; **Identification:** identifiedBy: AJ Fleming; dateIdentified: 2014; **Event:** samplingProtocol: reared from caterpillar of *Conga chydea* (Hesperiidae); verbatimEventDate: 20-Apr-2009; **Record Level:** language: en; institutionCode: CNC; collectionCode: Insects; basisOfRecord: Pinned Specimen
- **Type status:** Paratype. **Occurrence:** occurrenceDetails: http://janzen.sas.upenn.edu; catalogNumber: DHJPAR0034386; recordedBy: D.H. Janzen & W. Hallwachs; individualID: DHJPAR0034386; individualCount: 1; lifeStage: adult; preparations: pinned; otherCatalogNumbers: 09-SRNP-35183; **Taxon:** scientificName: Spathidexia luisrobertogallegosi; phylum: Arthropoda; class: Insecta; order: Diptera; family: Tachinidae; genus: Spathidexia; specificEpithet: luisrobertogallegosi; scientificNameAuthorship: Fleming & Wood, 2015; **Location:** continent: Central America; country: Costa Rica; countryCode: CR; stateProvince: Guanacaste; county: Area de Conservacion Guanacaste; locality: Sector Cacao; verbatimLocality: Sendero Nayo; verbatimElevation: 1090; verbatimLatitude: 10.924; verbatimLongitude: -85.47; verbatimCoordinateSystem: Decimal; decimalLatitude: 10.924; decimalLongitude: -85.47; **Identification:** identifiedBy: AJ Fleming; dateIdentified: 2014; **Event:** samplingProtocol: reared from caterpillar of *Conga chydea* (Hesperiidae); verbatimEventDate: 27-Sep-2009; **Record Level:** language: en; institutionCode: CNC; collectionCode: Insects; basisOfRecord: Pinned Specimen
- **Type status:** Paratype. **Occurrence:** occurrenceDetails: http://janzen.sas.upenn.edu; catalogNumber: DHJPAR0034388; recordedBy: D.H. Janzen & W. Hallwachs; individualID: DHJPAR0034388; individualCount: 1; lifeStage: adult; preparations: pinned; otherCatalogNumbers: 09-SRNP-35198; **Taxon:** scientificName: Spathidexia luisrobertogallegosi; phylum: Arthropoda; class: Insecta; order: Diptera; family: Tachinidae; genus: Spathidexia; specificEpithet: luisrobertogallegosi; scientificNameAuthorship: Fleming & Wood, 2015; **Location:** continent: Central America; country: Costa Rica; countryCode: CR; stateProvince: Guanacaste; county: Area de Conservacion Guanacaste; locality: Sector Cacao; verbatimLocality: Sendero Nayo; verbatimElevation: 1090; verbatimLatitude: 10.924; verbatimLongitude: -85.47; verbatimCoordinateSystem: Decimal; decimalLatitude: 10.924; decimalLongitude: -85.47; **Identification:** identifiedBy: AJ Fleming; dateIdentified: 2014; **Event:** samplingProtocol: reared from caterpillar of *Conga chydea* (Hesperiidae); verbatimEventDate: 27-Sep-2009; **Record Level:** language: en; institutionCode: CNC; collectionCode: Insects; basisOfRecord: Pinned Specimen
- **Type status:** Paratype. **Occurrence:** occurrenceDetails: http://janzen.sas.upenn.edu; catalogNumber: DHJPAR0034389; recordedBy: D.H. Janzen & W. Hallwachs; individualID: DHJPAR0034389; individualCount: 1; lifeStage: adult; preparations: pinned; otherCatalogNumbers: 09-SRNP-35187; **Taxon:** scientificName: Spathidexia luisrobertogallegosi; phylum: Arthropoda; class: Insecta; order: Diptera; family: Tachinidae; genus: Spathidexia; specificEpithet: luisrobertogallegosi; scientificNameAuthorship: Fleming & Wood, 2015; **Location:** continent: Central America; country: Costa Rica; countryCode: CR; stateProvince: Guanacaste; county: Area de Conservacion Guanacaste; locality: Sector Cacao; verbatimLocality: Sendero Nayo; verbatimElevation: 1090; verbatimLatitude: 10.924; verbatimLongitude: -85.47; verbatimCoordinateSystem: Decimal; decimalLatitude: 10.924; decimalLongitude: -85.47; **Identification:** identifiedBy: AJ Fleming; dateIdentified: 2014; **Event:** samplingProtocol: reared from caterpillar of *Conga chydea* (Hesperiidae); verbatimEventDate: 18-Apr-2009; **Record Level:** language: en; institutionCode: CNC; collectionCode: Insects; basisOfRecord: Pinned Specimen
- **Type status:** Paratype. **Occurrence:** occurrenceDetails: http://janzen.sas.upenn.edu; catalogNumber: DHJPAR0034390; recordedBy: D.H. Janzen & W. Hallwachs; individualID: DHJPAR0034390; individualCount: 1; lifeStage: adult; preparations: pinned; otherCatalogNumbers: 09-SRNP-35194; **Taxon:** scientificName: Spathidexia luisrobertogallegosi; phylum: Arthropoda; class: Insecta; order: Diptera; family: Tachinidae; genus: Spathidexia; specificEpithet: luisrobertogallegosi; scientificNameAuthorship: Fleming & Wood, 2015; **Location:** continent: Central America; country: Costa Rica; countryCode: CR; stateProvince: Guanacaste; county: Area de Conservacion Guanacaste; locality: Sector Cacao; verbatimLocality: Sendero Nayo; verbatimElevation: 1090; verbatimLatitude: 10.924; verbatimLongitude: -85.47; verbatimCoordinateSystem: Decimal; decimalLatitude: 10.924; decimalLongitude: -85.47; **Identification:** identifiedBy: AJ Fleming; dateIdentified: 2014; **Event:** samplingProtocol: reared from caterpillar of *Conga chydea* (Hesperiidae); verbatimEventDate: 23-Apr-2009; **Record Level:** language: en; institutionCode: CNC; collectionCode: Insects; basisOfRecord: Pinned Specimen
- **Type status:** Paratype. **Occurrence:** occurrenceDetails: http://janzen.sas.upenn.edu; catalogNumber: DHJPAR0034391; recordedBy: D.H. Janzen & W. Hallwachs; individualID: DHJPAR0034391; individualCount: 1; lifeStage: adult; preparations: pinned; otherCatalogNumbers: 09-SRNP-35189; **Taxon:** scientificName: Spathidexia luisrobertogallegosi; phylum: Arthropoda; class: Insecta; order: Diptera; family: Tachinidae; genus: Spathidexia; specificEpithet: luisrobertogallegosi; scientificNameAuthorship: Fleming & Wood, 2015; **Location:** continent: Central America; country: Costa Rica; countryCode: CR; stateProvince: Guanacaste; county: Area de Conservacion Guanacaste; locality: Sector Cacao; verbatimLocality: Sendero Nayo; verbatimElevation: 1090; verbatimLatitude: 10.924; verbatimLongitude: -85.47; verbatimCoordinateSystem: Decimal; decimalLatitude: 10.924; decimalLongitude: -85.47; **Identification:** identifiedBy: AJ Fleming; dateIdentified: 2014; **Event:** samplingProtocol: reared from caterpillar of *Conga chydea* (Hesperiidae); verbatimEventDate: 27-Sep-2009; **Record Level:** language: en; institutionCode: CNC; collectionCode: Insects; basisOfRecord: Pinned Specimen
- **Type status:** Paratype. **Occurrence:** occurrenceDetails: http://janzen.sas.upenn.edu; catalogNumber: DHJPAR0034392; recordedBy: D.H. Janzen & W. Hallwachs; individualID: DHJPAR0034392; individualCount: 1; lifeStage: adult; preparations: pinned; otherCatalogNumbers: 09-SRNP-35202; **Taxon:** scientificName: Spathidexia luisrobertogallegosi; phylum: Arthropoda; class: Insecta; order: Diptera; family: Tachinidae; genus: Spathidexia; specificEpithet: luisrobertogallegosi; scientificNameAuthorship: Fleming & Wood, 2015; **Location:** continent: Central America; country: Costa Rica; countryCode: CR; stateProvince: Guanacaste; county: Area de Conservacion Guanacaste; locality: Sector Cacao; verbatimLocality: Sendero Arenales; verbatimElevation: 1080; verbatimLatitude: 10.925; verbatimLongitude: -85.467; verbatimCoordinateSystem: Decimal; decimalLatitude: 10.925; decimalLongitude: -85.467; **Identification:** identifiedBy: AJ Fleming; dateIdentified: 2014; **Event:** samplingProtocol: reared from caterpillar of *Conga chydea* (Hesperiidae); verbatimEventDate: 27-Sep-2009; **Record Level:** language: en; institutionCode: CNC; collectionCode: Insects; basisOfRecord: Pinned Specimen
- **Type status:** Paratype. **Occurrence:** occurrenceDetails: http://janzen.sas.upenn.edu; catalogNumber: DHJPAR0034393; recordedBy: D.H. Janzen & W. Hallwachs; individualID: DHJPAR0034393; individualCount: 1; lifeStage: adult; preparations: pinned; otherCatalogNumbers: 09-SRNP-35192; **Taxon:** scientificName: Spathidexia luisrobertogallegosi; phylum: Arthropoda; class: Insecta; order: Diptera; family: Tachinidae; genus: Spathidexia; specificEpithet: luisrobertogallegosi; scientificNameAuthorship: Fleming & Wood, 2015; **Location:** continent: Central America; country: Costa Rica; countryCode: CR; stateProvince: Guanacaste; county: Area de Conservacion Guanacaste; locality: Sector Cacao; verbatimLocality: Sendero Nayo; verbatimElevation: 1090; verbatimLatitude: 10.924; verbatimLongitude: -85.47; verbatimCoordinateSystem: Decimal; decimalLatitude: 10.924; decimalLongitude: -85.47; **Identification:** identifiedBy: AJ Fleming; dateIdentified: 2014; **Event:** samplingProtocol: reared from caterpillar of *Conga chydea* (Hesperiidae); verbatimEventDate: 12-Apr-2009; **Record Level:** language: en; institutionCode: CNC; collectionCode: Insects; basisOfRecord: Pinned Specimen
- **Type status:** Paratype. **Occurrence:** occurrenceDetails: http://janzen.sas.upenn.edu; catalogNumber: DHJPAR0042574; recordedBy: D.H. Janzen & W. Hallwachs; individualID: DHJPAR0042574; individualCount: 1; lifeStage: adult; preparations: pinned; otherCatalogNumbers: 11-SRNP-30498; **Taxon:** scientificName: Spathidexia luisrobertogallegosi; phylum: Arthropoda; class: Insecta; order: Diptera; family: Tachinidae; genus: Spathidexia; specificEpithet: luisrobertogallegosi; scientificNameAuthorship: Fleming & Wood, 2015; **Location:** continent: Central America; country: Costa Rica; countryCode: CR; stateProvince: Guanacaste; county: Area de Conservacion Guanacaste; locality: Sector Pitilla; verbatimLocality: Sendero Nacho; verbatimElevation: 710; verbatimLatitude: 10.984; verbatimLongitude: -85.425; verbatimCoordinateSystem: Decimal; decimalLatitude: 10.984; decimalLongitude: -85.425; **Identification:** identifiedBy: AJ Fleming; dateIdentified: 2014; **Event:** samplingProtocol: reared from caterpillar of *Conga chydea* (Hesperiidae); verbatimEventDate: 13-Apr-2011; **Record Level:** language: en; institutionCode: CNC; collectionCode: Insects; basisOfRecord: Pinned Specimen
- **Type status:** Paratype. **Occurrence:** occurrenceDetails: http://janzen.sas.upenn.edu; catalogNumber: DHJPAR0042576; recordedBy: D.H. Janzen & W. Hallwachs; individualID: DHJPAR0042576; individualCount: 1; lifeStage: adult; preparations: pinned; otherCatalogNumbers: 11-SRNP-30640; **Taxon:** scientificName: Spathidexia luisrobertogallegosi; phylum: Arthropoda; class: Insecta; order: Diptera; family: Tachinidae; genus: Spathidexia; specificEpithet: luisrobertogallegosi; scientificNameAuthorship: Fleming & Wood, 2015; **Location:** continent: Central America; country: Costa Rica; countryCode: CR; stateProvince: Guanacaste; county: Area de Conservacion Guanacaste; locality: Sector Pitilla; verbatimLocality: Sendero Laguna; verbatimElevation: 680; verbatimLatitude: 10.989; verbatimLongitude: -85.423; verbatimCoordinateSystem: Decimal; decimalLatitude: 10.989; decimalLongitude: -85.423; **Identification:** identifiedBy: AJ Fleming; dateIdentified: 2014; **Event:** samplingProtocol: reared from caterpillar of *Conga chydea* (Hesperiidae); verbatimEventDate: 18-Apr-2011; **Record Level:** language: en; institutionCode: CNC; collectionCode: Insects; basisOfRecord: Pinned Specimen
- **Type status:** Paratype. **Occurrence:** occurrenceDetails: http://janzen.sas.upenn.edu; catalogNumber: DHJPAR0048559; recordedBy: D.H. Janzen & W. Hallwachs; individualID: DHJPAR0048559; individualCount: 1; lifeStage: adult; preparations: pinned; otherCatalogNumbers: 11-SRNP-33677; **Taxon:** scientificName: Spathidexia luisrobertogallegosi; phylum: Arthropoda; class: Insecta; order: Diptera; family: Tachinidae; genus: Spathidexia; specificEpithet: luisrobertogallegosi; scientificNameAuthorship: Fleming & Wood, 2015; **Location:** continent: Central America; country: Costa Rica; countryCode: CR; stateProvince: Guanacaste; county: Area de Conservacion Guanacaste; locality: Sector Pitilla; verbatimLocality: Sendero Laguna; verbatimElevation: 680; verbatimLatitude: 10.989; verbatimLongitude: -85.423; verbatimCoordinateSystem: Decimal; decimalLatitude: 10.989; decimalLongitude: -85.423; **Identification:** identifiedBy: AJ Fleming; dateIdentified: 2014; **Event:** samplingProtocol: reared from caterpillar of *Conga chydea* (Hesperiidae); verbatimEventDate: 07-Dec-2011; **Record Level:** language: en; institutionCode: CNC; collectionCode: Insects; basisOfRecord: Pinned Specimen
- **Type status:** Paratype. **Occurrence:** occurrenceDetails: http://janzen.sas.upenn.edu; catalogNumber: DHJPAR0048614; recordedBy: D.H. Janzen & W. Hallwachs; individualID: DHJPAR0048614; individualCount: 1; lifeStage: adult; preparations: pinned; otherCatalogNumbers: 12-SRNP-30000; **Taxon:** scientificName: Spathidexia luisrobertogallegosi; phylum: Arthropoda; class: Insecta; order: Diptera; family: Tachinidae; genus: Spathidexia; specificEpithet: luisrobertogallegosi; scientificNameAuthorship: Fleming & Wood, 2015; **Location:** continent: Central America; country: Costa Rica; countryCode: CR; stateProvince: Guanacaste; county: Area de Conservacion Guanacaste; locality: Sector Pitilla; verbatimLocality: Loaiciga; verbatimElevation: 445; verbatimLatitude: 11.01983; verbatimLongitude: -85.41342; verbatimCoordinateSystem: Decimal; decimalLatitude: 11.01983; decimalLongitude: -85.41342; **Identification:** identifiedBy: AJ Fleming; dateIdentified: 2014; **Event:** samplingProtocol: reared from caterpillar of *Conga chydea* (Hesperiidae); verbatimEventDate: 04-Jan-2012; **Record Level:** language: en; institutionCode: CNC; collectionCode: Insects; basisOfRecord: Pinned Specimen
- **Type status:** Paratype. **Occurrence:** occurrenceDetails: http://janzen.sas.upenn.edu; catalogNumber: DHJPAR0048564; recordedBy: D.H. Janzen & W. Hallwachs; individualID: DHJPAR0048564; individualCount: 1; lifeStage: adult; preparations: pinned; otherCatalogNumbers: 12-SRNP-30066; **Taxon:** scientificName: Spathidexia luisrobertogallegosi; phylum: Arthropoda; class: Insecta; order: Diptera; family: Tachinidae; genus: Spathidexia; specificEpithet: luisrobertogallegosi; scientificNameAuthorship: Fleming & Wood, 2015; **Location:** continent: Central America; country: Costa Rica; countryCode: CR; stateProvince: Guanacaste; county: Area de Conservacion Guanacaste; locality: Sector Pitilla; verbatimLocality: Ingas; verbatimElevation: 580; verbatimLatitude: 11.00311; verbatimLongitude: -85.42041; verbatimCoordinateSystem: Decimal; decimalLatitude: 11.00311; decimalLongitude: -85.42041; **Identification:** identifiedBy: AJ Fleming; dateIdentified: 2014; **Event:** samplingProtocol: reared from caterpillar of Conga chydea (Hesperiidae); verbatimEventDate: 07-Jan-2012; **Record Level:** language: en; institutionCode: CNC; collectionCode: Insects; basisOfRecord: Pinned Specimen
- **Type status:** Paratype. **Occurrence:** occurrenceDetails: http://janzen.sas.upenn.edu; catalogNumber: DHJPAR0048608; recordedBy: D.H. Janzen & W. Hallwachs; individualID: DHJPAR0048608; individualCount: 1; lifeStage: adult; preparations: pinned; otherCatalogNumbers: 12-SRNP-30209; **Taxon:** scientificName: Spathidexia luisrobertogallegosi; phylum: Arthropoda; class: Insecta; order: Diptera; family: Tachinidae; genus: Spathidexia; specificEpithet: luisrobertogallegosi; scientificNameAuthorship: Fleming & Wood, 2015; **Location:** continent: Central America; country: Costa Rica; countryCode: CR; stateProvince: Guanacaste; county: Area de Conservacion Guanacaste; locality: Sector Pitilla; verbatimLocality: Pasmompa; verbatimElevation: 440; verbatimLatitude: 11.01926; verbatimLongitude: -85.40997; verbatimCoordinateSystem: Decimal; decimalLatitude: 11.01926; decimalLongitude: -85.40997; **Identification:** identifiedBy: AJ Fleming; dateIdentified: 2014; **Event:** samplingProtocol: reared from caterpillar of *Justinia norda* (Hesperiidae); verbatimEventDate: 04-Jan-2012; **Record Level:** language: en; institutionCode: CNC; collectionCode: Insects; basisOfRecord: Pinned Specimen
- **Type status:** Paratype. **Occurrence:** occurrenceDetails: http://janzen.sas.upenn.edu; catalogNumber: DHJPAR0048609; recordedBy: D.H. Janzen & W. Hallwachs; individualID: DHJPAR0048609; individualCount: 1; lifeStage: adult; preparations: pinned; otherCatalogNumbers: 12-SRNP-30302; **Taxon:** scientificName: Spathidexia luisrobertogallegosi; phylum: Arthropoda; class: Insecta; order: Diptera; family: Tachinidae; genus: Spathidexia; specificEpithet: luisrobertogallegosi; scientificNameAuthorship: Fleming & Wood, 2015; **Location:** continent: Central America; country: Costa Rica; countryCode: CR; stateProvince: Guanacaste; county: Area de Conservacion Guanacaste; locality: Sector Pitilla; verbatimLocality: Sendero Rotulo; verbatimElevation: 510; verbatimLatitude: 11.01355; verbatimLongitude: -85.42406; verbatimCoordinateSystem: Decimal; decimalLatitude: 11.01355; decimalLongitude: -85.42406; **Identification:** identifiedBy: AJ Fleming; dateIdentified: 2014; **Event:** samplingProtocol: reared from caterpillar of *Conga chydea* (Hesperiidae); verbatimEventDate: 31-Jan-2012; **Record Level:** language: en; institutionCode: CNC; collectionCode: Insects; basisOfRecord: Pinned Specimen
- **Type status:** Paratype. **Occurrence:** occurrenceDetails: http://janzen.sas.upenn.edu; catalogNumber: DHJPAR0048629; recordedBy: D.H. Janzen & W. Hallwachs; individualID: DHJPAR0048629; individualCount: 1; lifeStage: adult; preparations: pinned; otherCatalogNumbers: 12-SRNP-30522; **Taxon:** scientificName: Spathidexia luisrobertogallegosi; phylum: Arthropoda; class: Insecta; order: Diptera; family: Tachinidae; genus: Spathidexia; specificEpithet: luisrobertogallegosi; scientificNameAuthorship: Fleming & Wood, 2015; **Location:** continent: Central America; country: Costa Rica; countryCode: CR; stateProvince: Guanacaste; county: Area de Conservacion Guanacaste; locality: Sector Pitilla; verbatimLocality: Pasmompa; verbatimElevation: 440; verbatimLatitude: 11.01926; verbatimLongitude: -85.40997; verbatimCoordinateSystem: Decimal; decimalLatitude: 11.01926; decimalLongitude: -85.40997; **Identification:** identifiedBy: AJ Fleming; dateIdentified: 2014; **Event:** samplingProtocol: reared from caterpillar of *Conga chydea* (Hesperiidae); verbatimEventDate: 26-Feb-2012; **Record Level:** language: en; institutionCode: CNC; collectionCode: Insects; basisOfRecord: Pinned Specimen
- **Type status:** Paratype. **Occurrence:** occurrenceDetails: http://janzen.sas.upenn.edu; catalogNumber: DHJPAR0048603; recordedBy: D.H. Janzen & W. Hallwachs; individualID: DHJPAR0048603; individualCount: 1; lifeStage: adult; preparations: pinned; otherCatalogNumbers: 12-SRNP-30526; **Taxon:** scientificName: Spathidexia luisrobertogallegosi; phylum: Arthropoda; class: Insecta; order: Diptera; family: Tachinidae; genus: Spathidexia; specificEpithet: luisrobertogallegosi; scientificNameAuthorship: Fleming & Wood, 2015; **Location:** continent: Central America; country: Costa Rica; countryCode: CR; stateProvince: Guanacaste; county: Area de Conservacion Guanacaste; locality: Sector Pitilla; verbatimLocality: Ingas; verbatimElevation: 580; verbatimLatitude: 11.00311; verbatimLongitude: -85.42041; verbatimCoordinateSystem: Decimal; decimalLatitude: 11.00311; decimalLongitude: -85.42041; **Identification:** identifiedBy: AJ Fleming; dateIdentified: 2014; **Event:** samplingProtocol: reared from caterpillar of *Anthoptus epictetus* (Hesperiidae); verbatimEventDate: 15-Feb-2012; **Record Level:** language: en; institutionCode: CNC; collectionCode: Insects; basisOfRecord: Pinned Specimen
- **Type status:** Paratype. **Occurrence:** occurrenceDetails: http://janzen.sas.upenn.edu; catalogNumber: DHJPAR0048636; recordedBy: D.H. Janzen & W. Hallwachs; individualID: DHJPAR0048636; individualCount: 1; lifeStage: adult; preparations: pinned; otherCatalogNumbers: 12-SRNP-30636; **Taxon:** scientificName: Spathidexia luisrobertogallegosi; phylum: Arthropoda; class: Insecta; order: Diptera; family: Tachinidae; genus: Spathidexia; specificEpithet: luisrobertogallegosi; scientificNameAuthorship: Fleming & Wood, 2015; **Location:** continent: Central America; country: Costa Rica; countryCode: CR; stateProvince: Guanacaste; county: Area de Conservacion Guanacaste; locality: Sector Pitilla; verbatimLocality: Estacion Pitilla; verbatimElevation: 675; verbatimLatitude: 10.98931; verbatimLongitude: -85.42581; verbatimCoordinateSystem: Decimal; decimalLatitude: 10.98931; decimalLongitude: -85.42581; **Identification:** identifiedBy: AJ Fleming; dateIdentified: 2014; **Event:** samplingProtocol: reared from caterpillar of *Conga chydea* (Hesperiidae); verbatimEventDate: 27-Mar-2012; **Record Level:** language: en; institutionCode: CNC; collectionCode: Insects; basisOfRecord: Pinned Specimen
- **Type status:** Paratype. **Occurrence:** occurrenceDetails: http://janzen.sas.upenn.edu; catalogNumber: DHJPAR0034394; recordedBy: D.H. Janzen & W. Hallwachs; individualID: DHJPAR0034394; individualCount: 1; lifeStage: adult; preparations: pinned; otherCatalogNumbers: 09-SRNP-35179; **Taxon:** scientificName: Spathidexia luisrobertogallegosi; phylum: Arthropoda; class: Insecta; order: Diptera; family: Tachinidae; genus: Spathidexia; specificEpithet: luisrobertogallegosi; scientificNameAuthorship: Fleming & Wood, 2015; **Location:** continent: Central America; country: Costa Rica; countryCode: CR; stateProvince: Guanacaste; county: Area de Conservacion Guanacaste; locality: Sector Cacao; verbatimLocality: Sendero Nayo; verbatimElevation: 1090; verbatimLatitude: 10.92446; verbatimLongitude: -85.46953; verbatimCoordinateSystem: Decimal; decimalLatitude: 10.92446; decimalLongitude: -85.46953; **Identification:** identifiedBy: AJ Fleming; dateIdentified: 2014; **Event:** samplingProtocol: reared from caterpillar of *Conga chydea* (Hesperiidae); verbatimEventDate: 18-Feb-2009; **Record Level:** language: en; institutionCode: CNC; collectionCode: Insects; basisOfRecord: Pinned Specimen

#### Description

**Male** (Fig. 3a, b, c), **head**: Frontal vitta dark black, narrowed apically to less than the width of the ocellar triangle, face 4 times as wide as frontal vitta; frontal bristles arise no lower than level of pedicel; yellow hairs lining the margin of the frontal vitta; antenna orange with dark blotches on the apical outer surface; arista brown and plumose, trichia at least 4 times as long as width of base of arista, tapering at apex of arista; proclinate orbital bristles present; parafrontal gold to just below lower proclinate orbital bristles, then transitioning to silver with minute hairs over its entire surface; parafacial silver; palpi orange; short row of yellow supravibrissal hairs along facial margin. **Thorax**: greyish-gold when viewed dorsally with four faintly visible longitudinal grey vittae, these becoming slightly more visible post-suturally, appearing broken at thoracic suture; three post sutural dorsocentral bristles; scutellum concolorous with thorax over its entirety, thorax silver grey when viewed laterally; anterior femur bearing silvery pruinosity, anterior tibiae slightly yellow and hirsute with black tarsi, 2^nd^ and 3^rd^ pair of legs entirely black. **Wings**: pale smoky greyish in color, vein R_4+5_ setose along its entirety; vein R_1_ bare. **Abdomen**: abdominal tergites dark black medially, with bright grey band covering 1/3^rd^ or more of tergal surface arising at the anterior margins of abdominal tergites T3, T4 and T5, these bands wrapping around to the underside. Bright yellow blotches appear along tergites T1+2, T3 and the up to half of T4, these blotches do not continue ventrally to the tip of the abdomen; tergites T3, T4 and T5 possessing medial marginal bristles. Lateral marginal bristles on T1+2. Medium length yellow hairs visible over entire body, in particular visible extending from underside of T1+2.

**Female** (Fig. 3d, e, f), **Head**: Frontal vitta dark black, narrowed apically to less than the width of the ocellar triangle, face 6 times as wide as frontal vitta; frontal bristles arise no lower than level of pedicel; yellow hairs lining the margin of the frontal vitta; antenna orange with dark blotches on the apical outer surface; arista brown and plumose, trichia at least 4 times as long as width of base of arista, tapering at apex of arista; proclinate orbital bristles present; parafrontal entirely gold at apex up to insertion of upper proclinate orbital bristle with minute hairs over upper half of its surface; parafacial silver; palpi orange; short row of yellow supravibrissal hairs along facial margin. **Thorax**: greyish-gold when viewed dorsally with four faintly visible longitudinal grey vittae, these becoming slightly more visible post-suturally, appearing broken at thoracic suture; three post sutural dorsocentral bristles; scutellum concolorous with thorax over its entirety, thorax silver grey when viewed laterally; anterior femur bearing silvery pruinosity; legs anterior femur bearing silvery pruinosity, anterior tibiae slightly yellow and hirsute with black tarsi, 2^nd^ and 3^rd^ pair of legs entirely black. **Wings**: pale smoky greyish in color, vein R_4+5_ setose along its entirety; vein R_1_ bare. **Abdomen**: abdominal tergites dark shiny black medially, with a mid-dorsally broke bright grey band covering 1/3^rd^ or more of tergal surface arising at the anterior margins of abdominal tergites T3, T4 and T5, these bands wrapping around to the underside; tergites T3, T4 and T5 possessing medial marginal bristles, sharp ovipositor extending out 2 times the length of T5. Lateral marginal bristles on T1+2. Medium length yellow hairs visible over entire body, not as hirsute as males, in particular visible extending from underside of T1+2.

#### Diagnosis

Differs from *S. cerussata*, who also possesses fine white hairs on parafacialia, due to its gold thorax, broadly yellow lateral coloration, and yellow orange antenna with darkened apices.

#### Etymology

This species is named in honor of Sr. Luis Roberto Gallegos (RIP), whose large land holdings in Sector El Hacha, Sector Orosi and Sector Pocosol became major parts of the newly forming Area de Conservacion Guanacaste in the late 1980's.

#### Distribution

Costa Rica, ACG, Prov. Guanacaste, rain forest, cloud forest (marginally) and dry forest-rain forest interface (primarily), 280–1460 m elevation.

#### Ecology

Reared 50 times from *Conga chydea* (Hesperiidae, Hesperiinae) only, always feeding on grasses on forest edges in rain forest and the interface between rain forest and dry forest.

### Spathidexia luteola

Fleming & Wood 2015 sp. n.

urn:lsid:zoobank.org:act:46DF154E-AFF3-43B8-A06A-11B253F90E49

#### Materials

- **Type status:** Holotype. **Occurrence:** occurrenceDetails: http://janzen.sas.upenn.edu; catalogNumber: 04-SRNP-45008; recordedBy: D.H. Janzen & W. Hallwachs; individualID: 04-SRNP-45008; individualCount: 1; sex: M; lifeStage: adult; preparations: pinned; **Taxon:** scientificName: Spathidexia luteola; phylum: Arthropoda; class: Insecta; order: Diptera; family: Tachinidae; genus: Spathidexia; specificEpithet: luteola; **Location:** continent: Central America; country: Costa Rica; countryCode: CR; stateProvince: Guanacaste; county: Area de Conservacion Guanacaste; locality: Sector Cacao; verbatimLocality: Gongora Bananal; verbatimElevation: 600; verbatimLatitude: 10.88919; verbatimLongitude: -85.47609; verbatimCoordinateSystem: Decimal; decimalLatitude: 10.88919; decimalLongitude: -85.47609; **Identification:** identifiedBy: AJ Fleming; dateIdentified: 2014; **Event:** samplingProtocol: reared from caterpillar of Vacerra aeas (Hesperiidae); verbatimEventDate: 03-Apr-2004; **Record Level:** language: en; institutionCode: CNC; collectionCode: Insects; basisOfRecord: Pinned Specimen
- **Type status:** Paratype. **Occurrence:** occurrenceDetails: http://janzen.sas.upenn.edu; catalogNumber: DHJPAR0011066; recordedBy: D.H. Janzen & W. Hallwachs; individualID: DHJPAR0011066; individualCount: 1; lifeStage: adult; preparations: pinned; otherCatalogNumbers: 04-SRNP-14634; **Taxon:** scientificName: Spathidexia luteola; phylum: Arthropoda; class: Insecta; order: Diptera; family: Tachinidae; genus: Spathidexia; specificEpithet: luteola; **Location:** continent: Central America; country: Costa Rica; countryCode: CR; stateProvince: Guanacaste; county: Area de Conservacion Guanacaste; locality: Sector Santa Rosa; verbatimLocality: Bosque San Emilio; verbatimElevation: 300; verbatimLatitude: 10.84389; verbatimLongitude: -85.61384; verbatimCoordinateSystem: Decimal; decimalLatitude: 10.84389; decimalLongitude: -85.61384; **Identification:** identifiedBy: AJ Fleming; dateIdentified: 2014; **Event:** samplingProtocol: reared from caterpillar of *Moeris stroma* (Hesperiidae); verbatimEventDate: 22-Oct-2004; **Record Level:** language: en; institutionCode: CNC; collectionCode: Insects; basisOfRecord: Pinned Specimen
- **Type status:** Paratype. **Occurrence:** occurrenceDetails: http://janzen.sas.upenn.edu; catalogNumber: DHJPAR0018741; recordedBy: D.H. Janzen & W. Hallwachs; individualID: DHJPAR0018741; individualCount: 1; lifeStage: adult; preparations: pinned; otherCatalogNumbers: 04-SRNP-45008; **Taxon:** scientificName: Spathidexia luteola; phylum: Arthropoda; class: Insecta; order: Diptera; family: Tachinidae; genus: Spathidexia; specificEpithet: luteola; **Location:** continent: Central America; country: Costa Rica; countryCode: CR; stateProvince: Guanacaste; county: Area de Conservacion Guanacaste; locality: Sector Cacao; verbatimLocality: Gongora Bananal; verbatimElevation: 600; verbatimLatitude: 10.88919; verbatimLongitude: -85.47609; verbatimCoordinateSystem: Decimal; decimalLatitude: 10.88919; decimalLongitude: -85.47609; **Identification:** identifiedBy: AJ Fleming; dateIdentified: 2014; **Event:** samplingProtocol: reared from caterpillar of *Vacerra aeas* (Hesperiidae); verbatimEventDate: 03-Apr-2004; **Record Level:** language: en; institutionCode: CNC; collectionCode: Insects; basisOfRecord: Pinned Specimen
- **Type status:** Paratype. **Occurrence:** occurrenceDetails: http://janzen.sas.upenn.edu; catalogNumber: DHJPAR0018742; recordedBy: D.H. Janzen & W. Hallwachs; individualID: DHJPAR0018742; individualCount: 1; lifeStage: adult; preparations: pinned; otherCatalogNumbers: 04-SRNP-45008; **Taxon:** scientificName: Spathidexia luteola; phylum: Arthropoda; class: Insecta; order: Diptera; family: Tachinidae; genus: Spathidexia; specificEpithet: luteola; **Location:** continent: Central America; country: Costa Rica; countryCode: CR; stateProvince: Guanacaste; county: Area de Conservacion Guanacaste; locality: Sector Cacao; verbatimLocality: Gongora Bananal; verbatimElevation: 600; verbatimLatitude: 10.88919; verbatimLongitude: -85.47609; verbatimCoordinateSystem: Decimal; decimalLatitude: 10.88919; decimalLongitude: -85.47609; **Identification:** identifiedBy: AJ Fleming; dateIdentified: 2014; **Event:** samplingProtocol: reared from caterpillar of *Vacerra aeas* (Hesperiidae); verbatimEventDate: 03-Apr-2004; **Record Level:** language: en; institutionCode: CNC; collectionCode: Insects; basisOfRecord: Pinned Specimen
- **Type status:** Paratype. **Occurrence:** occurrenceDetails: http://janzen.sas.upenn.edu; catalogNumber: 04-SRNP-45008; recordedBy: D.H. Janzen & W. Hallwachs; individualID: 04-SRNP-45008; individualCount: 1; lifeStage: adult; preparations: pinned; **Taxon:** scientificName: Spathidexia luteola; phylum: Arthropoda; class: Insecta; order: Diptera; family: Tachinidae; genus: Spathidexia; specificEpithet: luteola; **Location:** continent: Central America; country: Costa Rica; countryCode: CR; stateProvince: Guanacaste; county: Area de Conservacion Guanacaste; locality: Sector Cacao; verbatimLocality: Gongora Bananal; verbatimElevation: 600; verbatimLatitude: 10.88919; verbatimLongitude: -85.47609; verbatimCoordinateSystem: Decimal; decimalLatitude: 10.88919; decimalLongitude: -85.47609; **Identification:** identifiedBy: AJ Fleming; dateIdentified: 2014; **Event:** samplingProtocol: reared from caterpillar of *Vacerra aeas* (Hesperiidae); verbatimEventDate: 03-Apr-2004; **Record Level:** language: en; institutionCode: CNC; collectionCode: Insects; basisOfRecord: Pinned Specimen

#### Description

**Male** (Fig. 4a, b, c), **head**: Frontal vitta dark black, narrowed apically to less than the width of the ocellar triangle, face 6 times as wide as frontal vitta; frontal bristles arise no lower than level of pedicel; yellow hairs lining the margin of the frontal vitta; antenna orange along upper half, first flagellomere darkening apically; arista black and plumose, trichia at least 4 times as long as width of base of arista, tapering at apex of arista; proclinate orbital bristles absent; parafrontal almost entirely silver, gold along apex following margin of frontal vitta, with minute hairs over its entire surface; parafacial silver; palpi orange; short row of yellow supravibrissal hairs along facial margin. **Thorax**: dull greyish-gold when viewed dorsally with two smoky longitudinal grey bands; three post sutural dorsocentral bristles; scutellum concolorous with thorax over its entirety, thorax silver grey when viewed laterally; anterior femur bearing silvery pruinosity; anterior pair of legs with femur bearing silvery pruinosity slightly yellow 1/6 of its length past knee, tibiae yellow and hirsute with black tarsi, 2^nd^ and 3^rd^ pair of legs black entirely. **Wings**: pale smoky greyish in color, vein R_4+5_ setose up to crossvein r-m; vein R_1_ bare. **Abdomen**: abdominal tergites dark shiny black medially, with bright grey band covering 1/10^th^ of anterior margin of T3, and up to 1/5^th^ or more of T4 and T5, these bands wrapping around to the underside. Bright yellow blotches appear along tergites T1+2, T3 and the anterior half of T4; T3, T4 and T5 possessing medial marginal bristles. Lateral marginal bristles on T1+2. Medium length yellow hairs visible over entire body, in particular visible extending from underside of T1+2.

**Female** (Fig. 4d, e, f), **head**: Frontal vitta dark black, narrowed apically to less than the width of the ocellar triangle, face 8 times as wide as frontal vitta; frontal bristles arise no lower than level of pedicel; yellow hairs lining the margin of the frontal vitta; antenna orange along upper half, first flagellomere darkening apically; arista black and plumose, trichia at least 4 times as long as width of base of arista, tapering at apex of arista; proclinate orbital bristles present; parafrontal almost entirely silver, gold along apex surrounding ocellar triangle and following margin of frontal vitta, sparse minute hairs over upper half of fronto-orbital plate; parafacial silver; palpi orange; short row of yellow supravibrissal hairs along facial margin. **Thorax**: greyish-gold when viewed dorsally with four longitudinal grey vittae, these only slightly visible post-suturally, appearing broken at thoracic suture; three post sutural dorsocentral bristles; scutellum bearing white or yellowish pruinosity over its entirety; legs anterior femur bearing silvery pruinosity, anterior pair of legs with tibiae yellow and hirsute with black tarsi, 2^nd^ and 3^rd^ pair of legs black entirely. **Wings**: pale smoky greyish in color, vein R_4+5_ setose up to crossvein c-m; vein R_1_ bare. **Abdomen**: abdominal tergites dark shiny black medially, with bright grey band covering 1/3^rd^ or more of tergal surface arising at the margins of abdominal tergites T3, T4 and T5, these bands wrapping around to the underside. Bright yellow blotches appear along tergites T1+2, T3 and the anterior margin of T4; T3, T4 and T5 possessing medial marginal bristles, ovipositor concealed within segment 5. Lateral marginal bristles on T1+2. Small yellow hairs visible over entire body, not as hirsute as males but still present, in particular visible extending from anepimeron, and from underside of T1+2.

#### Diagnosis

Species closely resembling *S. clemonsi*, differs from the former due to the presence of yellow tibiae on the front pair of legs, yellow/pale hairs covering the entire body, and costal spine is reduced in *S. luteola*, not absent as in *S. clemonsi*. This species is quite similar to *Spathidexia brasiliensis*, from which it can be differentiated by the former having an orange pedicel and narrow ocellar triangle.

#### Etymology

From the Latin adjective, “*luteus*”, yellow, in reference to the pale yellow hairs covering the body of this species, particularly prominent on the underside of the abdomen.

#### Distribution

Costa Rica, ACG, Prov. Guanacaste, dry forest and dry forest-rain forest interface, 300­­–600 m elevation.

#### Ecology

Reared from 2 species of Hesperiidae, Hesperiinae, eating *Lasiacis* (Poaceae) (2 records), bearing 1–5 larvae per caterpillar.

### Spathidexia hernanrodriguezi

Fleming & Wood 2015 sp. n.

urn:lsid:zoobank.org:act:068E420E-0AB0-40CF-8B60-686603F44031

#### Materials

- **Type status:** Holotype. **Occurrence:** occurrenceDetails: http://janzen.sas.upenn.edu; catalogNumber: DHJPAR0018743; recordedBy: D.H. Janzen & W. Hallwachs; individualID: DHJPAR0018743; individualCount: 1; sex: M; lifeStage: adult; preparations: pinned; otherCatalogNumbers: 04-SRNP-10616; **Taxon:** scientificName: Spathidexia hernanrodriguezi; phylum: Arthropoda; class: Insecta; order: Diptera; family: Tachinidae; genus: Spathidexia; specificEpithet: hernanrodriguezi; scientificNameAuthorship: Fleming & Wood, 2015; **Location:** continent: Central America; country: Costa Rica; countryCode: CR; stateProvince: Guanacaste; county: Area de Conservacion Guanacaste; locality: Sector Pocosol; verbatimLocality: Quebrada Aserradero; verbatimElevation: 160; verbatimLatitude: 10.899; verbatimLongitude: -85.564; verbatimCoordinateSystem: Decimal; decimalLatitude: 10.899; decimalLongitude: -85.564; **Identification:** identifiedBy: AJ Fleming; dateIdentified: 2014; **Event:** samplingProtocol: reared from caterpillar of *Corticea corticea* (Hesperiidae); verbatimEventDate: 24-Apr-2006; **Record Level:** language: en; institutionCode: CNC; collectionCode: Insects; basisOfRecord: Pinned Specimen
- **Type status:** Paratype. **Occurrence:** occurrenceDetails: http://janzen.sas.upenn.edu; catalogNumber: DHJPAR0010022; recordedBy: D.H. Janzen & W. Hallwachs; individualID: DHJPAR0010022; individualCount: 1; lifeStage: adult; preparations: pinned; otherCatalogNumbers: 06-SRNP-45170; **Taxon:** scientificName: Spathidexia hernanrodriguezi; phylum: Arthropoda; class: Insecta; order: Diptera; family: Tachinidae; genus: Spathidexia; specificEpithet: hernanrodriguezi; scientificNameAuthorship: Fleming & Wood, 2015; **Location:** continent: Central America; country: Costa Rica; countryCode: CR; stateProvince: Guanacaste; county: Area de Conservacion Guanacaste; locality: Sector Cacao; verbatimLocality: Puente Gongora; verbatimElevation: 540; verbatimLatitude: 10.885; verbatimLongitude: -85.472; verbatimCoordinateSystem: Decimal; decimalLatitude: 10.885; decimalLongitude: -85.472; **Identification:** identifiedBy: AJ Fleming; dateIdentified: 2014; **Event:** samplingProtocol: reared from caterpillar of *Quasimellana antipazina* (Hesperiidae); verbatimEventDate: 02-May-2004; **Record Level:** language: en; institutionCode: CNC; collectionCode: Insects; basisOfRecord: Pinned Specimen
- **Type status:** Paratype. **Occurrence:** occurrenceDetails: http://janzen.sas.upenn.edu; catalogNumber: DHJPAR0018744; recordedBy: D.H. Janzen & W. Hallwachs; individualID: DHJPAR0018744; individualCount: 1; lifeStage: adult; preparations: pinned; otherCatalogNumbers: 04-SRNP-10616; **Taxon:** scientificName: Spathidexia hernanrodriguezi; phylum: Arthropoda; class: Insecta; order: Diptera; family: Tachinidae; genus: Spathidexia; specificEpithet: hernanrodriguezi; scientificNameAuthorship: Fleming & Wood, 2015; **Location:** continent: Central America; country: Costa Rica; countryCode: CR; stateProvince: Guanacaste; county: Area de Conservacion Guanacaste; locality: Sector Pocosol; verbatimLocality: Quebrada Aserradero; verbatimElevation: 160; verbatimLatitude: 10.899; verbatimLongitude: -85.564; verbatimCoordinateSystem: Decimal; decimalLatitude: 10.899; decimalLongitude: -85.564; **Identification:** identifiedBy: AJ Fleming; dateIdentified: 2014; **Event:** samplingProtocol: reared from caterpillar of *Corticea corticea* (Hesperiidae); verbatimEventDate: 02-May-2004; **Record Level:** language: en; institutionCode: CNC; collectionCode: Insects; basisOfRecord: Pinned Specimen
- **Type status:** Paratype. **Occurrence:** occurrenceDetails: http://janzen.sas.upenn.edu; catalogNumber: DHJPAR0018745; recordedBy: D.H. Janzen & W. Hallwachs; individualID: DHJPAR0018745; individualCount: 1; lifeStage: adult; preparations: pinned; otherCatalogNumbers: 06-SRNP-45170; **Taxon:** scientificName: Spathidexia hernanrodriguezi; phylum: Arthropoda; class: Insecta; order: Diptera; family: Tachinidae; genus: Spathidexia; specificEpithet: hernanrodriguezi; scientificNameAuthorship: Fleming & Wood, 2015; **Location:** continent: Central America; country: Costa Rica; countryCode: CR; stateProvince: Guanacaste; county: Area de Conservacion Guanacaste; locality: Sector Cacao; verbatimLocality: Puente Gongora; verbatimElevation: 540; verbatimLatitude: 10.885; verbatimLongitude: -85.472; verbatimCoordinateSystem: Decimal; decimalLatitude: 10.885; decimalLongitude: -85.472; **Identification:** identifiedBy: AJ Fleming; dateIdentified: 2014; **Event:** samplingProtocol: reared from caterpillar of *Quasimellana antipazina* (Hesperiidae); verbatimEventDate: 24-Apr-2006; **Record Level:** language: en; institutionCode: CNC; collectionCode: Insects; basisOfRecord: Pinned Specimen
- **Type status:** Paratype. **Occurrence:** occurrenceDetails: http://janzen.sas.upenn.edu; catalogNumber: DHJPAR0024529; recordedBy: D.H. Janzen & W. Hallwachs; individualID: DHJPAR0024529; individualCount: 1; lifeStage: adult; preparations: pinned; otherCatalogNumbers: 08-SRNP-12254; **Taxon:** scientificName: Spathidexia hernanrodriguezi; phylum: Arthropoda; class: Insecta; order: Diptera; family: Tachinidae; genus: Spathidexia; specificEpithet: hernanrodriguezi; scientificNameAuthorship: Fleming & Wood, 2015; **Location:** continent: Central America; country: Costa Rica; countryCode: CR; stateProvince: Guanacaste; county: Area de Conservacion Guanacaste; locality: Sector Pocosol; verbatimLocality: Pozo Centeno; verbatimElevation: 155; verbatimLatitude: 10.9; verbatimLongitude: -85.566; verbatimCoordinateSystem: Decimal; decimalLatitude: 10.9; decimalLongitude: -85.566; **Identification:** identifiedBy: AJ Fleming; dateIdentified: 2014; **Event:** samplingProtocol: reared from caterpillar of *Cymaenes odilia trebius* (Hesperiidae); verbatimEventDate: 22-Apr-2008; **Record Level:** language: en; institutionCode: CNC; collectionCode: Insects; basisOfRecord: Pinned Specimen
- **Type status:** Paratype. **Occurrence:** occurrenceDetails: http://janzen.sas.upenn.edu; catalogNumber: DHJPAR0024530; recordedBy: D.H. Janzen & W. Hallwachs; individualID: DHJPAR0024530; individualCount: 1; lifeStage: adult; preparations: pinned; otherCatalogNumbers: 08-SRNP-12251; **Taxon:** scientificName: Spathidexia hernanrodriguezi; phylum: Arthropoda; class: Insecta; order: Diptera; family: Tachinidae; genus: Spathidexia; specificEpithet: hernanrodriguezi; scientificNameAuthorship: Fleming & Wood, 2015; **Location:** continent: Central America; country: Costa Rica; countryCode: CR; stateProvince: Guanacaste; county: Area de Conservacion Guanacaste; locality: Sector Pocosol; verbatimLocality: Pozo Centeno; verbatimElevation: 155; verbatimLatitude: 10.9; verbatimLongitude: -85.566; verbatimCoordinateSystem: Decimal; decimalLatitude: 10.9; decimalLongitude: -85.566; **Identification:** identifiedBy: AJ Fleming; dateIdentified: 2014; **Event:** samplingProtocol: reared from caterpillar of *Vacerra aeas*; verbatimEventDate: 29-Apr-2008; **Record Level:** language: en; institutionCode: CNC; collectionCode: Insects; basisOfRecord: Pinned Specimen

#### Description

**Male** (Fig. 5a, b, c), **head**: Frontal vitta dark black, narrowed apically to less than the width of the ocellar triangle, face 8 times as wide as frontal vitta; frontal bristles arise no lower than level of pedicel; yellow hairs lining the margin of the frontal vitta; antenna orange along upper quarter of first flagellomere and pedicel, darkening apically; arista black and plumose, trichia at least 2 times as long as width of base of arista, tapering at apex of arista; proclinate orbital bristles present; parafrontal almost entirely silver, gold only along apex surrounding ocellar triangle, with minute hairs over its entire surface; parafacial silver; palpi orange; lacking short row of yellow supravibrissal hairs along facial margin. **Thorax**: dull greyish-gold when viewed dorsally with two faint smoky longitudinal grey bands; three post sutural dorsocentral bristles; scutellum concolorous with thorax over its entirety, thorax silver grey when viewed laterally; anterior femur bearing silvery pruinosity; anterior pair of legs with femur bearing silvery pruinosity, tibiae dark brownish yellow and hirsute with black tarsi, 2^nd^ and 3^rd^ pair of legs black entirely; claws of male shorter than apical tarsal segment. **Wings**: pale smoky greyish in color, Vein R_4+5_ setose to just past crossvein r-m; vein R_1_ haired along its entirety. **Abdomen**: abdominal tergites dark shiny black medially, with bright grey band covering 1/5^th^ or more of tergal surface arising along the anterior margins of abdominal tergites T3, T4 and T5, these bands wrapping around to the underside. Bright yellow blotches appear along tergites T1+2, T3 and the anterior half of T4; median marginal bristles on T3, T4 and T5. Lateral marginal bristles on T1+2. Medium length yellow hairs visible over entire body, in particular visible extending from underside of T1+2.

**Female** (Fig. 5d, e, f), **head**: Frontal vitta dark black, narrowed apically to less than the width of the ocellar triangle, face 6 times as wide as frontal vitta; frontal bristles arise no lower than level of pedicel; yellow hairs lining the margin of the frontal vitta; antenna orange along upper quarter of first flagellomere and pedicel, darkening apically; arista black and plumose, trichia at least 2 times as long as width of base of arista, tapering at apex of arista; proclinate orbital bristles present; parafrontal almost entirely silver, gold only along apex surrounding ocellar triangle, up to insertion of upper proclinate orbital, and following margin of frontal vitta, sparse minute hairs over upper half of fronto-orbital plate; parafacial silver; palpi orange; lacking short row of yellow supravibrissal hairs along facial margin. **Thorax**: greyish-gold when viewed dorsally with four faint longitudinal grey vittae, these only slightly visible post-suturally, appearing broken at thoracic suture; three post sutural dorsocentral bristles; scutellum bearing white or yellowish pruinosity over its entirety; legs anterior femur bearing silvery pruinosity, anterior pair of legs with tibiae yellowish tinge and hirsute with black tarsi, 2^nd^ and 3^rd^ pair of legs black entirely. **Wings**: pale smoky greyish in color, vein R_4+5_ setose to just past crossvein r-m; R_1_ haired along its entirety. **Abdomen**: abdominal tergites entirely dark shiny black, with bright grey band covering 1/3^rd^ or more of tergal surface arising at the margins of abdominal tergites T3, T4 and T5, these bands wrapping around to the underside. Silver pruinose patch appearing along underside of tergites T1+2; tergites T3, T4 and T5 possessing medial marginal bristles, sharp ovipositor extending out approximately the same length as T5. Lateral marginal bristles on T1+2. Small yellow hairs visible over entire body, not as hirsute as males but still present, in particular visible extending from anepimeron, and from underside of T1+2.

#### Diagnosis

Resembles *S. cerussata*, differs from the former in the presence of apically enlarged palpus, thorax with almost indistinguisheable vittae, a haired wing vein R_1_, and orange pedicel. Females in *S. cerussata* are similarly colored to males a characteristic not shared by *S. hernanrodriguezi*.

#### Etymology

This species is named in honor of Sr. Hernan Rodriguez Arce (RIP), whose Finca San Gerardo became a large part of Sector San Cristobal of the newly forming Area de Conservación Guanacaste in the early 1990's.

#### Distribution

Costa Rica, ACG, Guanacaste, dry forest, 155–540 m elevation.

#### Ecology

Reared from 3 species of Hesperiidae, Hesperiinae (4 records), feeding on grasses. 5–12 larvae per caterpillar.

### Spathidexia aurantiaca

Fleming & Wood 2015 sp. n.

urn:lsid:zoobank.org:act:3D3061BA-CA4C-443D-9B1D-92AED17B758D

#### Materials

- **Type status:** Holotype. **Occurrence:** occurrenceDetails: http://janzen.sas.upenn.edu; catalogNumber: DHJPAR0010326; recordedBy: D.H. Janzen & W. Hallwachs; individualID: DHJPAR0010326; individualCount: 1; sex: M; lifeStage: adult; preparations: pinned; otherCatalogNumbers: 06-SRNP-42439; **Taxon:** scientificName: Spathidexia aurantiaca; phylum: Arthropoda; class: Insecta; order: Diptera; family: Tachinidae; genus: Spathidexia; specificEpithet: aurantiaca; **Location:** continent: Central America; country: Costa Rica; countryCode: CR; stateProvince: Alajuela; county: Area de Conservacion Guanacaste; locality: Sector Rincon Rain Forest; verbatimLocality: Caverna; verbatimElevation: 420; verbatimLatitude: 10.927; verbatimLongitude: -85.288; verbatimCoordinateSystem: Decimal; decimalLatitude: 10.927; decimalLongitude: -85.288; **Identification:** identifiedBy: AJ Fleming; dateIdentified: 2014; **Event:** samplingProtocol: reared from caterpillars of *Justinia* Burns01 (Hesperiidae); verbatimEventDate: 01-Aug-2006; **Record Level:** language: en; institutionCode: CNC; collectionCode: Insects; basisOfRecord: Pinned Specimen
- **Type status:** Paratype. **Occurrence:** occurrenceDetails: http://janzen.sas.upenn.edu; catalogNumber: DHJPAR0006934; recordedBy: D.H. Janzen & W. Hallwachs; individualID: DHJPAR0006934; individualCount: 1; lifeStage: adult; preparations: pinned; otherCatalogNumbers: 06-SRNP-40499; **Taxon:** scientificName: Spathidexia aurantiaca; phylum: Arthropoda; class: Insecta; order: Diptera; family: Tachinidae; genus: Spathidexia; specificEpithet: aurantiaca; **Location:** continent: Central America; country: Costa Rica; countryCode: CR; stateProvince: Alajuela; county: Area de Conservacion Guanacaste; locality: Sector Rincon Rain Forest; verbatimLocality: Puente Rio Negro; verbatimElevation: 340; verbatimLatitude: 10.904; verbatimLongitude: -85.303; verbatimCoordinateSystem: Decimal; decimalLatitude: 10.904; decimalLongitude: -85.303; **Identification:** identifiedBy: AJ Fleming; dateIdentified: 2014; **Event:** samplingProtocol: reared from caterpillars of *Justinia* Burns01 (Hesperiidae); verbatimEventDate: 12-Mar-2006; **Record Level:** language: en; institutionCode: CNC; collectionCode: Insects; basisOfRecord: Pinned Specimen
- **Type status:** Paratype. **Occurrence:** occurrenceDetails: http://janzen.sas.upenn.edu; catalogNumber: DHJPAR0018746; recordedBy: D.H. Janzen & W. Hallwachs; individualID: DHJPAR0018746; individualCount: 1; lifeStage: adult; preparations: pinned; otherCatalogNumbers: 06-SRNP-40499; **Taxon:** scientificName: Spathidexia aurantiaca; phylum: Arthropoda; class: Insecta; order: Diptera; family: Tachinidae; genus: Spathidexia; specificEpithet: aurantiaca; **Location:** continent: Central America; country: Costa Rica; countryCode: CR; stateProvince: Alajuela; county: Area de Conservacion Guanacaste; locality: Sector Rincon Rain Forest; verbatimLocality: Puente Rio Negro; verbatimElevation: 340; verbatimLatitude: 10.904; verbatimLongitude: -85.303; verbatimCoordinateSystem: Decimal; decimalLatitude: 10.904; decimalLongitude: -85.303; **Identification:** identifiedBy: AJ Fleming; dateIdentified: 2014; **Event:** samplingProtocol: reared from caterpillars of *Justinia* Burns01 (Hesperiidae); verbatimEventDate: 12-Mar-2006; **Record Level:** language: en; institutionCode: CNC; collectionCode: Insects; basisOfRecord: Pinned Specimen
- **Type status:** Paratype. **Occurrence:** occurrenceDetails: http://janzen.sas.upenn.edu; catalogNumber: DHJPAR0040837; recordedBy: D.H. Janzen & W. Hallwachs; individualID: DHJPAR0040837; individualCount: 1; lifeStage: adult; preparations: pinned; otherCatalogNumbers: 10-SRNP-81062; **Taxon:** scientificName: Spathidexia aurantiaca; phylum: Arthropoda; class: Insecta; order: Diptera; family: Tachinidae; genus: Spathidexia; specificEpithet: aurantiaca; **Location:** continent: Central America; country: Costa Rica; countryCode: CR; stateProvince: Alajuela; county: Area de Conservacion Guanacaste; locality: Sector Rincon Rain Forest; verbatimLocality: Selva; verbatimElevation: 410; verbatimLatitude: 10.923; verbatimLongitude: -85.319; verbatimCoordinateSystem: Decimal; decimalLatitude: 10.923; decimalLongitude: -85.319; **Identification:** identifiedBy: AJ Fleming; dateIdentified: 2014; **Event:** samplingProtocol: reared from caterpillars of *Euphyes* Janzen01 (Hesperiidae); verbatimEventDate: 05-Dec-2010; **Record Level:** language: en; institutionCode: CNC; collectionCode: Insects; basisOfRecord: Pinned Specimen
- **Type status:** Paratype. **Occurrence:** occurrenceDetails: http://janzen.sas.upenn.edu; catalogNumber: DHJPAR0040838; recordedBy: D.H. Janzen & W. Hallwachs; individualID: DHJPAR0040838; individualCount: 1; lifeStage: adult; preparations: pinned; otherCatalogNumbers: 10-SRNP-81061; **Taxon:** scientificName: Spathidexia aurantiaca; phylum: Arthropoda; class: Insecta; order: Diptera; family: Tachinidae; genus: Spathidexia; specificEpithet: aurantiaca; **Location:** continent: Central America; country: Costa Rica; countryCode: CR; stateProvince: Alajuela; county: Area de Conservacion Guanacaste; locality: Sector Rincon Rain Forest; verbatimLocality: Selva; verbatimElevation: 410; verbatimLatitude: 10.923; verbatimLongitude: -85.319; verbatimCoordinateSystem: Decimal; decimalLatitude: 10.923; decimalLongitude: -85.319; **Identification:** identifiedBy: AJ Fleming; dateIdentified: 2014; **Event:** samplingProtocol: reared from caterpillars of *Euphyes* Janzen01 (Hesperiidae); verbatimEventDate: 04-Dec-2010; **Record Level:** language: en; institutionCode: CNC; collectionCode: Insects; basisOfRecord: Pinned Specimen
- **Type status:** Paratype. **Occurrence:** occurrenceDetails: http://janzen.sas.upenn.edu; catalogNumber: DHJPAR0042708; recordedBy: D.H. Janzen & W. Hallwachs; individualID: DHJPAR0042708; individualCount: 1; lifeStage: adult; preparations: pinned; otherCatalogNumbers: 11-SRNP-80003; **Taxon:** scientificName: Spathidexia aurantiaca; phylum: Arthropoda; class: Insecta; order: Diptera; family: Tachinidae; genus: Spathidexia; specificEpithet: aurantiaca; **Location:** continent: Central America; country: Costa Rica; countryCode: CR; stateProvince: Alajuela; county: Area de Conservacion Guanacaste; locality: Sector Rincon Rain Forest; verbatimLocality: Selva; verbatimElevation: 410; verbatimLatitude: 10.923; verbatimLongitude: -85.319; verbatimCoordinateSystem: Decimal; decimalLatitude: 10.923; decimalLongitude: -85.319; **Identification:** identifiedBy: AJ Fleming; dateIdentified: 2014; **Event:** samplingProtocol: reared from caterpillars of *Euphyes* Janzen01 (Hesperiidae); verbatimEventDate: 06-May-2011; **Record Level:** language: en; institutionCode: CNC; collectionCode: Insects; basisOfRecord: Pinned Specimen

#### Description

**Male** (Fig. 6a, b, c), **head**: Frontal vitta dark black, and narrow slightly less than the width of the ocellar triangle, face 4 times as wide as frontal vitta; frontal bristles arise no lower than level of pedicel; lacking yellow hairs lining the margin of the frontal vitta; antenna orange along upper quarter of first flagellomere and pedicel, darkening apically; arista black and plumose, enalarged basally up to 1/5 total length, trichia at least 4 times as long as width of base of arista, tapering at apex of arista; proclinate orbital bristles present; parafrontal entirely silver grey, lacking minute hairs over its entire surface; parafacial silver; palpi orange; short row of black supravibrissal hairs along facial margin. **Thorax**: silver-grey when viewed dorsally, thoracic vittae not visible; three post sutural dorsocentral bristles; scutellum concolorous with thorax over its entirety, thorax dull grey when viewed laterally; anterior femur bearing silvery pruinosity; legs entirely black. **Wings**: pale smoky greyish in color, vein R_4+5_ setose to just past crossvein r-m; vein R_1_ bare. **Abdomen**: abdominal tergites entirely bright orange, with slight grey pruinosity covering less than 1/3^rd^ of tergal surface arising at the margins of abdominal tergites T3, T4 and T5, these bands not wrapping around to the underside, darkening on posterior 1/3^rd^ of T5. Tergites T3,T4 and T5 possessing medial marginal bristles. Lateral marginal bristles on T1+2. Medium length yellow hairs visible over entire body, in particular visible extending from underside of T1+2.

**Female** (Fig. 6d, e, f), **head**: Frontal vitta dark black, and narrow to slightly less than the width of the ocellar triangle, face 4 times as wide as frontal vitta; frontal bristles arise no lower than level of pedicel; yellow hairs lining the margin of the frontal vitta; antenna orange only along upper ¼ of first flagellomere, and pedicel then darkening apically; arista black and plumose, enalarged basally up to 1/5 total length, trichia at least 4 times as long as width of base of arista, tapering at apex of arista; proclinate orbital bristles present; parafrontal almost entirely silver, with only slight gold margin surrounding ocellar triangle, sparse minute hairs over upper half of fronto-orbital plate lacking; parafacial silver; palpi orange; short row of black supravibrissal hairs along facial margin. **Thorax**: silver-grey when viewed dorsally with two longitudinal stripes visible only slightly darker than surrounding thorax, only slightly visible post-suturally, appearing broken at thoracic suture; three post sutural dorsocentral bristles; scutellum concolorous with thorax; legs anterior femur bearing silvery pruinosity, anterior pair of legs with tibiae yellowish and hirsute with black tarsi, 2^nd^ and 3^rd^ pair of legs black entirely. **Wings**: pale smoky greyish in color, vein R_4+5_ setose to just past crossvein r-m; vein R_1_ bare. **Abdomen**: abdominal tergites entirely bright orange only darkening on posterior 1/3^rd^ of T5, slight grey pruinosity covering less than 1/3^rd^ of tergal surface arising at the margins of abdominal tergites T3, T4 and T5, traces of which is apparent along the ventral margins of T3 and T4. Tergites T3, T4 and T5 possessing medial marginal bristles, ovipositor concealed within segment 5. Lateral marginal bristles on T1+2. Small yellow hairs visible over entire body, not as hirsute as males but still present, in particular visible extending from anepimeron, and from underside of T1+2.

#### Diagnosis

This species differs from the only other reddish-orange species in *Spathidexia*, *S. creolensis* from the southern US in that *Spathidexia aurantiaca* has more pronounced pruinosity along abdominal margins, black ground color on T5 in both sexes, and gold along the fronto-orbital plate.

#### Etymology

From Latin, "*aurantiaco*" referring to the orange ground color of the abdomen.

#### Distribution

Costa Rica, ACG, Prov. Alajuela, rain forest, 340–420 m elevation.

#### Ecology

Reared from Hesperiidae, specifically two species of sedge (Cyperaceae)-eating Hesperiinae (4 records). 1–5 larvae per caterpillar.

### Spathidexia juanvialesi

Fleming & Wood 2015 sp. n.

urn:lsid:zoobank.org:act:2C61259B-77E7-41F6-86BF-0A6292CB8181

#### Materials

- **Type status:** Holotype. **Occurrence:** occurrenceDetails: http://janzen.sas.upenn.edu; catalogNumber: DHJPAR0046610; recordedBy: D.H. Janzen & W. Hallwachs; individualID: DHJPAR0046610; individualCount: 1; sex: F; lifeStage: adult; preparations: pinned; otherCatalogNumbers: 11-SRNP-72577; **Taxon:** scientificName: Spathidexia juanvialesi; phylum: Arthropoda; class: Insecta; order: Diptera; family: Tachinidae; genus: Spathidexia; specificEpithet: juanvialesi; scientificNameAuthorship: Fleming & Wood, 2015; **Location:** continent: Central America; country: Costa Rica; countryCode: CR; stateProvince: Guanacaste; county: Area de Conservacion Guanacaste; locality: Sector Pitilla; verbatimLocality: Bullas; verbatimElevation: 440; verbatimLatitude: 10.9867; verbatimLongitude: -85.38503; verbatimCoordinateSystem: Decimal; decimalLatitude: 10.9867; decimalLongitude: -85.38503; **Identification:** identifiedBy: AJ Fleming; dateIdentified: 2014; **Event:** samplingProtocol: reared from caterpillar of *Cissia* 95-SRNP-7306 (Nymphalidae); verbatimEventDate: 6/Jan/12; **Record Level:** language: en; institutionCode: CNC; collectionCode: Insects; basisOfRecord: Pinned Specimen
- **Type status:** Paratype. **Occurrence:** occurrenceDetails: http://janzen.sas.upenn.edu; catalogNumber: DHJPAR0046623; recordedBy: D.H. Janzen & W. Hallwachs; individualID: DHJPAR0046623; individualCount: 1; lifeStage: adult; preparations: pinned; otherCatalogNumbers: 11-SRNP-33171; **Taxon:** scientificName: Spathidexia juanvialesi; phylum: Arthropoda; class: Insecta; order: Diptera; family: Tachinidae; genus: Spathidexia; specificEpithet: juanvialesi; scientificNameAuthorship: Fleming & Wood, 2015; **Location:** continent: Central America; country: Costa Rica; countryCode: CR; stateProvince: Guanacaste; county: Area de Conservacion Guanacaste; locality: Sector Pitilla; verbatimLocality: Ingas; verbatimElevation: 580; verbatimLatitude: 11.00311; verbatimLongitude: -85.42041; verbatimCoordinateSystem: Decimal; decimalLatitude: 11.00311; decimalLongitude: -85.42041; **Identification:** identifiedBy: AJ Fleming; dateIdentified: 2014; **Event:** samplingProtocol: reared from caterpillar of *Hermeuptychia* hermesDHJ04 (Nymphalidae); verbatimEventDate: 2/Dec/11; **Record Level:** language: en; institutionCode: CNC; collectionCode: Insects; basisOfRecord: Pinned Specimen

#### Description

**Female**(Fig. 7a, b, c), (to date only females have been reared from the inventory efforts) **head**: Frontal vitta light to dark brown, apically twice as wide as ocellar triangle, face 3 times as wide as frontal vitta; frontal bristles arise no lower than level of pedicel; lacking yellow hairs lining the margin of the frontal vitta; antenna bearing a black first flagellomere on an orange pedicel; arista black and plumose, sparsely haired with widely interspersed trichia at least 2 times as long as width of base of arista, tapering at apex of arista; proclinate orbital bristles present; parafrontal entirely silver, sparse minute hairs over upper half of fronto-orbital plate; parafacial silver; palpi orange with black bulbous tips; lacking any supravibrissal hairs. **Thorax**: dull grey when viewed dorsally, longitudinal vittae smudging together presuturally appearing as two black smudges separated by grey, then into a large precircular dark smudge post suturally. Vittae are nor readily discernible as bands; scutellum dark over more than 2/3 of its surface becoming grey apically concolorous with rest of thorax; anterior pair of legs yellow from coxae to tibiae, anterior femur bearing silvery pruinosity on yellow ground color, 2^nd^ and 3^rd^ pair of legs yellow from coxae to ½ of femur, with black from tibiae to tarsi. **Wings**: pale smoky greyish in color, vein R_4+5_ setose beyond crossvein r-m, but ending approximately 1/3 of vein length away from wing margin; R_1_ haired along its entirety. **Abdomen**: abdominal tergites dark shiny black medially, with bright grey band covering 1/3^rd^ or more of tergal surface arising at the anterior margins of abdominal tergites T4 and T5, these bands wrapping around to the underside. T3 does not bear these grey bands of pruinosity but there is a conspicuous lack of pruinosity and bristles along the anterior margin of T3. Bright yellow blotches appear along tergites T1+2, T3 and the anterior margin of T4; T3, T4 and T5 possessing medial marginal bristles, ovipositor concealed within segment 5. Lateral marginal bristles on T1+2. Lacking any discernible small yellow hairs over entire body, not as hirsute as other species, in particular lacking along anepimeron, and from underside of T1+2.

#### Diagnosis

Similar to *S. setipennis* in the thoracic markings, however *S. juanvialesi* is strongly different in the coloration of the antenna, and the extent of the yellow on the legs.

#### Etymology

This species is named in honor of Sr. Juan Viales (RIP), whose Finca San Cristobal became a large part of Sector San Cristobal of the newly forming Area de Conservación Guanacaste in the eary 1990's.

#### Distribution

Costa Rica, ACG, Prov. Guanacaste, rain forest, 440–580 m elevation.

#### Ecology

Reared twice from grass-eating rain forest Hesperiinae (Hesperiidae).

## Identification Keys

### Key to the species of *Spathidexia* reared from Area de Conservación Guanacaste, Northwestern Costa Rica

**Table:** 

1	Proclinate orbital bristles absent	2
–	Proclinate orbital bristles present	4
2	Fronto-orbital plate entirely silver (no trace of gold)(Figs 1b, 2b)	3
–	Fronto-orbital plate bearing at least traces of gold at least at apex, and surrounding ocellar triangle (Figs 3b, 4b)	*Spathidexia luteola***sp. n.** (♂)
3	Thoracic vittae black, prominent; vein R_1_ bare (Fig. 1a); antenna black; palpus black (Fig. 1b); and legs entirely black	*Spathidexia atripalpus***sp. n.** (♂)
–	Grey thorax with greyish indistinct vittae (only slightly visible, Fig. 2a); vein R_1_ setose along its entirety; antenna orange; palpus orange (Fig. 2b); tibiae yellow	*Spathidexia marioburgosi***sp. n.** (♂)
4	Ground color of abdomen dark, but bearing lateral orange/yellow blotches; or mostly orange (Figs 3a, c, 5a, c)	5
–	Abdominal tergites black, with grayish tomentose bands covering up to basal 1/3 of tergal surface, and with no traces of orange/yellow blotching on abdomen (Figs 3d, f, 5d, f)	9
5	Ground color of abdomen dark, but bearing lateral orange/yellow blotches (Figs 1f, 2f, 4f, 7c)	6
–	Ground color of abdomen orange along its entirety; or with darkened T5 in the case of females (Fig. 6)	*Spathidexia aurantiaca***sp. n.** (♂ & ♀)
6	Abdomen bearing orange/yellow blotches along sides of abdominal tergites, (T1+2, T3, T4, and sometimes T5) (Figs 2f, 4f, 7c)	7
–	Abdomen with yellow blotches only on T1+2 (Fig. 1f), and usually confined to the underside of the abdominal tergite; antenna black; palpi black (Fig. 1e); and legs black	*Spathidexia atripalpus***sp. n.** (♀)
7	Fronto-orbital plate entirely silver with no trace of gold, or if gold present, then only in trace amount alongside ocellar triangle (Figs 2e, 7b); vein R_1_ setose along its entirety; vein R_4+5_ setose along its entirety	8
–	Fronto-orbital plate bearing traces of gold at least at apex, and surrounding ocellar triangle up to insertion of first proclinate orbital bristle (Fig. 4e); antenna orange but darkened apically; orange/yellow blotches present along sides of T1+2, T3, and spilling into T4, but only marginally (Fig. 4d, f); legs black with yellowish tibiae; vein R_1_ bare; vein R_4+5_ setose up to crossvein r-m; grey bands present along margins of T3, T4, and T5	*Spathidexia luteola***sp. n.** (♀)
8	Fronto-orbital plate entirely silver with no trace of gold; wide light brown frontal vitta; antenna black only orange on pedicel (Fig. 7b); thoracic vittae joined pre-suturally appearing as two black blotches, and postsuturally as one large black smudge (Fig. 7a); legs bright yellow from coxae to tibiae; grey abdominal banding only along margin of T4 and T5, and no trace of grey band on T3 (Fig. 7c).	*Spathidexia juanvialesi***sp. n.** (♀)
–	Fronto-orbital plate entirely silver with slight trace of gold alongside ocellar triangle; narrow black frontal vitta; antenna bright orange (Fig. 2e); thorax greyish with light grey thoracic vittae only slightly visible when viewed dorsally (Fig. 2d); anterior legs with yellow tibia, while the mid and posterior legs are entirely black; grey abdominal banding along margin of T3, T4 and T5; yellow abdominal blotches present on T1+2, T3, and slightly on underside of T5 (Fig. 2f).	*Spathidexia marioburgosi***sp. n.** (♀)
9	Fronto-orbital plate with gold color only at apex surrounding ocellar triangle, descending to insertion of upper proclinate orbital in the case of females; antenna dark, only pedicel appears orange (Fig. 5b); thorax grey when viewed dorsally with only slight golden tinge (Fig. 5a, d); legs entirely black (Fig. 5c, f); vein R_1_ bare; vein R_4+5_ setose beyond cross vein r-m, but only up to half its total length in males and up to crossvein r-m in females.	*Spathidexia hernanrodriguezi***sp. n.** (♂ & ♀)
–	Fronto-orbital plate with gold color extending up to second proclinate orbital bristle; antenna orange (Fig. 3b, e); thorax greyish gold when viewed dorsally (Fig. 3a, d); anterior pair of legs entirely with distinctive yellow ground color on tibiae (Fig. 3c, f); vein R_1_ bare; vein R_4+5_ setose along its entirety, or up to 2/3 total length in females	*Spathidexia luisgallegosi***sp. n.** (♂ & ♀)

This key was prepared based on the specimens collected as a result of the 40+ year rearing effort conducted in ACG. Our key is intended to speak to the fauna present within the confines of the ACG and is not meant to be an exhaustive key to the species within the confines of Costa Rica.

## Supplementary Material

Supplementary material 1 Supplemental Appendix 1 Data type: Neighbor-joining tree
File: oo_36659.pdf DHJ, WH

XML Treatment for Spathidexia atripalpus

XML Treatment for Spathidexia marioburgosi

XML Treatment for Spathidexia luisrobertogallegosi

XML Treatment for Spathidexia luteola

XML Treatment for Spathidexia hernanrodriguezi

XML Treatment for Spathidexia aurantiaca

XML Treatment for Spathidexia juanvialesi

## Figures and Tables

**Figure 1a.**
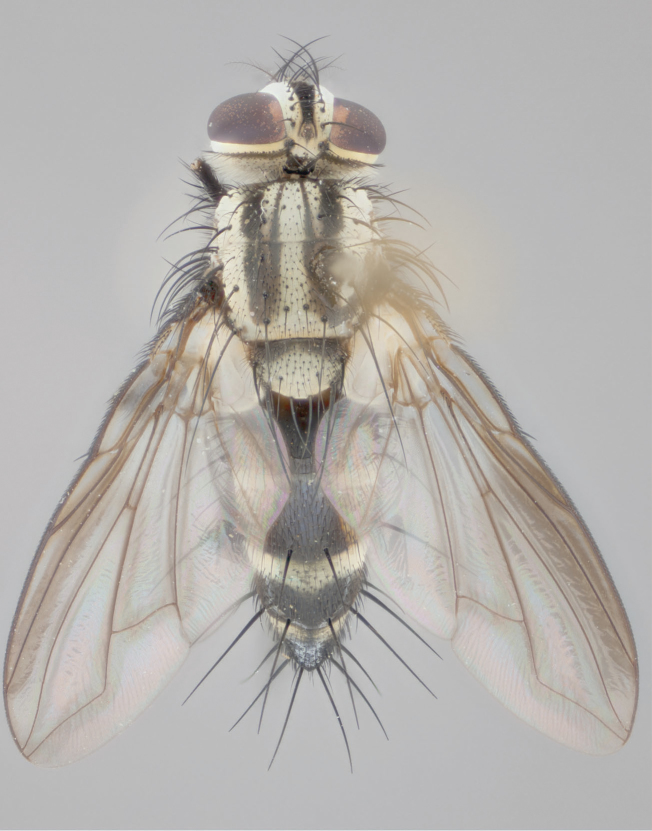
male, dorsal.

**Figure 1b.**
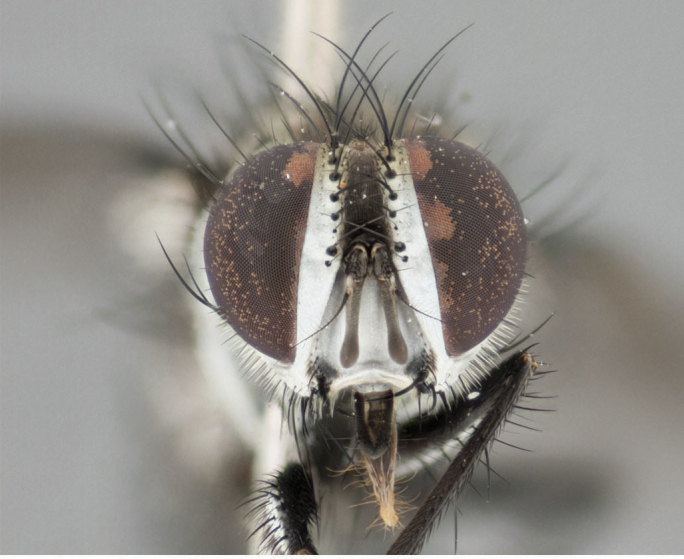
male, head.

**Figure 1c.**
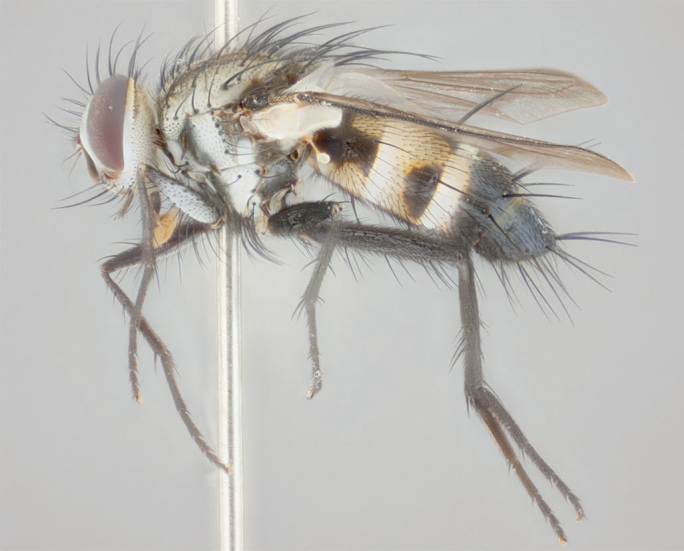
male, lateral.

**Figure 1d.**
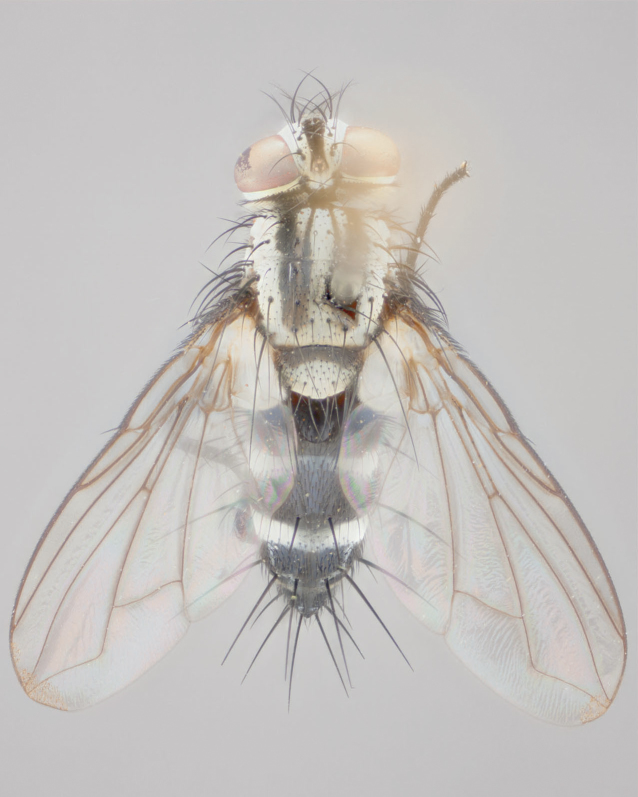
female, dorsal.

**Figure 1e.**
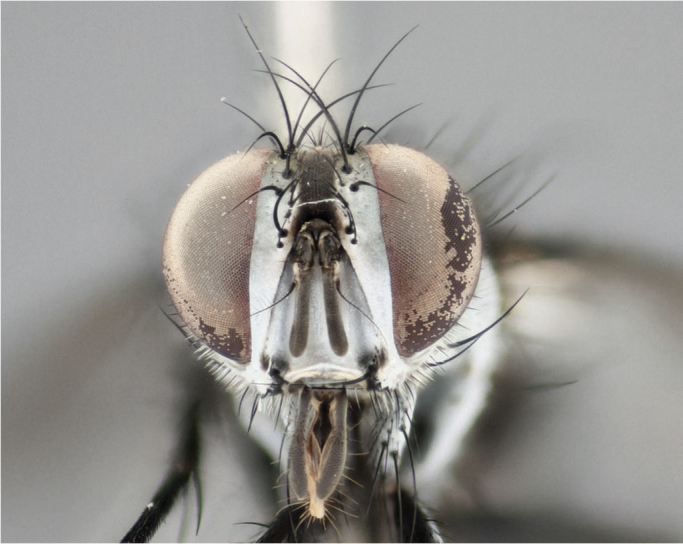
female, head.

**Figure 1f.**
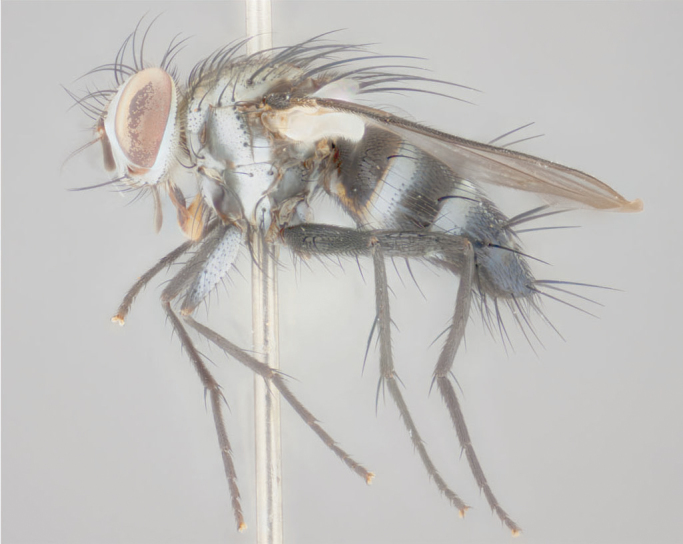
female, lateral.

**Figure 2a.**
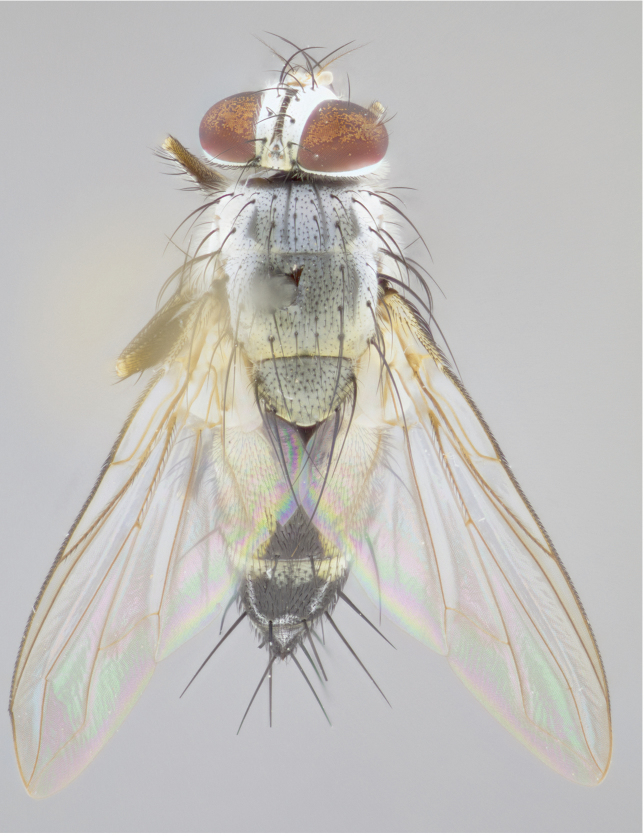
male dorsal.

**Figure 2b.**
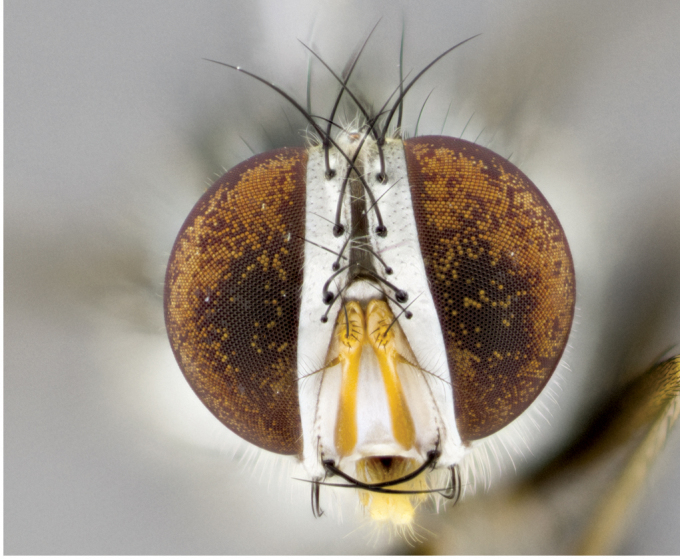
male head.

**Figure 2c.**
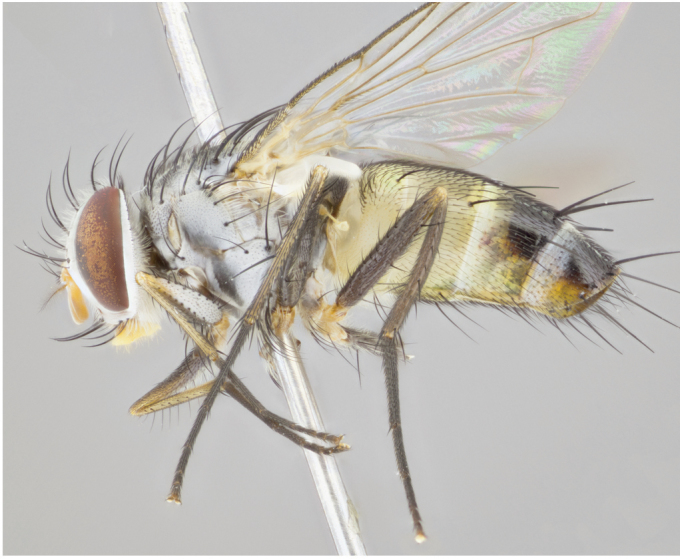
male lateral.

**Figure 2d.**
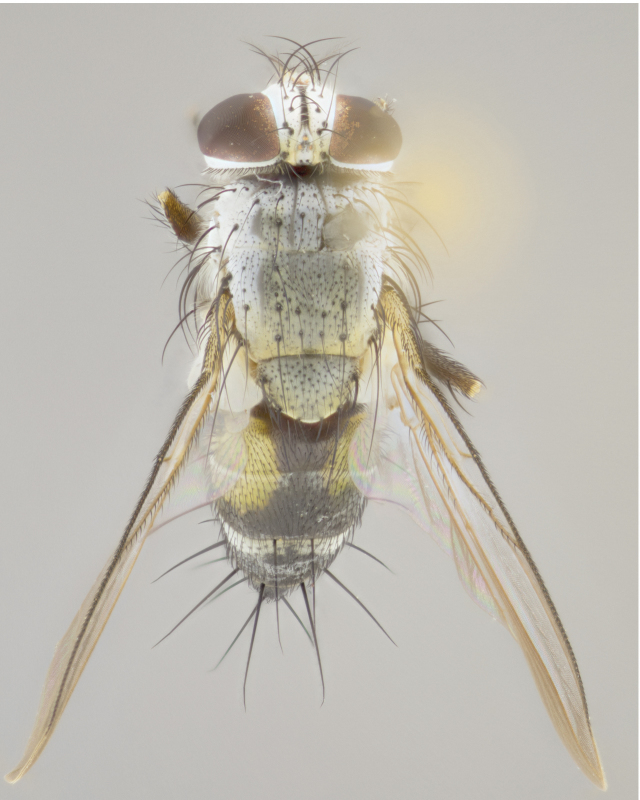
female dorsal.

**Figure 2e.**
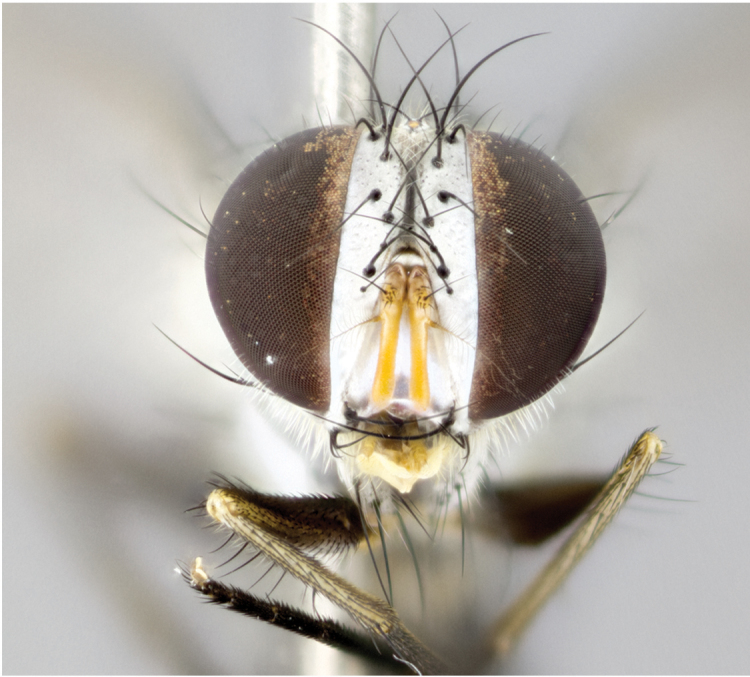
female head.

**Figure 2f.**
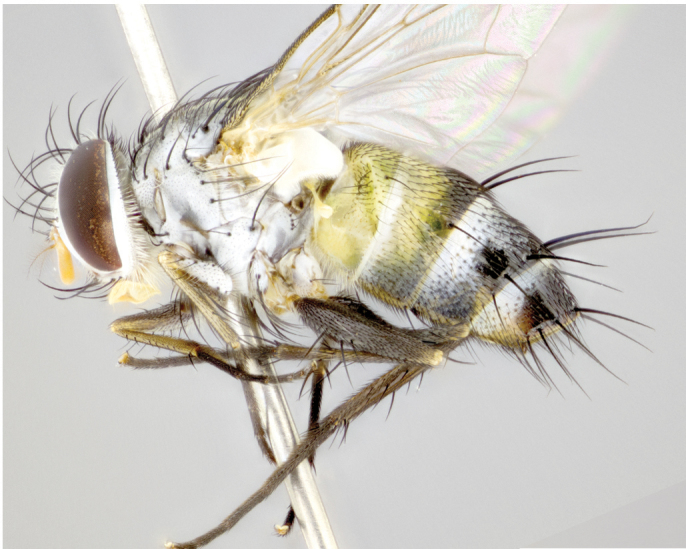
female lateral.

**Figure 3a.**
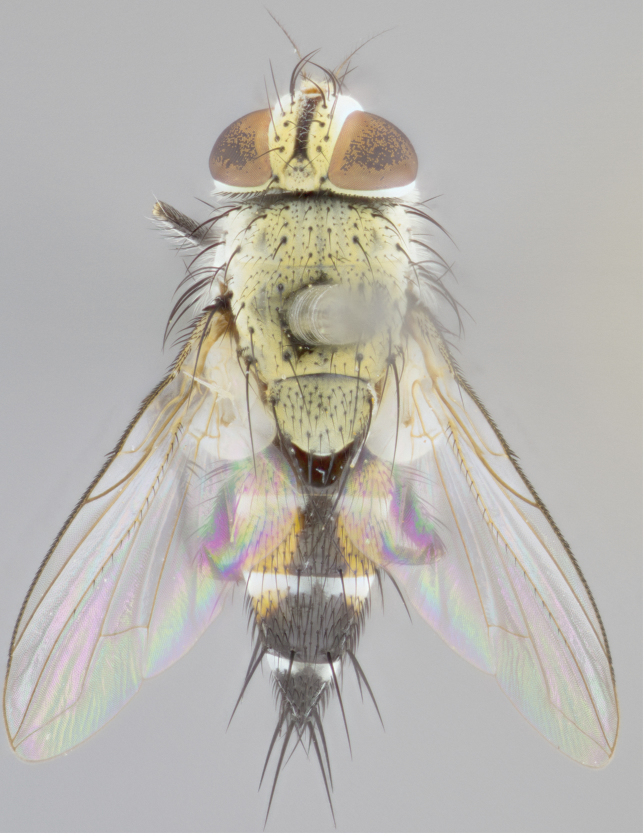
male dorsal.

**Figure 3b.**
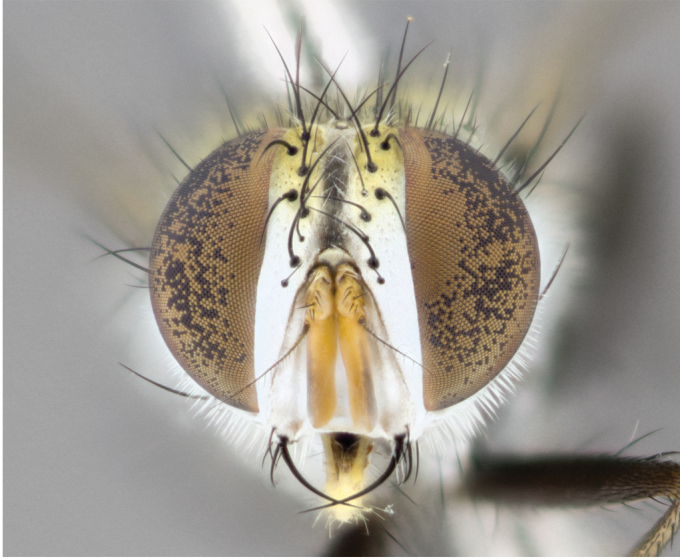
male head.

**Figure 3c.**
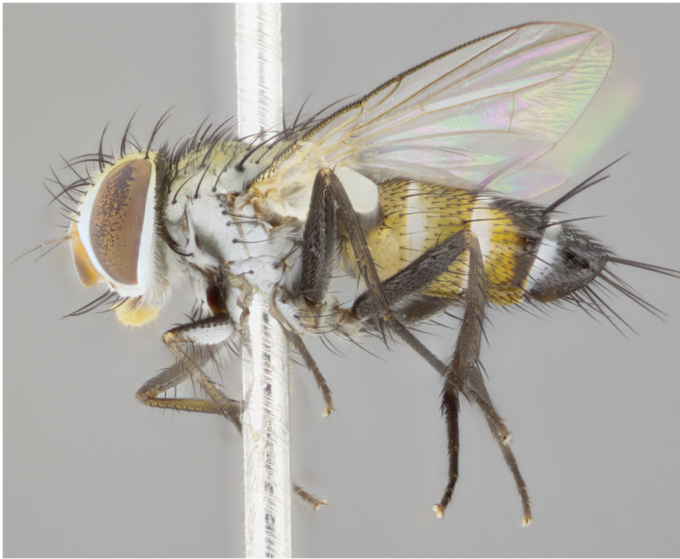
male lateral.

**Figure 3d.**
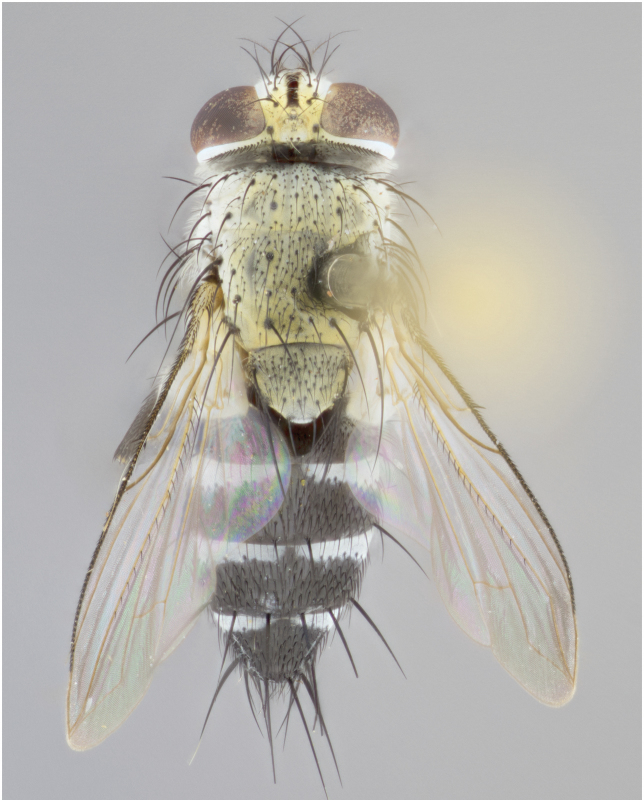
female dorsal.

**Figure 3e.**
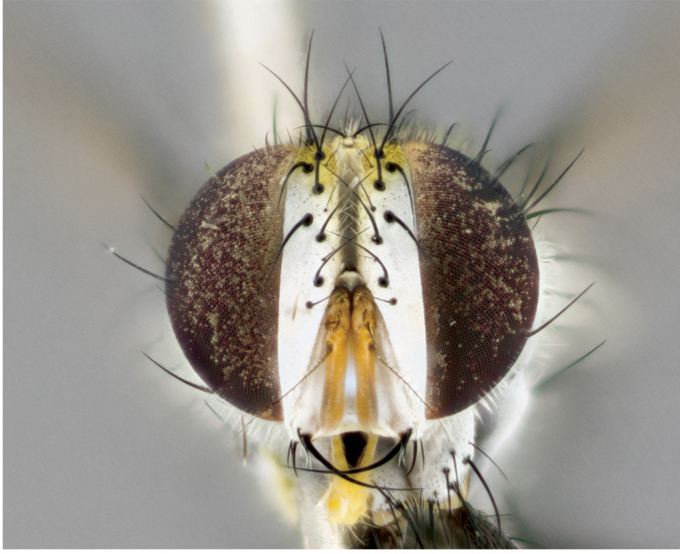
female head.

**Figure 3f.**
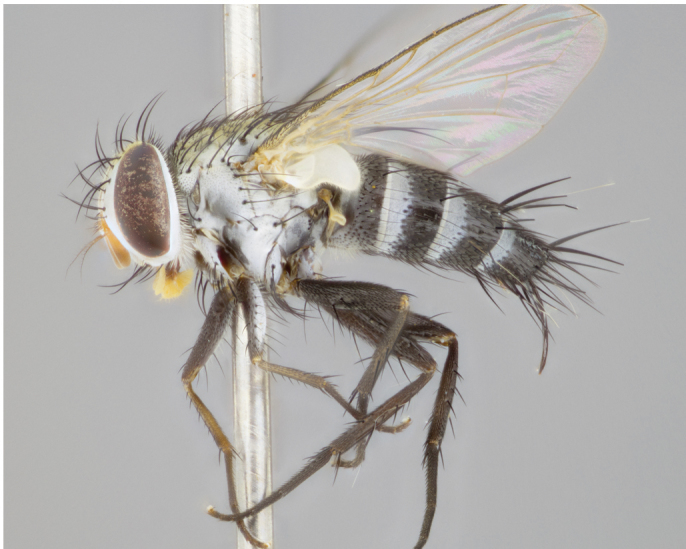
female lateral.

**Figure 4a.**
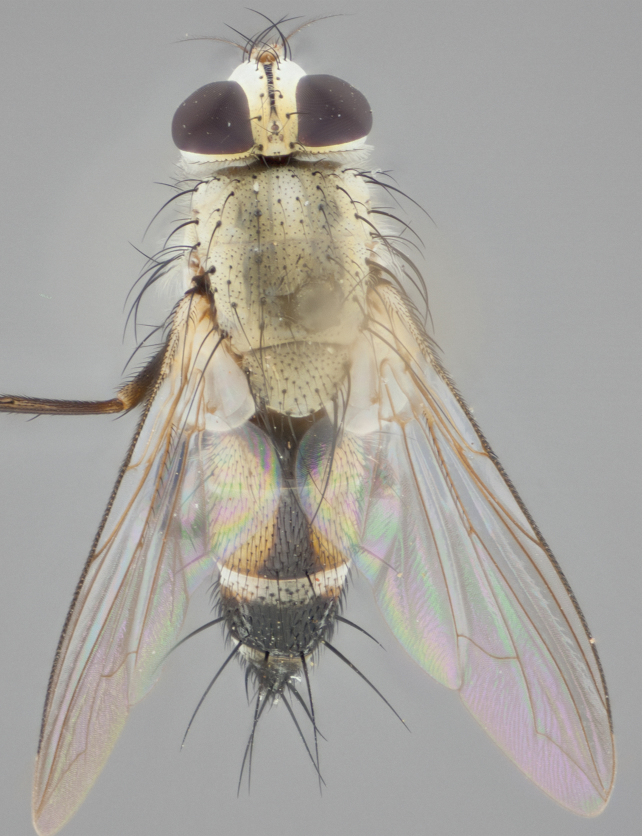
male dorsal.

**Figure 4b.**
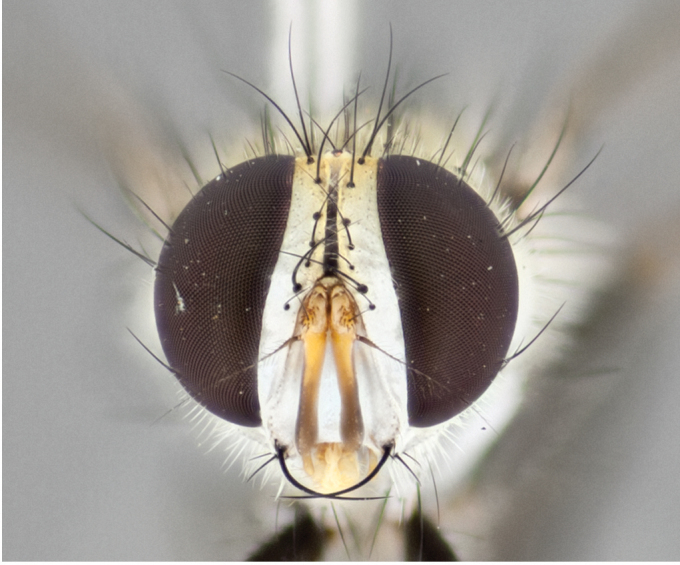
male head.

**Figure 4c.**
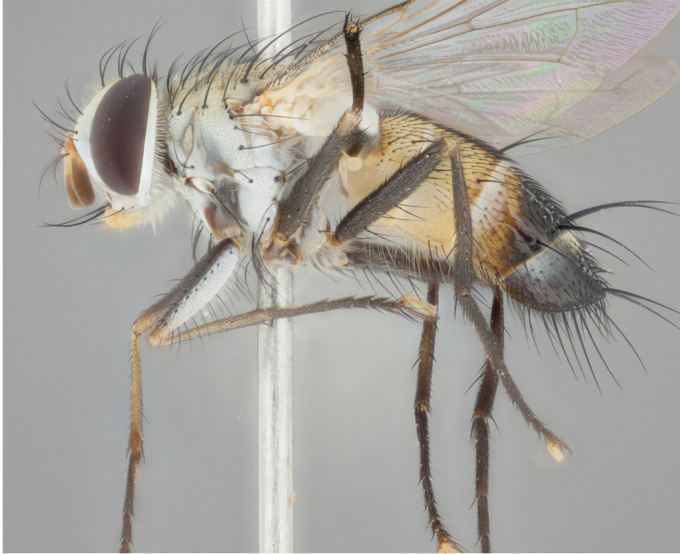
male lateral

**Figure 4d.**
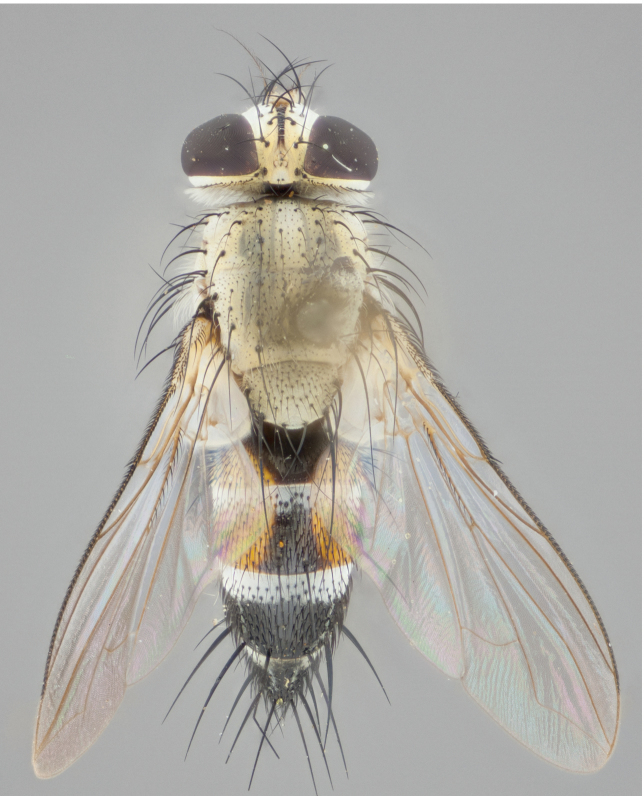
female dorsal

**Figure 4e.**
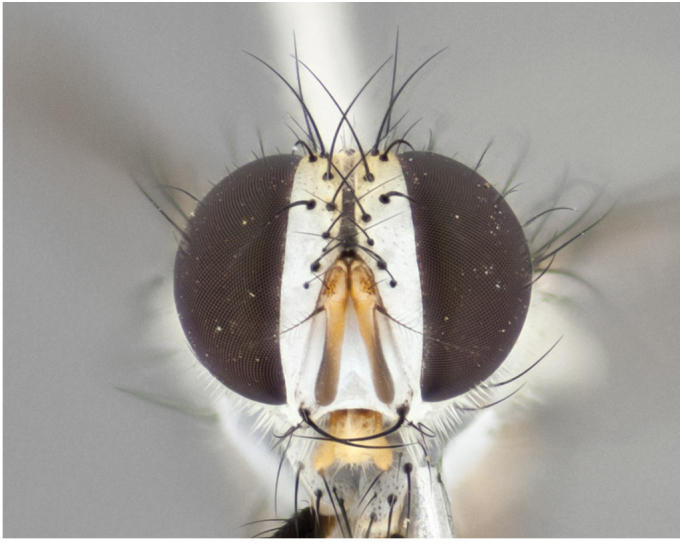
female head

**Figure 4f.**
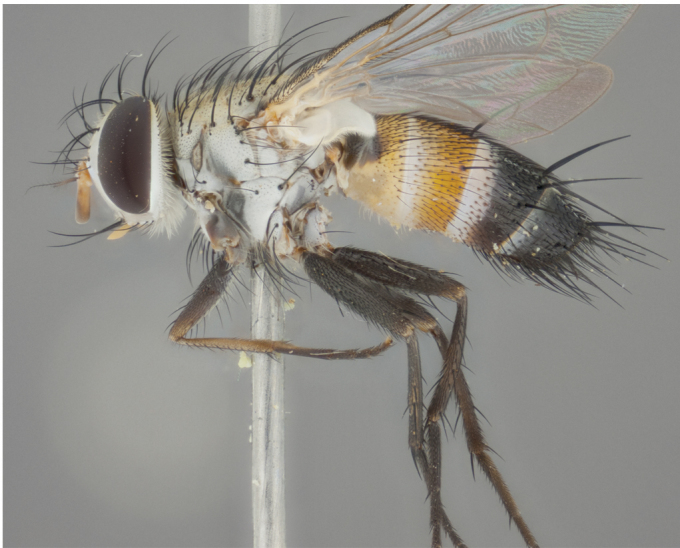
female lateral

**Figure 5a.**
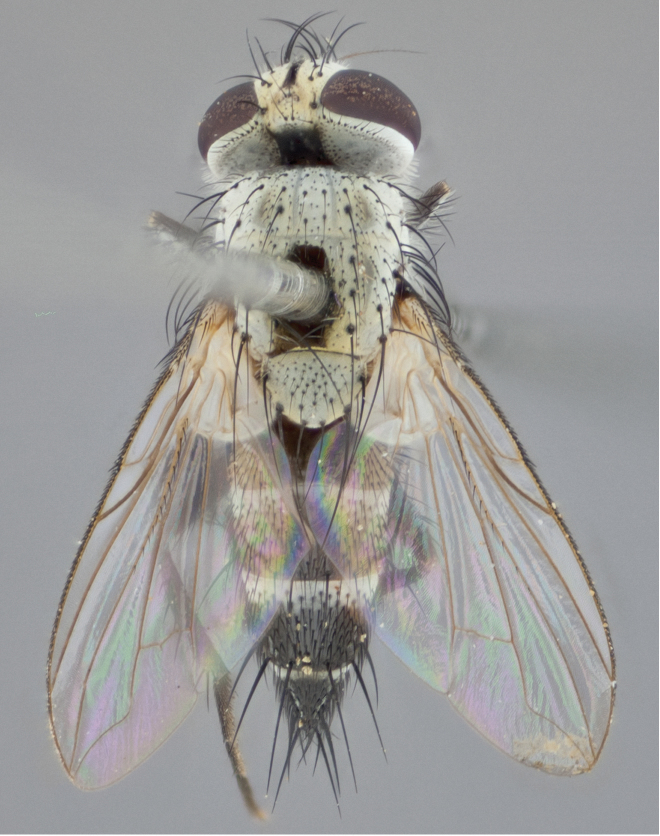
male dorsal.

**Figure 5b.**
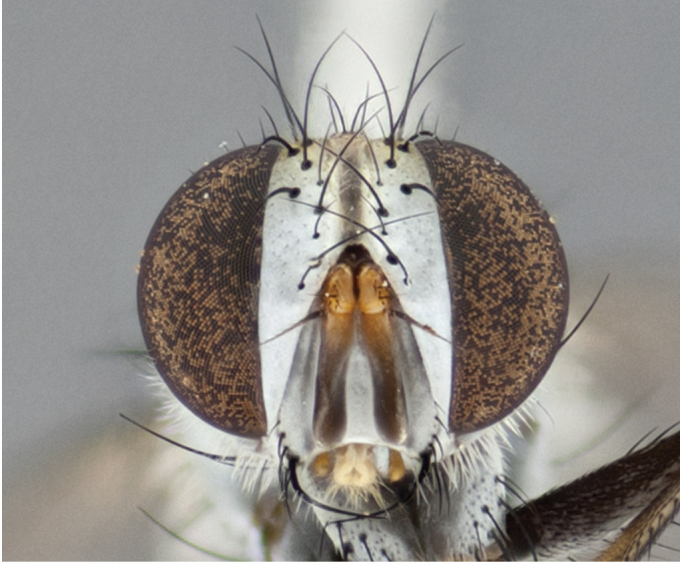
male head.

**Figure 5c.**
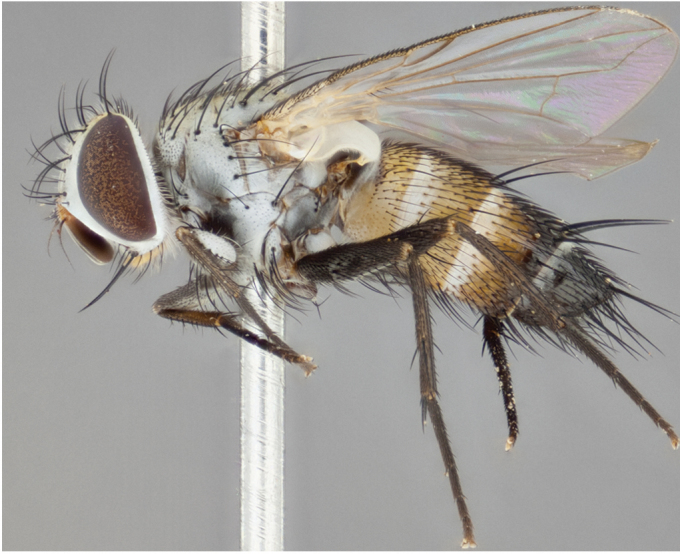
male lateral.

**Figure 5d.**
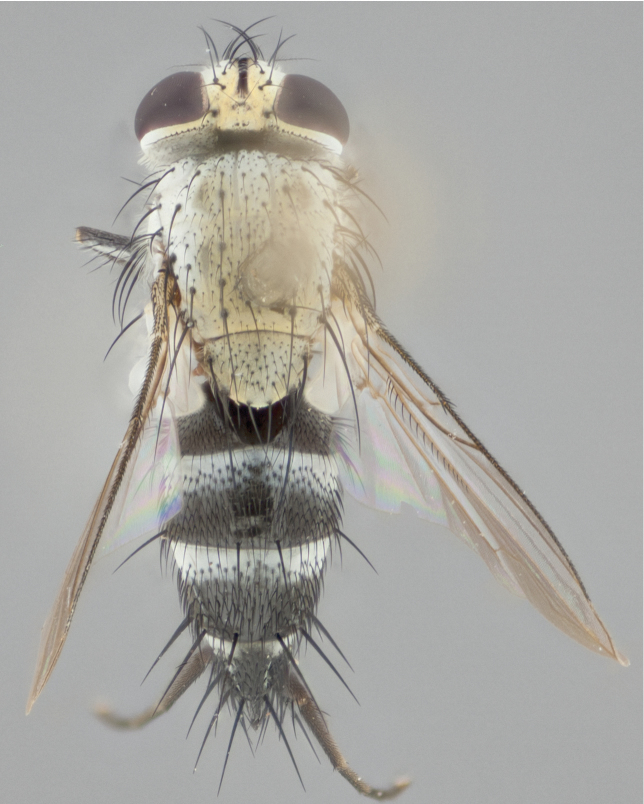
female dorsal.

**Figure 5e.**
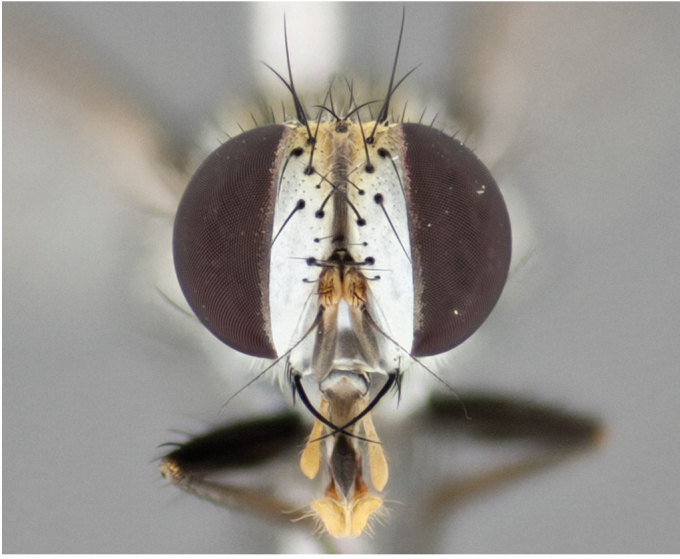
female head.

**Figure 5f.**
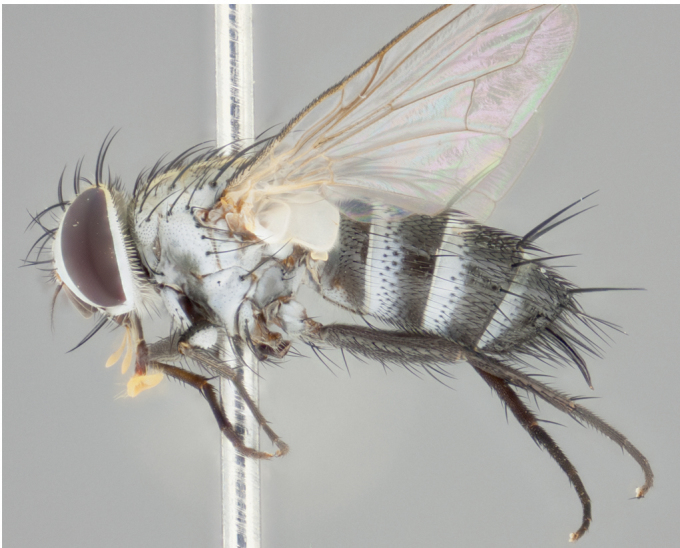
female lateral.

**Figure 6a.**
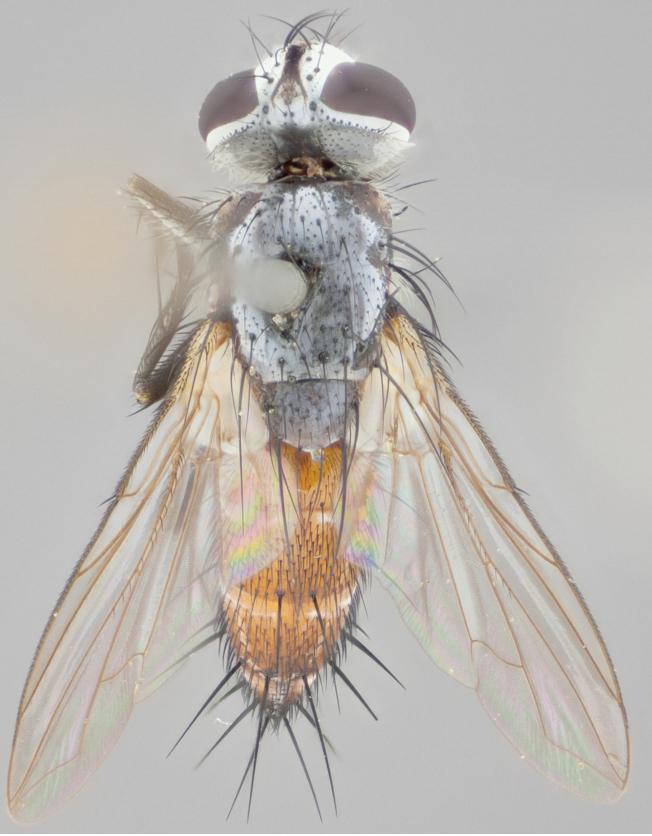
male dorsal.

**Figure 6b.**
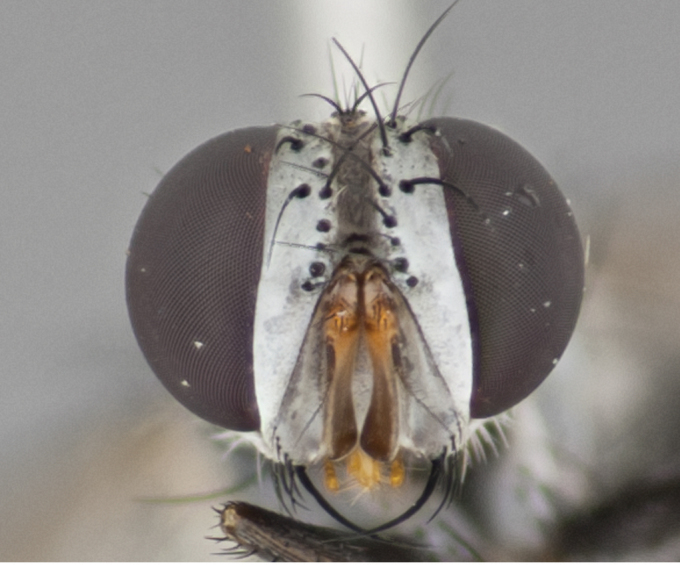
male head.

**Figure 6c.**
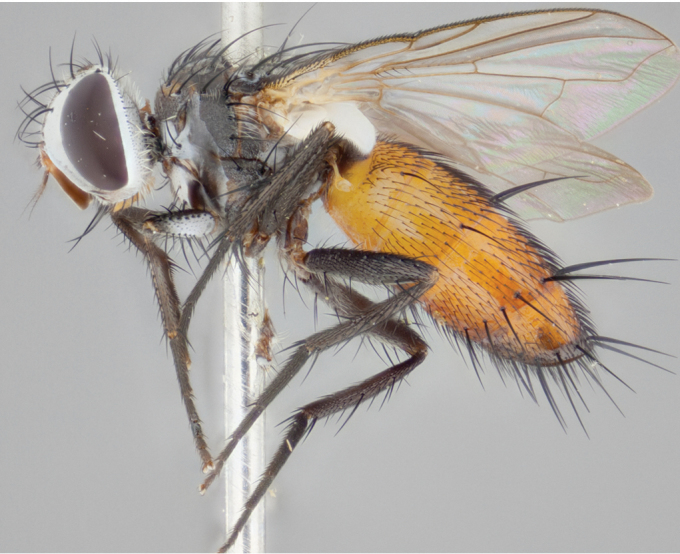
male lateral.

**Figure 6d.**
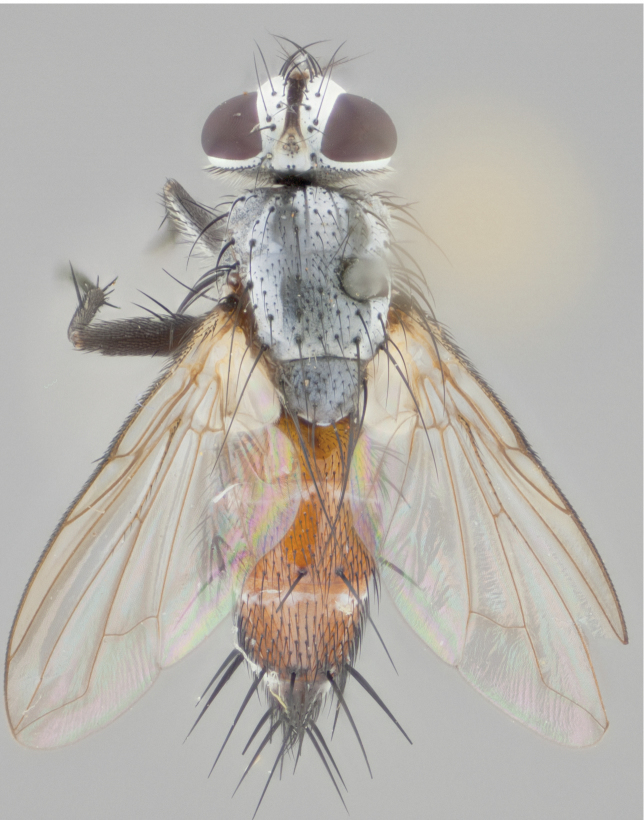
female dorsal.

**Figure 6e.**
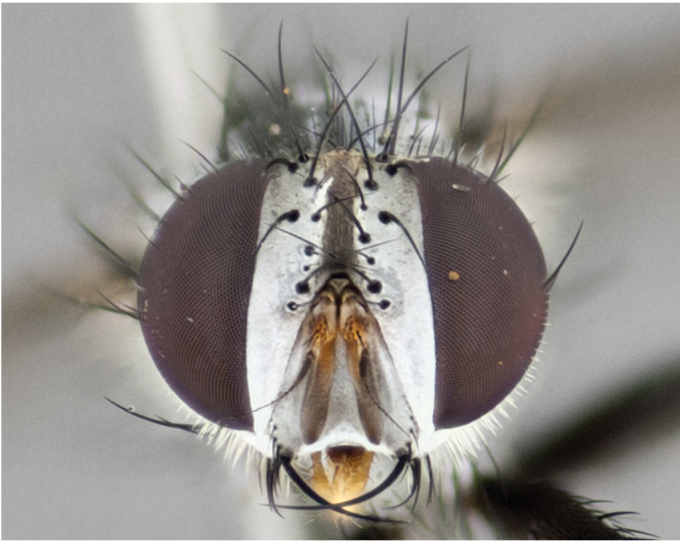
female head.

**Figure 6f.**
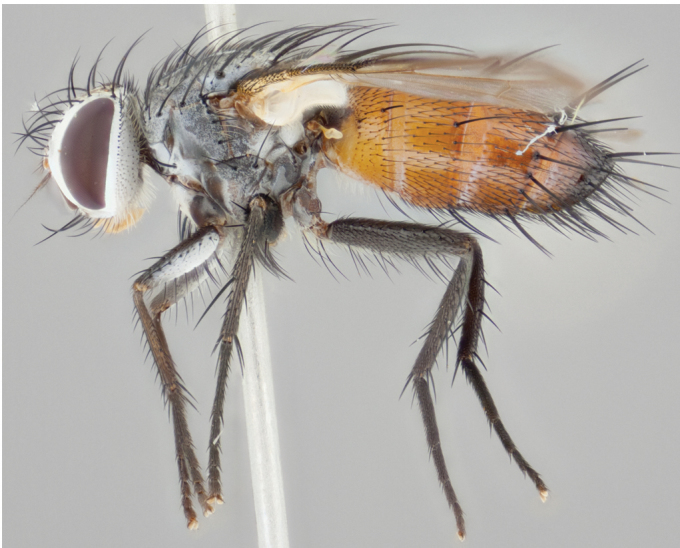
female lateral.

**Figure 7a.**
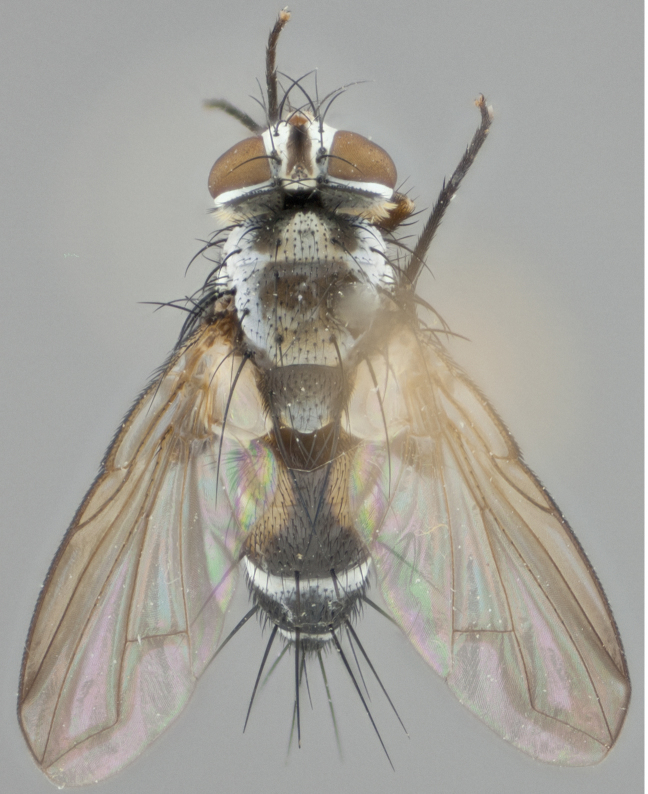
female dorsal.

**Figure 7b.**
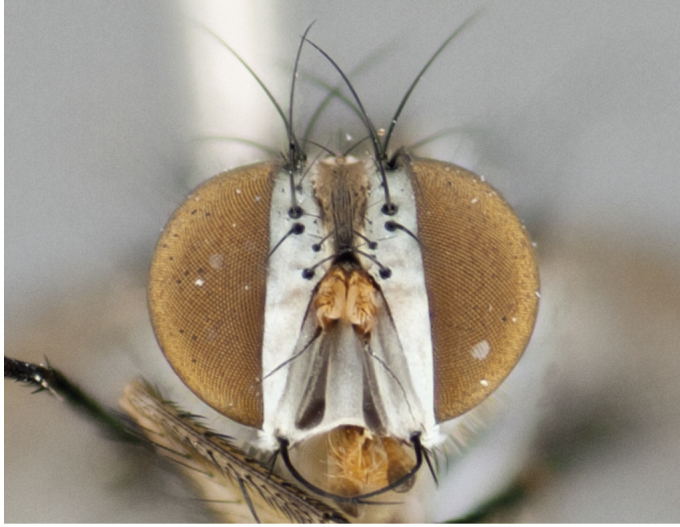
female head.

**Figure 7c.**
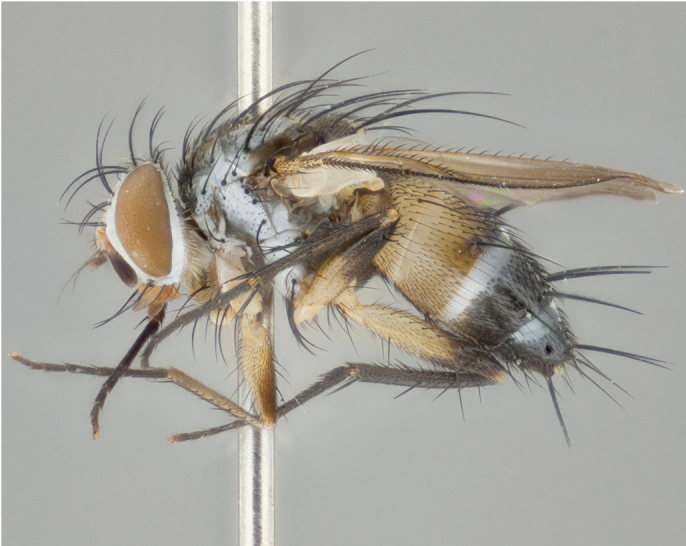
female lateral.
