# Supplementary material for: Seven new species of Spathidexia Townsend (Diptera: Tachinidae) reared from caterpillars in Area de Conservación Guanacaste, Costa Rica
Source: Biodivers Data J. 2015 Mar 25;(3):e4597. doi: 10.3897/BDJ.3.e4597 (PMC4385903; doi:10.3897/BDJ.3.e4597)
Supplement: Supplementary material 1 — Supplemental Appendix 1 [file biodiversity_data_journal-3-e4597-s001.pdf]

BOLD TaxonID Tree

Title	:	SEARCH: Tax(Spathidexia) [SEARCH1]
Date	:	6-January-2015
Data Type	:	Nucleotide
Distance Model	:	Kimura 2 Parameter
Marker	:	COI-5P
Codon Positions	:	1st, 2nd, 3rd
Labels	:	Extra Info, SampleID, Sequence Length
Filters	:	Length > 200
Colorization	:	[blue]=Stop Codons [red]=Contamination or misidentification

Sequence Count	:	168
Species count	:	6
Genus count	:	1
Family count	:	1
Unidentified	:	0

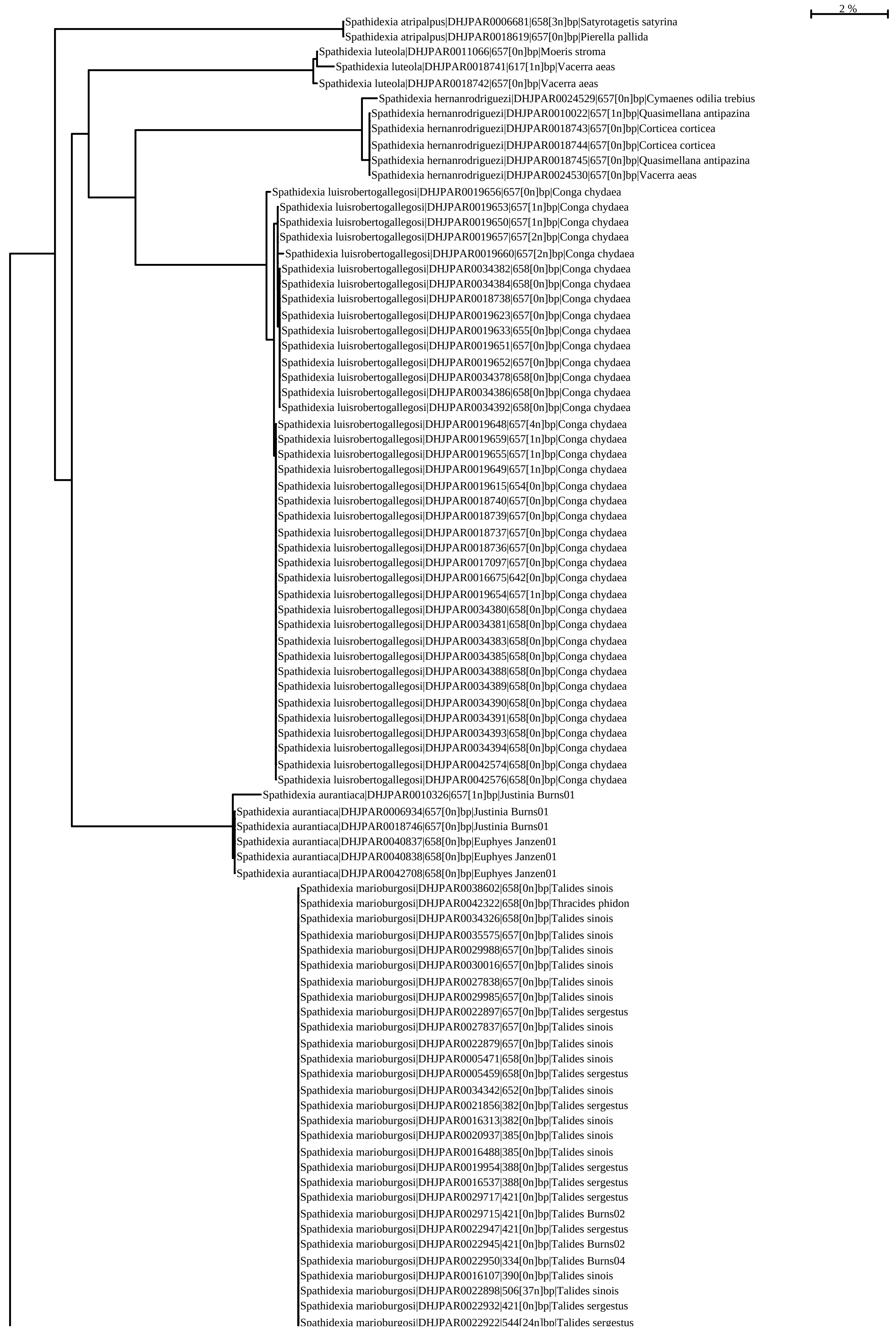
2 %
Spathidexia atripalpus|DHJPAR0006681|658[3n]bp|Satyrotagetis satyrina
Spathidexia atripalpus|DHJPAR0018619|657[0n]bp|Pierella pallida
Spathidexia luteola|DHJPAR0011066|657[0n]bp|Moeris stroma
Spathidexia luteola|DHJPAR0018741|617[1n]bp|Vacerra aeas
Spathidexia luteola|DHJPAR0018742|657[0n]bp|Vacerra aeas
Spathidexia hernanrodriguezi|DHJPAR0024529|657[0n]bp|Cymaenes odilia trebius
Spathidexia hernanrodriguezi|DHJPAR0010022|657[1n]bp|Quasimellana antipazina
Spathidexia hernanrodriguezi|DHJPAR0018743|657[0n]bp|Corticea corticea
Spathidexia hernanrodriguezi|DHJPAR0018744|657[0n]bp|Corticea corticea
Spathidexia hernanrodriguezi|DHJPAR0018745|657[0n]bp|Quasimellana antipazina
Spathidexia hernanrodriguezi|DHJPAR0024530|657[0n]bp|Vacerra aeas
Spathidexia luisrobertogallegosi|DHJPAR0019656|657[0n]bp|Conga chydaea
Spathidexia luisrobertogallegosi|DHJPAR0019653|657[1n]bp|Conga chydaea
Spathidexia luisrobertogallegosi|DHJPAR0019650|657[1n]bp|Conga chydaea
Spathidexia luisrobertogallegosi|DHJPAR0019657|657[2n]bp|Conga chydaea
Spathidexia luisrobertogallegosi|DHJPAR0019660|657[2n]bp|Conga chydaea
Spathidexia luisrobertogallegosi|DHJPAR0034382|658[0n]bp|Conga chydaea
Spathidexia luisrobertogallegosi|DHJPAR0034384|658[0n]bp|Conga chydaea
Spathidexia luisrobertogallegosi|DHJPAR0018738|657[0n]bp|Conga chydaea
Spathidexia luisrobertogallegosi|DHJPAR0019623|657[0n]bp|Conga chydaea
Spathidexia luisrobertogallegosi|DHJPAR0019633|655[0n]bp|Conga chydaea
Spathidexia luisrobertogallegosi|DHJPAR0019651|657[0n]bp|Conga chydaea
Spathidexia luisrobertogallegosi|DHJPAR0019652|657[0n]bp|Conga chydaea
Spathidexia luisrobertogallegosi|DHJPAR0034378|658[0n]bp|Conga chydaea
Spathidexia luisrobertogallegosi|DHJPAR0034386|658[0n]bp|Conga chydaea
Spathidexia luisrobertogallegosi|DHJPAR0034392|658[0n]bp|Conga chydaea
Spathidexia luisrobertogallegosi|DHJPAR0019648|657[4n]bp|Conga chydaea
Spathidexia luisrobertogallegosi|DHJPAR0019659|657[1n]bp|Conga chydaea
Spathidexia luisrobertogallegosi|DHJPAR0019655|657[1n]bp|Conga chydaea
Spathidexia luisrobertogallegosi|DHJPAR0019649|657[1n]bp|Conga chydaea
Spathidexia luisrobertogallegosi|DHJPAR0019615|654[0n]bp|Conga chydaea
Spathidexia luisrobertogallegosi|DHJPAR0018740|657[0n]bp|Conga chydaea
Spathidexia luisrobertogallegosi|DHJPAR0018739|657[0n]bp|Conga chydaea
Spathidexia luisrobertogallegosi|DHJPAR0018737|657[0n]bp|Conga chydaea
Spathidexia luisrobertogallegosi|DHJPAR0018736|657[0n]bp|Conga chydaea
Spathidexia luisrobertogallegosi|DHJPAR0017097|657[0n]bp|Conga chydaea
Spathidexia luisrobertogallegosi|DHJPAR0016675|642[0n]bp|Conga chydaea
Spathidexia luisrobertogallegosi|DHJPAR0019654|657[1n]bp|Conga chydaea
Spathidexia luisrobertogallegosi|DHJPAR0034380|658[0n]bp|Conga chydaea
Spathidexia luisrobertogallegosi|DHJPAR0034381|658[0n]bp|Conga chydaea
Spathidexia luisrobertogallegosi|DHJPAR0034383|658[0n]bp|Conga chydaea
Spathidexia luisrobertogallegosi|DHJPAR0034385|658[0n]bp|Conga chydaea
Spathidexia luisrobertogallegosi|DHJPAR0034388|658[0n]bp|Conga chydaea
Spathidexia luisrobertogallegosi|DHJPAR0034389|658[0n]bp|Conga chydaea
Spathidexia luisrobertogallegosi|DHJPAR0034390|658[0n]bp|Conga chydaea
Spathidexia luisrobertogallegosi|DHJPAR0034391|658[0n]bp|Conga chydaea
Spathidexia luisrobertogallegosi|DHJPAR0034393|658[0n]bp|Conga chydaea
Spathidexia luisrobertogallegosi|DHJPAR0034394|658[0n]bp|Conga chydaea
Spathidexia luisrobertogallegosi|DHJPAR0042574|658[0n]bp|Conga chydaea
Spathidexia luisrobertogallegosi|DHJPAR0042576|658[0n]bp|Conga chydaea
Spathidexia aurantiaca|DHJPAR0010326|657[1n]bp|Justinia Burns01
Spathidexia aurantiaca|DHJPAR0006934|657[0n]bp|Justinia Burns01
Spathidexia aurantiaca|DHJPAR0018746|657[0n]bp|Justinia Burns01
Spathidexia aurantiaca|DHJPAR0040837|658[0n]bp|Euphyes Janzen01
Spathidexia aurantiaca|DHJPAR0040838|658[0n]bp|Euphyes Janzen01
Spathidexia aurantiaca|DHJPAR0042708|658[0n]bp|Euphyes Janzen01
Spathidexia marioburgosi|DHJPAR0038602|658[0n]bp|Talides sinois
Spathidexia marioburgosi|DHJPAR0042322|658[0n]bp|Thracides phidon
Spathidexia marioburgosi|DHJPAR0034326|658[0n]bp|Talides sinois
Spathidexia marioburgosi|DHJPAR0035575|657[0n]bp|Talides sinois
Spathidexia marioburgosi|DHJPAR0029988|657[0n]bp|Talides sinois
Spathidexia marioburgosi|DHJPAR0030016|657[0n]bp|Talides sinois
Spathidexia marioburgosi|DHJPAR0027838|657[0n]bp|Talides sinois
Spathidexia marioburgosi|DHJPAR0029985|657[0n]bp|Talides sinois
Spathidexia marioburgosi|DHJPAR0022897|657[0n]bp|Talides sergestus
Spathidexia marioburgosi|DHJPAR0027837|657[0n]bp|Talides sinois
Spathidexia marioburgosi|DHJPAR0022879|657[0n]bp|Talides sinois
Spathidexia marioburgosi|DHJPAR0005471|658[0n]bp|Talides sinois
Spathidexia marioburgosi|DHJPAR0005459|658[0n]bp|Talides sergestus
Spathidexia marioburgosi|DHJPAR0034342|652[0n]bp|Talides sinois
Spathidexia marioburgosi|DHJPAR0021856|382[0n]bp|Talides sergestus
Spathidexia marioburgosi|DHJPAR0016313|382[0n]bp|Talides sinois
Spathidexia marioburgosi|DHJPAR0020937|385[0n]bp|Talides sinois
Spathidexia marioburgosi|DHJPAR0016488|385[0n]bp|Talides sinois
Spathidexia marioburgosi|DHJPAR0019954|388[0n]bp|Talides sergestus
Spathidexia marioburgosi|DHJPAR0016537|388[0n]bp|Talides sergestus
Spathidexia marioburgosi|DHJPAR0029717|421[0n]bp|Talides sergestus
Spathidexia marioburgosi|DHJPAR0029715|421[0n]bp|Talides Burns02
Spathidexia marioburgosi|DHJPAR0022947|421[0n]bp|Talides sergestus
Spathidexia marioburgosi|DHJPAR0022945|421[0n]bp|Talides Burns02
Spathidexia marioburgosi|DHJPAR0022950|334[0n]bp|Talides Burns04
Spathidexia marioburgosi|DHJPAR0016107|390[0n]bp|Talides sinois
Spathidexia marioburgosi|DHJPAR0022898|506[37n]bp|Talides sinois
Spathidexia marioburgosi|DHJPAR0022932|421[0n]bp|Talides sergestus
Spathidexia marioburgosi|DHJPAR0022922|544[24n]bp|Talides sergestus

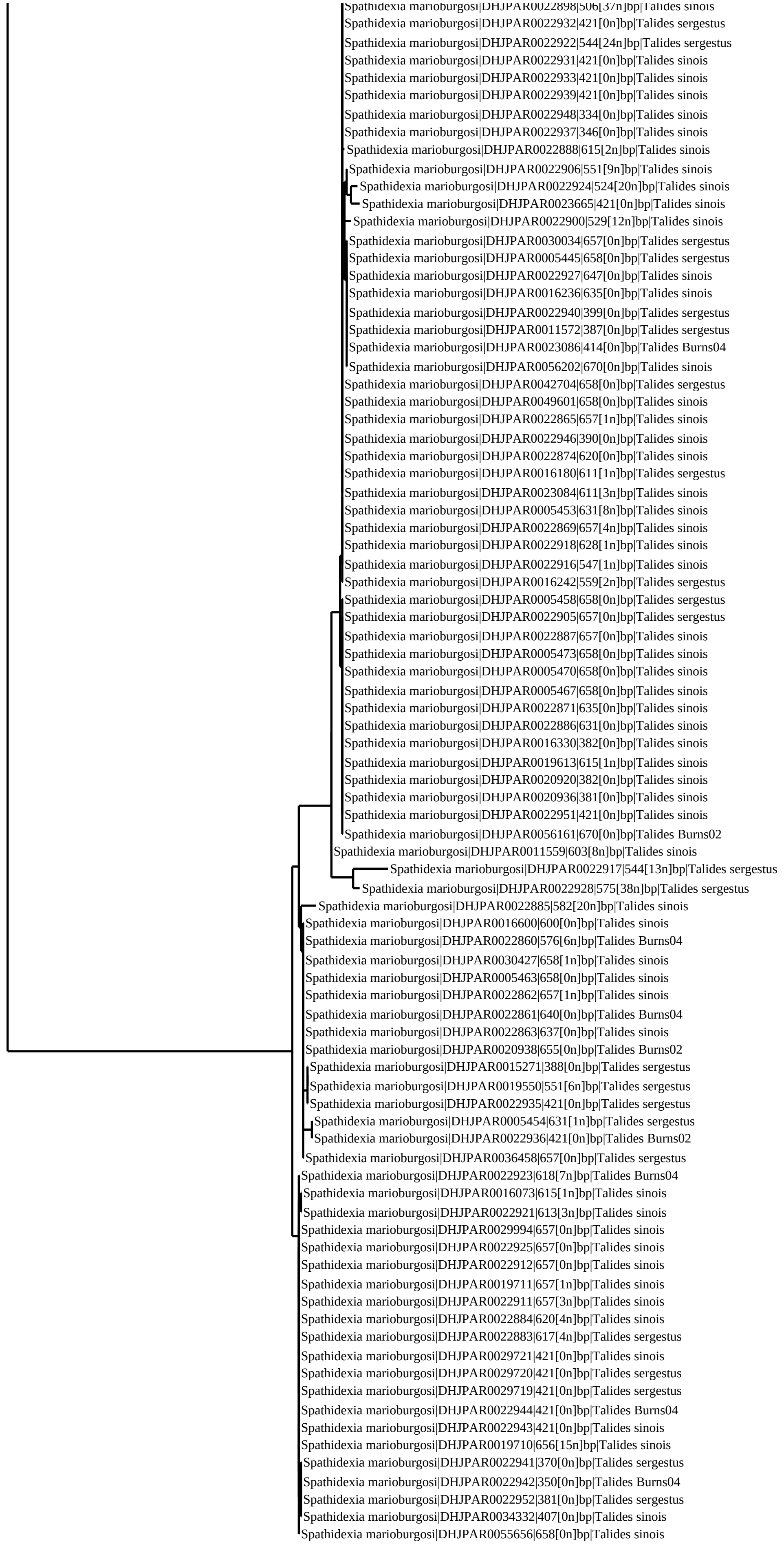

Spathidexia marioburgosi|DHJPAR0022898|506[37n]bp|Talides sinois
Spathidexia marioburgosi|DHJPAR0022932|421[0n]bp|Talides sergestus
Spathidexia marioburgosi|DHJPAR0022922|544[24n]bp|Talides sergestus
Spathidexia marioburgosi|DHJPAR0022931|421[0n]bp|Talides sinois
Spathidexia marioburgosi|DHJPAR0022933|421[0n]bp|Talides sinois
Spathidexia marioburgosi|DHJPAR0022939|421[0n]bp|Talides sinois
Spathidexia marioburgosi|DHJPAR0022948|334[0n]bp|Talides sinois
Spathidexia marioburgosi|DHJPAR0022937|346[0n]bp|Talides sinois
Spathidexia marioburgosi|DHJPAR0022888|615[2n]bp|Talides sinois
Spathidexia marioburgosi|DHJPAR0022906|551[9n]bp|Talides sinois
Spathidexia marioburgosi|DHJPAR0022924|524[20n]bp|Talides sinois
Spathidexia marioburgosi|DHJPAR0023665|421[0n]bp|Talides sinois
Spathidexia marioburgosi|DHJPAR0022900|529[12n]bp|Talides sinois
Spathidexia marioburgosi|DHJPAR0030034|657[0n]bp|Talides sergestus
Spathidexia marioburgosi|DHJPAR0005445|658[0n]bp|Talides sergestus
Spathidexia marioburgosi|DHJPAR0022927|647[0n]bp|Talides sinois
Spathidexia marioburgosi|DHJPAR0016236|635[0n]bp|Talides sinois
Spathidexia marioburgosi|DHJPAR0022940|399[0n]bp|Talides sergestus
Spathidexia marioburgosi|DHJPAR0011572|387[0n]bp|Talides sergestus
Spathidexia marioburgosi|DHJPAR0023086|414[0n]bp|Talides Burns04
Spathidexia marioburgosi|DHJPAR0056202|670[0n]bp|Talides sinois
Spathidexia marioburgosi|DHJPAR0042704|658[0n]bp|Talides sergestus
Spathidexia marioburgosi|DHJPAR0049601|658[0n]bp|Talides sinois
Spathidexia marioburgosi|DHJPAR0022865|657[1n]bp|Talides sinois
Spathidexia marioburgosi|DHJPAR0022946|390[0n]bp|Talides sinois
Spathidexia marioburgosi|DHJPAR0022874|620[0n]bp|Talides sinois
Spathidexia marioburgosi|DHJPAR0016180|611[1n]bp|Talides sergestus
Spathidexia marioburgosi|DHJPAR0023084|611[3n]bp|Talides sinois
Spathidexia marioburgosi|DHJPAR0005453|631[8n]bp|Talides sinois
Spathidexia marioburgosi|DHJPAR0022869|657[4n]bp|Talides sinois
Spathidexia marioburgosi|DHJPAR0022918|628[1n]bp|Talides sinois
Spathidexia marioburgosi|DHJPAR0022916|547[1n]bp|Talides sinois
Spathidexia marioburgosi|DHJPAR0016242|559[2n]bp|Talides sergestus
Spathidexia marioburgosi|DHJPAR0005458|658[0n]bp|Talides sergestus
Spathidexia marioburgosi|DHJPAR0022905|657[0n]bp|Talides sergestus
Spathidexia marioburgosi|DHJPAR0022887|657[0n]bp|Talides sinois
Spathidexia marioburgosi|DHJPAR0005473|658[0n]bp|Talides sinois
Spathidexia marioburgosi|DHJPAR0005470|658[0n]bp|Talides sinois
Spathidexia marioburgosi|DHJPAR0005467|658[0n]bp|Talides sinois
Spathidexia marioburgosi|DHJPAR0022871|635[0n]bp|Talides sinois
Spathidexia marioburgosi|DHJPAR0022886|631[0n]bp|Talides sinois
Spathidexia marioburgosi|DHJPAR0016330|382[0n]bp|Talides sinois
Spathidexia marioburgosi|DHJPAR0019613|615[1n]bp|Talides sinois
Spathidexia marioburgosi|DHJPAR0020920|382[0n]bp|Talides sinois
Spathidexia marioburgosi|DHJPAR0020936|381[0n]bp|Talides sinois
Spathidexia marioburgosi|DHJPAR0022951|421[0n]bp|Talides sinois
Spathidexia marioburgosi|DHJPAR0056161|670[0n]bp|Talides Burns02
Spathidexia marioburgosi|DHJPAR0011559|603[8n]bp|Talides sinois
Spathidexia marioburgosi|DHJPAR0022917|544[13n]bp|Talides sergestus
Spathidexia marioburgosi|DHJPAR0022928|575[38n]bp|Talides sergestus
Spathidexia marioburgosi|DHJPAR0022885|582[20n]bp|Talides sinois
Spathidexia marioburgosi|DHJPAR0016600|600[0n]bp|Talides sinois
Spathidexia marioburgosi|DHJPAR0022860|576[6n]bp|Talides Burns04
Spathidexia marioburgosi|DHJPAR0030427|658[1n]bp|Talides sinois
Spathidexia marioburgosi|DHJPAR0005463|658[0n]bp|Talides sinois
Spathidexia marioburgosi|DHJPAR0022862|657[1n]bp|Talides sinois
Spathidexia marioburgosi|DHJPAR0022861|640[0n]bp|Talides Burns04
Spathidexia marioburgosi|DHJPAR0022863|637[0n]bp|Talides sinois
Spathidexia marioburgosi|DHJPAR0020938|655[0n]bp|Talides Burns02
Spathidexia marioburgosi|DHJPAR0015271|388[0n]bp|Talides sergestus
Spathidexia marioburgosi|DHJPAR0019550|551[6n]bp|Talides sergestus
Spathidexia marioburgosi|DHJPAR0022935|421[0n]bp|Talides sergestus
Spathidexia marioburgosi|DHJPAR0005454|631[1n]bp|Talides sergestus
Spathidexia marioburgosi|DHJPAR0022936|421[0n]bp|Talides Burns02
Spathidexia marioburgosi|DHJPAR0036458|657[0n]bp|Talides sergestus
Spathidexia marioburgosi|DHJPAR0022923|618[7n]bp|Talides Burns04
Spathidexia marioburgosi|DHJPAR0016073|615[1n]bp|Talides sinois
Spathidexia marioburgosi|DHJPAR0022921|613[3n]bp|Talides sinois
Spathidexia marioburgosi|DHJPAR0029994|657[0n]bp|Talides sinois
Spathidexia marioburgosi|DHJPAR0022925|657[0n]bp|Talides sinois
Spathidexia marioburgosi|DHJPAR0022912|657[0n]bp|Talides sinois
Spathidexia marioburgosi|DHJPAR0019711|657[1n]bp|Talides sinois
Spathidexia marioburgosi|DHJPAR0022911|657[3n]bp|Talides sinois
Spathidexia marioburgosi|DHJPAR0022884|620[4n]bp|Talides sinois
Spathidexia marioburgosi|DHJPAR0022883|617[4n]bp|Talides sergestus
Spathidexia marioburgosi|DHJPAR0029721|421[0n]bp|Talides sinois
Spathidexia marioburgosi|DHJPAR0029720|421[0n]bp|Talides sergestus
Spathidexia marioburgosi|DHJPAR0029719|421[0n]bp|Talides sergestus
Spathidexia marioburgosi|DHJPAR0022944|421[0n]bp|Talides Burns04
Spathidexia marioburgosi|DHJPAR0022943|421[0n]bp|Talides sinois
Spathidexia marioburgosi|DHJPAR0019710|656[15n]bp|Talides sinois
Spathidexia marioburgosi|DHJPAR0022941|370[0n]bp|Talides sergestus
Spathidexia marioburgosi|DHJPAR0022942|350[0n]bp|Talides Burns04
Spathidexia marioburgosi|DHJPAR0022952|381[0n]bp|Talides sergestus
Spathidexia marioburgosi|DHJPAR0034332|407[0n]bp|Talides sinois
Spathidexia marioburgosi|DHJPAR0055656|658[0n]bp|Talides sinois
